# Synthesis and Structure–Activity Relationships of Novel Non-Steroidal CYP17A1 Inhibitors as Potential Prostate Cancer Agents

**DOI:** 10.3390/biom12020165

**Published:** 2022-01-20

**Authors:** Tomasz M. Wróbel, Oksana Rogova, Katyayani Sharma, Maria Natalia Rojas Velazquez, Amit V. Pandey, Flemming Steen Jørgensen, Frederic S. Arendrup, Kasper L. Andersen, Fredrik Björkling

**Affiliations:** 1 Department of Drug Design and Pharmacology, University of Copenhagen, Universitetsparken 2, DK-2100 Copenhagen, Denmark; oksana.rogova@chem.lu.se (O.R.); fsj@sund.ku.dk (F.S.J.); fb@sund.ku.dk (F.B.); 2 Department of Synthesis and Chemical Technology of Pharmaceutical Substances, Medical University of Lublin, Chodźki 4a, 20093 Lublin, Poland; 3 Division of Pediatric Endocrinology, Department of Pediatrics, University Children’s Hospital Bern, 3010 Bern, Switzerland; katyayani.sharma@dbmr.unibe.ch (K.S.); maria.rojasvelazquez@students.unibe.ch (M.N.R.V.); amit@pandeylab.org (A.V.P.); 4 Department of Biomedical Research, University of Bern, 3010 Bern, Switzerland; 5 Biotech Research and Innovation Centre (BRIC), University of Copenhagen, Ole Maaløes Vej 5, DK-2200 Copenhagen, Denmark; frederic.arendrup@bric.ku.dk (F.S.A.); kasper.andersen@bric.ku.dk (K.L.A.)

**Keywords:** cytochrome P450 17A1, CYP17A1, prostate cancer, enzyme inhibition

## Abstract

Twenty new compounds, targeting CYP17A1, were synthesized, based on our previous work on a benzimidazole scaffold, and their biological activity evaluated. Inhibition of CYP17A1 is an important modality in the treatment of prostate cancer, which remains the most abundant cancer type in men. The biological assessment included CYP17A1 hydroxylase and lyase inhibition, CYP3A4 and P450 oxidoreductase (POR) inhibition, as well as antiproliferative activity in PC3 prostate cancer cells. The most potent compounds were selected for further analyses including in silico modeling. This combined effort resulted in a compound (comp **2**, IC_50_ 1.2 µM, in CYP17A1) with a potency comparable to abiraterone and selectivity towards the other targets tested. In addition, the data provided an understanding of the structure–activity relationship of this novel non-steroidal compound class.

## 1. Introduction

Prostate cancer (PCa) remains the most common type of cancer diagnosed in men [1]. While the localized disease can be treated with surgery or radiation therapy, androgen deprivation therapy (ADT) is engaged when cancer spreads. ADT continues to be the cornerstone of prostate cancer treatment. Despite treatment, eventually, the disease develops into castration-resistant prostate cancer (CRPC), consequently yielding a poor patient prognosis.

Current discovery efforts towards new therapies focus on androgen receptor (AR) signaling. Those endeavors introduced the next-generation AR antagonists represented by enzalutamide and apalutamide [2]. Other notable discoveries include proteolysis-targeting chimeras (PROTACs), poly ADP-ribose polymerase (PARP) [3] inhibitors, histone deacetylase (HDAC) inhibitors [4], and various forms of immunotherapy [5]. The emergence of novel targets such as fatty-acid binding protein 5 (FABP5) [6] also illustrates the progress that has been made in the field of prostate cancer. However, despite these efforts, PCa still presents a significant problem.

In addition to the interventions mentioned above, cytochrome P450 17A1 (CYP17A1) inhibition has in recent years received increased attention as a valid treatment modality. CYP17A1 is a dual-function oxygenase membrane-bound enzyme that catalyzes the biosynthesis of steroids [7,8,9]. Its dual activity stems from the ability to produce precursors for glucocorticoids via 17α-hydroxylase reaction and androgens/estrogens via 17,20-lyase reaction [10]. Therefore, CYP17A1 is an attractive target for the treatment of prostate cancers that proliferate in response to androgens [11]. CYP17A1 is required in both the “classic” and “back-door” pathways of steroid biosynthesis, and by its inhibition, the production of androgens can be limited [12,13].

**Figure 1:**
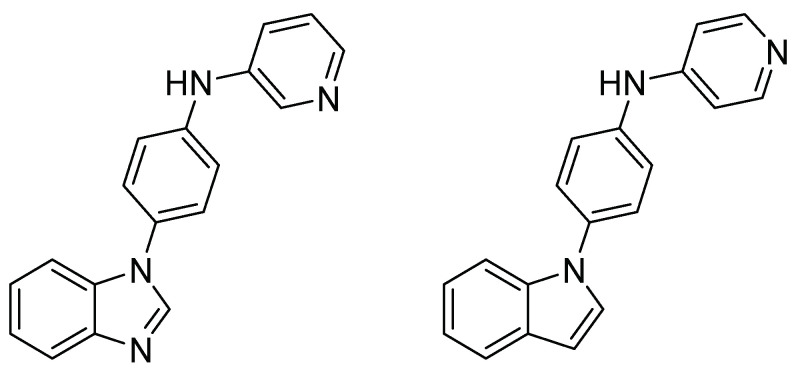
Early hit compounds identified in the previous study.

In our previous work, we identified hit compounds based on a benzimidazole/indole scaffold with satisfactory properties (Figure 1) [14]. Encouraged by these early results, we set off to explore related analogs in search of an optimized molecule and to analyze the structure–activity relationships. Here, we present the synthesis of 20 novel derivatives and their biological evaluation together with computational analysis. The compounds were designed to determine how the following modifications influence the inhibitory activity of CYP17A1, and compound properties: (a) substituent on benzimidazole moiety, to increase compound polarity; (b) introduction of selected heterocycles; (c) replacing linker nitrogen atom with oxygen bridging the aromatic moieties; (d) introduction of more sp^3^ carbons to improve physico-chemical properties; (e) addition of bulk to the middle-linker part of the molecules to take advantage of hydrophobic space in a binding pocket. In addition, assays with PC3 cells, which display a high degree of metastatic potential and tumorigenicity, were performed to see if the compounds would affect hormone-insensitive cells, thus implying an additional mechanism of action.

## 2. Materials and Methods

### 2.1. Synthesis

All reagents and solvents were used as purchased from commercial sources and reactions were carried out under anhydrous and air-free conditions unless stated otherwise. Reaction conditions and yields were not optimized. Dry column vacuum chromatography (DCVC) was performed with silica gel 60 (15–40 µm, Merck KGaA, Darmstadt, Germany). Ion exchange chromatography was performed on ISOLUTE^®^ MP-TsOH columns (sulfonated macroporous polystyrene resin, 500 mg, 6 mL, Biotage, Uppsala, Sweden). ^1^H and ^13^C spectra were recorded on 600 MHz Bruker Avance III HD, 400 MHz WB Bruker Avance, or 300 MHz Bruker Fourier spectrometers (Bruker, Billerica, MA, USA). Coupling constants (J) are reported in Hertz (Hz). Chemical shifts are reported in parts per million (ppm, δ scale) relative either to an internal standard (TMS) or residual solvent peak. High-resolution mass spectroscopy (HRMS) was carried out on a Bruker Solarix XR 7T ESI/MALDI-FT-ICR with positive MALDI ionization mode using NaTFA cluster-ions for external calibration or Bruker microTOF-Q II with ESI ion source mass spectrometers (Bruker, Billerica, MA, USA). Data obtained were processed in Bruker DataAnalysis Software. Analytical HPLC was carried out on Dionex UltiMate HPLC system (Thermo-Fisher, Waltham, MA, USA) consisting of LPG-3400A pump, WPS-3000SL autosampler, and DAD-3000D diode array detector using Gemini-NX C18 column (4.6 mm × 250 mm, 3 µm, 110 Å) or Kinetex PS18 column (2.1 mm × 100 mm, 2.6 µM, 100 Å). Preparative HPLC was carried out on a Dionex UltiMate HPLC system consisting of HPG-3200BX pump, Rheodyne 9725i injector, 10 mL loop, MWD-3000 detector, and FCA_Multi automated fraction collector using Gemini-NX C18 (21.2 mm × 250 mm, 5 µm, 110 Å). Data were acquired and processed using the Chromeleon software (Thermo Fisher Scientific).

### 2.2. Assays

Assay of CYP17A1 activities: Assay of CYP17A1 17α-hydroxylase and 17,20 lyase activities were performed as described previously [15,16]. The 17α-hydroxylase activity was measured by conversion of Prog to 17OH-Prog, while production of DHEA from 17OH-Preg was used for monitoring the 17,20 lyase activity of CYP17A1. For the 17-hydroxylase reaction, radiolabeled [^14^C]-PROG (20,000 cpm/reaction) was used as a tracer. Steroids were extracted and separated by thin-layer chromatography (TLC) on silicagel (SIL G/UV_254_) TLC plates (Macherey-Nagel, Oensingen, Switzerland) as previously described [16,17,18]. The separated steroids were visualized on a Fuji FLA-7000 PhosphoImager (Fujifilm, Dielsdorf, Switzerland) and quantified using Multi Gauge software (Fujifilm, Dielsdorf, Switzerland). Steroid conversion was calculated as a percentage of incorporated radioactivity into a specific steroid product to total radioactivity measured for the whole sample (internal control). For the 17,20 lyase activity of CYP17A1, a water release assay was used. We used 17OH-Preg labeled with [^3^H] at the C21 position, which upon conversion to DHEA releases an equimolar amount of [^3^H] H_2_O. Tritiated water is separated and measured by scintillation counting based on a method described by Simpson for the assay of aromatase activity [19].

Assay of CYP3A4 activity: The activity of the major drug-metabolizing enzyme CYP3A4 in the presence of selected compounds was tested using the fluorogenic substrate BOMCC (7-Benzyloxy-4-trifluoromethylcoumarin) (Invitrogen Corp, Carlsbad, CA, USA) as described earlier [20]. In vitro CYP3A4 assays were performed using a liposome system consisting of POR, CYP3A4 and cytochrome b_5_ at a ratio of 4:1:1 (POR:CYP3A4:b_5_). Reconstitution of liposomes was carried out as described before [21]. The final assay mixture consisted of liposomes and proteins (80 pmol POR: 20 pmol CYP3A4: 20 pmol b_5_), 2.5 mM MgCl_2_, 2.5 μM GSH and 20 μM BOMCC in 50 mM HEPES buffer and the reaction volume was 200 μL. The catalytic reaction was initiated by the addition of NADPH to 1 mM final concentration and fluorescence was monitored on a Spectramax M2e plate reader (Molecular Devices, Sunnyvale, CA, USA) at an excitation wavelength of 415 nm and emission wavelength of 460 nm for BOMCC metabolism.

Assay of P450 oxidoreductase (POR) activity: POR assay was performed as described previously, using purified human POR [22]. The activity of the bacterially expressed POR was tested for its ability to reduce resazurin into resorufin, which is highly fluorescent. The reaction was performed in triplicate in 96-well format using a microplate reader (Molecular Devices, Sunnyvale, CA, USA). The reaction components were 10 μM resazurin, 100 ng of POR in 50 mM Tris-HCL (pH 7.8) containing 150 mM NaCl. NADPH was added at a concentration of 100 μM as the source of electrons to start the reaction, and the change in emission (570 nm excitation, 585 nm emission for resorufin) was monitored for 30 min.

Antiproliferation assay: PC-3 prostate cancer-derived cells were propagated in RPMI-1640, GlutaMAX + 25 mM HEPES (Gibco) supplemented with 100 U/mL penicillin, 100 mg/mL streptomycin (P/S) (Gibco), and 6% (PC-3) fetal bovine serum (FBS) (HyClone). Cells were grown to approximately 80% confluence and harvested by 0.25% Trypsin/EDTA (Gibco) treatment. The released cells were counted and seeded in 384-well plates (Falcon, ref. 353962). The cells were allowed to settle for 24 h, after which cell numbers in 44 wells/cell line of one plate were determined (T_0_). In parallel, the indicated compounds (10 mM stock in DMSO) or DMSO alone were delivered to cells in three replicate 384-well plates by acoustic droplet ejection using an Echo 550 Liquid Handler (Labcyte) for a final concentration of 0–25 µM compound and 0.25% DMSO. Cells were then incubated for an additional 72 h before determining end-point cell numbers. CellTiter-Glo 2.0 (Promega) luminescent cell viability assay was used as a proxy for cell number according to manufacturer instructions. Luminescence was measured with a 384-well plate reader on a SpectraMax Paradigm using Softmax Pro 6.5.1 software. Liquid handling in cell seeding and CellTiter GLO addition was performed on a MicroLab STARlet liquid handling workstation with a CO-RE 384 probe head (Hamilton Company, Reno, NV, USA).

### 2.3. Molecular Modeling

For the cross-docking, the ligands were extracted from their respective protein structures and prepared for docking by the LigPrep procedure in Maestro (v. 9.8, Schrodinger 2018-3 release, Schrödinger, LLC, New York, NY, USA, 2014) securing proper atom and bond typing, protonation and partial atomic charges [23]. The remaining compounds were constructed in Maestro and prepared for docking in a similar way. The proteins were extracted from the Protein DataBank [24] and subjected to the Protein Preparation procedure [23] to secure proper protonation and hydrogen bonding.

The GOLD (Genetic Optimization for Ligand Docking) program version 5.6 [25] was used for docking. Binding sites were defined by a 20 Å sphere centered at the Fe atom in the heme group. Ligands were docked with the slow genetic algorithm using the CYP450 version of the ChemScore scoring function [26,27]. Ten poses were sampled and analyzed for each docking.

The MD simulations were performed with the Desmond program (version 3.6, Schrödinger, LLC) using the OPLS4 force field [28]. The protein-ligand complexes were embedded in an orthorhombic box of SPC water molecules with a 10 Å buffer size between protein and box boundary using the Desmond system builder. The MD systems comprised approximately 70,000 atoms, including approximately 7500 atoms for the protein including the heme group, 36 atoms for the ligand, one chloride ion to neutralize the system, and approximately 21,000 water molecules. The systems were equilibrated prior to the production runs applying the Desmond default equilibration protocol. Subsequently, the systems were simulated for 100 ns and 100 frames collected.

## 3. Results and Discussion

### 3.1. Synthesis

**Scheme 1:**
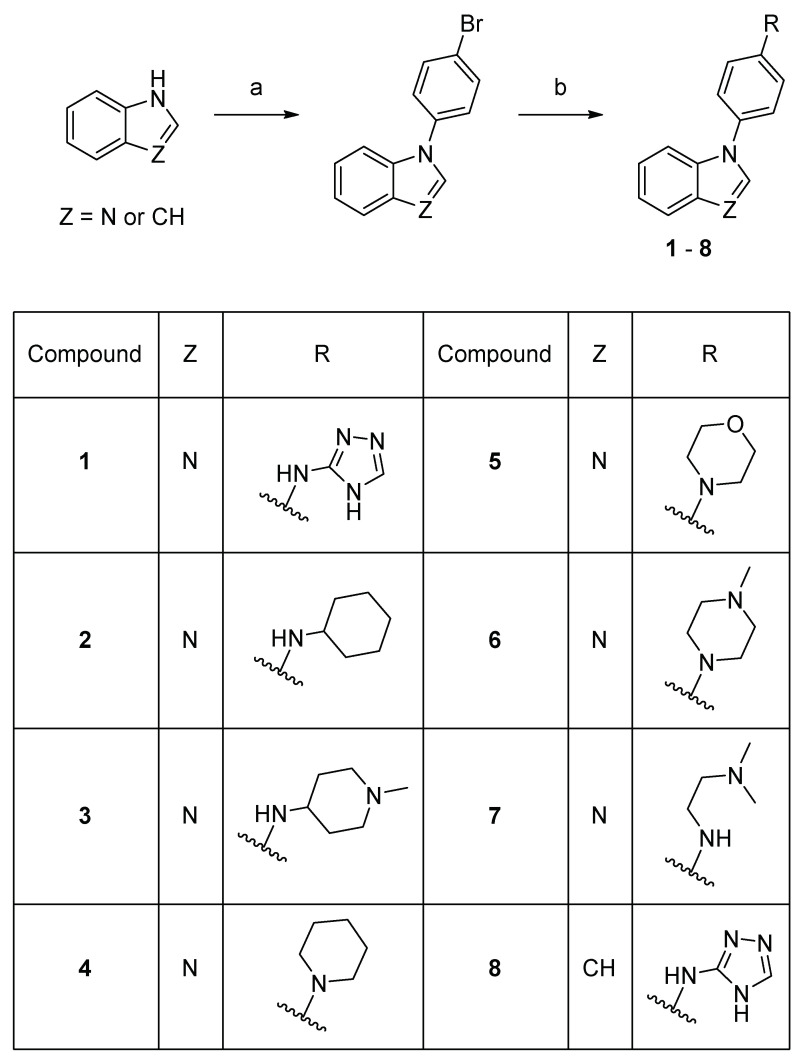
Synthesis of compounds **1**–**8**. Reaction conditions: (a) 1-bromo-4-fluorobenzene, K_3_PO_4_, DMF, 150–160 °C, 53–78%; (b) amine, precatalyst Pd G1 or G3, tBuXPhos, NaOtBu, THF or tBuOH, 21–73%.

**Scheme 2:**
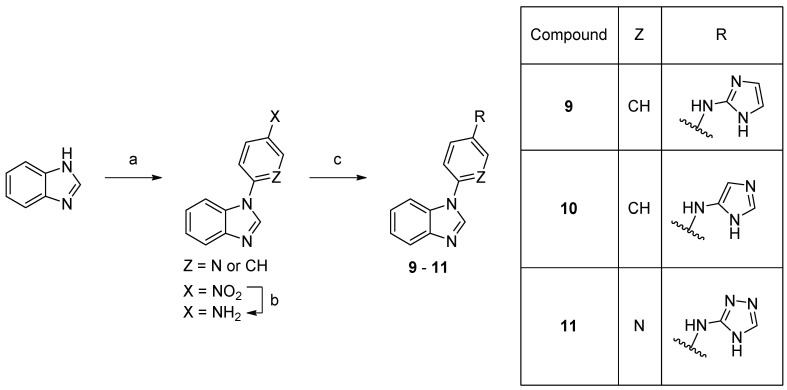
Synthesis of compounds **9**–**11**. Reaction conditions: (a) 1-fluoro-4-nitrobenzene, K_3_PO_4_, DMF 150 °C, 66% or 2-fluoro-5-nitropyridine, K_3_PO_4_, DMSO, rt, 74%; (b) 10% Pd/C, MeOH, rt, 82%; (c) bromide, precatalyst Pd G3, tBuBrettPhos, LHMDS, THF, 60 °C, 4–15%.

**Scheme 3:**
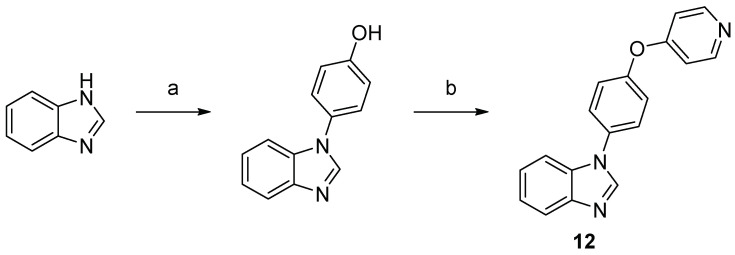
Synthesis of compound **12**. Reaction conditions: (a) 4-hydroxyphenylboronic acid, O_2_, Cu_2_S, TMEDA, MeOH, rt, 71%; (b) 4-bromopyridine hydrochloride, NaOtBu, DMF, 150 °C, 32%.

**Scheme 4:**
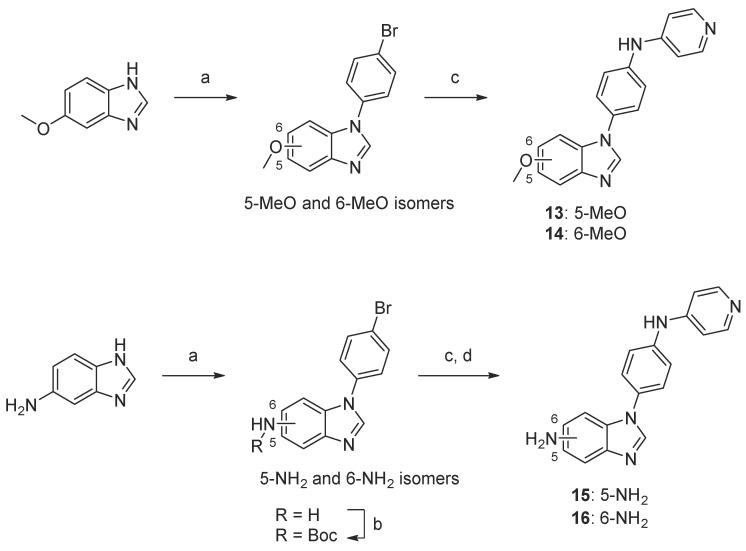
Synthesis of compounds **13**–**16**. Reaction conditions: (a) 1-bromo-4-fluorobenzene, K_3_PO_4_, DMF, 160 °C, 5–44%; (b) Boc_2_O, TEA, tBuOH, 40 °C, 74–90%; (c) 4-aminopyridine, precatalyst Pd G1, tBuXPhos, NaOtBu, tBuOH, 70 °C, 11–50%; (d) TFA, DCM, 23–50% (over two steps, c and d).

**Scheme 5:**
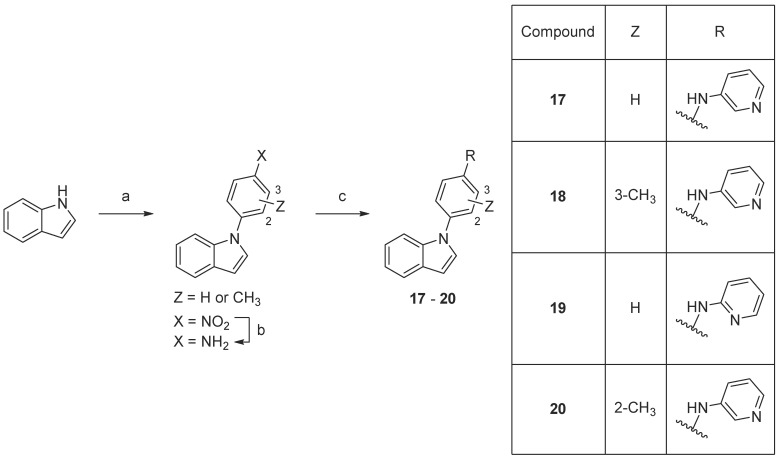
Synthesis of compounds **17**–**20**. Reaction conditions: (a) 1-fluoro-4-nitrobenzene or fluoronitrotoluene, K_3_PO_4_, DMF, 160 °C, 18–93%; (b) 10% Pd/C, MeOH, rt, 91–96%; (c) 2- or 4-aminopyridine, precatalyst Pd G3, tBuXPhos, NaOtBu, THF, MW 100 °C, 23–32%.

A total of 20 new compounds were synthesized using the previously described method [14]. Briefly, indole or benzimidazole were N-arylated and the resulting bromide intermediate was subjected to Buchwald–Hartwig coupling with amines to obtain compounds **1**–**8** (Scheme 1). Access to compounds **9**–**11** was realized via intermediates that had reversed polarity for Buchwald–Hartwig coupling (Scheme 2). Using heterocyclic bromides, as coupling partners, required switching to tBuBrettPhos as a ligand and LHMDS as a base. Compound **12** was obtained from a phenol intermediate, which was synthesized employing Chan–Lam reaction (Scheme 3). Since different tautomeric forms of 5-substituted benzimidazole may react, two distinct isomers can be obtained [29]. Indeed, after transforming 5-methoxybenzimidazole into its N-arylated derivative, two regioisomers (5-methoxy and 6-methoxy) readily separable by chromatography were produced. Similar results were obtained with 5-aminobenzimidazole. Structures of these isomers were identified by NMR spectroscopy employing NOE experiments. Eventually, these intermediates were converted into the final compounds **13**–**16** via established chemistry. Coupling of compounds **15** and **16** required prior protection of the amino group, which was achieved with the Boc group (Scheme 4). Compounds **17**–**20** were synthesized in a similar fashion utilizing nitro compounds for the first step arylation, followed by catalytic reduction and finally Buchwald–Hartwig coupling (Scheme 5).

The final compounds were fully characterized by ^1^HNMR and ^13^CNMR spectroscopy, as well as HRMS spectrometry, and were found to be >99% pure (HPLC). Detailed synthetic procedures, spectra, and HPLC chromatograms can be found in Supplementary Materials.

### 3.2. Biological Evaluation

**Figure 2:**
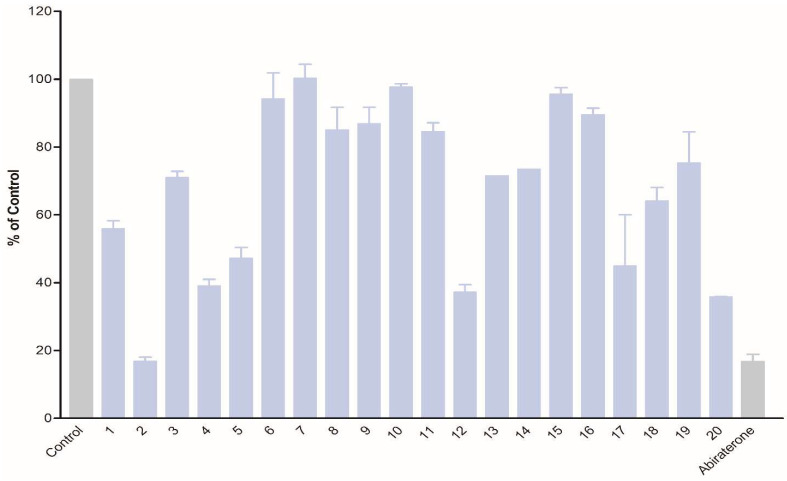
Inhibition of CYP17A1 17α-hydroxylase activity by compounds **1**–**20** at 10 µM concentration.

**Figure 3:**
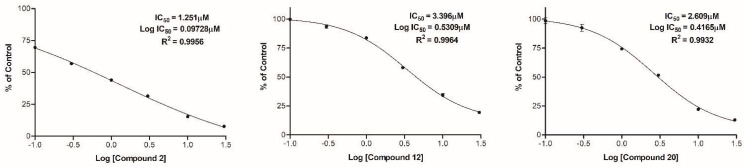
Determination of IC_50_ of compounds **2**, **12,** and **20** for inhibition of the 17α-hydroxylase activity of CYP17A1.

**Figure 4:**
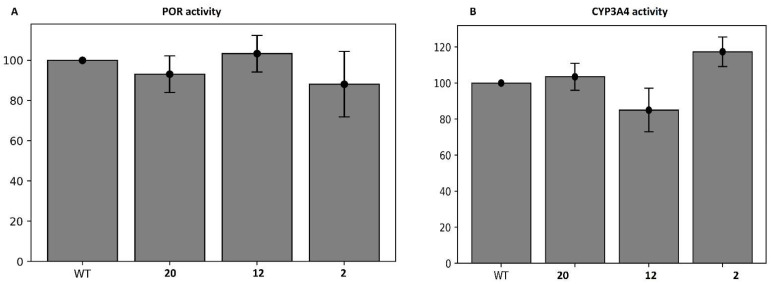
The activity of compounds **2**, **12,** and **20** towards POR (**A**) and CYP3A4 (**B**).

**Figure 5:**
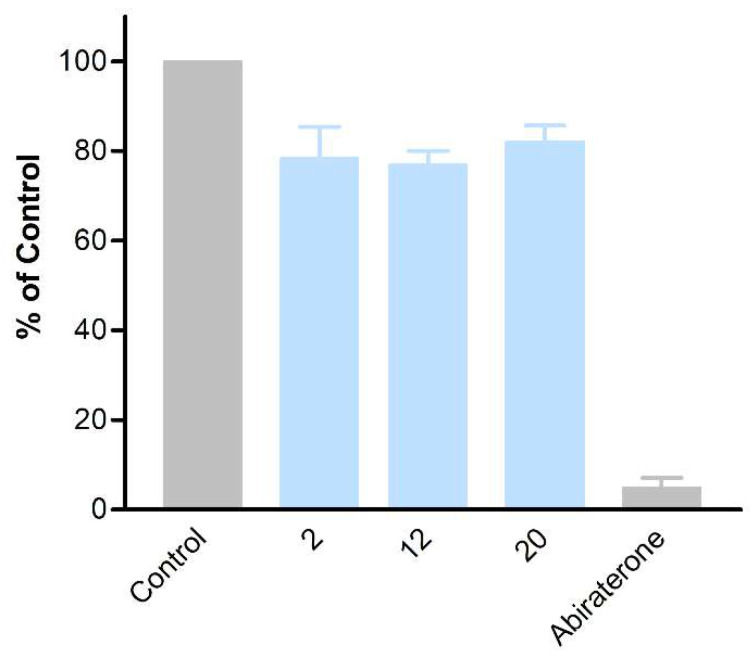
Inhibition of CYP17A1 lyase reaction by compounds **2**, **12,** and **20**.

The obtained compounds were subjected to initial screening for inhibition of CYP17A1 activity at a fixed concentration (10 µM) (Figure 2). Most compounds did not display improved activity over the previous hits [14]. However, we observed substantial activity in the case of compounds **2**, **12,** and **20**. Compound **2** was the most potent and comparable to abiraterone used as a reference compound in this assay. These three most potent compounds were selected for more rigorous testing comprising determination of IC_50_, selectivity towards CYP isoform CYP3A4, the ability to inhibit cytochrome P450 reductase (POR) and the ability to inhibit the lyase reaction of CYP17A1. Compounds **2**, **12** and **20** displayed IC_50_ of 1.2, 3.4 and 2.6 µM, respectively, in the inhibition of the CYP17A1 17α-hydroxylase activity (Figure 3). All three selected compounds exhibited selectivity vs. CYP3A4 (**2**: 104 ± 4%, **12**: 75 ± 5% and **20**: 117 ± 8% compared to untreated control) and importantly they do not appear to inhibit POR (**2**: 93 ± 9%, **12**: 103 ± 9% and **20**: 88 ± 16% compared to untreated control) (Figure 4), suggesting a targeted activity towards CYP17A1. Finally, checking compounds **2**, **12** and **20** against the lyase reaction, unfortunately, revealed little influence compared to abiraterone (**2**: 78 ± 10%, **12**: 77 ± 4% and **20**: 82 ± 5% of untreated control, compared to 5 ± 2% activity observed for abiraterone) (Figure 5).

These results highlight several features regarding the structural composition of the compounds. Introduction of the cyclohexane ring in **2** had the most profound effect and **2** showed similar potency to abiraterone. The presence of an aliphatic ring presumably allows for better allocation in the hydrophobic binding region. This is further corroborated when comparing **2** to **3**. Both compounds have similar shapes, but compound **3** contains a protonable amine embedded into cyclohexane. This modification causes a significant loss of activity. Compound **7,** which contains a flexible chain ending with a tertiary amine is devoid of any activity, indicating that the presence of a ring system is a necessary requirement. When the ring system is directly connected to the benzene linker, the activity is attenuated as observed with compounds **4** and **5**, the effect being more pronounced in the latter. Similar to the pattern observed with compound **3** in relation to compound **2**, we observed a sharp drop in activity when an amine moiety was introduced, evidenced by the low activity of compound **6**. Embedding polar fragments and increasing the number of sp^3^ carbons was aimed at improving the drug-likeness of our molecules [30]. Interestingly, replacing the nitrogen atom, primarily serving as a part of a linker, with an oxygen atom gave compound **12**, which was among the three most potent compounds investigated in this work. Here, we hypothesize that an analog containing a 3-pyridyl fragment instead of 4-pyridyl would have been even more potent as corroborated by computational analyses. Unfortunately, this analog was not obtained due to difficulty in chemical synthesis. Exploration of different heterocyclic ring systems did not deliver compounds with significant potency. However, it is noteworthy that in those cases a benzimidazole fragment was superior to indole, as evidenced when comparing the activity of **1** to the activity of **8**. Upon considering the structure of a five-membered ring attached to the linker, it is evident that a molecule with triazole fragment (**1**) exerts stronger inhibitory activity than molecules with imidazole (**9** and **10**). Comparison of two imidazole-containing compounds suggests that 2-imidazolyl (**9**) is preferred to 4-imidazolyl (**10**), although both compounds displayed very weak activity. The importance of having a more hydrophobic middle part of a molecule is indicated by compound **11**. This nitrogen-rich molecule has pyridine as the linker and displayed decreased activity compared to its benzene analog **1**. Compounds **13**–**16** were designed to explore the effect of substituting benzimidazole with various polar groups. It was hoped that by doing so we would make possible hydrogen bonding to Asn202 in helix F, as observed in the X-ray structure of CYP17A1 bound to abiraterone [7]. This operation did not bring a significant boost in the activity. However, it can be noted that the methoxy group (**13** and **14**) was a better choice than the amino group (**15** and **16**), regardless of the substitution pattern present in these two pairs of analogs. Lastly, we wanted to explore the effect of adding an extra methyl group to the benzene linker, taking advantage of the hydrophobic region of the binding site. The so-called “magic methyl” effect, which involves the addition of a single methyl group in the “right” location on a molecule can result in a significant activity boost [31,32]. In our case, we were able to obtain compounds **18** and **20** bearing this substituent. Interestingly, only **20** displayed significant activity. This compound has a methyl group that is ortho to the indole ring and is the second most potent compound in this series. In comparison, compound **18** was less active. In that case, the methyl group is meta to the indole ring, which seemingly has less influence and conformational benefit, as evidenced by our computational analysis. The activities of compounds **17** and **19** deliver further rationale for designing compounds with a 3-pyridyl substitution pattern instead of 2-pyridyl. As shown by our calculations and previous studies, compounds with a 2-pyridyl moiety adopt unfavourable binding poses.

Although we were unable to obtain compounds with significantly improved activities compared to the initial hits [14], we were nevertheless happy to observe that our design was still selective for CYP17A1 versus CYP3A4, as indicated by the assay performed on the three most potent compounds (Figure 4B). CYP3A4 is the major drug-metabolizing enzyme in the liver that has broad specificity and can also metabolize steroids [33]. A severe impact on CYP3A4 is, therefore, considered a negative criterion for compounds targeting another P450 enzyme. While CYP3A4 itself does not show much variability in humans, like CYP2D6 and CYP2C19, variations in POR have been shown to alter the activity of CYP3A4 with potentially negative consequences [20,21,33,34]. Furthermore, these compounds also do not influence POR activity (Figure 4A). This is important because all microsomal P450s depend on POR for the supply of electrons and severe disruption of POR activities may affect all microsomal P450 enzymes with serious consequences [35]. Recently, the modulation of some P450 activities by binding to POR has been reported, indicating that a direct impact on the conformation or activity of POR may also influence P450 enzymes [22].

**Figure 6:**
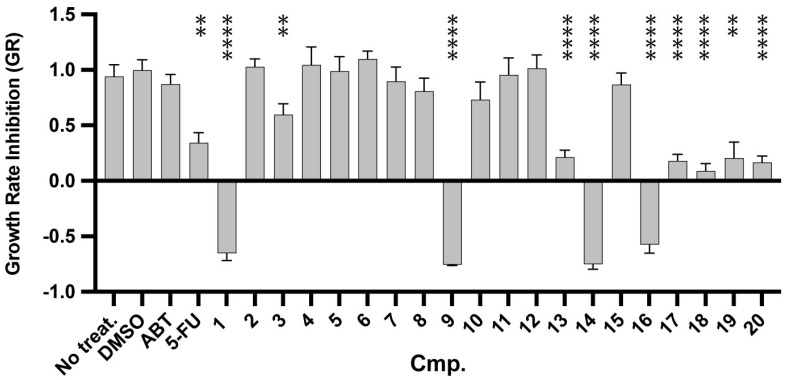
Growth rate inhibition (GR) of compounds **1**–**20**. All compounds were tested at 25 µM, with DMSO as a control and 5-fluorouracil (5-FU) and abiraterone (ABT) as reference compounds. The sign of the GR value relates directly to response phenotype: Values between 0 and 1 show partial growth inhibition, a value of 0 equals cytostasis, and values between 0 and −1 show that compounds are cytotoxic. Significant differences of growth in presence of compounds compared to DMSO were determined by the T-test and indicated by asterisks: ** *p* < 0.01, **** *p* < 0.0001.

Since our research efforts are aimed at prostate cancer, we were also interested in evaluating our compounds in the PC3 prostate cancer cell line. This cell line is hormone insensitive and presents no AR [36]; thus, any effect observed on cell growth might be attributed to additional mechanisms of action. Indeed, we observed a negative growth rate inhibition (GR) value for compounds **1**, **9**, **14,** and **16** when tested at 25 µM concentration (Figure 6, Supplementary Materials). The negative GR values indicate that the compounds are cytotoxic [37]. These compounds did not exhibit significant inhibition of CYP17A1; therefore, it is likely that they exert a different mechanism of action responsible for the observed cytotoxicity. They were more cytotoxic than the DNA replication inhibitor 5-fluorouracil used as a reference compound in this assay. Moreover, abiraterone, which was also used as a reference compound, had a very small effect on PC3 proliferation.

### 3.3. Molecular Modeling

The three compounds, **2**, **12,** and **20**, showing the highest affinity for the CYP17A1 enzyme were subjected to a molecular modeling analysis to determine their potential binding mode. The compounds represent different combinations of ring systems yielding three different scaffolds. Compounds **2** and **20** only contain a single potential heme-coordinating moiety, a benzimidazole and a pyridine moiety, respectively. Compound **12** contains both these ring systems and, accordingly, this compound may bind to CYP17A1 in two different modes.

We selected the five recently determined experimental structures of the A105L mutant of CYP17A1 as the target for our docking studies since they represent structures of CYP17A1 in complex with an inhibitor, abiraterone (4NKV), two hydroxylase substrates, pregnenolone (4NKW) and progesterone (4NKX), and two lyase substrates, 17α-hydroxyprogesterone (4NKY) and 17α-hydroxypregnenolone (4NKZ) [38].

**Figure 7:**
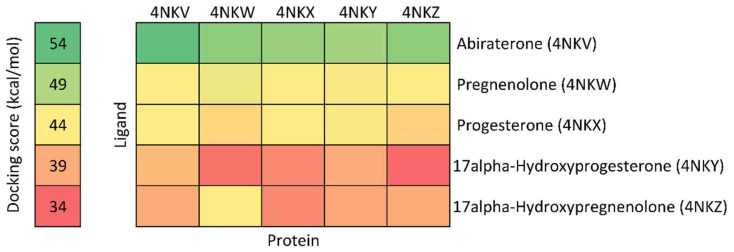
Heat map showing the docking scores for cross-docking of substrates into the experimentally determined CYP17A1 structures.

Initially, we performed a cross-docking study of the five substrates in each of the protein structures to test if the docking software was able to identify the correct binding mode for these ligands. Inspection of the docking scores (Figure 7) shows that abiraterone binds significantly better (>10 kcal/mol) to all five protein structures. It is also interesting to note that the hydroxylase substrates generally bind better than the lyase substrates. The high degree of similarity in binding scores between the five proteins is probably a consequence of the experimental structures being nearly identical.

Initially, we docked all 20 compounds to the five CYP17A1 structures, but since we observed nearly identical results for the five structures, we only describe the results for docking into the 4NKV structure.

**Figure 8:**
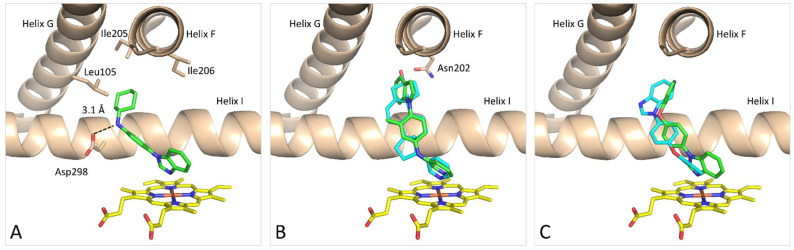
Preferred binding mode predicted by the GOLD docking program of compound **2** (green) (**A**), **20** (green) in comparison with abiraterone (cyan) (**B**) and **12** in two docking poses (pose #1 green and pose #2 cyan) (**C**). Coloring: protein is beige; heme is yellow; and all heteroatoms are colored based on type.

The docking of **2** yields poses with the nitrogen lone pair on the benzimidazole ring pointing towards the Fe atom in the heme moiety (Fe···N = 2.5 Å). The binding mode of **2** is similar to the binding mode we previously identified for the structurally related compound (**1d** in [14]) with the cyclohexane ring located in a shallow hydrophobic cavity formed by Leu105, Ile205, and Ile206, which confirms that the enzyme may accommodate different hydrophobic moieties in this part of the active site (Figure 8A). The benzimidazole ring is also located nearly identical to the benzimidazole ring in the CYP17A1–galeterone complex (PDB 3SWZ) [7]. We also observed hydrogen bonding between Asp298 and the amine situated between the aromatic and aliphatic rings (O···N = 3.1 Å). Hydrogen bonding to Asp298 has also been observed in the structure of (S)-orteronel [39] complexed with CYP17A1 (PDB 5IRQ) [40]. Recently, Fehl et al. designed inhibitors derived from abiraterone with polar substituents on the B ring in the steroid framework, which formed hydrogen bonds to Asp298 (PDB 6CHI and 6CIR) [41].

The docking of **20** showed, as expected, that the pyridine nitrogen coordinated to the Fe atom in the heme group with a Fe···N distance of 2.5 Å (Figure 8B). Comparison with the X-ray structures of the CYP17A1–abiraterone complexes (PDB 3RUK and 4NKV) [7,38] revealed that the pyridine rings in the two structures overlap and that the benzene ring in **20** occupies the same space as the C ring of the steroid moiety of abiraterone. The methyl group in **20** was originally introduced to fill the hydrophobic cavity occupied by the B ring of abiraterone. It is possible that the position of the indole moiety relative to the benzene ring is caused by steric repulsion from that methyl group. Another possibility relies simply on a better fit to the upper part of the cavity. In any case, the indole moiety occupies the same space as the A ring and the C10 methyl group in the abiraterone structure. The docking of **20** also suggests that adding a polar substituent on the indole ring could make hydrogen bonding to Asn202, as observed in the CYP17A1–abiraterone complexes.

Compound **12** contains both a pyridine and a benzimidazole system and, accordingly, two possible binding modes are theoretically possible (Figure 8C). The docking revealed these with moieties coordinating to the Fe in the heme group with Fe···N = 2.4 Å (benzimidazole) and 2.5 Å (pyridine), respectively, referred to as pose #1 and pose #2. For compound **12**, pose #1 the benzimidazole system is located similarly to the benzimidazole system in the galeterone complex (3SWZ) [7], but in the opposite end of the compound, the pyridine is not close enough to make a contact to Asn202 as observed for galeterone. There are distinct differences in the orientation of the pyridine ring in pose #2 and in the abiraterone complexes 3RUK and 4NVK relative to the heme system. The 4-substituted pyridine ring requires more space above the heme group to adopt an ideal orthogonal orientation [42]. It suggests that the 3-substituted pyridine is the preferred moiety relative to the 2- and 4-substituted systems.

**Figure 9:**
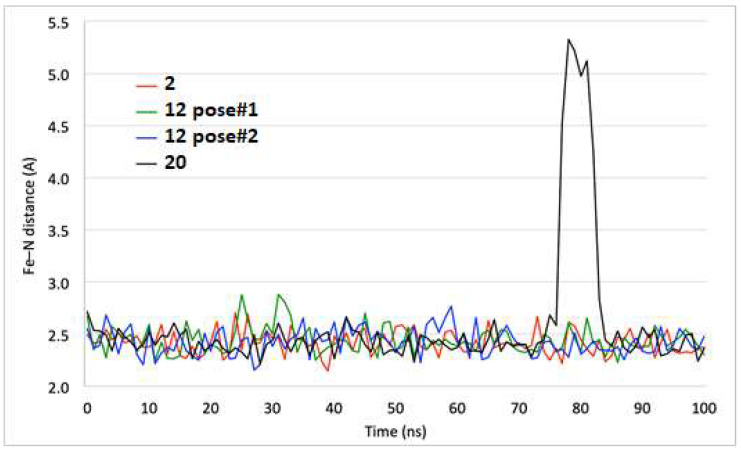
Fe···N distances for the four CYP17A1–ligand complexes during the 100 ns molecular dynamics simulations.

To investigate the stability of the protein–ligand complexes described above, we have subjected these to molecular dynamics (MD) simulations without any constraints, allowing both the protein and the ligands to move freely. The Fe···N distance is an important parameter when considering if the docking poses were realistic. The results of these short MD simulations revealed that all four complexes appeared to be stable (Figure 9). Although the Fe···N distance for **20** displayed extreme values for six frames around 80 ns, where the pyridine ring changed orientation and lost contact with the Fe atom, the original distance on approx. 2.4 Å was quickly re-established. The average Fe···N (benzimidazole) distances are 2.42 ± 0.11 Å and 2.44 ± 0.1 Å for compound **2** and **12**, respectively. The corresponding Fe···N (pyridine) distances are 2.42 ± 0.13 Å and 2.43 ± 0.11 Å for **12** and **20** (excluding the six frames around 80 ns).

The MD simulations confirm that the docking complexes represent low energy structures and may represent likely binding modes for the compounds, which we hope to confirm by ongoing efforts to get crystals and subsequently determine X-ray structures of some of our compounds. Computational analyses indicate that benzimidazole and pyridine fragments are important pharmacophores when considering the design of CYP17A1 binders. In the case of the pyridyl fragment, the preferred substitution pattern is 3-pyridyl over 4-pyridyl, with 2-pyridyl being unable to coordinate the Fe atom in heme effectively.

## 4. Conclusions

A series of compounds based on benzimidazole and indole scaffolds were synthesized and evaluated as inhibitors of CYP17A1 hydroxylase and lyase activity. Selected compounds were further assessed as inhibitors of CYP3A4 and P450 oxidoreductase (POR). Three compounds, **2**, **12,** and **20**, were found to be potent inhibitors of CYP17A1 hydroxylase activity, with an IC_50_ of 1.2, 3.4 and 2.6 µM, respectively. Although they were not able to inhibit the CYP17A1 lyase catalyzed reaction, they were nevertheless selective towards CYP17A1, as indicated by their low influence on CYP3A4 and POR. Compound **2** displayed inhibitory potency comparable to the clinically used compound abiraterone in the CYP17A1 hydroxylase assay. Compounds **1**, **9**, **14** and **16** were cytotoxic towards PC3 cancer cell lines. However, as these compounds were much less potent in the CYP17A1 hydroxylase assay, the cytotoxicity is tentatively mediated through another mechanism of action, not further explored in this study. Finally, analysis of the SAR of the new compounds provided valuable information for further design of this class of compounds.

## Data Availability

The data presented in this study are available in the Supplementary Materials here and are also available on request from the corresponding author.

## Supplementary Materials

The following supporting information can be downloaded at: https://www.mdpi.com/article/10.3390/biom12020165/s1.
