# Supplementary material for: Synthesis and Structure–Activity Relationships of Novel Non-Steroidal CYP17A1 Inhibitors as Potential Prostate Cancer Agents
Source: Biomolecules. 2022 Jan 20;12(2):165. doi: 10.3390/biom12020165 (PMC8961587; doi:10.3390/biom12020165)
Supplement: Supplementary file 1 [file biomolecules-12-00165-s001.zip › biomolecules-1541349-supplementary.pdf]

Synthesis and Structure–Activity Relationships of Novel Non-Steroidal CYP17A1 Inhibitors as Potential Prostate Cancer Agents

Supplementary Information

Contents

General Procedures 2

Scheme S1 Synthetic Details 3

Scheme S2 Synthetic Details 23

Scheme S3 Synthetic Details 37

Scheme S4 Synthetic Details 41

Scheme S5 Synthetic Details 65

HPLC Purity Chromatograms 85

CYP17A1 Hydroxylase, CYP17A1 Lyase, CYP3A4 and POR Inhibition Values 105

PC-3 Whole-Cell Assay GR50 Values 106

References 107

General Procedures

General procedure for processing compounds through an SCX column (catch and release):

A Biotage MP-TsOH SCX column 500 mg/6 mL is conditioned with 4 mL of MeOH. Then, the compound dissolved in the minimum amount of MeOH is loaded onto a column under gravity. The column is washed with 10 mL of MeOH and finally the compound is eluted with 4 mL of 2M NH_3 in MeOH followed by 8 mL of MeOH.

General procedure for Boc deprotection:

A Boc-protected amine is mixed with 1mL of DCM, and 1 mL of TFA is added. The reaction is stirred at room temperature for 10 minutes. Solvent is removed in vacuo and the residue is purified by preparative HPLC.

Scheme S1 Synthetic Details

1-(4-bromophenyl)-1*H*-benzo[*d*]imidazole (2a)

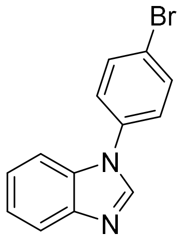

Benzimidazole (5.91 g, 50 mmol), 1-bromo-4-fluorobenzene (9.63 g, 55 mmol) and potassium phosphate (21.23 g, 100 mmol) were stirred in anhydrous DMF (200 mL) overnight at 160 °C. Reaction was partitioned between 100 mL of DCM and 400 mL of water. Aqueous was extracted twice with 100 mL of DCM, and combined organics were washed three times with 100 mL of water and dried with anhydrous $MgSO_4$. Crude was purified by column chromatography eluting with heptane/EtOAc (50:50) and recrystallized from EtOAc/heptane (1:1) to obtain the title compound as a white solid (10.6 g, 78%).

^{1}H NMR (600 MHz, DMSO-d_6) δ 8.58 (s, 1H), 7.85 – 7.77 (m, 3H), 7.70 – 7.65 (m, 2H), 7.65 – 7.61 (m, 1H), 7.34 (pd, J = 7.2, 1.4 Hz, 2H). ^{13}C NMR (151 MHz, DMSO-d_6) δ 143.8, 143.1, 135.2, 132.9, 132.9, 132.9, 132.8, 132.8, 125.7, 125.6, 123.5, 122.5, 120.2, 119.9, 110.5.

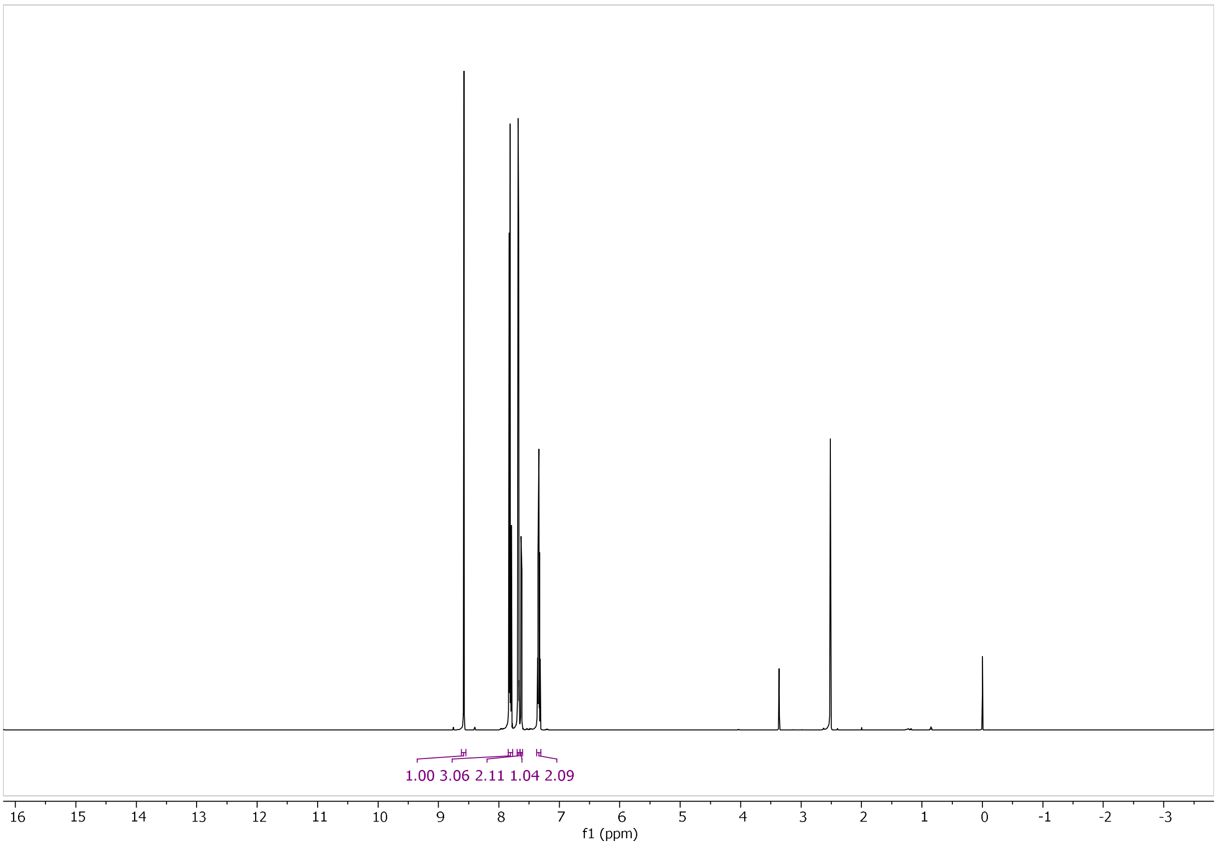

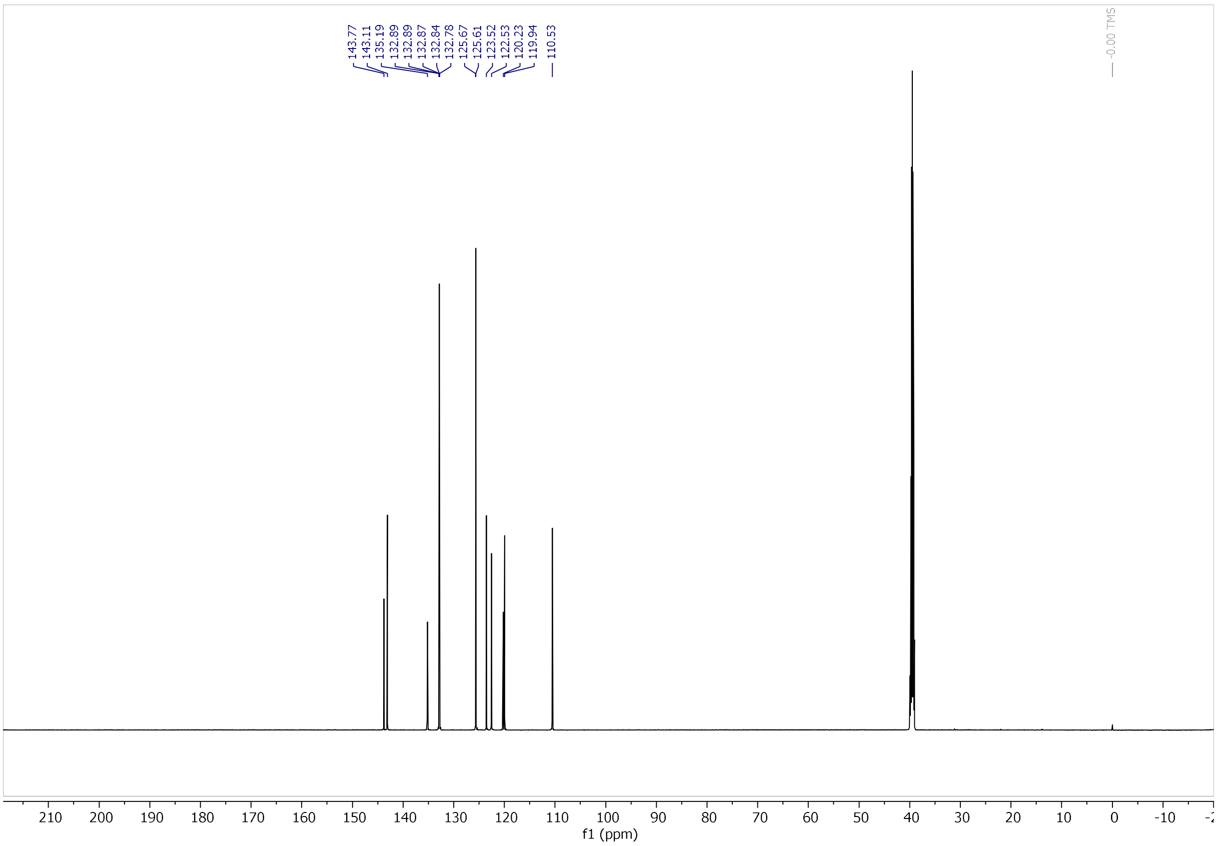
143.77
143.11
135.19
132.89
132.89
132.87
132.84
132.78
125.67
125.61
123.52
122.53
120.23
119.94
110.53
-0.00 TMS
210 200 190 180 170 160 150 140 130 120 110 100 90 80 70 60 50 40 30 20 10 0 -10
f1 (ppm)

1-(4-bromophenyl)-1H-indole (2b)

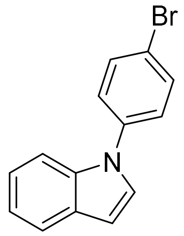

Indole (1.76 g, 15 mmol), 1-bromo-4-fluorobenzene (5.25 g, 30 mmol) and potassium phosphate (15.92 g, 75 mmol) were stirred in anhydrous DMF (150 mL) overnight at 150 °C. Reaction was diluted with 200 mL of ether, washed 3 × 150 mL of water and dried with anhydrous $MgSO_4$. Crude was purified by column chromatography eluting with heptane/EtOAc (95:5) to obtain the title compound as a colourless oil (2.15 g, 53%).

^{1}H NMR (600 MHz, $CDCl_3$) δ 7.72 (dt, *J* = 7.8, 1.0 Hz, 1H), 7.67 – 7.64 (m, 2H), 7.55 (dt, *J* = 8.3, 0.9 Hz, 1H), 7.42 – 7.39 (m, 2H), 7.31 (d, *J* = 3.3 Hz, 1H), 7.26 (ddd, *J* = 8.3, 6.1, 1.3 Hz, 1H), 7.21 (ddd, *J* = 8.0, 7.1, 1.1 Hz, 1H), 6.72 (dd, *J* = 3.3, 0.9 Hz, 1H). ^{13}C NMR (151 MHz, $CDCl_3$) δ 139.0, 135.8, 132.9, 129.5, 127.7, 125.9, 122.8, 121.4, 120.8, 119.8, 110.4, 104.3.

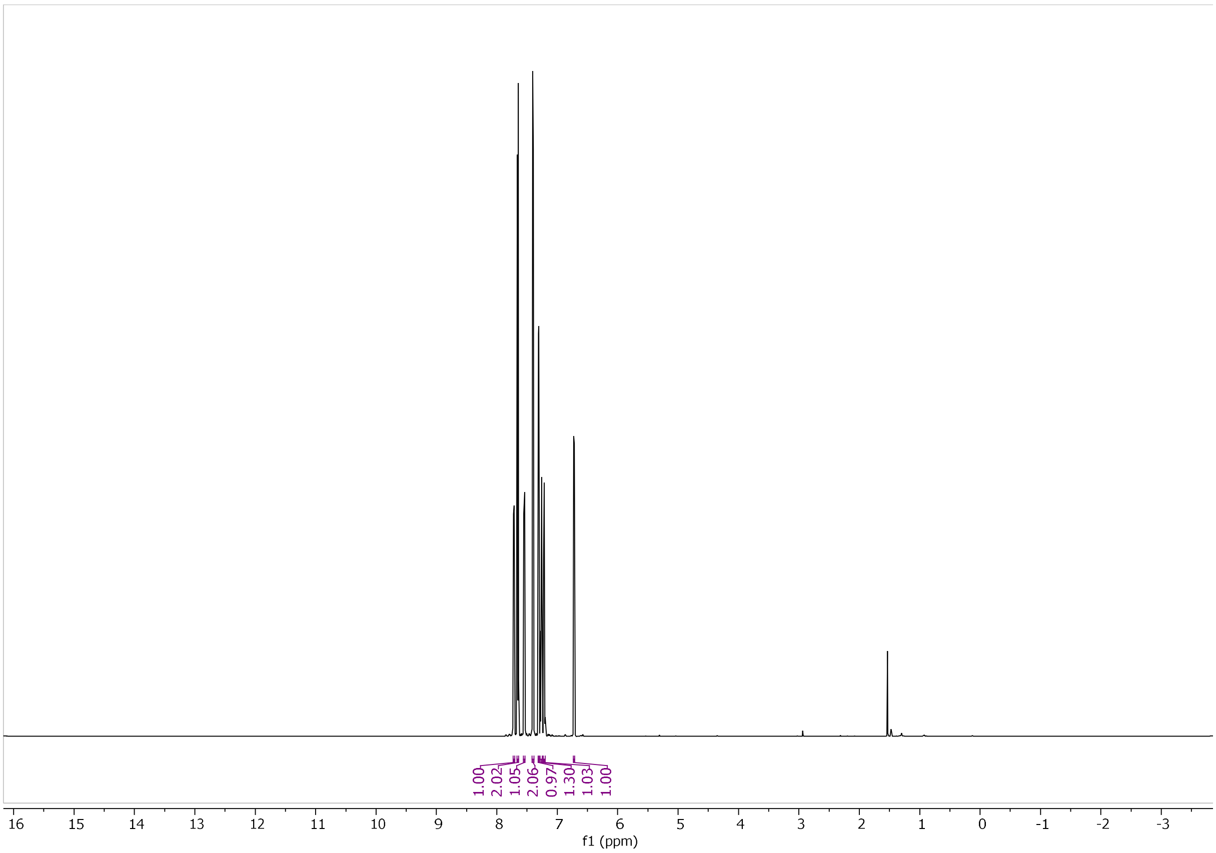

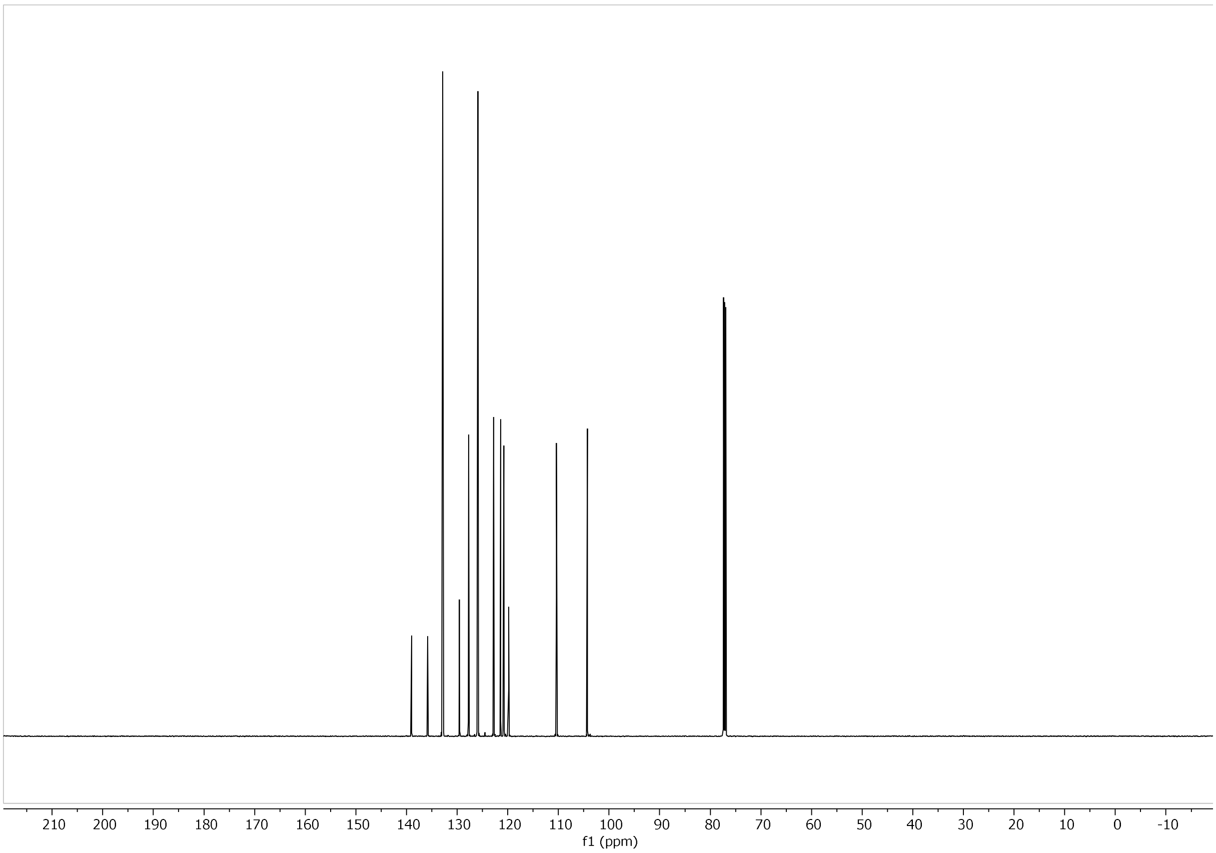
210 200 190 180 170 160 150 140 130 120 110 100 90 80 70 60 50 40 30 20 10 0 -10
f1 (ppm)

N-(4-(1H-benzo[d]imidazol-1-yl)phenyl)-4H-1,2,4-triazol-3-amine (1)

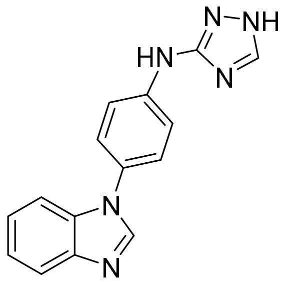

A flame-dried vial was charged with **2a** (137 mg, 0.5 mmol), 3-amino-1,2,4-triazole (50 mg, 0.6 mmol), tBuXPhos Pd G3 (20 mg, 0.025 mmol), tBuXPhos (11 mg, 0.025 mmol) and sodium t-butoxide (96 mg, 1 mmol). It was sealed and evacuated/backfilled with argon three times. A total of 3 mL of anhydrous t-BuOH was added and the reaction was stirred at 70 °C for 4 hours. Reaction was diluted with MeOH and filtered through a plug of silica. Crude was purified by preparative HPLC and processed through an SCX column to obtain the title compound as a white solid (32 mg, 23%). Compound was freeze-dried as HCl salt.

^{1}H NMR (600 MHz, D_2O) δ 9.49 (s, 1H), 8.55 (s, 1H), 7.94 (d, J = 8.2 Hz, 1H), 7.79 (d, J = 8.2 Hz, 1H), 7.77 – 7.66 (m, 6H). ^{13}C NMR (151 MHz, D_2O) δ 151.7, 140.4, 139.8, 139.8, 131.5, 130.4, 128.9, 127.4, 127.2, 126.5, 121.1, 114.8, 113.1. HRMS (MALDI): m/z calculated for $[M+H]^+$ 277.11962, found 277.11956.

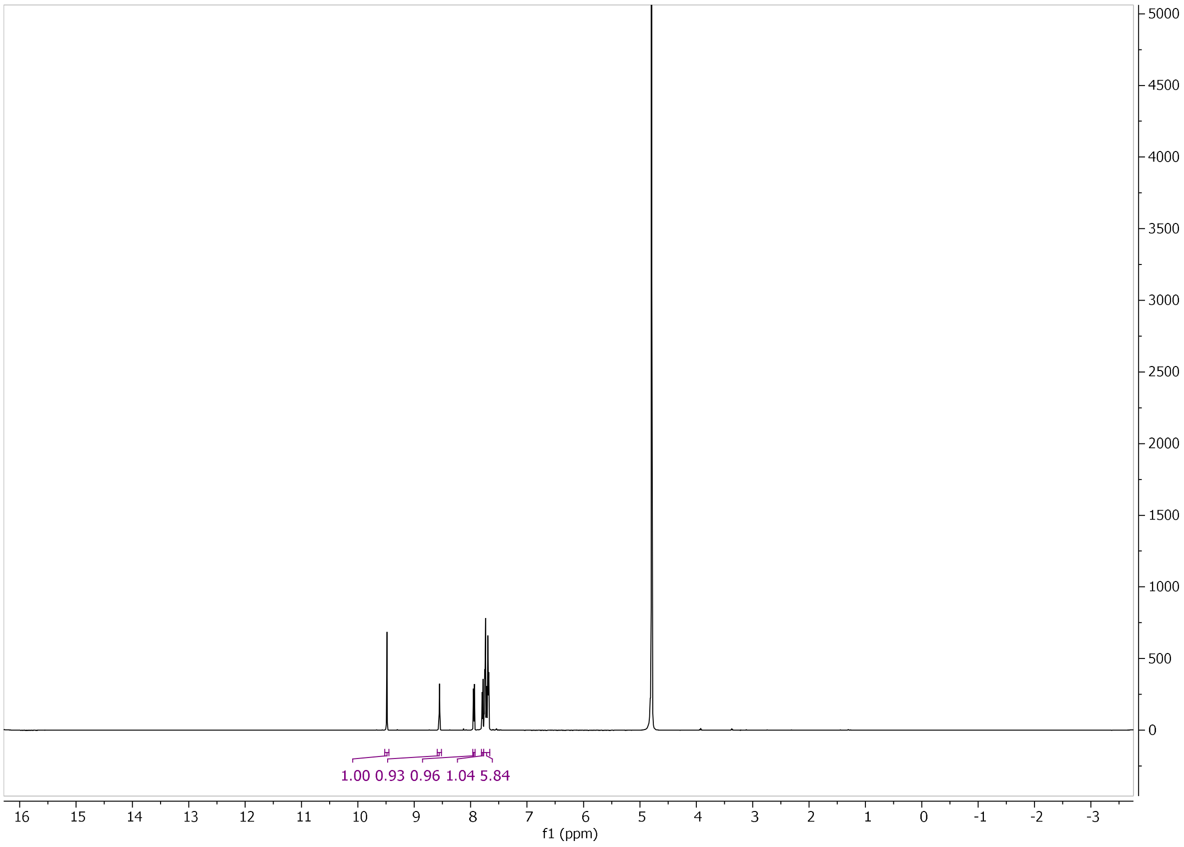

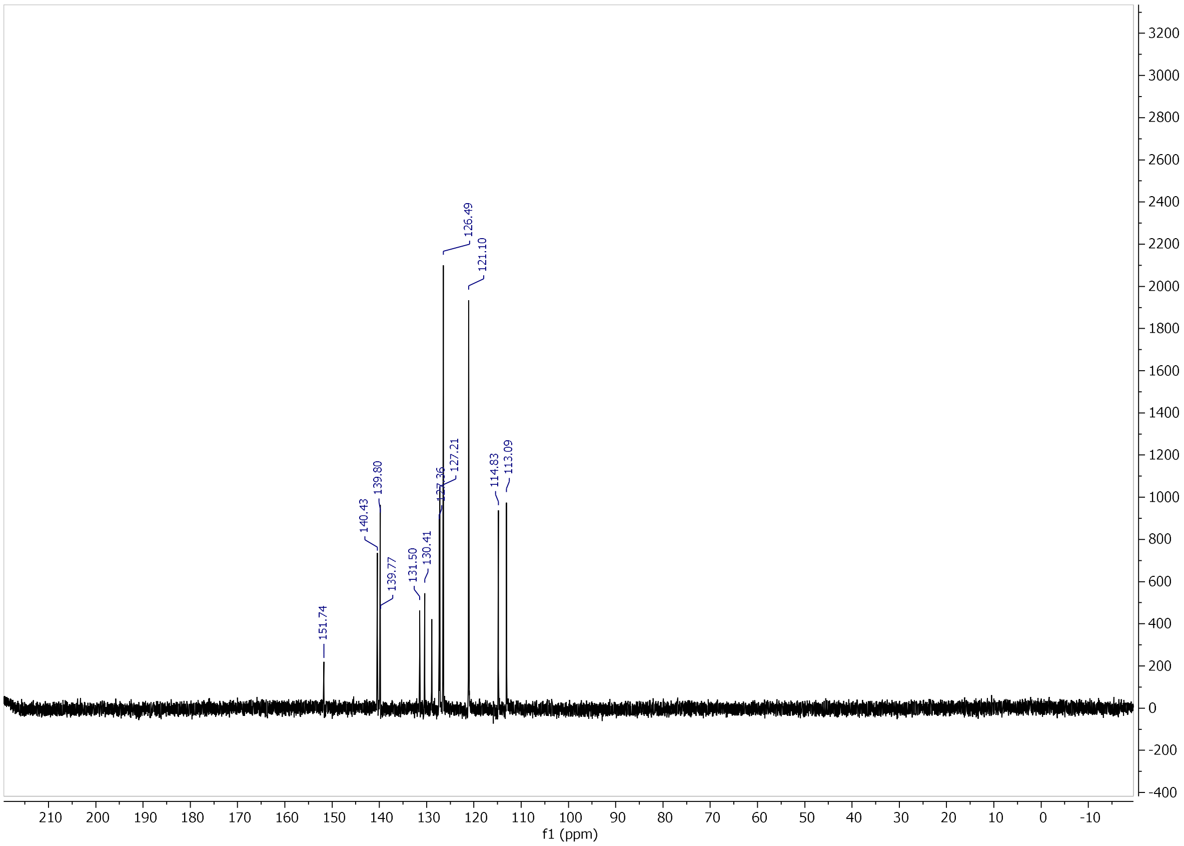

151.74
140.43
139.80
139.77
131.50
130.41
127.36
127.21
126.49
121.10
114.83
113.09
f1 (ppm)

4-(1*H*-benzo[*d*]imidazol-1-yl)-*N*-cyclohexylaniline (2)

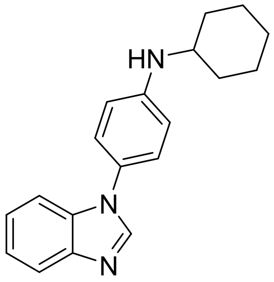

A flame-dried vial was charged with **2a** (273 mg, 1 mmol), tBuXPhos Pd G1 (7 mg, 0.01 mmol), tBuXPhos (4 mg, 0.01 mmol) and sodium t-butoxide (115 mg, 1.2 mmol). It was sealed and evacuated/backfilled with argon three times. Cyclohexylamine (0.14 mL, 1.2 mmol) was added followed by 2 mL of anhydrous THF. Reaction was stirred at 60 °C for 2 hours. It was then diluted with 10 mL of EtOAc and filtered through Celite. Crude was purified by column chromatography on silica eluting with 40% EtOAc in heptane to obtain the title compound as a white solid (54 mg, 19%).

^{1}H NMR (600 MHz, DMSO-*d*$_6$) δ 8.33 (s, 1H), 7.75 – 7.71 (m, 1H), 7.47 – 7.42 (m, 1H), 7.31 – 7.23 (m, 4H), 6.74 (d, *J* = 8.6 Hz, 2H), 5.81 (d, *J* = 8.0 Hz, 1H), 3.29 – 3.21 (m, 1H), 2.01 – 1.92 (m, 2H), 1.78 – 1.70 (m, 2H), 1.65 – 1.58 (m, 1H), 1.42 – 1.31 (m, 2H), 1.26 – 1.15 (m, 3H). ^{13}C NMR (151 MHz, DMSO-*d*$_6$) δ 147.7, 143.4, 143.4, 133.9, 125.1, 123.5, 122.8, 121.8, 119.6, 112.5, 110.4, 50.5, 32.4, 25.5, 24.4. HRMS (MALDI): *m*/*z* calculated for [M+H]$^+$ 292.18082, found 292.18078.

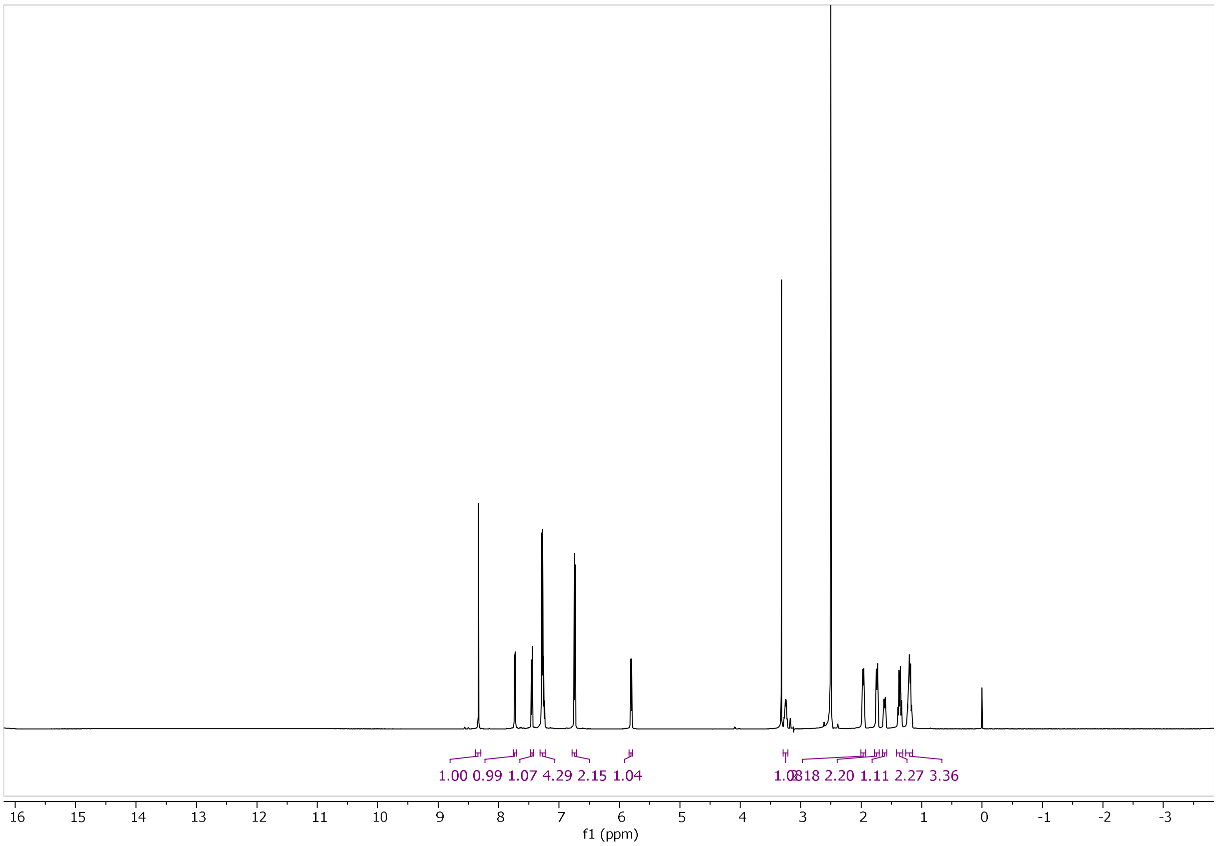

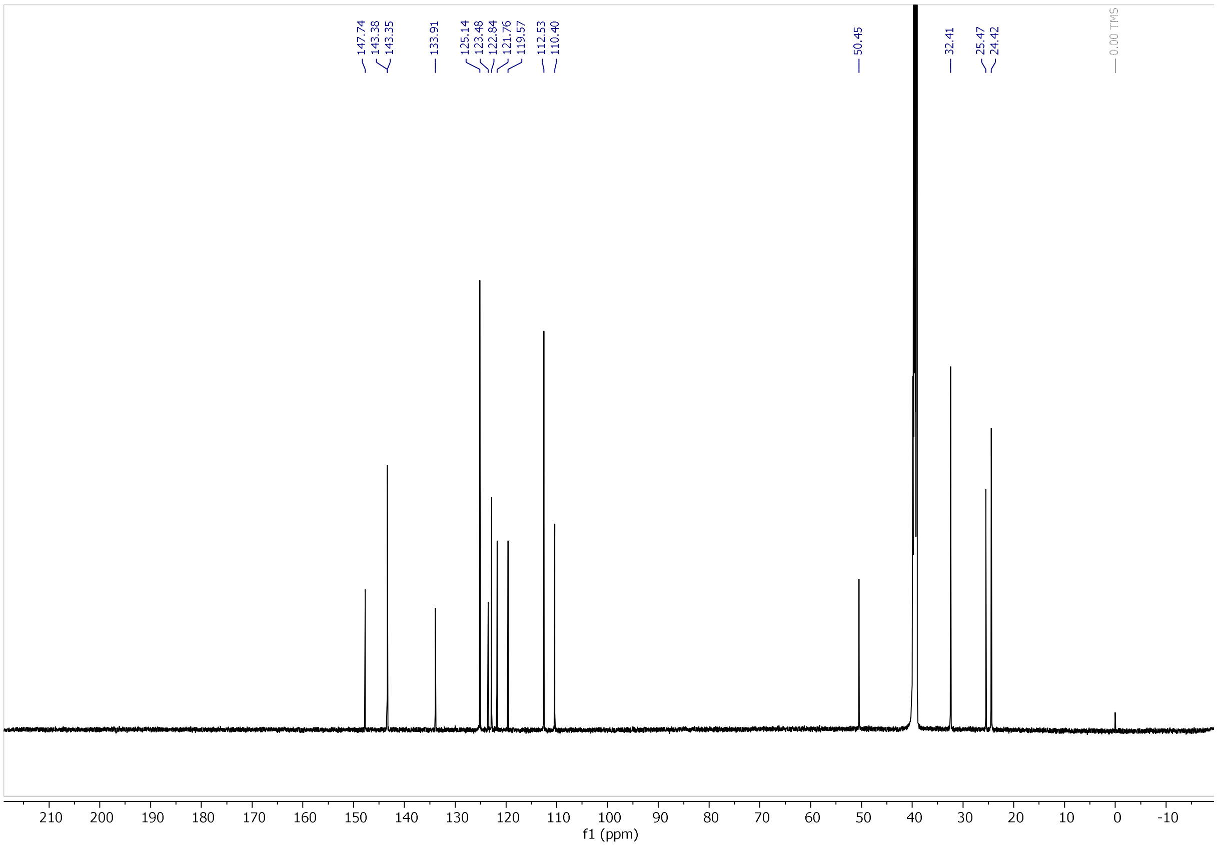
147.74
143.38
143.35
133.91
125.14
123.48
122.84
121.76
119.57
112.53
110.40
50.45
32.41
25.47
24.42
0.00 TMS
210 200 190 180 170 160 150 140 130 120 110 100 90 80 70 60 50 40 30 20 10 0 -10
f1 (ppm)

N-(4-(1H-benzo[d]imidazol-1-yl)phenyl)-1-methylpiperidin-4-amine (3)

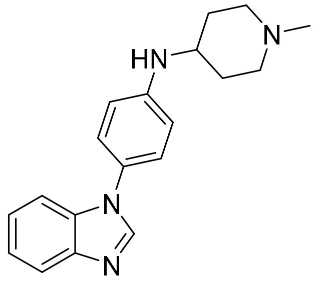

A flame-dried vial was charged with **2a** (273 mg, 1 mmol), tBuXPhos Pd G1 (7 mg, 0.01 mmol), tBuXPhos (4 mg, 0.01 mmol) and sodium t-butoxide (115 mg, 1.2 mmol). It was sealed and evacuated/backfilled with argon three times. 1-Methylpiperidin-4-amine (0.15 mL, 1.2 mmol) was added followed by 2 mL of anhydrous THF. Reaction was stirred at 60 °C for 2 hours. It was then diluted with 10 mL of EtOAc and filtered through Celite. Crude was purified by column chromatography on silica eluting with MeOH/EtOAc (0:100 → 7:93) + 2% TEA to obtain the title compound as a light brown solid (220 mg, 73%).

^{1}H NMR (600 MHz, DMSO-d_6) δ 8.34 (s, 1H), 7.76 – 7.71 (m, 1H), 7.47 – 7.43 (m, 1H), 7.31 – 7.24 (m, 4H), 6.78 – 6.74 (m, 2H), 5.85 (d, J = 7.9 Hz, 1H), 3.23 (tdt, J = 11.1, 8.4, 4.3 Hz, 1H), 2.74 (dt, J = 11.9, 3.5 Hz, 2H), 2.18 (s, 3H), 2.03 (td, J = 11.6, 2.6 Hz, 2H), 1.94 – 1.89 (m, 2H), 1.47 – 1.39 (m, 2H). ^{13}C NMR (151 MHz, DMSO-d_6) δ 148.2, 143.9, 143.9, 134.4, 125.7, 124.3, 123.4, 122.3, 120.1, 113.3, 111.0, 54.6, 49.1, 46.5, 32.1. HRMS (MALDI): m/z calculated for $[M+H]^+$ 307.19172, found 307.19169.

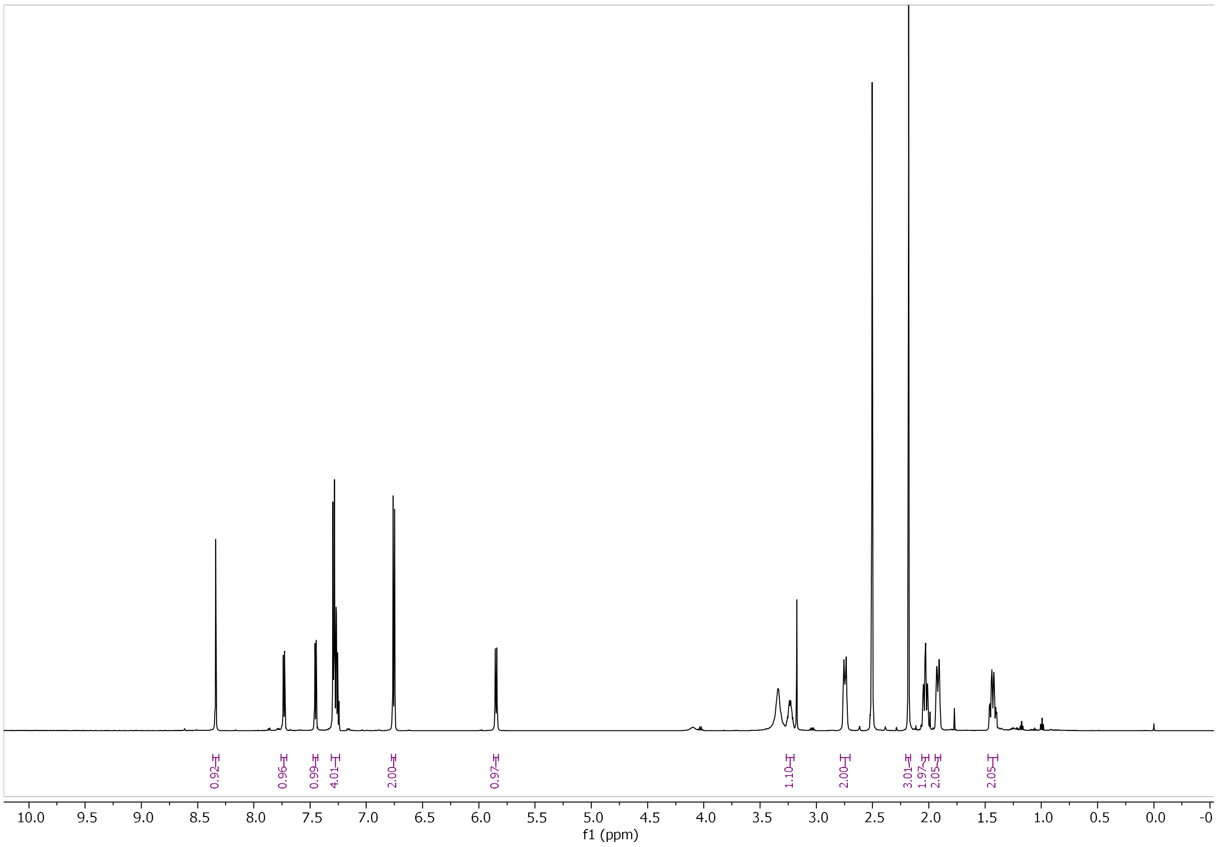

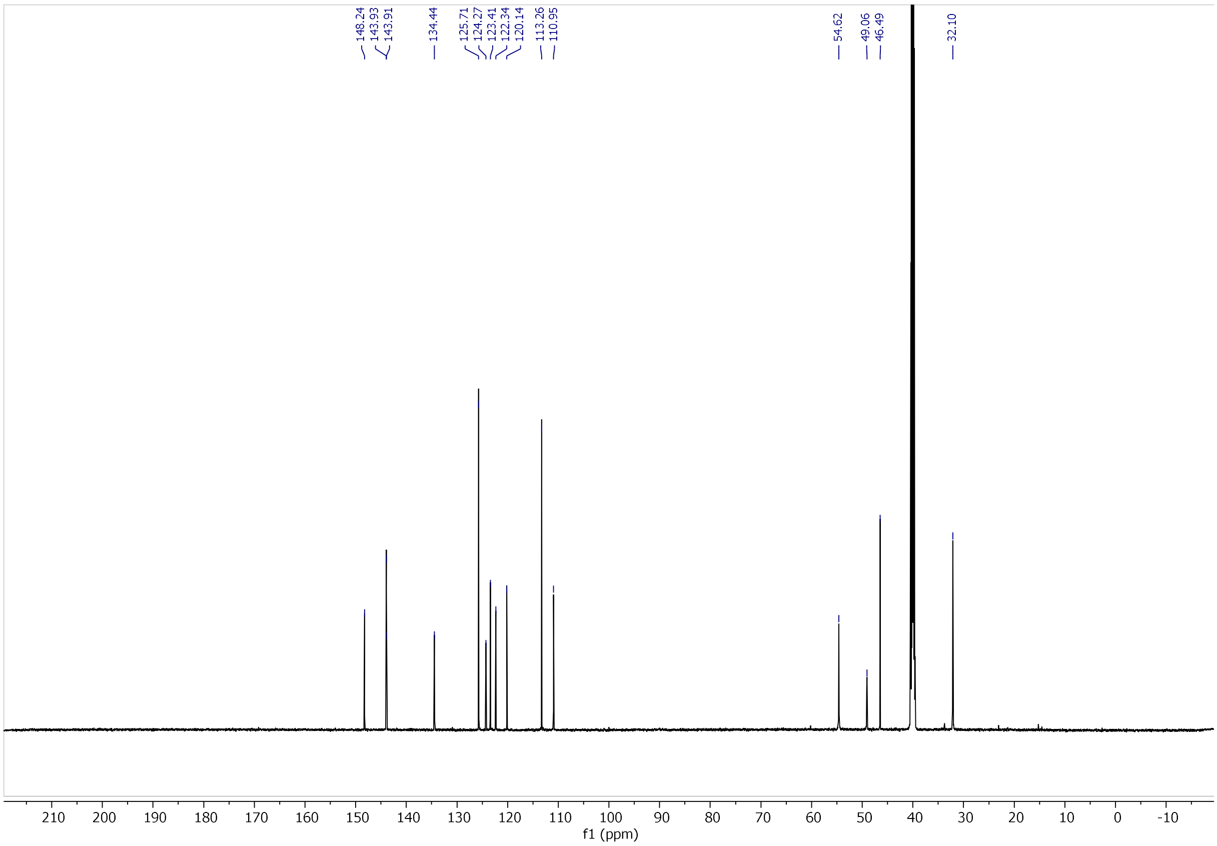
148.24
143.93
143.91
134.44
125.71
124.27
123.41
122.34
120.14
113.26
110.95
54.62
49.06
46.49
32.10
210 200 190 180 170 160 150 140 130 120 110 100 90 80 70 60 50 40 30 20 10 0 -10
f1 (ppm)

1-(4-(piperidin-1-yl)phenyl)-1*H*-benzo[*d*]imidazole (4)

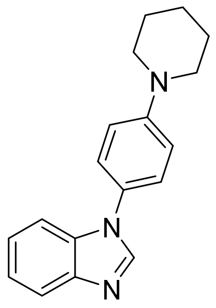

A flame-dried vial was charged with **2a** (273 mg, 1 mmol), tBuXPhos Pd G1 (7 mg, 0.01 mmol), tBuXPhos (4 mg, 0.01 mmol) and sodium t-butoxide (115 mg, 1.2 mmol). It was sealed and evacuated/backfilled with argon three times. Piperidine (0.12 mL, 1.2 mmol) was added followed by 2 mL of anhydrous THF. Reaction was stirred at 60 °C overnight and filtered. Solvent was removed and the residue passed through silica eluting 40% EtOAc in heptane + 1% 2M ammonia in MeOH. Crude was purified by preparative HPLC and processed through an SCX column to afford the title compound as an off-white solid (58 mg, 21%).

^{1}H NMR (600 MHz, DMSO-*d*$_6$) δ 8.42 (s, 1H), 7.78 – 7.73 (m, 1H), 7.53 – 7.48 (m, 1H), 7.44 (d, *J* = 8.8 Hz, 2H), 7.29 (pd, *J* = 7.1, 1.5 Hz, 2H), 7.12 (d, *J* = 8.9 Hz, 2H), 3.28 – 3.21 (m, 4H), 1.67 – 1.62 (m, 4H), 1.57 (q, *J* = 5.4, 5.0 Hz, 2H). ^{13}C NMR (151 MHz, DMSO-*d*$_6$) δ 150.8, 143.5, 143.3, 133.6, 126.3, 124.7, 123.0, 122.0, 119.7, 116.2, 110.4, 49.1, 25.0, 23.8. HRMS (MALDI): *m*/*z* calculated for [M+H]$^+$ 278.16517, found 278.16513.

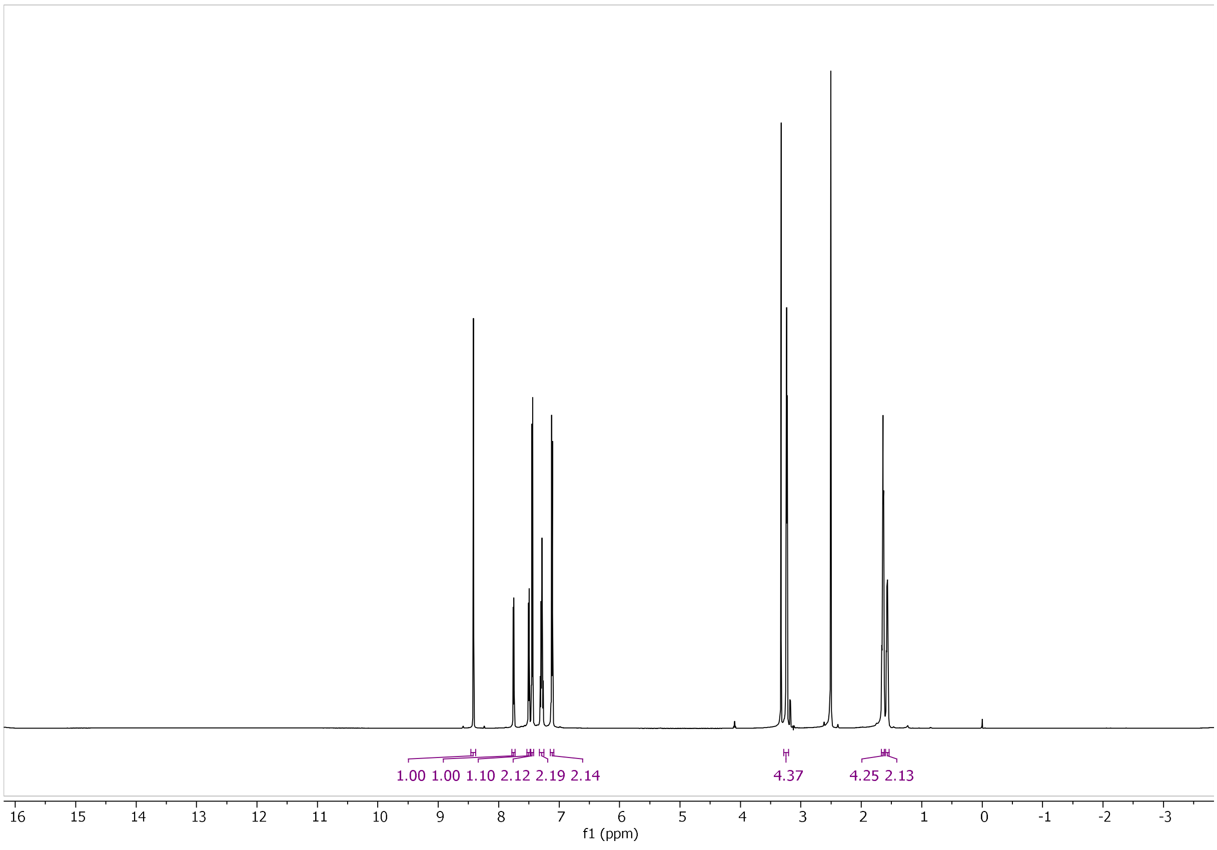

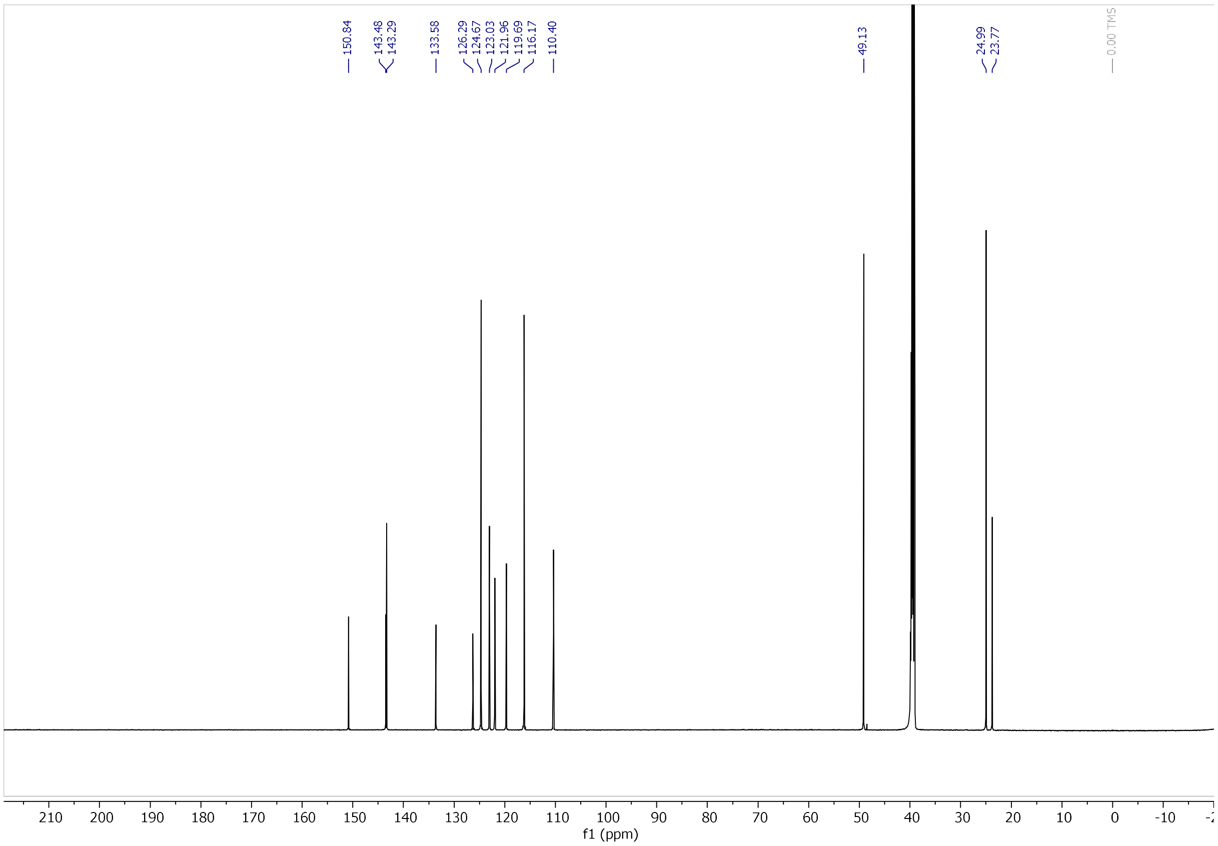

150.84
143.48
143.29
133.58
126.29
124.67
123.03
121.96
119.69
116.17
110.40
49.13
24.99
23.77
0.00 TMS
210 200 190 180 170 160 150 140 130 120 110 100 90 80 70 60 50 40 30 20 10 0 -10
f1 (ppm)

4-(4-(1*H*-benzo[*d*]imidazol-1-yl)phenyl)morpholine (5)

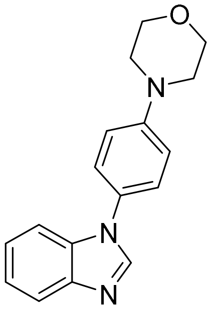

A flame-dried vial was charged with **2a** (273 mg, 1 mmol), tBuXPhos Pd G1 (7 mg, 0.01 mmol), tBuXPhos (4 mg, 0.01 mmol) and sodium t-butoxide (115 mg, 1.2 mmol). It was sealed and evacuated/backfilled with argon three times. Morpholine (0.11 mL, 1.2 mmol) was added followed by 2 mL of anhydrous THF. Reaction was stirred at 60 °C for 2 hours. It was diluted with 10 mL of EtOAc and filtered. Crude was purified by column chromatography on silica eluting with EtOAc/heptane (40:60 → 90:10) to obtain the title compound as a white solid (184 mg, 66%).

^{1}H NMR (600 MHz, DMSO-*d*$_6$) δ 8.44 (s, 1H), 7.85 – 7.75 (m, 1H), 7.57 – 7.45 (m, 3H), 7.37 – 7.25 (m, 2H), 7.14 (d, *J* = 8.9 Hz, 2H), 3.83 – 3.75 (m, 4H), 3.19 (dd, *J* = 5.9, 3.7 Hz, 4H). ^{13}C NMR (151 MHz, DMSO-*d*$_6$) δ 150.5, 143.5, 143.3, 133.6, 127.2, 124.7, 123.1, 122.1, 119.8, 115.7, 110.4, 66.0, 48.1. HRMS (MALDI): *m*/*z* calculated for [M+H]$^+$ 280.14444, found 280.14443.

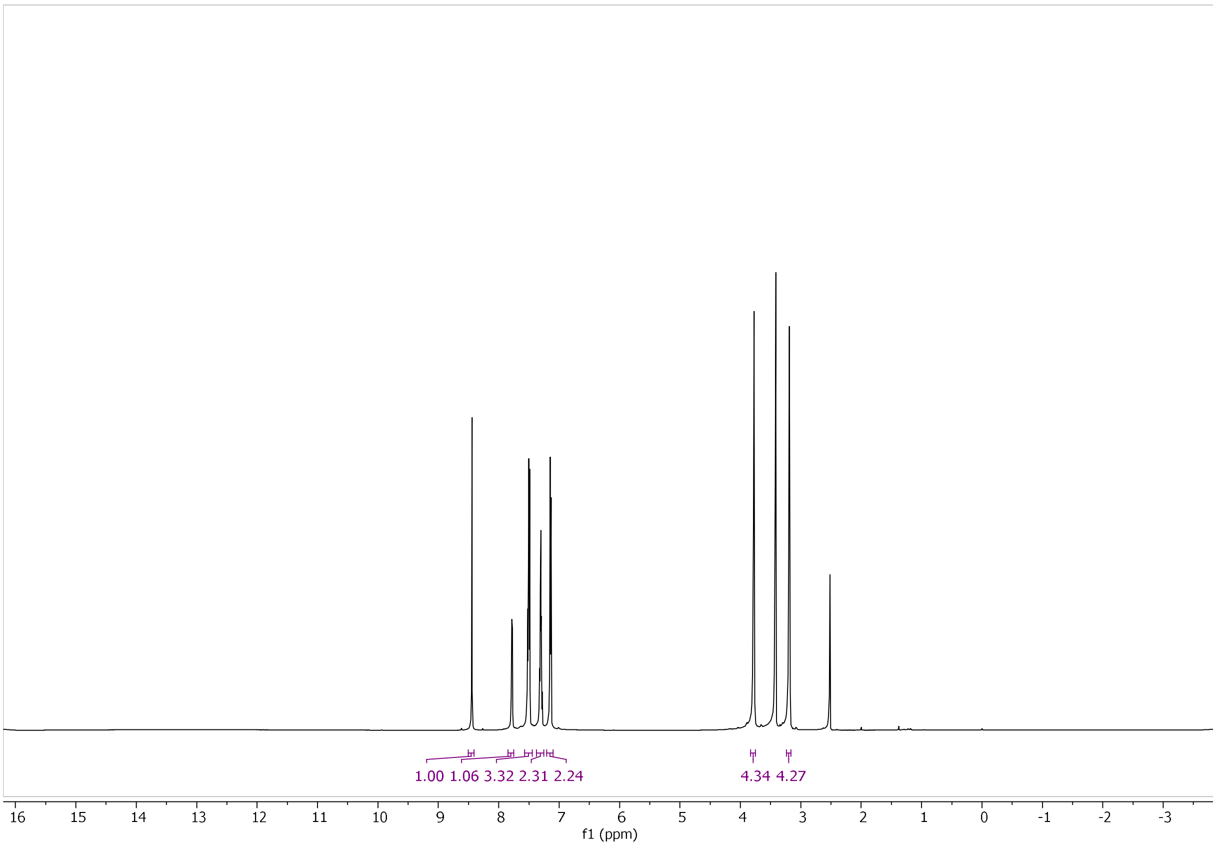

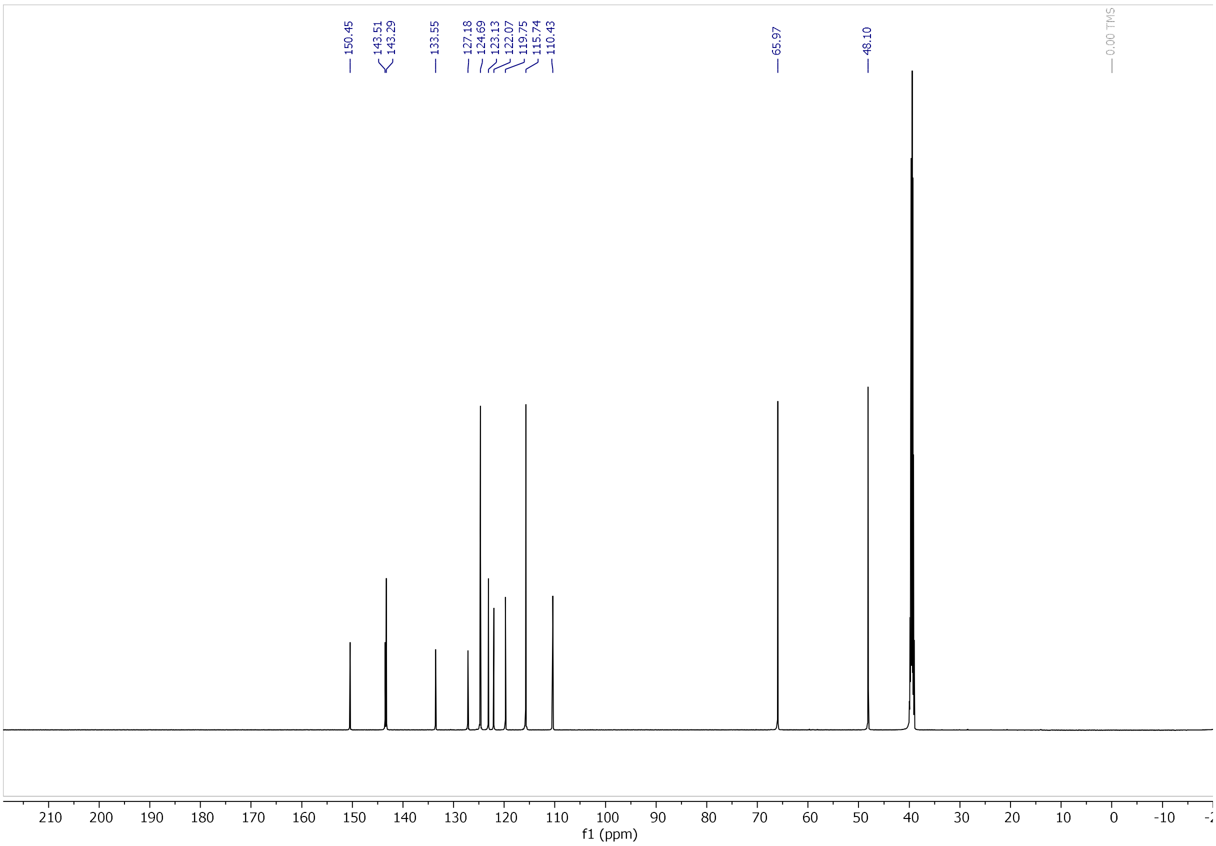
150.45
143.51
143.29
133.55
127.18
124.69
123.13
122.07
119.75
115.74
110.43
65.97
48.10
0.00 TMS
210 200 190 180 170 160 150 140 130 120 110 100 90 80 70 60 50 40 30 20 10 0 -10
f1 (ppm)

1-(4-(4-methylpiperazin-1-yl)phenyl)-1*H*-benzo[*d*]imidazole (6)

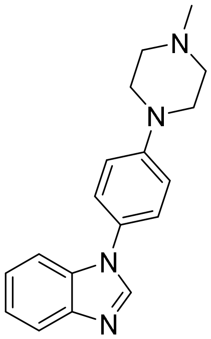

A flame-dried vial was charged with **2a** (273 mg, 1 mmol), tBuXPhos Pd G1 (7 mg, 0.01 mmol), tBuXPhos (4 mg, 0.01 mmol) and sodium t-butoxide (115 mg, 1.2 mmol). It was sealed and evacuated/backfilled with argon three times. Methylpiperazine (0.13 mL, 1.2 mmol) was added followed by 2 mL of anhydrous THF. Reaction was stirred at 60 °C for 2 hours. It was then diluted with 10 mL of EtOAc and filtered through Celite. Crude was purified by column chromatography on silica eluting with MeOH/EtOAc (0:100 → 5:95) + 2% TEA to obtain the title compound as a white solid (160 mg, 55%).

^{1}H NMR (600 MHz, DMSO-d_6) δ 8.42 (s, 1H), 7.75 (dd, *J* = 7.5, 1.8 Hz, 1H), 7.52 – 7.49 (m, 1H), 7.49 – 7.45 (m, 2H), 7.32 – 7.26 (m, 2H), 7.16 – 7.13 (m, 2H), 3.26 – 3.21 (m, 4H), 2.49 – 2.47 (m, 4H), 2.24 (s, 3H). ^{13}C NMR (151 MHz, DMSO-d_6) δ 150.4, 143.5, 143.3, 133.5, 126.7, 124.7, 123.1, 122.0, 119.7, 115.9, 110.4, 54.4, 47.7, 45.7. HRMS (MALDI): *m*/*z* calculated for $[M+H]^+$ 293.17607, found 293.17605.

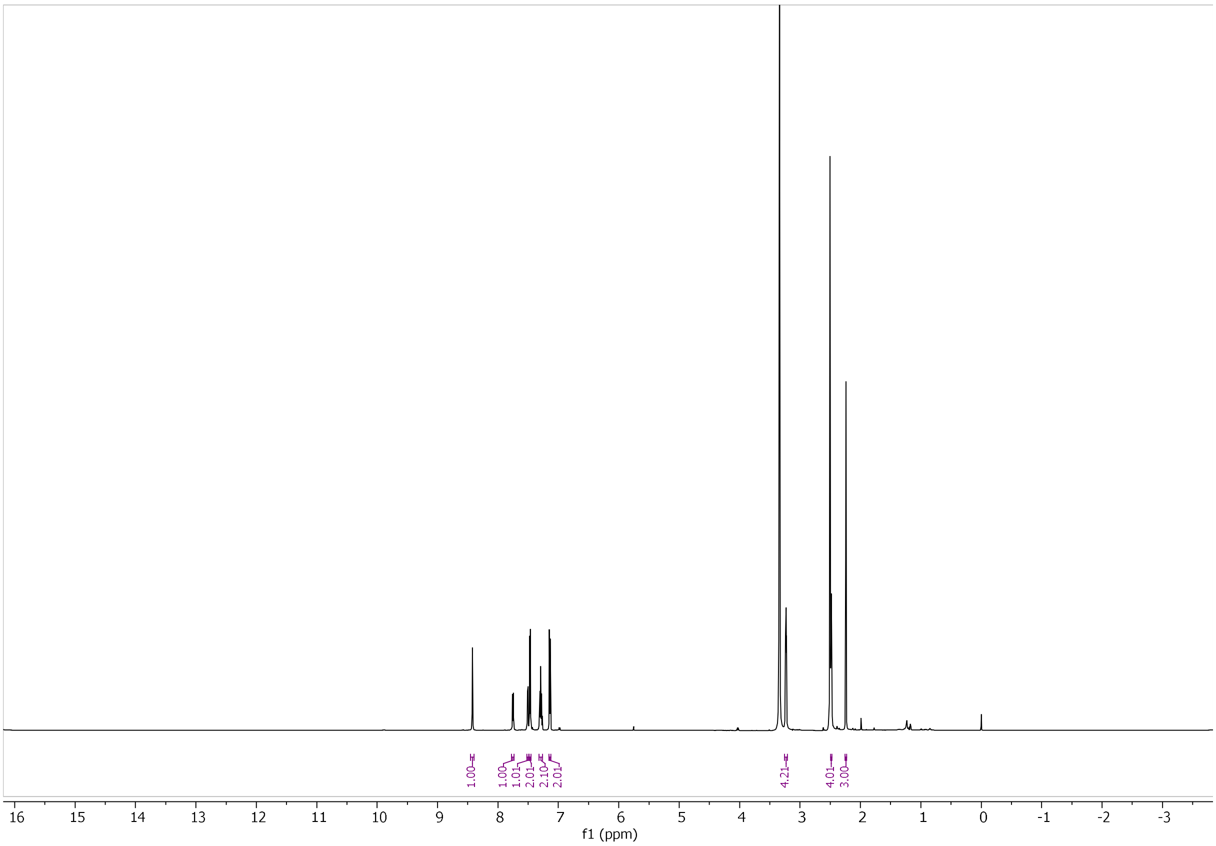

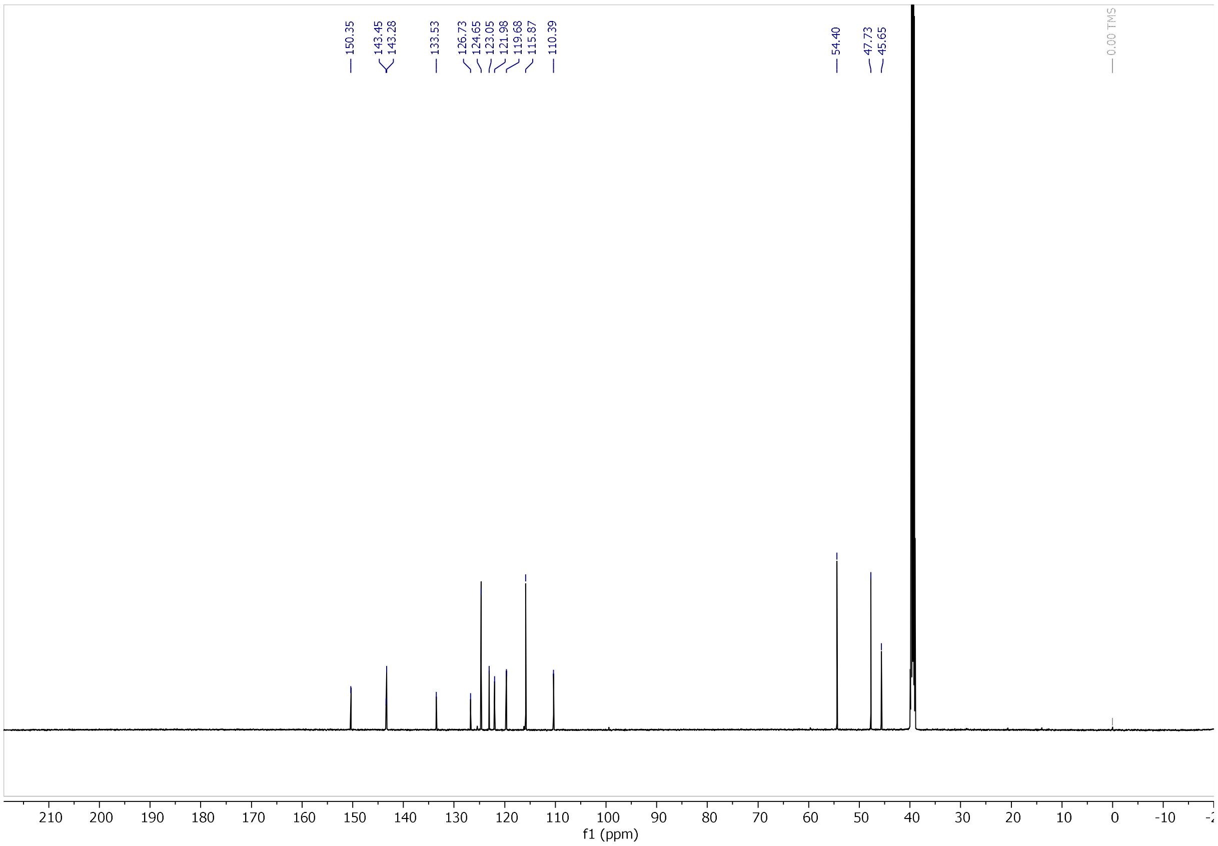

150.35
143.45
143.28
133.53
126.73
124.65
123.05
121.98
119.68
115.87
110.39
54.40
47.73
45.65
0.00 TMS
210 200 190 180 170 160 150 140 130 120 110 100 90 80 70 60 50 40 30 20 10 0 -10
f1 (ppm)

N^1-(4-(1H-benzo[d]imidazol-1-yl)phenyl)-N^2,N^2-dimethylethane-1,2-diamine (7)

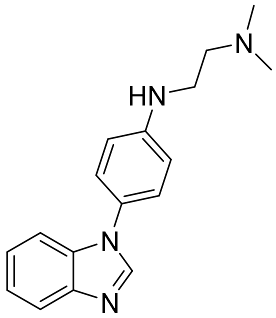

A flame-dried vial was charged with **2a** (273 mg, 1 mmol), tBuXPhos Pd G1 (7 mg, 0.01 mmol), tBuXPhos (4 mg, 0.01 mmol) and sodium t-butoxide (115 mg, 1.2 mmol). It was sealed and evacuated/backfilled with argon three times. Dimethylethylenediamine (0.13 mL, 1.2 mmol, freshly distilled) was added followed by 2 mL of anhydrous THF. Reaction was stirred at 60 °C for 2 hours. It was then diluted with 10 mL of EtOAc, filtered through Celite and stripped of solvent. Residue was taken up in ether, filtered through a cotton-wadded pipette and treated with 2 mL of 1M HCl in ether. Produced precipitate was recrystallized from EtOH to afford the title compound as an off-white solid in a form of a hydrochloride salt (98 mg, 28% calc. for 2×HCl).

^{1}H NMR (400 MHz, D_2O) δ 9.27 (s, 1H), 7.86 (dt, J = 8.2, 0.9 Hz, 1H), 7.70 – 7.57 (m, 3H), 7.47 (d, J = 8.8 Hz, 2H), 6.94 (d, J = 8.9 Hz, 2H), 3.62 (t, J = 6.1 Hz, 2H), 3.40 (t, J = 6.1 Hz, 2H), 2.92 (s, 6H). ^{13}C NMR (101 MHz, D_2O) δ 149.0, 139.7, 131.9, 130.7, 127.1, 126.9, 126.4, 123.5, 114.8, 113.9, 113.1, 56.0, 43.0, 38.1. HRMS (MALDI): m/z calculated for $[M+H]^+$ 281.17607, found 281.17606.

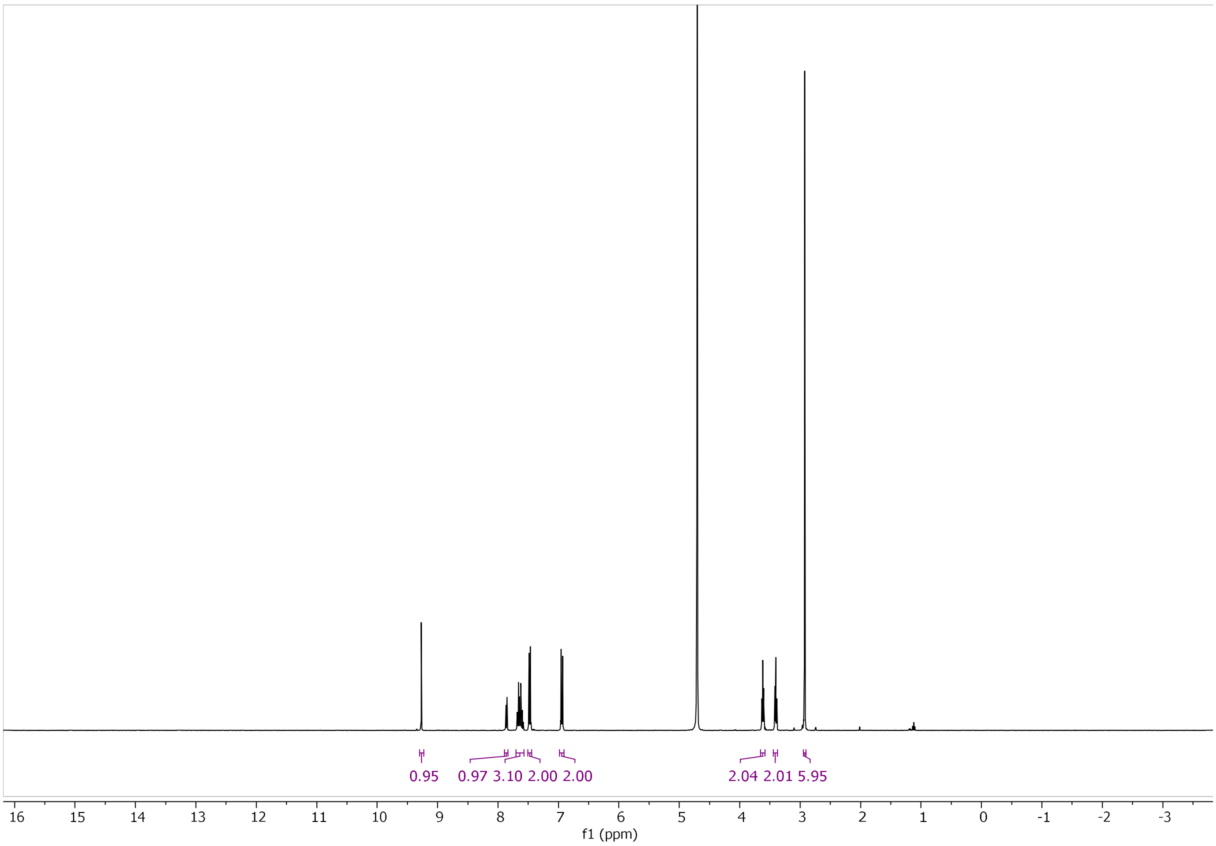

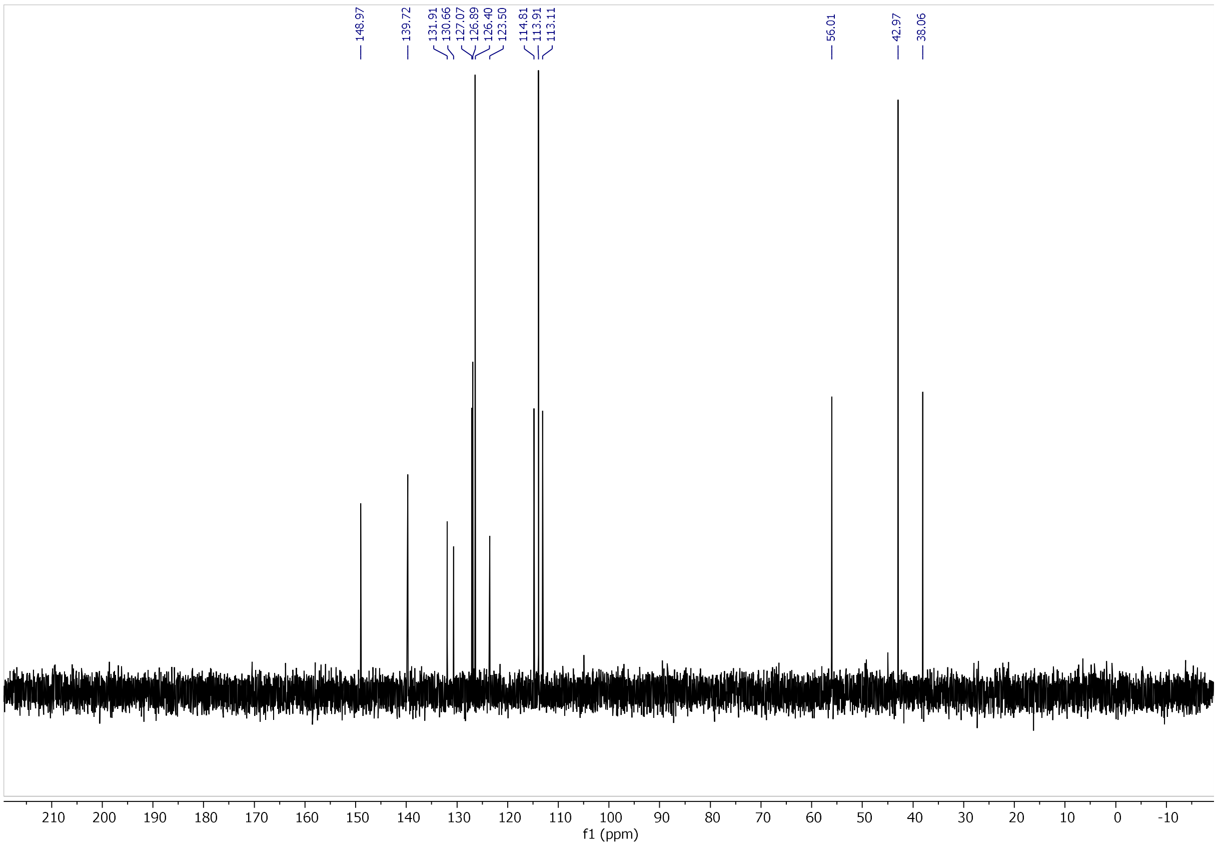
148.97
139.72
131.91
130.66
127.07
126.89
126.40
123.50
114.81
113.91
113.11
56.01
42.97
38.06
210
200
190
180
170
160
150
140
130
120
110
100
90
80
70
60
50
40
30
20
10
0
-10
f1 (ppm)

N-(4-(1H-indol-1-yl)phenyl)-1H-1,2,4-triazol-3-amine (8)

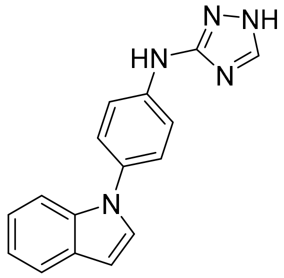

A flame-dried vial was charged with 3-amino-1,2,4-triazole (101 mg, 1.2 mmol), **2b** (272 mg, 1 mmol), tBuXPhos Pd G3 (40 mg, 0.05 mmol), tBuXPhos (21 mg, 0.05 mmol) and sodium t-butoxide (192 mg, 2 mmol). It was sealed and evacuated/backfilled with argon three times. A total of 10 mL of anhydrous tBuOH was added and the reaction was stirred at 70 °C overnight. Solvent was removed and the residue was partitioned between 10 mL of water and 20 mL of EtOAc. Aqueous was extracted with 2 × 20 mL of EtOAc. Combined organics were dried with anhydrous Na_2SO_4 and stripped of solvent. Remaining residue was purified by column chromatography on silica eluting neat EtOAc + 1% TEA to afford the title compound as a light brown solid (58 mg, 21%).

^{1}H NMR (600 MHz, DMSO-d_6) δ 13.32 (s, 1H), 9.36 (s, 1H), 8.31 (s, 1H), 7.77 – 7.72 (m, 2H), 7.63 (dd, J = 7.7, 1.1 Hz, 1H), 7.54 (t, J = 3.2 Hz, 1H), 7.47 – 7.44 (m, 1H), 7.44 – 7.38 (m, 2H), 7.16 (ddd, J = 8.2, 6.9, 1.2 Hz, 1H), 7.09 (td, J = 7.4, 6.9, 1.0 Hz, 1H), 6.64 (t, J = 3.0 Hz, 1H). ^{13}C NMR (151 MHz, DMSO-d_6) δ 160.2, 142.1, 140.9, 135.4, 130.4, 128.6, 128.5, 124.7, 121.8, 120.6, 119.7, 116.3, 110.2, 102.4. HRMS (MALDI): m/z calculated for $[M+H]^+$ 276.12437, found 276.12463.

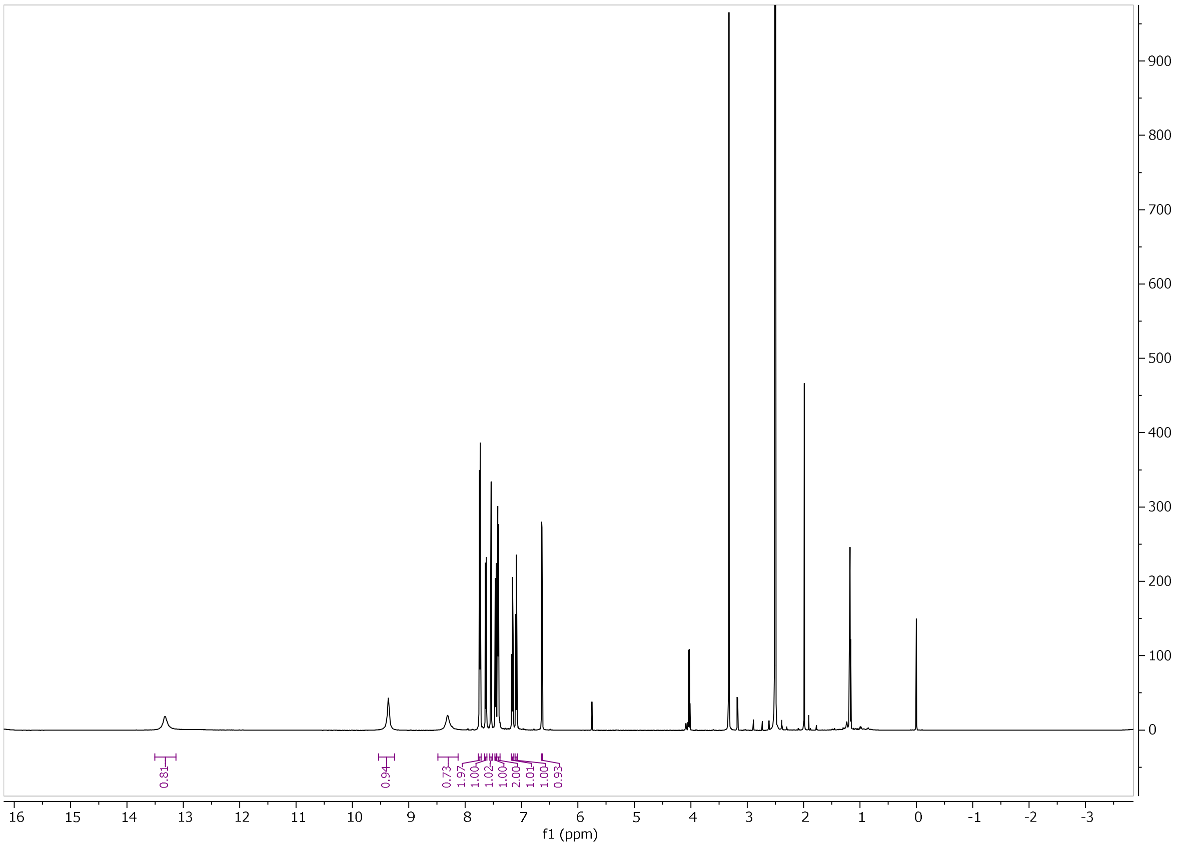

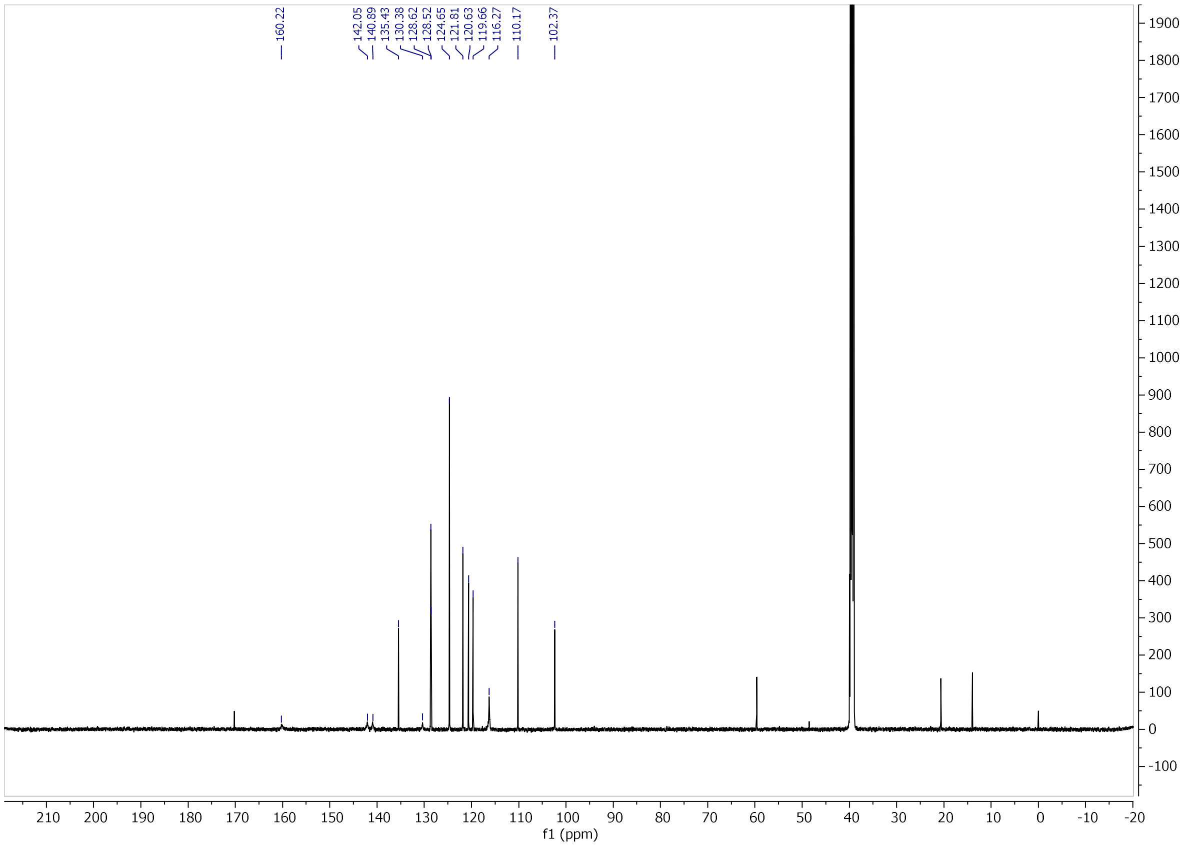

160.22
142.05
140.89
135.43
130.38
128.62
128.52
124.65
121.81
120.63
119.66
116.27
110.17
102.37
1900
1800
1700
1600
1500
1400
1300
1200
1100
1000
900
800
700
600
500
400
300
200
100
0
-100
210 200 190 180 170 160 150 140 130 120 110 100 90 80 70 60 50 40 30 20 10 0 -10 -20
f1 (ppm)

Scheme S2 Synthetic Details

1-(4-nitrophenyl)-1*H*-benzo[d]imidazole (4a)

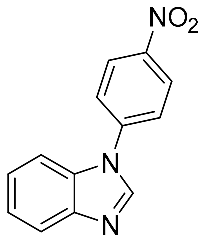

Benzimidazole (5.91 g, 50 mmol), 1-fluoro-4-nitrobenzene (7.76 g, 55 mmol) and potassium phosphate (21.23 g, 100 mmol) were stirred in anhydrous DMF (200 mL) overnight at 150 °C. Reaction was diluted with 500 mL of water and extracted 3×200 mL of ether. Combined organics were washed with brine and dried with anhydrous $MgSO_4$. Crude was purified by dissolving in hot EtOH, concentrating on a rotavap and filtering the formed solid. Thus, the title compound was obtained as a light brown solid (7.94 g, 66%).

^{1}H NMR (400 MHz, DMSO-d_6) δ 8.73 (s, 1H), 8.49 – 8.39 (m, 2H), 8.07 – 7.98 (m, 2H), 7.85 – 7.72 (m, 2H), 7.37 (pd, *J* = 7.2, 1.4 Hz, 2H). ^{13}C NMR (101 MHz, DMSO-d_6) δ 145.7, 144.1, 143.2, 141.3, 132.3, 125.5, 124.0, 123.7, 123.2, 120.2, 111.0.

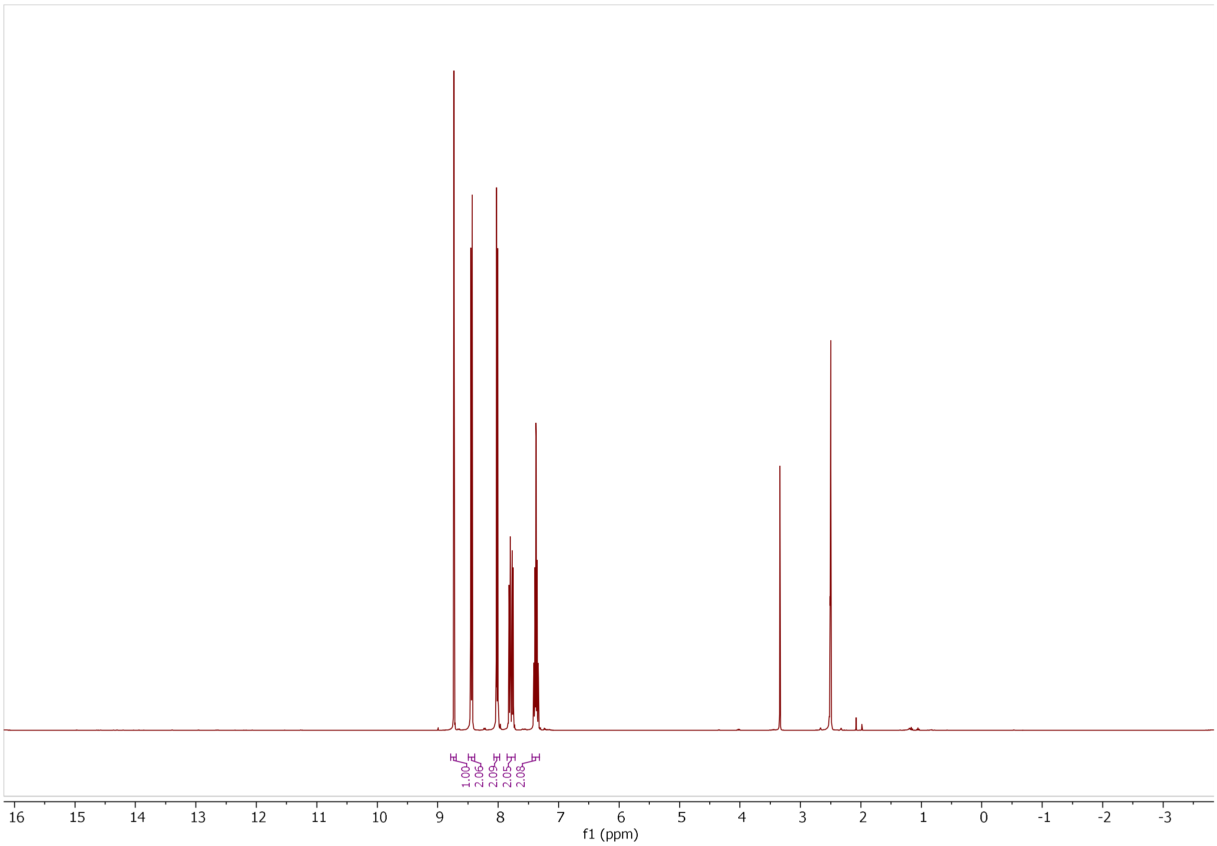

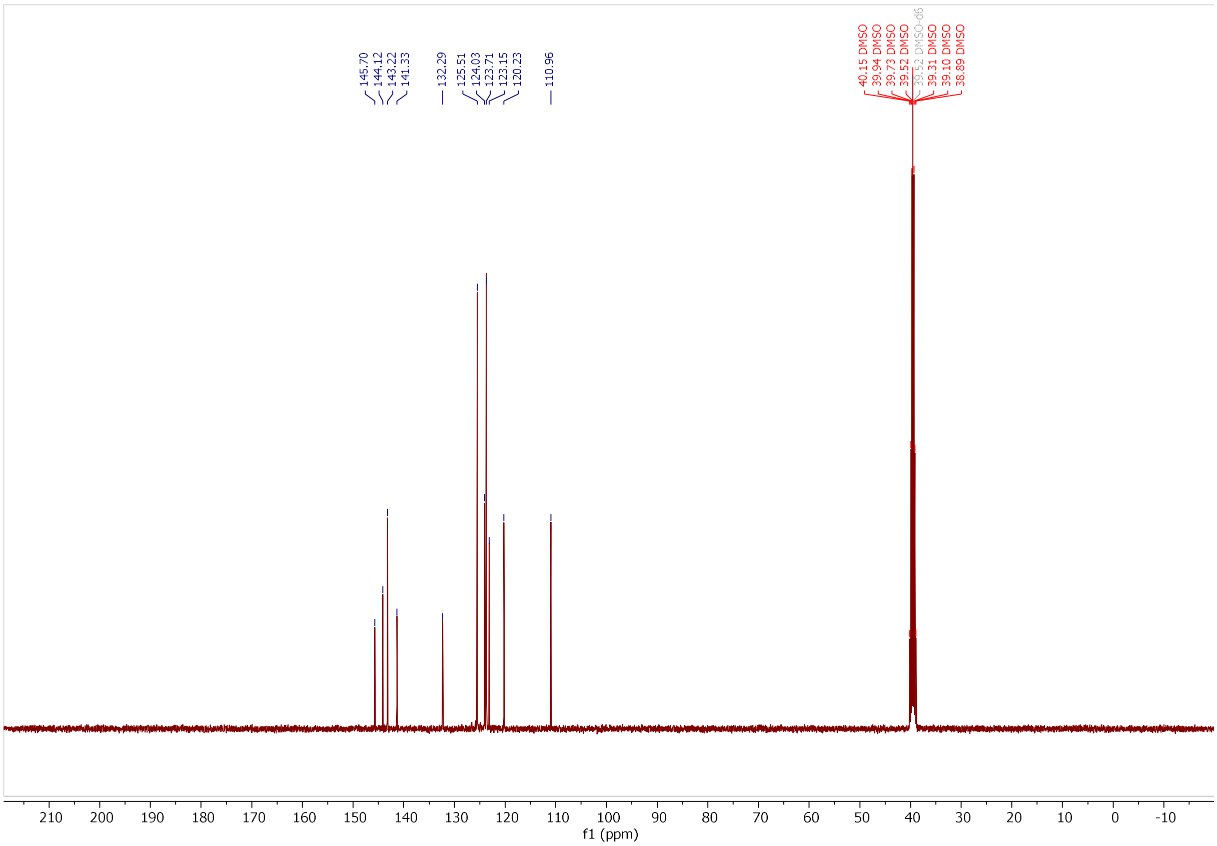

145.70
144.12
143.22
141.33
132.29
125.51
124.03
123.71
123.15
120.23
110.96
40.15 DMSO
39.94 DMSO
39.73 DMSO
39.52 DMSO
39.52 DMSO-d6
39.31 DMSO
39.10 DMSO
38.89 DMSO
210 200 190 180 170 160 150 140 130 120 110 100 90 80 70 60 50 40 30 20 10 0 -10
f1 (ppm)

1-(5-nitropyridin-2-yl)-1*H*-benzo[d]imidazole (4b)

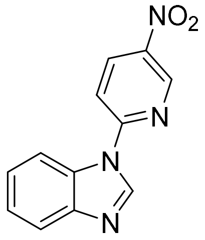

Benzimidazole (1.18 g, 10 mmol), 2-fluoro-5-nitropyridine (1.56 g, 11 mmol) and potassium phosphate (4.25 g, 20 mmol) were stirred in DMSO (40 mL) overnight at room temperature. Reaction was diluted with 200 mL of water and extracted 3×150 mL of EtOAc. Combined organics were washed with brine and dried with anhydrous $MgSO_4$. Obtained solid was washed subsequently with EtOAc and MeOH. Thus, the title compound was obtained as a yellow-grey solid (1.78 g, 74%).

^{1}H NMR (400 MHz, DMSO-d_6) δ 9.42 (dd, *J* = 2.9, 0.7 Hz, 1H), 9.16 (s, 1H), 8.81 (dd, *J* = 9.1, 2.8 Hz, 1H), 8.51 – 8.43 (m, 1H), 8.24 (dd, *J* = 9.2, 0.7 Hz, 1H), 7.84 – 7.76 (m, 1H), 7.49 – 7.36 (m, 2H). ^{13}C NMR (101 MHz, DMSO-d_6) δ 153.3, 145.0, 144.3, 142.5, 141.6, 134.8, 131.5, 124.6, 123.9, 120.0, 114.9, 113.8.

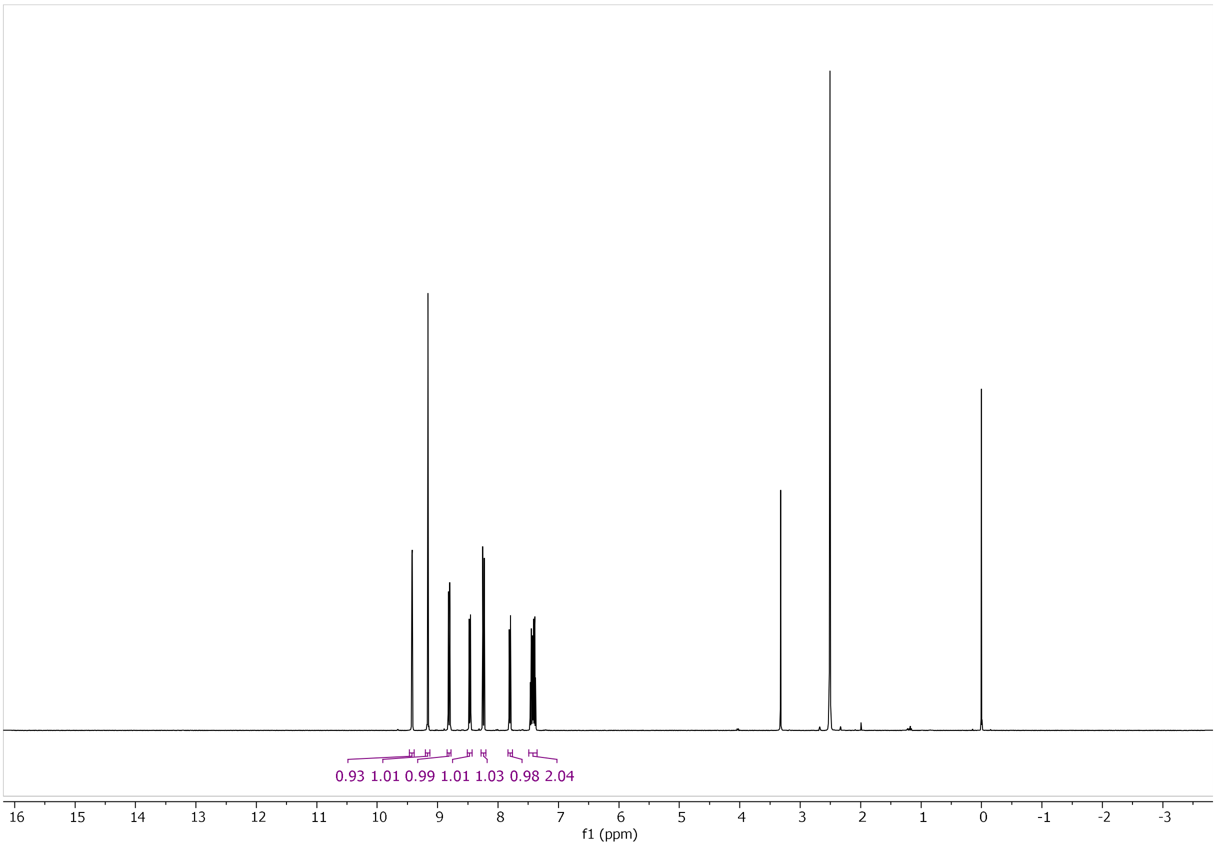

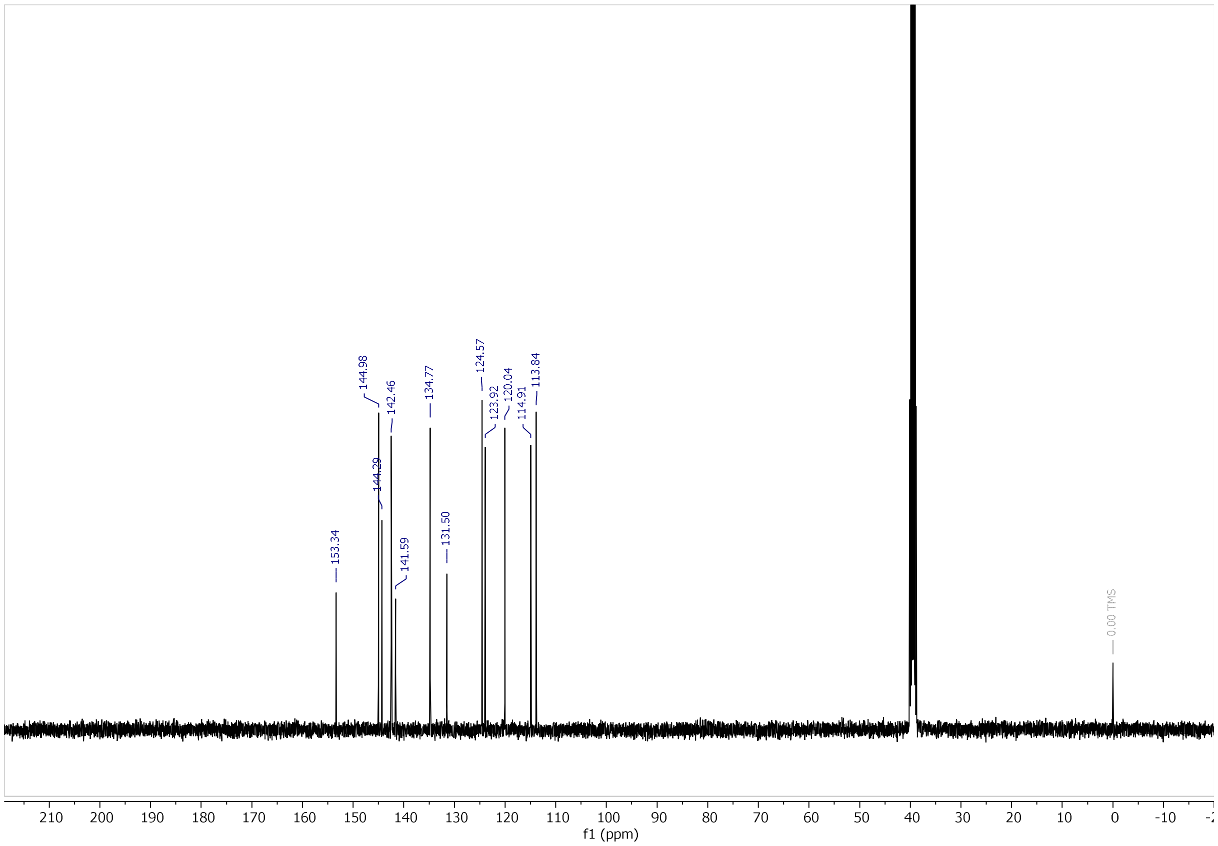

153.34
144.98
144.29
142.46
141.59
134.77
131.50
124.57
123.92
120.04
114.91
113.84
0.00 TMS
210
200
190
180
170
160
150
140
130
120
110
100
90
80
70
60
50
40
30
20
10
0
-10
f1 (ppm)

4-(1*H*-benzo[d]imidazol-1-yl)aniline (5a)

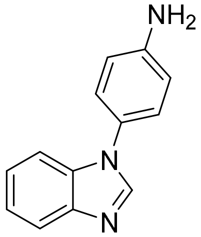

A total of 4.68 g (19.6 mmol) of **4a** was dissolved in 100 mL of MeOH and hydrogenated at room temperature and pressure overnight using 208 mg (0.2 mmol, 1mol%) of 10% Pd/C. Reaction was filtered from the catalyst and solvent removed on a rotavap. Crude was purified by column chromatography on silica eluting with 2M NH_3 MeOH/EtOAc (5:95) to obtain the title compound as an off-white oil (3.37 g, 82%). This compound can be converted into HCl salt and recrystallized from water/EtOH to produce spectacular long, fine needles.

^{1}H NMR (600 MHz, D_2O) δ 9.45 (s, 1H), 7.82 (dt, *J* = 8.3, 0.8 Hz, 1H), 7.80 – 7.76 (m, 2H), 7.65 (dd, *J* = 8.4, 1.7 Hz, 3H), 7.62 – 7.54 (m, 2H). ^{13}C NMR (151 MHz, D_2O) δ 140.0, 133.2, 132.9, 131.1, 130.4, 127.4, 127.4, 126.8, 124.9, 114.9, 112.9.

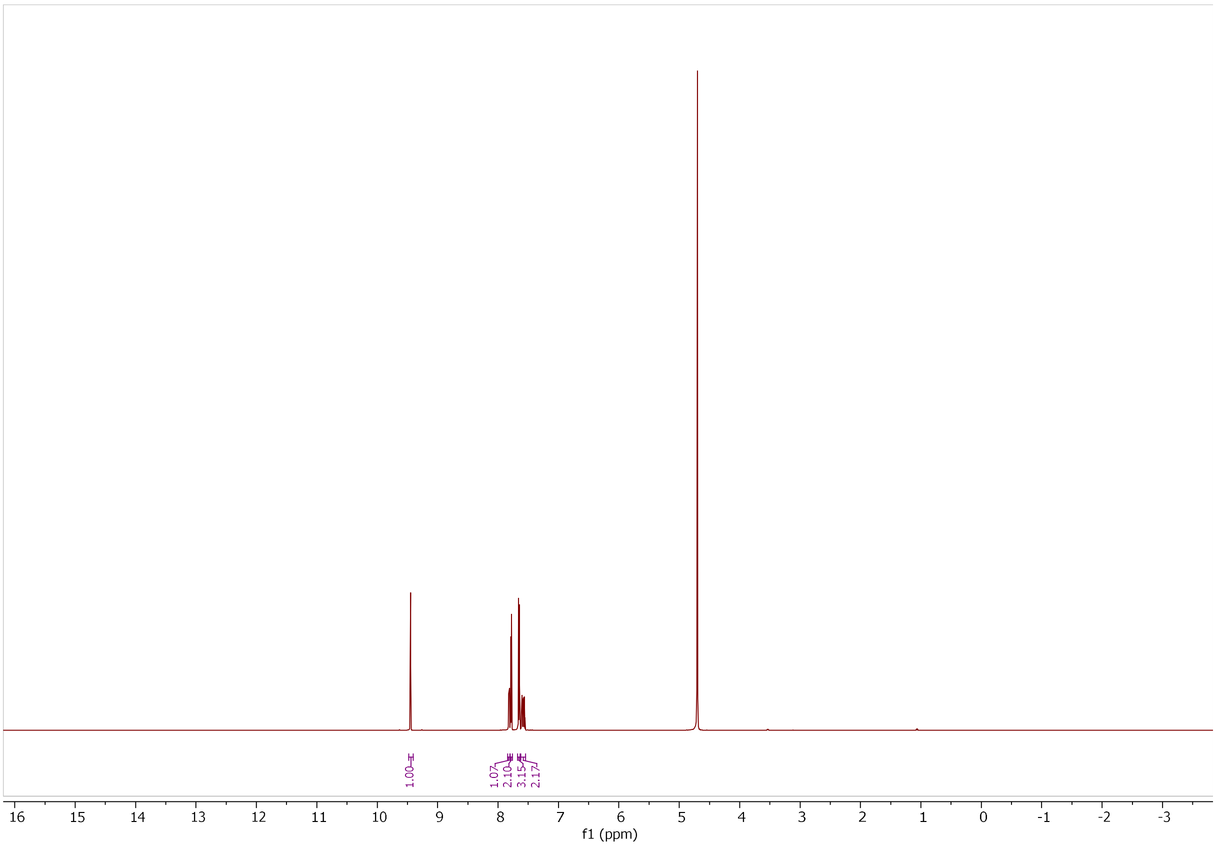

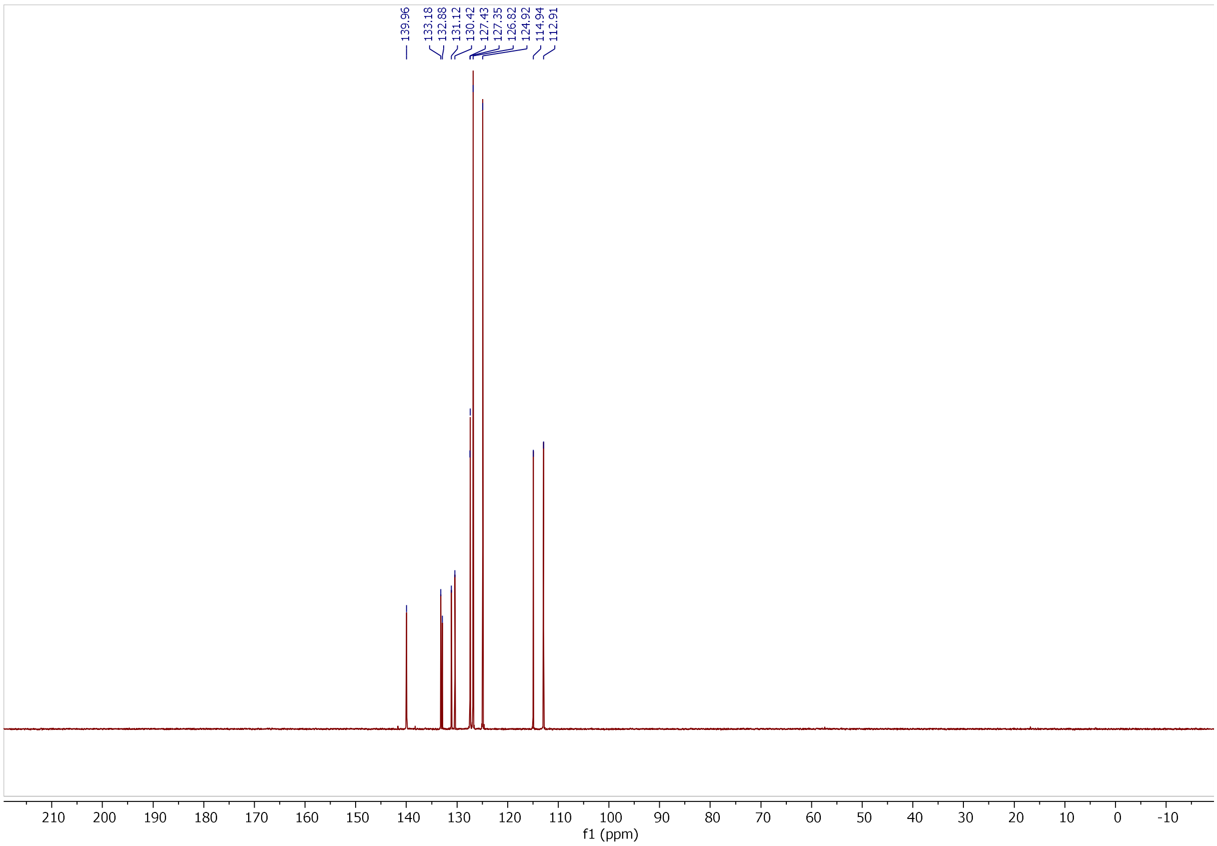
139.96
133.18
132.88
131.12
130.42
127.43
127.35
126.82
124.92
114.94
112.91
210 200 190 180 170 160 150 140 130 120 110 100 90 80 70 60 50 40 30 20 10 0 -10
f1 (ppm)

6-(1H-benzo[d]imidazol-1-yl)pyridin-3-amine (5b)

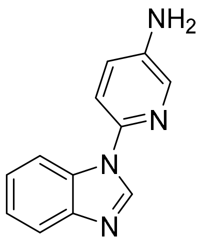

A total of 1.20 g (5 mmol) of **4b** was dissolved in 50 mL of MeOH and hydrogenated at room temperature and pressure overnight using 53 mg (0.05 mmol, 1mol%) of 10% Pd/C. Reaction was filtered from the catalyst and solvent removed on a rotavap. Crude was treated with DCM/ether and resulting solids were filtered. Those were subsequently dissolved in MeOH, leaving dark green residue that was filtered. Obtained filtrate was stripped of solvent to produce the title compound as a yellow solid (0.86 g, 82%).

^{1}H NMR (400 MHz, DMSO-d_6) δ 8.67 (s, 1H), 8.02 – 7.94 (m, 2H), 7.79 – 7.71 (m, 1H), 7.53 (dd, *J* = 8.7, 0.7 Hz, 1H), 7.31 (pd, *J* = 7.2, 1.4 Hz, 2H), 7.22 (dd, *J* = 8.6, 2.9 Hz, 1H), 5.60 (s, 2H). ^{13}C NMR (101 MHz, DMSO-d_6) δ 144.2, 143.7, 141.9, 138.7, 134.2, 132.3, 123.2, 122.8, 122.2, 119.6, 116.1, 112.6.

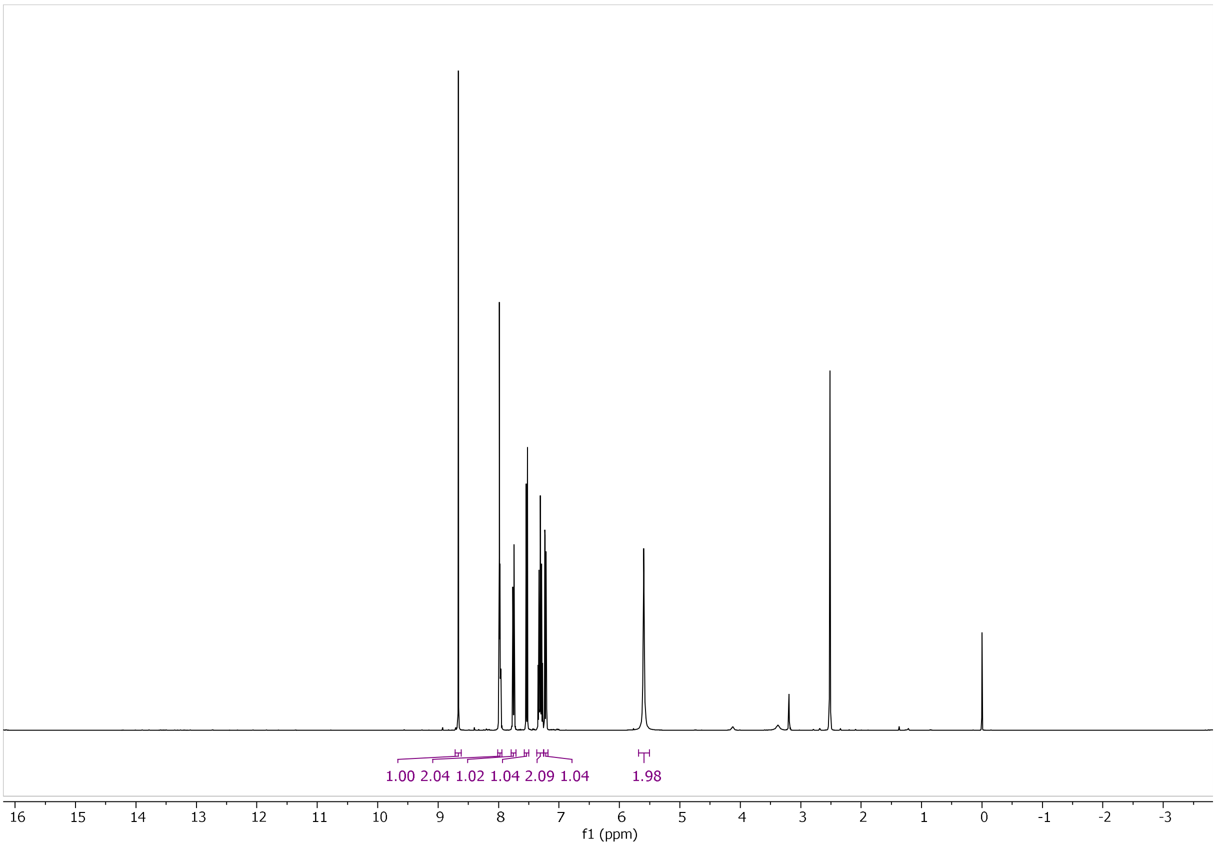

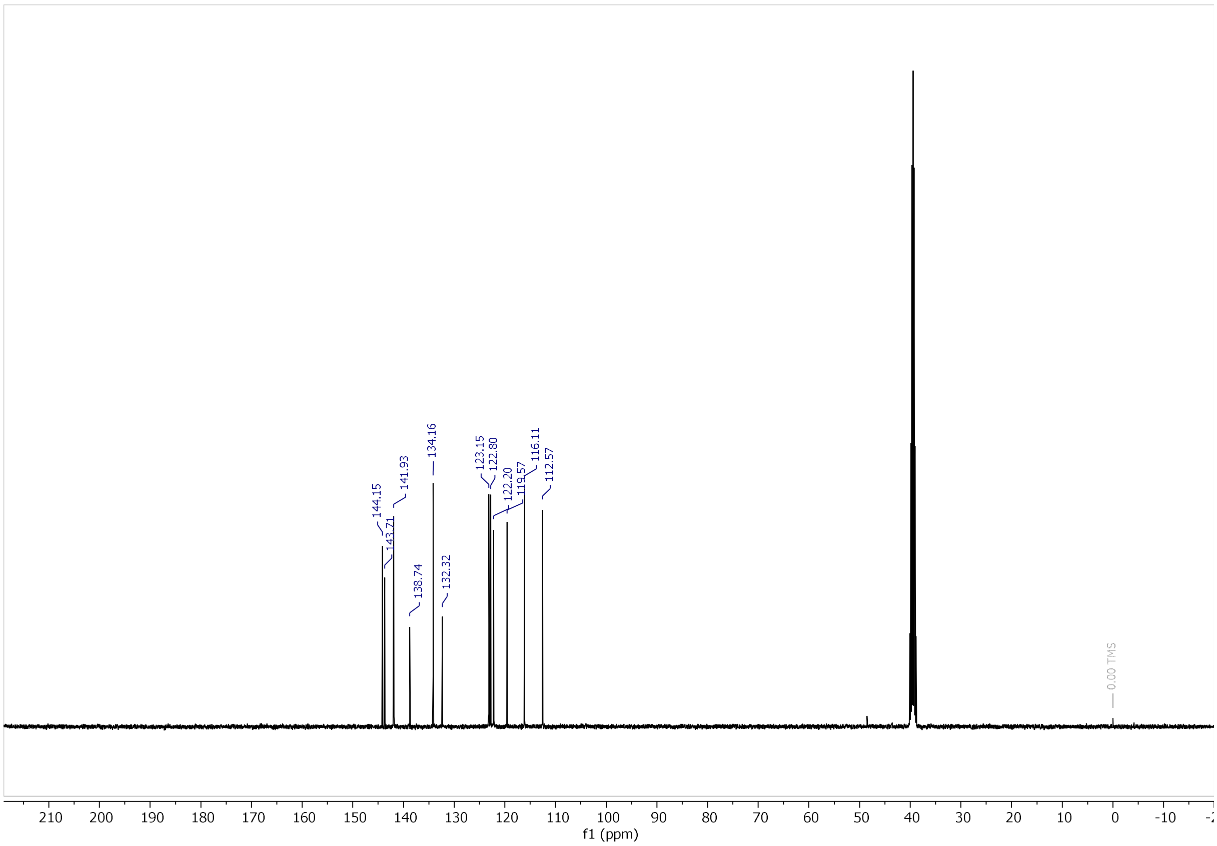
144.15
143.71
141.93
138.74
134.16
132.32
123.15
122.80
122.20
119.57
116.11
112.57
0.00 TMS
210
200
190
180
170
160
150
140
130
120
110
100
90
80
70
60
50
40
30
20
10
0
-10
f1 (ppm)

N-(4-(1H-benzo[d]imidazol-1-yl)phenyl)-1H-imidazol-2-amine (9)

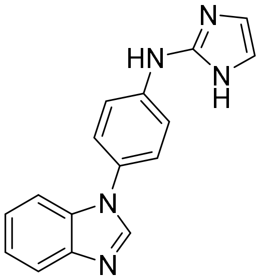

A flame-dried vial was charged with 2-bromo-1*H*-imidazole (147 mg, 1 mmol), **5a** (251 mg, 1.2 mmol), tBuBrettPhos Pd G3 (9 mg, 0.01 mmol) and tBuBrettPhos (5 mg, 0.01 mmol). It was sealed and evacuated/backfilled with argon three times. A total of 2.8 mL of anhydrous THF was added followed by LHMDS (2.2 mL, 2.2 mmol, 1M THF solution). Reaction was stirred at 60 °C overnight. It was then quenched with 10 mL of water and extracted 2 × 15 mL of EtOAc. Combined organics were washed with 20 mL of water, dried with anhydrous $MgSO_4$ and stripped of solvent. Remaining residue was passed through silica eluting 8% 2M NH_3 in MeOH/DCM. Crude was purified by preparative HPLC and processed through an SCX column to obtain the title compound as an off-white solid (40 mg, 15%).

^{1}H NMR (400 MHz, DMSO-d_6) δ 10.86 (s, 1H), 8.96 (s, 1H), 8.43 (s, 1H), 7.80 – 7.73 (m, 1H), 7.65 (d, *J* = 8.8 Hz, 2H), 7.56 – 7.51 (m, 1H), 7.47 (d, *J* = 8.8 Hz, 2H), 7.30 (tt, *J* = 7.2, 5.5 Hz, 2H), 6.75 (s, 2H). ^{13}C NMR (101 MHz, DMSO) δ 144.2, 143.5, 143.3, 142.5, 133.6, 126.9, 124.6, 124.3, 123.1, 122.0, 119.7, 115.9, 112.0, 110.4. HRMS (MALDI): *m*/*z* calculated for [M+H]$^+$ 276.12437, found 276.12464.

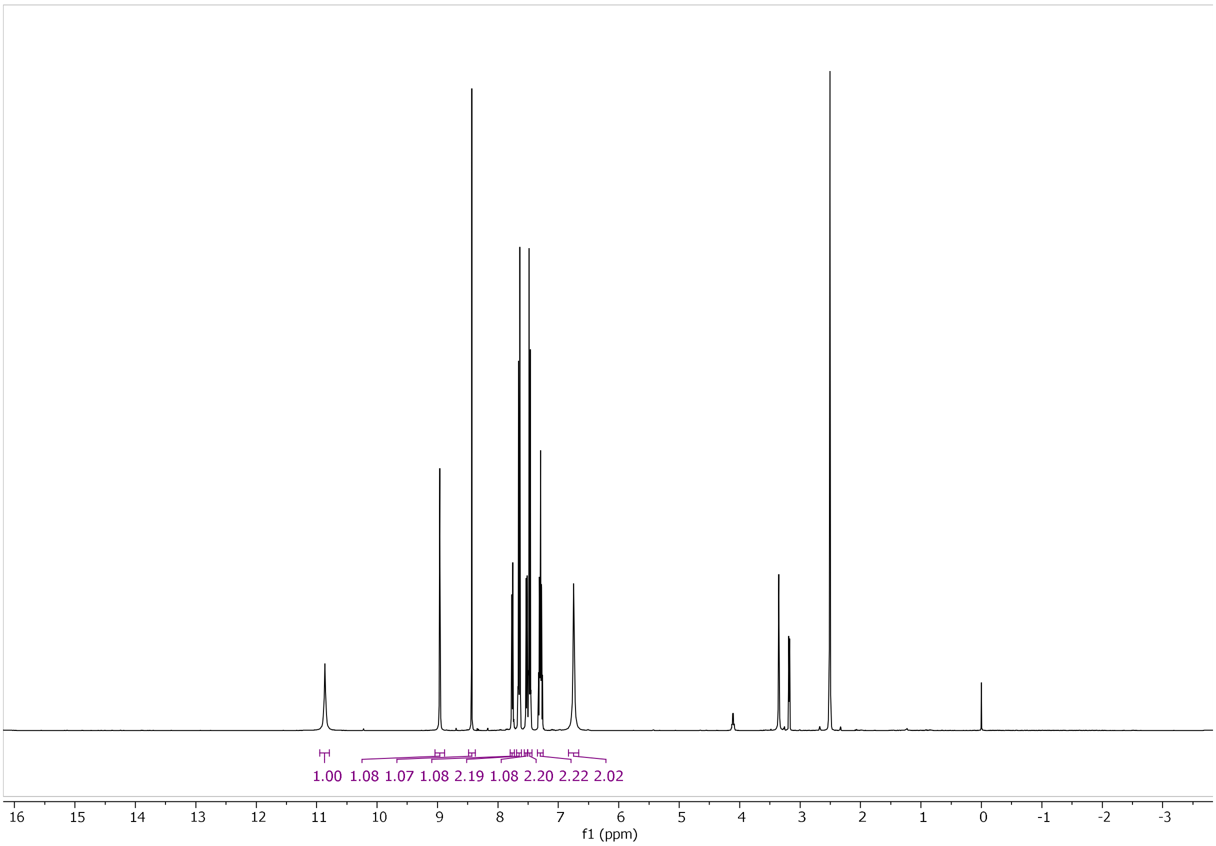

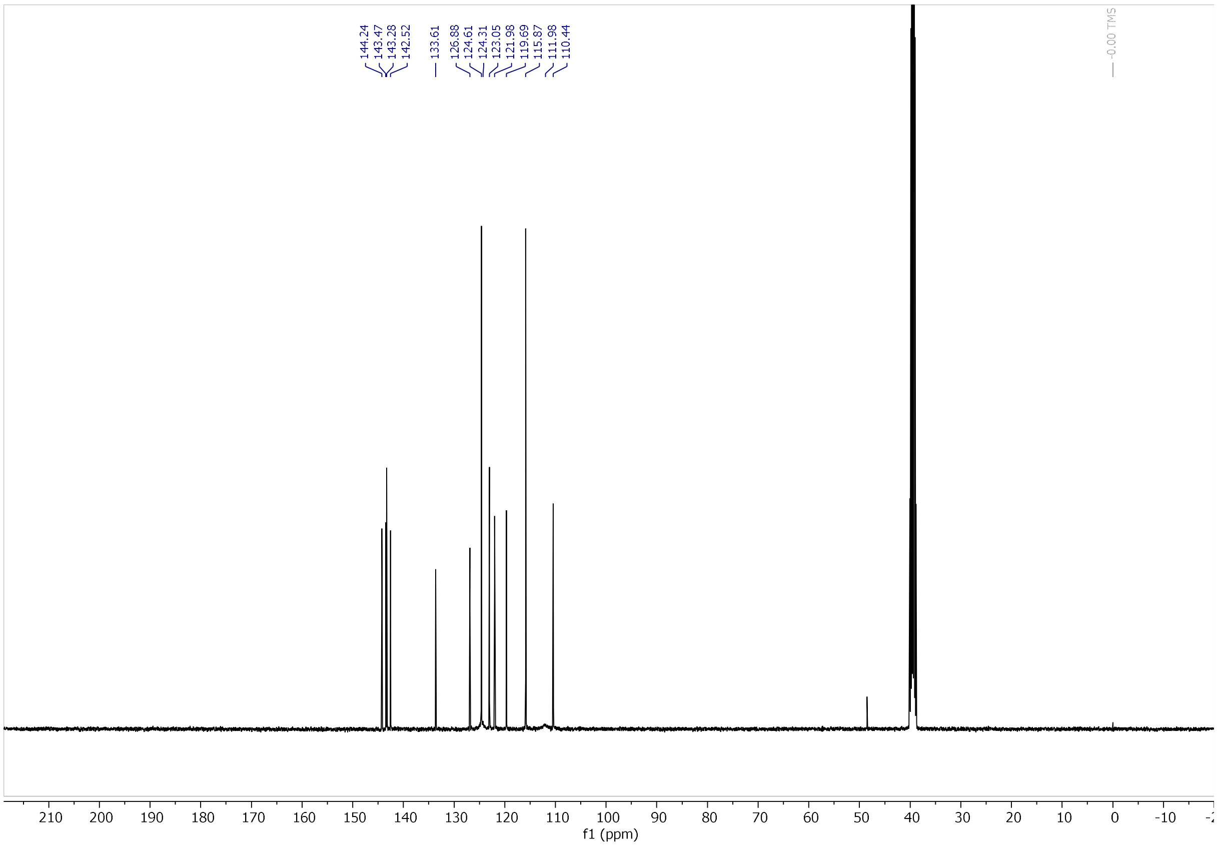

144.24
143.47
143.28
142.52
133.61
126.88
124.61
124.31
123.05
121.98
119.69
115.87
111.98
110.44
-0.00 TMS
210
200
190
180
170
160
150
140
130
120
110
100
90
80
70
60
50
40
30
20
10
0
-10
f1 (ppm)

N-(4-(1H-benzo[d]imidazol-1-yl)phenyl)-1H-imidazol-4-amine (10)

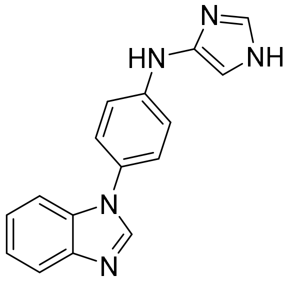

A flame-dried vial was charged with 4-bromo-1*H*-imidazole (147 mg, 1 mmol), **5a** (251 mg, 1.2 mmol), tBuBrettPhos Pd G3 (9 mg, 0.01 mmol) and tBuBrettPhos (5 mg, 0.01 mmol). It was sealed and evacuated/backfilled with argon three times. A total of 2.8 mL of anhydrous THF was added followed by LHMDS (2.2 mL, 2.2 mmol, 1M THF solution). Reaction was stirred at 60 °C for 6 hours. It was then quenched with 10 mL of water and extracted 2 × 15 mL of EtOAc. Combined organics were washed with 20 mL of water, dried with anhydrous $MgSO_4$ and stripped of solvent. Remaining residue was passed through silica eluting 5% 2M NH_3 in MeOH/DCM. Crude was purified by preparative HPLC and processed through an SCX column to obtain the title compound as a grey solid after trituration with ether (23 mg, 8%).

^{1}H NMR (400 MHz, DMSO-d_6) δ 8.40 (s, 1H), 8.36 (s, 1H), 7.80 – 7.72 (m, 1H), 7.56 (d, *J* = 1.3 Hz, 1H), 7.51 – 7.46 (m, 1H), 7.42 – 7.34 (m, 2H), 7.31 – 7.24 (m, 2H), 7.22 (d, *J* = 8.8 Hz, 2H), 6.87 (d, *J* = 1.3 Hz, 1H). ^{13}C NMR (101 MHz, DMSO-d_6) δ 145.5, 143.5, 143.4, 140.4, 133.9, 132.3, 125.4, 125.0, 123.1, 122.0, 119.8, 114.3, 110.6, 102.2. HRMS (MALDI): *m/z* calculated for $[M+H]^+$ 276.12437, found 276.12464.

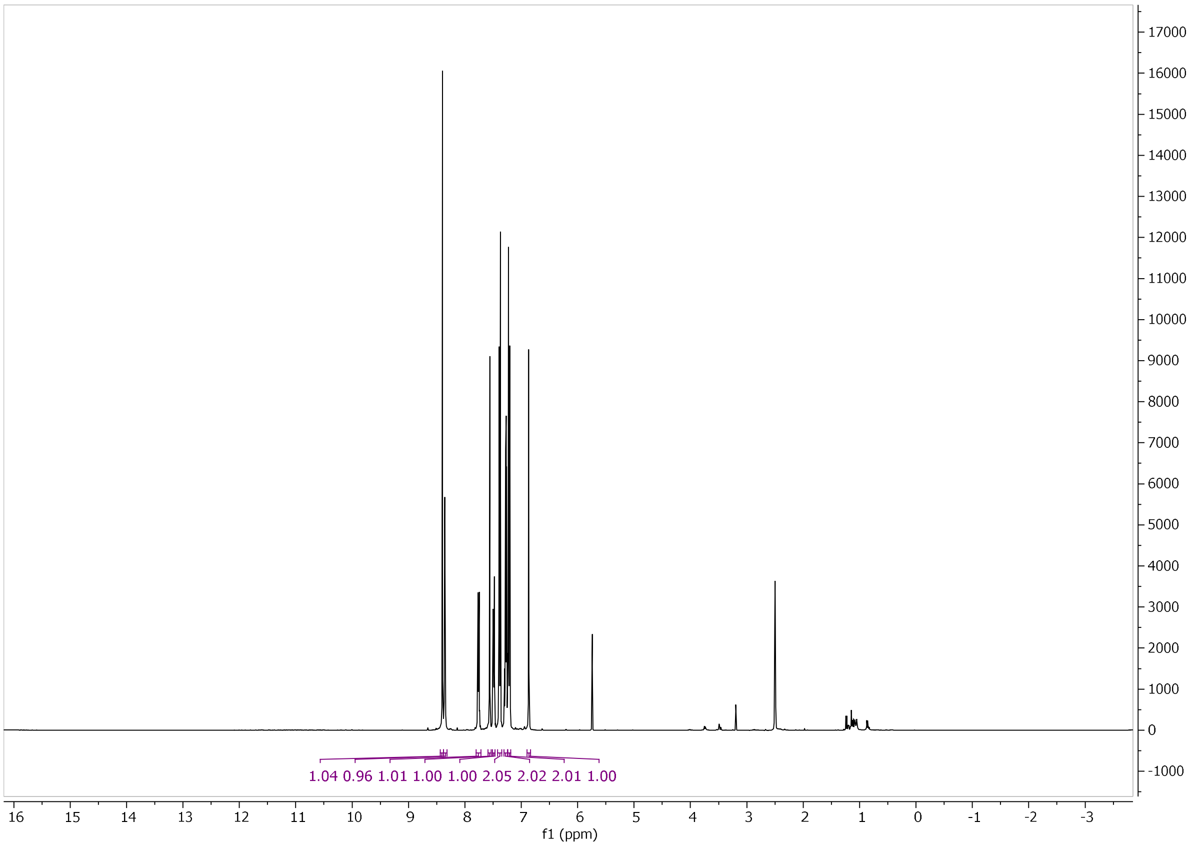

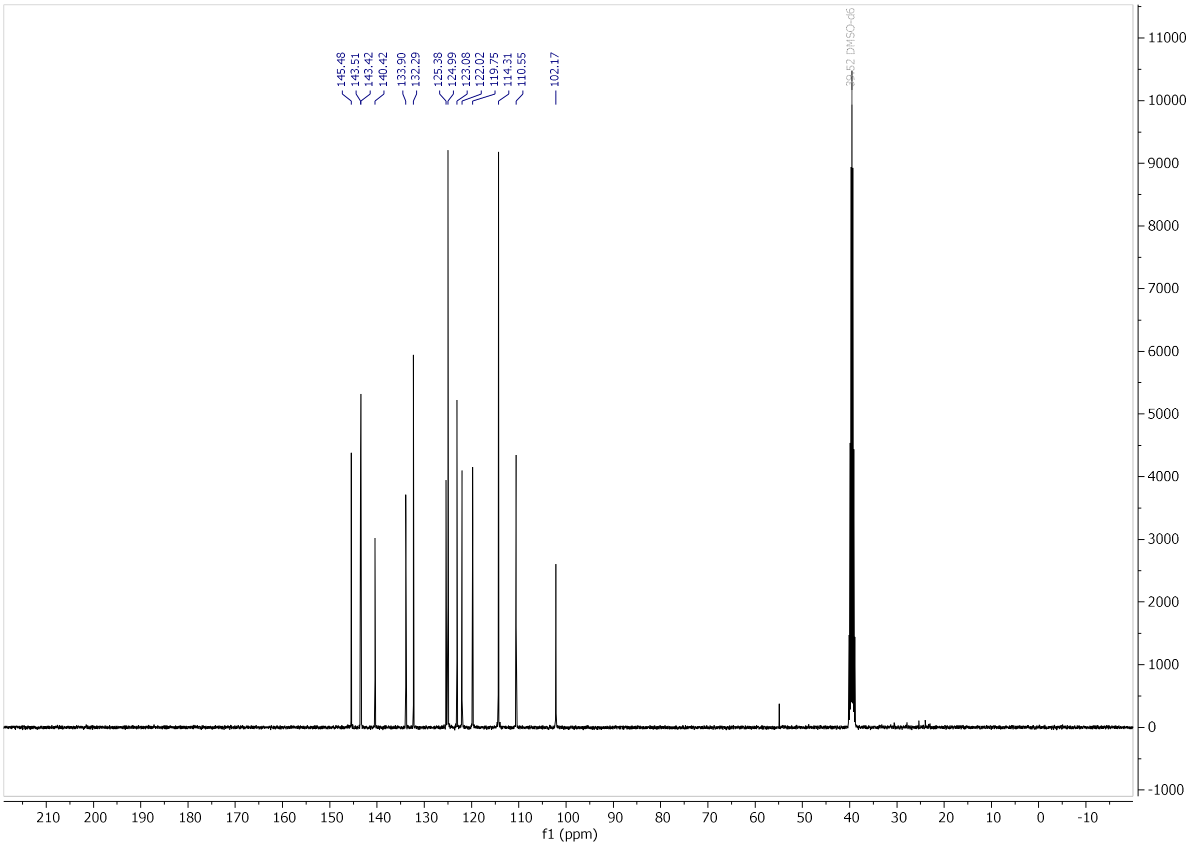

145.48
143.51
143.42
140.42
133.90
132.29
125.38
124.99
123.08
122.02
119.75
114.31
110.55
102.17
39.52 DMSO-d6
f1 (ppm)

6-(1*H*-benzo[*d*]imidazol-1-yl)-*N*-(1*H*-1,2,4-triazol-3-yl)pyridin-3-amine (11)

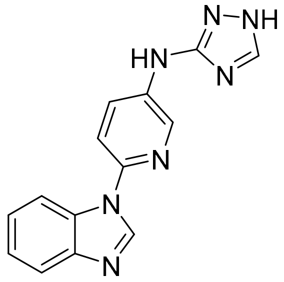

A flame-dried vial was charged with 3-bromo-1*H*-1,2,4-triazole (148 mg, 1 mmol), **5b** (252 mg, 1.2 mmol), tBuBrettPhos Pd G3 (17 mg, 0.02 mmol) and tBuBrettPhos (10 mg, 0.02 mmol). It was sealed and evacuated/backfilled with argon three times. A total of 2.8 mL of anhydrous THF was added followed by LHMDS (2.2 mL, 2.2 mmol, 1M THF solution). Reaction was stirred at 60 °C overnight. It was then quenched with 20 mL of water and extracted 3 × 15 mL of EtOAc. Combined organics were washed with 15 mL of brine, dried with anhydrous $MgSO_4$ and stripped of solvent. The remaining residue was passed through silica eluting 2M NH_3 in MeOH/DCM (5:95 → 15:85). Crude was purified by preparative HPLC and processed through an SCX column to obtain the title compound as an off-white solid (10 mg, 4%).

^{1}H NMR (400 MHz, DMSO-*d*$_6$) δ 13.66 (s, 1H), 9.69 (s, 1H), 8.84 – 8.77 (m, 2H), 8.43 – 8.24 (m, 2H), 8.16 – 8.07 (m, 1H), 7.85 – 7.72 (m, 2H), 7.43 – 7.24 (m, 2H). ^{13}C NMR (101 MHz, DMSO-*d*$_6$) δ 144.4, 142.5, 141.9, 138.2, 136.7, 132.7, 125.5, 124.0, 123.0, 120.2, 115.9, 113.6. HRMS (MALDI): *m*/*z* calculated for $[M+H]^+$ 278.11487, found 278.11513.

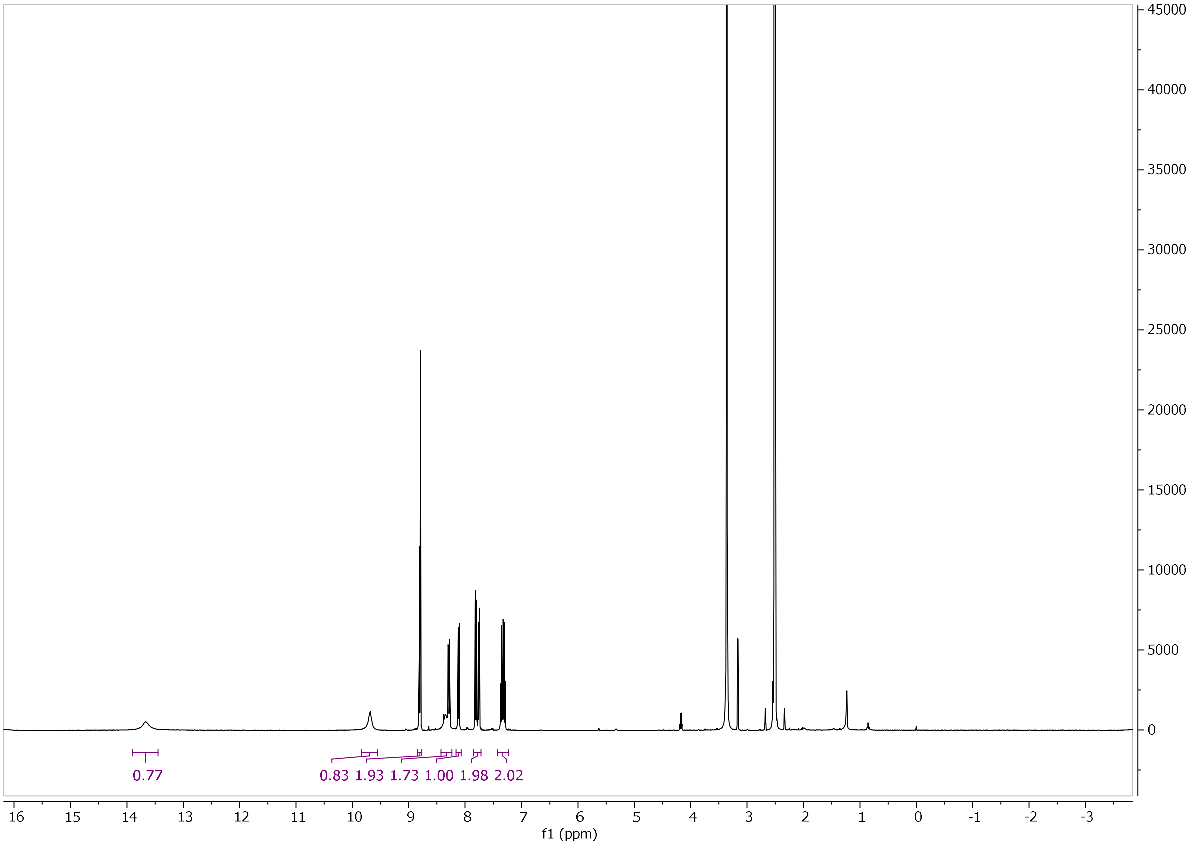

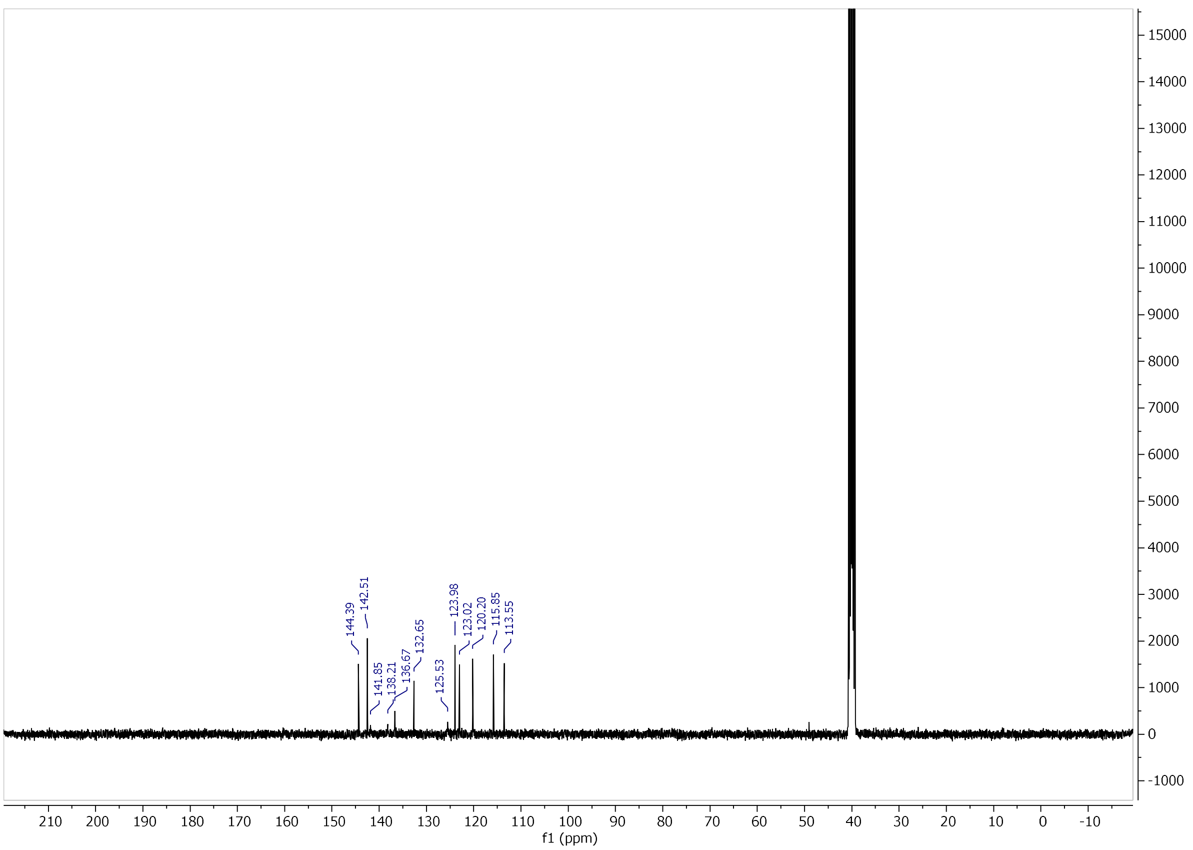
144.39
142.51
141.85
138.21
136.67
132.65
125.53
123.98
123.02
120.20
115.85
113.55
15000
14000
13000
12000
11000
10000
9000
8000
7000
6000
5000
4000
3000
2000
1000
0
-1000
210 200 190 180 170 160 150 140 130 120 110 100 90 80 70 60 50 40 30 20 10 0 -10
f1 (ppm)

Scheme S3 Synthetic Details

4-(1*H*-benzo[*d*]imidazol-1-yl)phenol (6a)

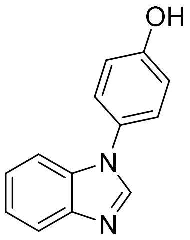

Benzimidazole (1.18 g, 10 mmol), 4-hydroxyphenylboronic acid (2.07 g, 15 mmol), copper (I) sulfide (0.08 g, 0.5 mmol) and *N,N,N,N*-tetramethylethylenediamine (1.50 mL, 10 mmol) were stirred in 40 mL of MeOH at room temperature under an oxygen balloon for 4 hours and later in an open flask overnight. Reaction was filtered and solvent was removed. Residue was taken up in 50 mL of EtOAc and washed subsequently with 15 mL of saturated $NaHCO_3$, 15 mL of EDTA and 15 mL of water. Organics were dried with anhydrous $MgSO_4$ and solvent was removed. Crude was taken up in iPrOH, filtered and stripped of solvent. Remaining residue was triturated with ether to provide the title compound as a livid solid (1.50 g, 71%).

^{1}H NMR (600 MHz, DMSO-*d*$_6$) δ 9.86 (s, 1H), 8.41 (s, 1H), 7.78 – 7.73 (m, 1H), 7.51 – 7.47 (m, 1H), 7.46 – 7.41 (m, 2H), 7.32 – 7.25 (m, 2H), 7.01 – 6.96 (m, 2H). ^{13}C NMR (151 MHz, DMSO-*d*$_6$) δ 157.0, 143.4, 143.3, 133.7, 127.2, 125.5, 123.1, 122.0, 119.7, 116.2, 110.4.

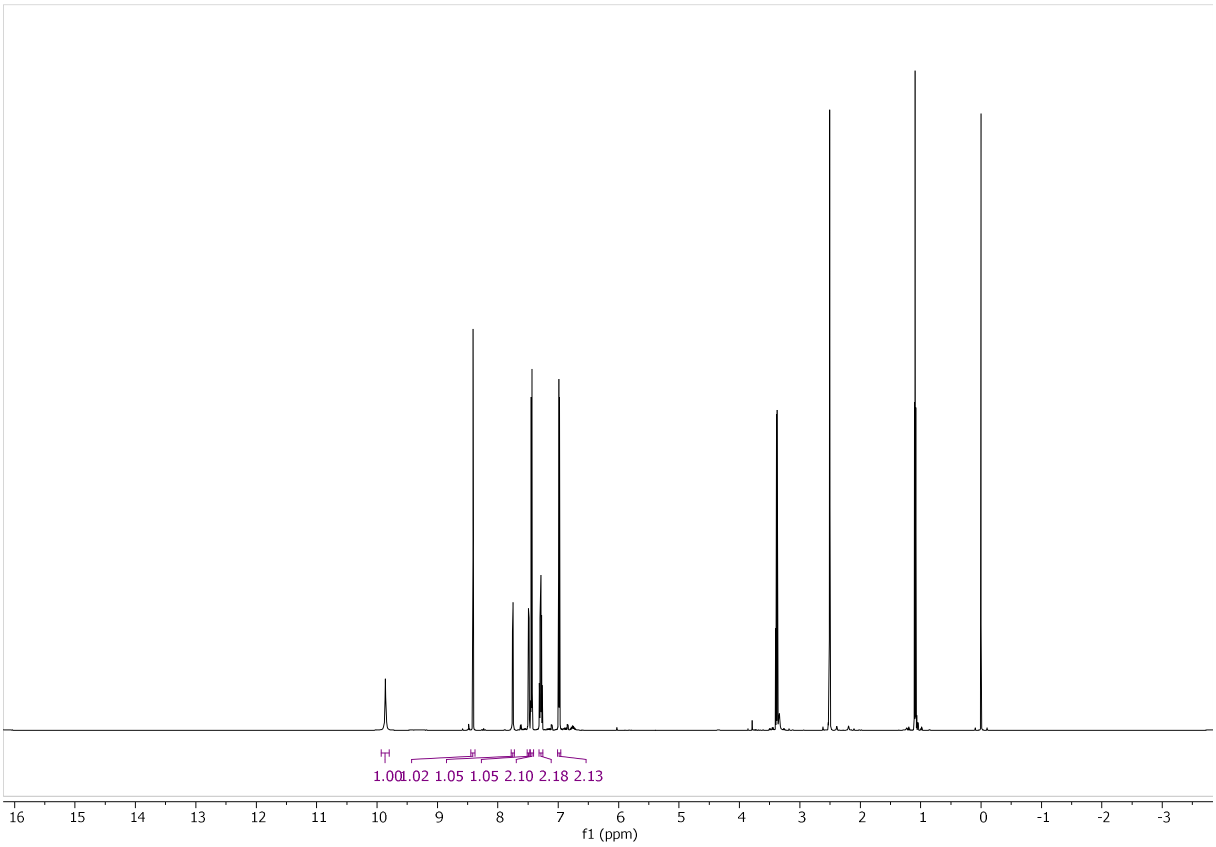

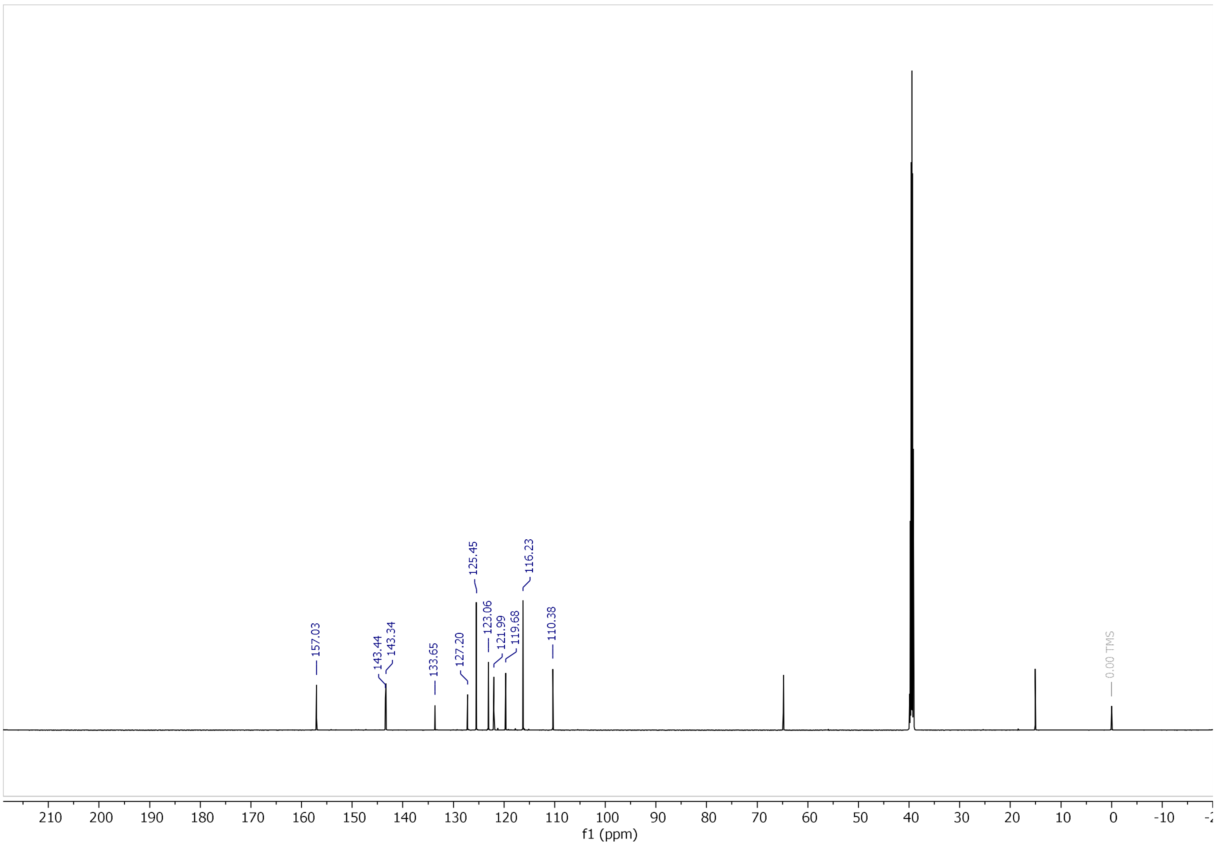
157.03
143.44
143.34
133.65
127.20
125.45
123.06
121.99
119.68
116.23
110.38
0.00 TMS
210 200 190 180 170 160 150 140 130 120 110 100 90 80 70 60 50 40 30 20 10 0 -10
f1 (ppm)

1-(4-(pyridin-4-yloxy)phenyl)-1*H*-benzo[*d*]imidazole (12)

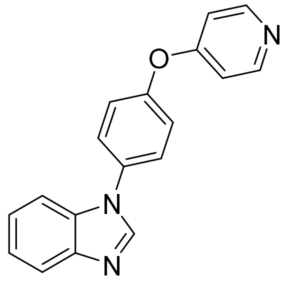

A flame-dried vial was charged with 4-bromopyridine hydrochloride (97 mg, 1 mmol), **6a** (105 mg, 1 mmol), sodium t-butoxide (112 mg, 2 mmol) and 2 mL of anhydrous DMF. It was sealed and evacuated/backfilled with argon three times. Reaction was microwaved at 150 °C for 2 hours. It was then filtered and partitioned between 18 mL of water and 10 mL of DCM. Aqueous was extracted with an additional 10 mL of DCM. Combined organics were washed 4 × 10 mL of water and dried with anhydrous $MgSO_4$. Remaining residue after solvent removal was passed through silica eluting 2M NH_3 in MeOH/DCM (1:99 → 3:97), purified by preparative HPLC and processed through an SCX column to obtain the title compound as an off-white solid after trituration with ether (46 mg, 32%).

^{1}H NMR (600 MHz, DMSO-d_6) δ 8.58 (s, 1H), 8.55 – 8.50 (m, 2H), 7.83 – 7.78 (m, 3H), 7.67 – 7.63 (m, 1H), 7.47 – 7.42 (m, 2H), 7.38 – 7.30 (m, 2H), 7.07 – 7.02 (m, 2H). ^{13}C NMR (151 MHz, DMSO-d_6) δ 163.7, 152.8, 151.6, 143.7, 143.3, 133.1, 133.0, 125.8, 123.4, 122.4, 122.0, 119.9, 112.2, 110.5. HRMS (MALDI): *m*/*z* calculated for $[M+H]^+$ 288.11314, found 288.11341.

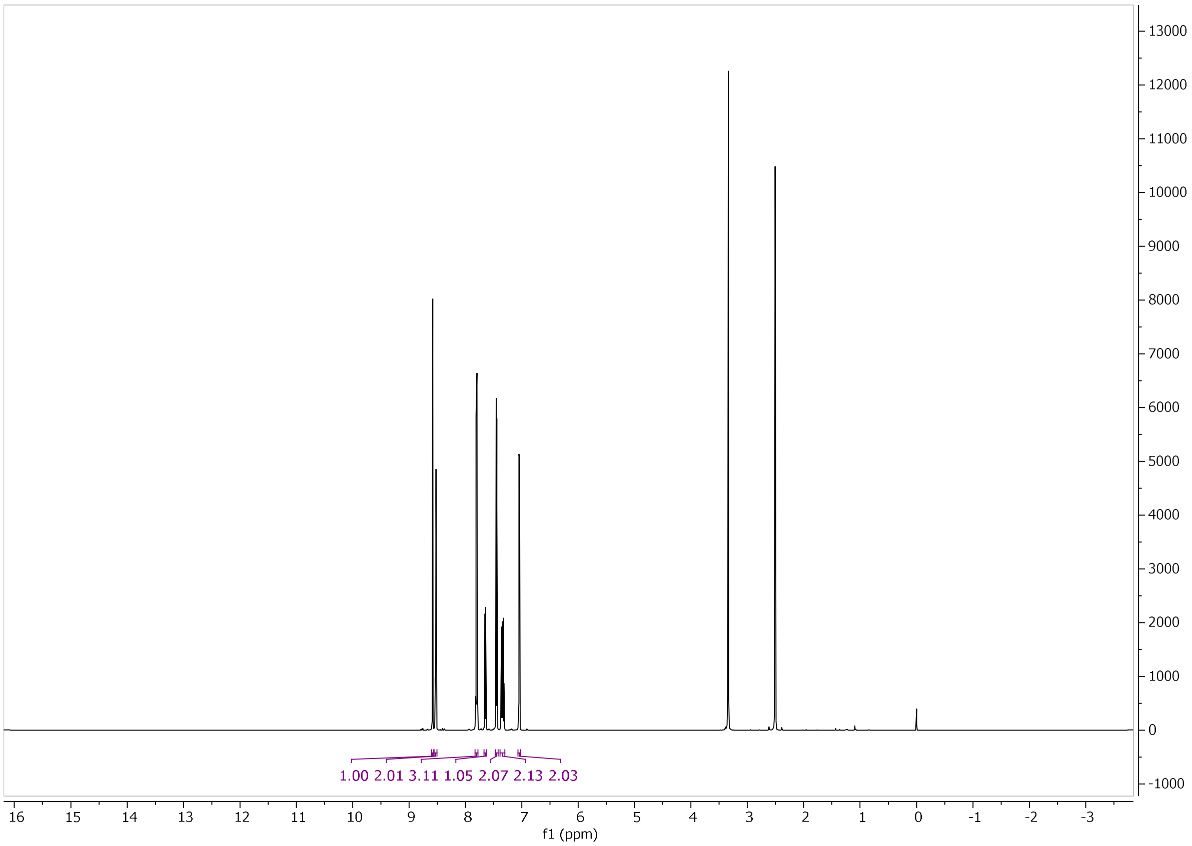

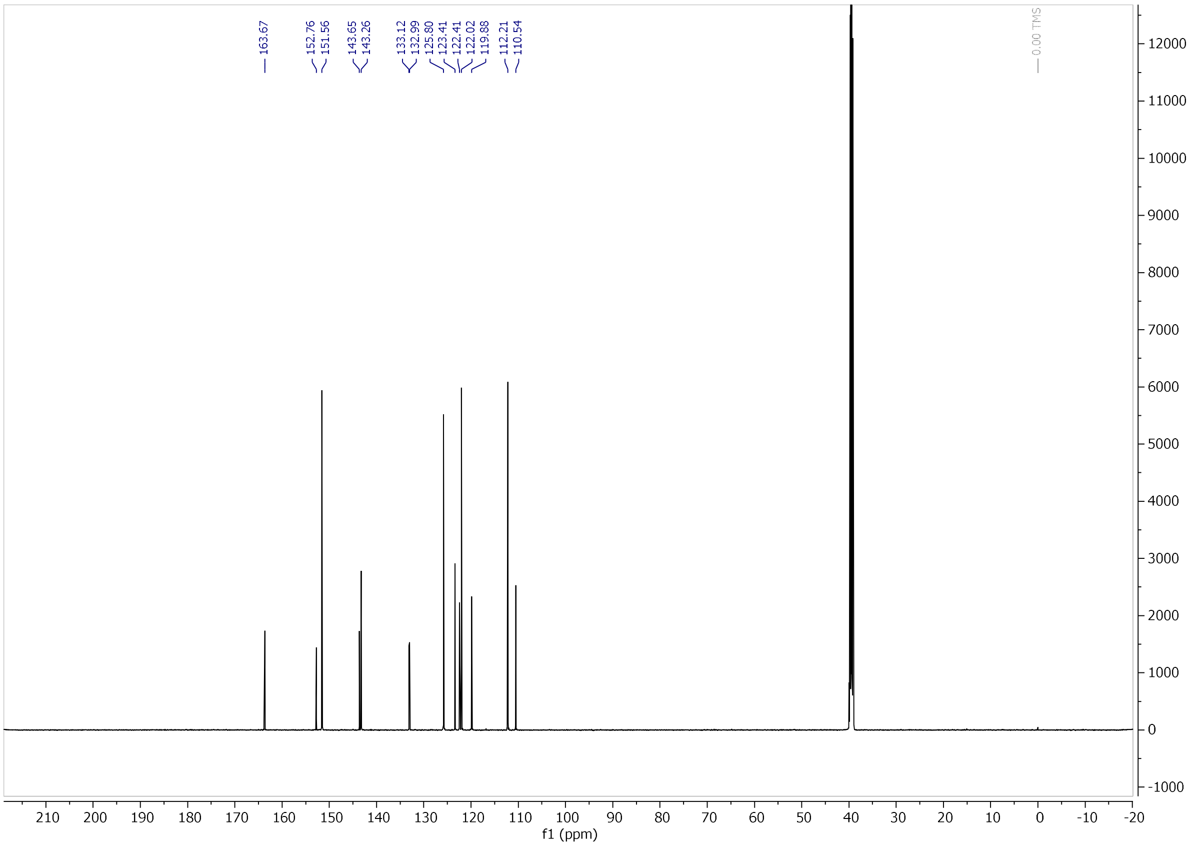

163.67
152.76
151.56
143.65
143.26
133.12
132.99
125.80
123.41
122.41
122.02
119.88
112.21
110.54
0.00 TMS
12000
11000
10000
9000
8000
7000
6000
5000
4000
3000
2000
1000
0
-1000
210
200
190
180
170
160
150
140
130
120
110
100
90
80
70
60
50
40
30
20
10
0
-10
-20
f1 (ppm)

Scheme S4 Synthetic Details

1-(4-bromophenyl)-5-methoxy-1*H*-benzo[*d*]imidazole (2c)

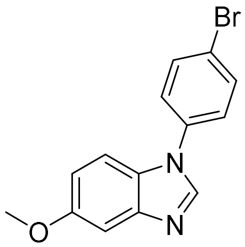

5-Methoxybenzimidazole (0.74 g, 5 mmol), 1-bromo-4-fluorobenzene (1.75 g, 10 mmol) and potassium phosphate (5.31 g, 25 mmol) were stirred in anhydrous DMF (50 mL) overnight at 160 °C. Reaction was partitioned between 50 mL of DCM and 50 mL of water. Aqueous was extracted with 50 mL of DCM, combined organics were washed four times with 50 mL of water and dried with anhydrous Na_2SO_4. Crude was purified by column chromatography eluting with EtOAc/heptane (10:90 → 70:30) to obtain the title compound as a yellow solid (slower eluting, 0.48 g, 32%).

^{1}H NMR (600 MHz, DMSO-d_6) δ 8.51 (s, 1H), 7.83 – 7.78 (m, 2H), 7.67 – 7.63 (m, 2H), 7.52 (d, J = 8.9 Hz, 1H), 7.31 (d, J = 2.4 Hz, 1H), 6.96 (dd, J = 8.9, 2.5 Hz, 1H), 3.82 (s, 3H). ^{13}C NMR (151 MHz, DMSO-d_6) δ 155.9, 144.8, 143.2, 135.3, 132.8, 127.2, 125.2, 120.0, 113.1, 111.0, 102.4, 55.5.

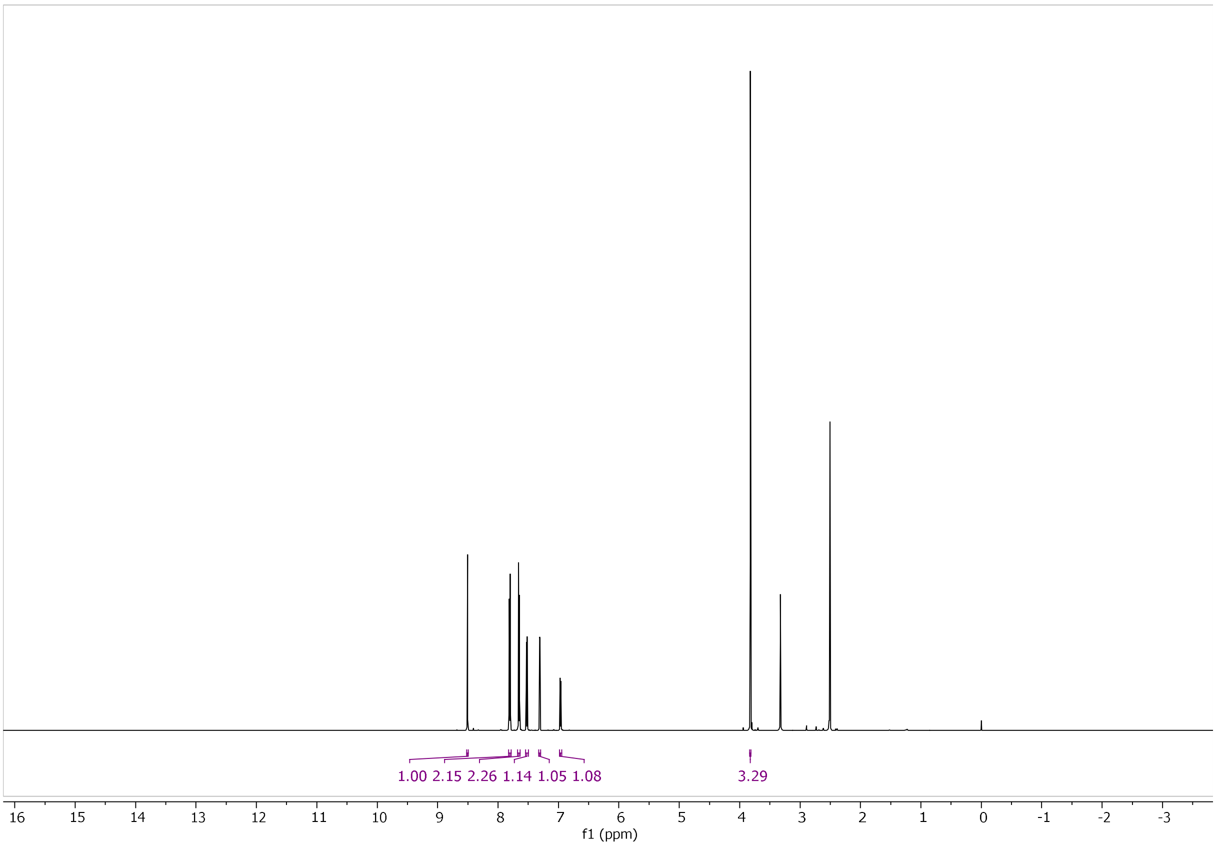

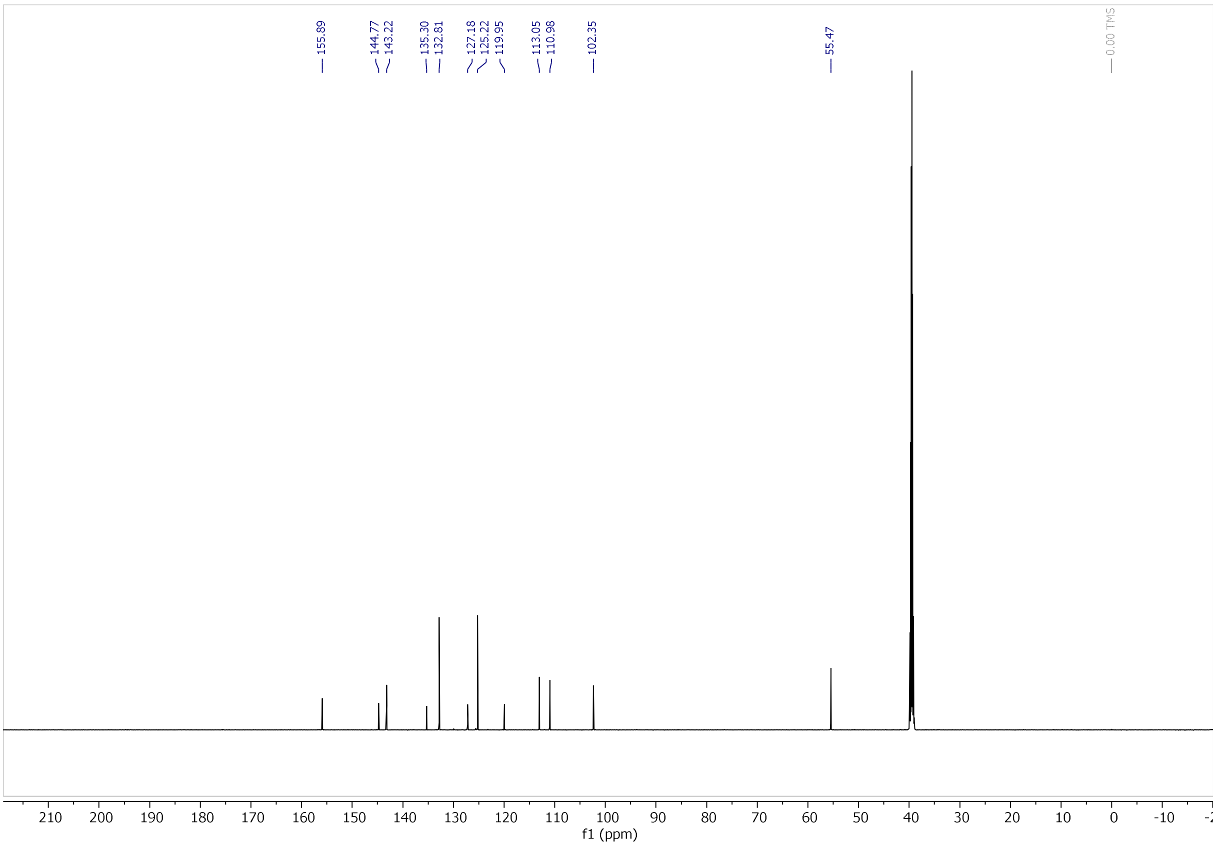

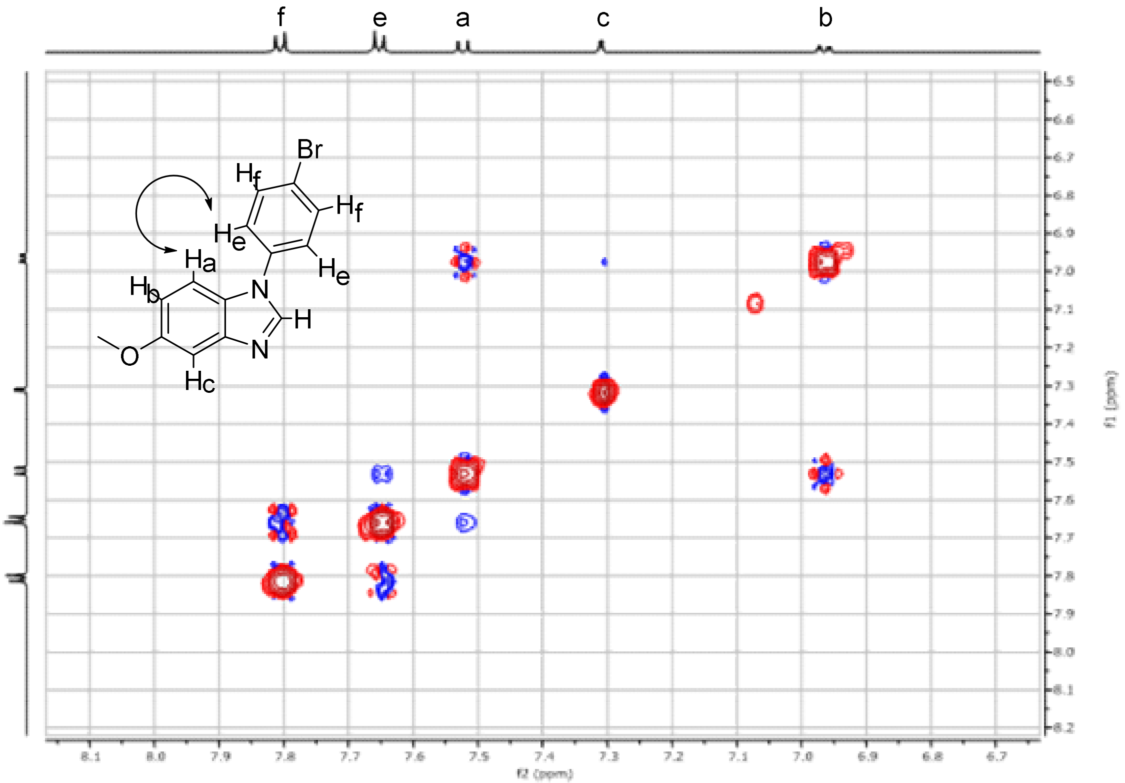

^{1}H-^{1}H NOESY of compound **2c**

key correlation shown with a curved arrow

1-(4-bromophenyl)-6-methoxy-1*H*-benzo[*d*]imidazole (2d)

The title compound was obtained from separation of **2c** as a yellow solid (faster eluting, 0.67 g, 44%).

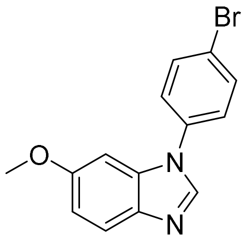

^{1}H NMR (600 MHz, DMSO-d_6) δ 8.41 (s, 1H), 7.84 – 7.80 (m, 2H), 7.69 – 7.64 (m, 3H), 7.08 (d, *J* = 2.4 Hz, 1H), 6.94 (dd, *J* = 8.8, 2.4 Hz, 1H), 3.80 (s, 3H). ^{13}C NMR (151 MHz, DMSO-d_6) δ 156.7, 142.2, 138.0, 135.3, 133.4, 132.9, 125.6, 120.4, 120.2, 112.0, 93.8, 55.5.

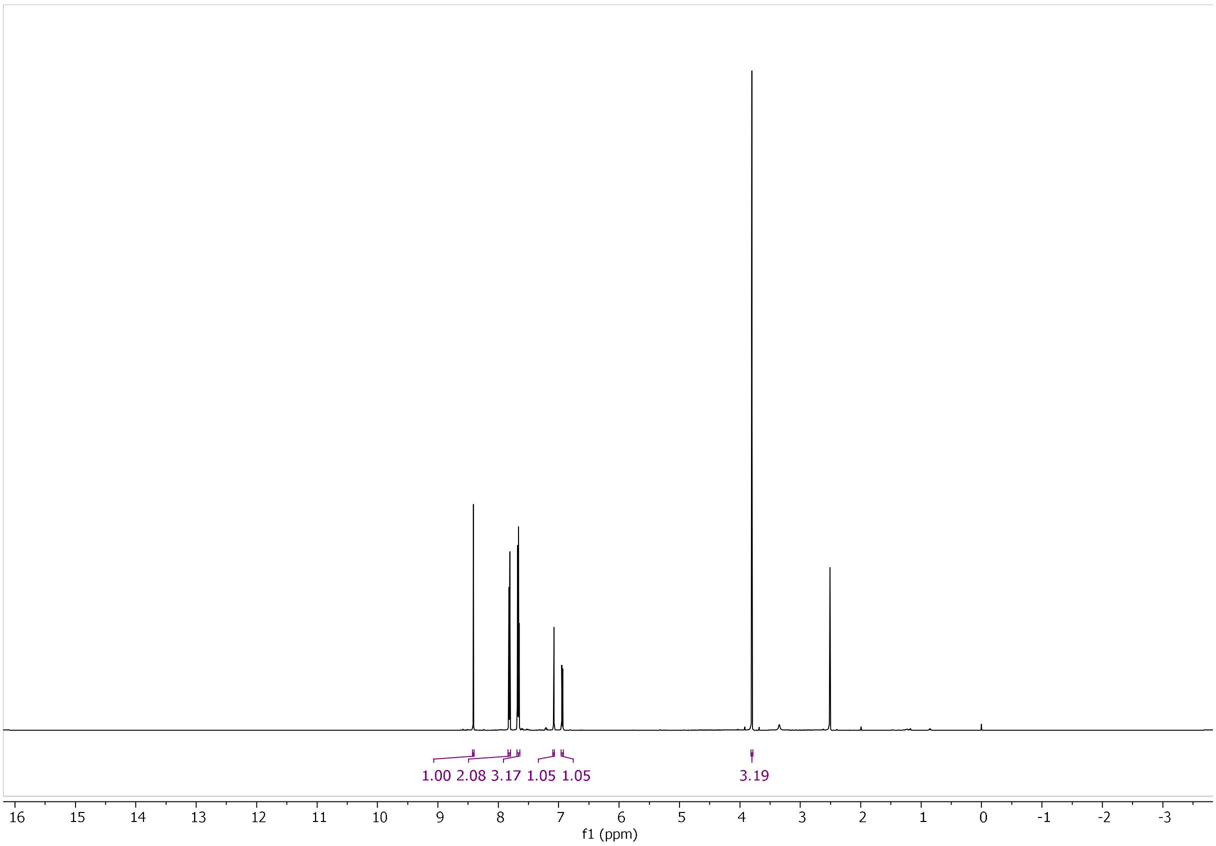

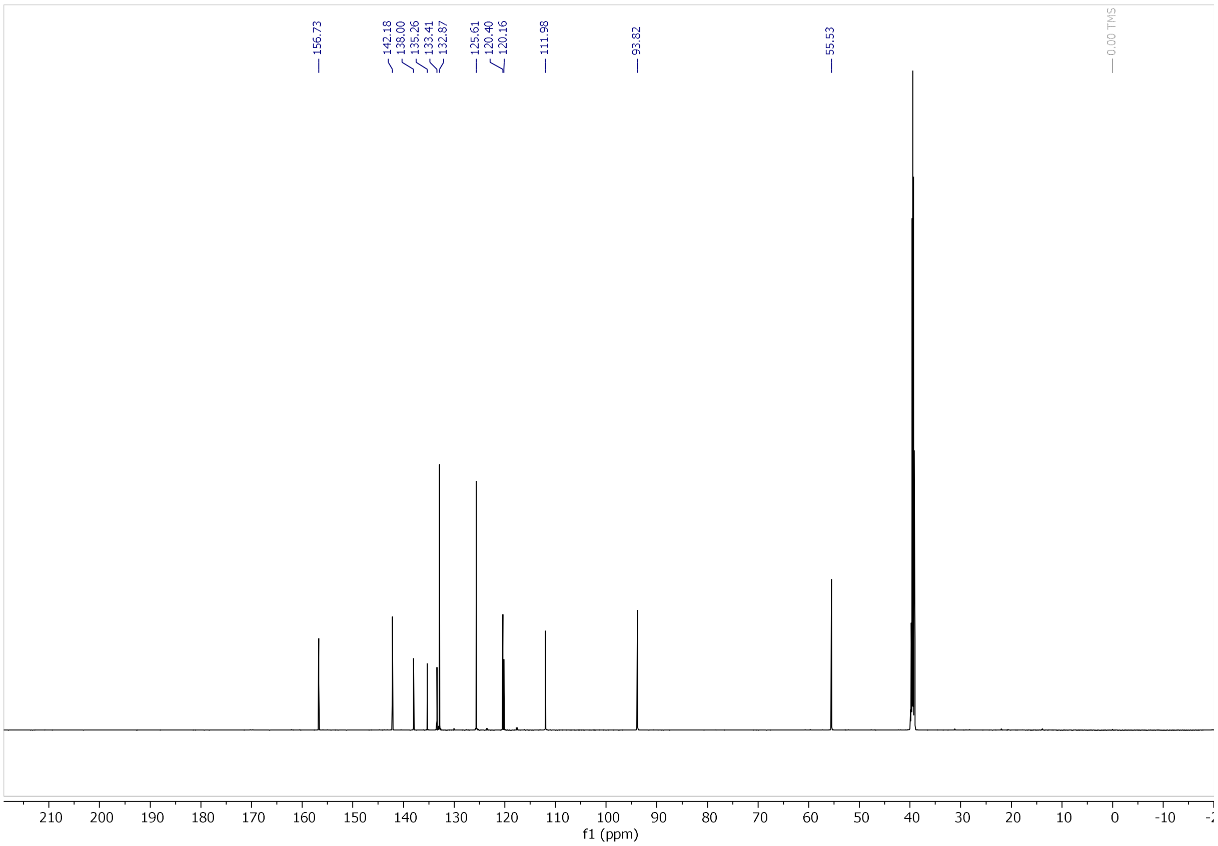

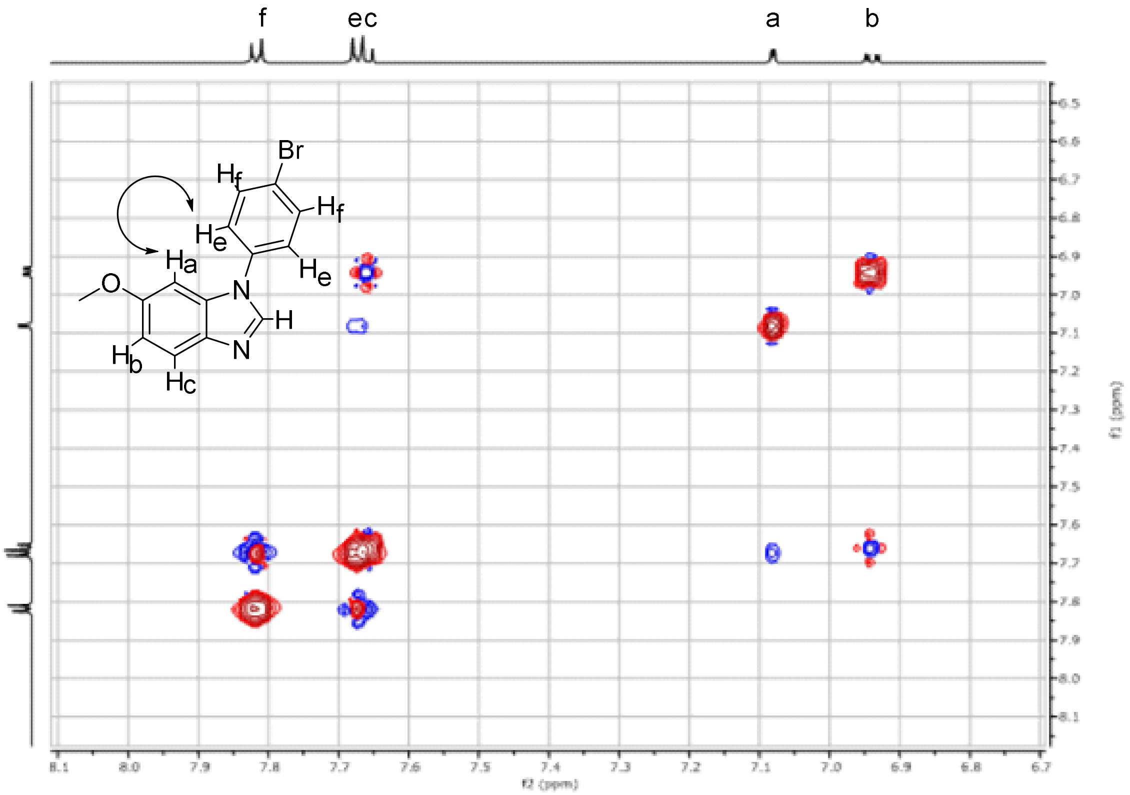

^1H-^1H NOESY of compound **2d**
key correlation shown with a curved arrow

N-(4-(5-methoxy-1H-benzo[d]imidazol-1-yl)phenyl)pyridin-4-amine (13)

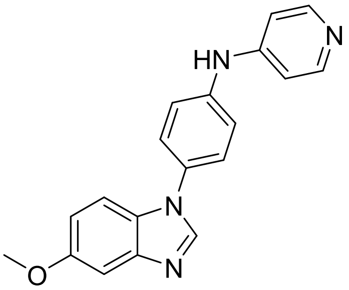

A flame-dried vial was charged with **2c** (152 mg, 0.5 mmol), 4-aminopyridine (57 mg, 0.6 mmol), tBuXPhos Pd G1 (17 mg, 0.025 mmol), tBuXPhos (11 mg, 0.025 mmol) and sodium t-butoxide (96 mg, 1 mmol). It was sealed and evacuated/backfilled with argon three times. A total of 5 mL of anhydrous t-BuOH was added and the reaction was stirred at 70 °C overnight. Solvent was removed and the residue was partitioned between 5 mL of water and 10 mL of EtOAc. Aqueous was extracted with 2 × 10 mL of EtOAc and combined organics dried with anhydrous Na_2SO_4. Crude was passed through a silica column eluting with 5% MeOH in EtOAc + 2% TEA and purified by preparative HPLC to obtain the title compound as a white solid (18 mg, 11%).

^{1}H NMR (600 MHz, DMSO-d_6) δ 9.03 (s, 1H), 8.44 (s, 1H), 8.27 – 8.23 (m, 2H), 7.63 – 7.59 (m, 2H), 7.49 (d, J = 8.8 Hz, 1H), 7.42 – 7.39 (m, 2H), 7.30 (d, J = 2.4 Hz, 1H), 7.01 – 6.97 (m, 2H), 6.95 (dd, J = 8.8, 2.4 Hz, 1H), 3.82 (s, 3H). ^{13}C NMR (151 MHz, DMSO-d_6) δ 155.7, 150.1, 149.5, 144.5, 143.3, 140.0, 130.1, 127.8, 124.5, 120.6, 112.9, 110.9, 109.4, 102.2, 55.5. HRMS (MALDI): m/z calculated for $[M+H]^+$ 317.13969, found 317.13961.

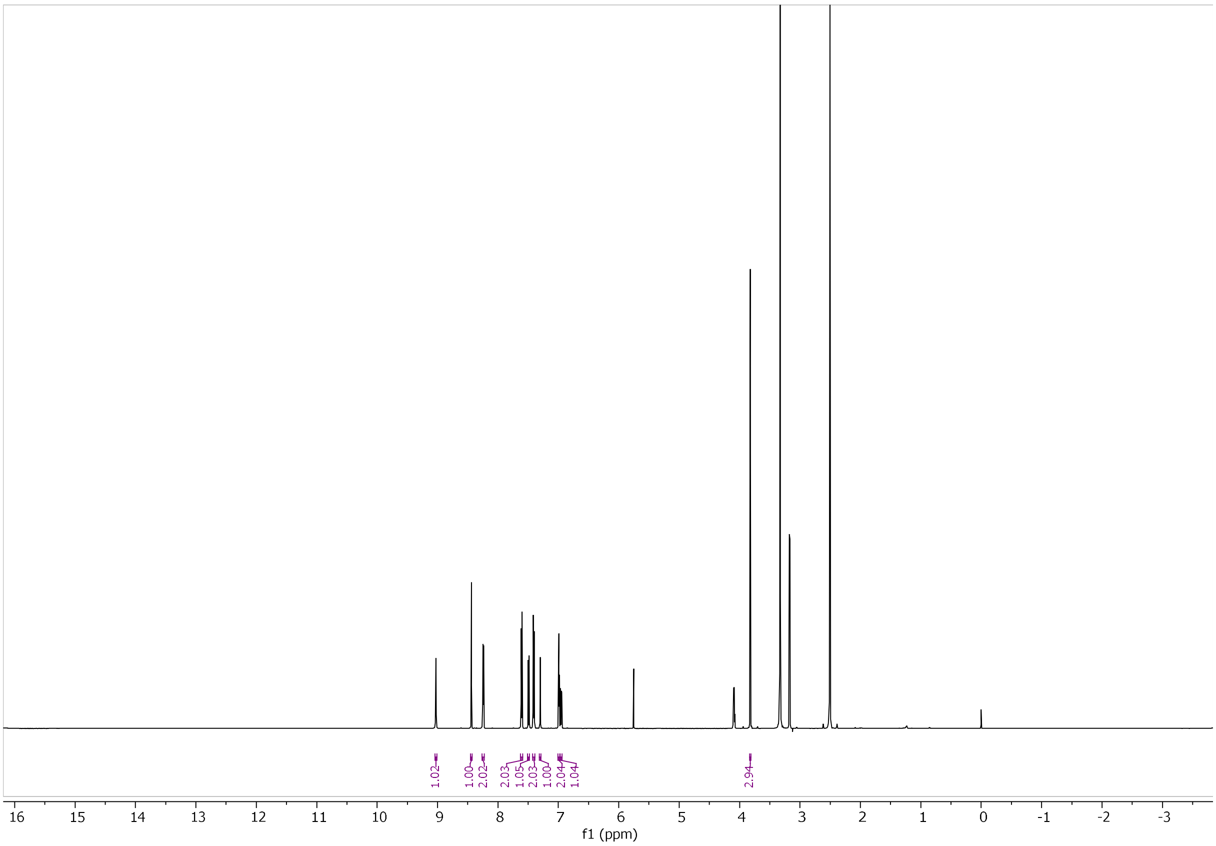

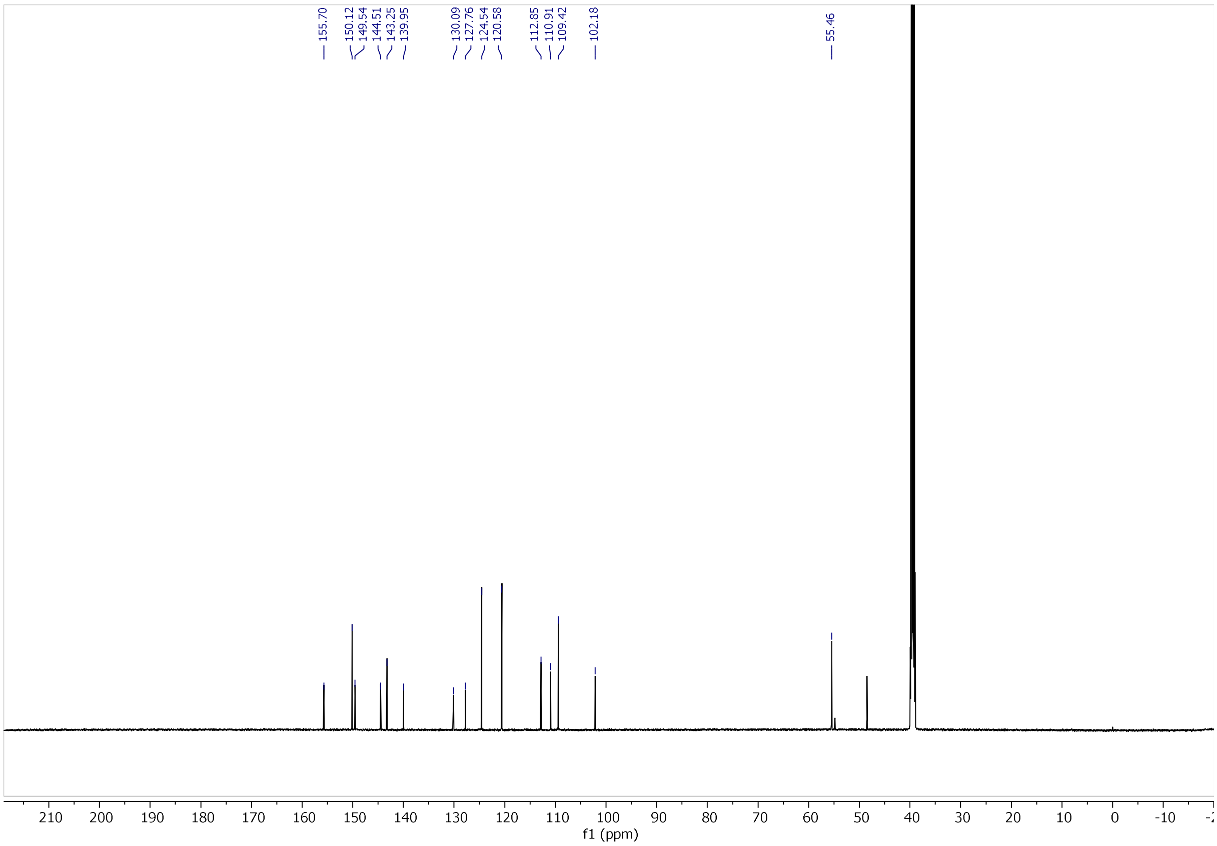
155.70
150.12
149.54
144.51
143.25
139.95
130.09
127.76
124.54
120.58
112.85
110.91
109.42
102.18
55.46
210 200 190 180 170 160 150 140 130 120 110 100 90 80 70 60 50 40 30 20 10 0 -10
f1 (ppm)

N-(4-(6-methoxy-1H-benzo[d]imidazol-1-yl)phenyl)pyridin-4-amine (14)

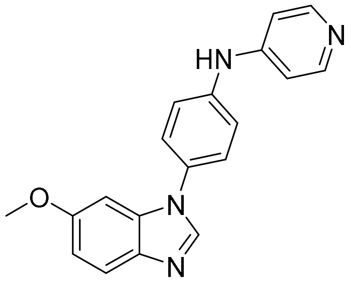

A flame-dried vial was charged with **2d** (152 mg, 0.5 mmol), 4-aminopyridine (57 mg, 0.6 mmol), tBuXPhos Pd G1 (17 mg, 0.025 mmol), tBuXPhos (11 mg, 0.025 mmol) and sodium t-butoxide (96 mg, 1 mmol). It was sealed and evacuated/backfilled with argon three times. A total of 5 mL of anhydrous t-BuOH was added and the reaction was stirred at 70 °C overnight. Solvent was removed and the residue was partitioned between 5 mL of water and 10 mL of EtOAc. Aqueous was extracted with 2 × 10 mL of EtOAc and combined organics dried with anhydrous Na_2SO_4. Crude was passed through a silica column eluting with 5% MeOH in EtOAc + 2% TEA and purified by preparative HPLC to obtain the title compound as a white solid (68 mg, 43%).

^{1}H NMR (600 MHz, DMSO-d_6) δ 9.07 (s, 1H), 8.36 (s, 1H), 8.28 – 8.23 (m, 2H), 7.68 – 7.60 (m, 3H), 7.45 – 7.39 (m, 2H), 7.05 (d, *J* = 2.4 Hz, 1H), 7.03 – 6.99 (m, 2H), 6.93 (dd, *J* = 8.8, 2.4 Hz, 1H), 3.80 (s, 3H). ^{13}C NMR (151 MHz, DMSO-d_6) δ 156.6, 150.0, 149.6, 142.3, 140.0, 137.9, 133.9, 130.0, 124.8, 120.6, 120.3, 111.7, 109.5, 93.7, 55.5. HRMS (MALDI): *m/z* calculated for $[M+H]^+$ 317.13969, found 317.13972.

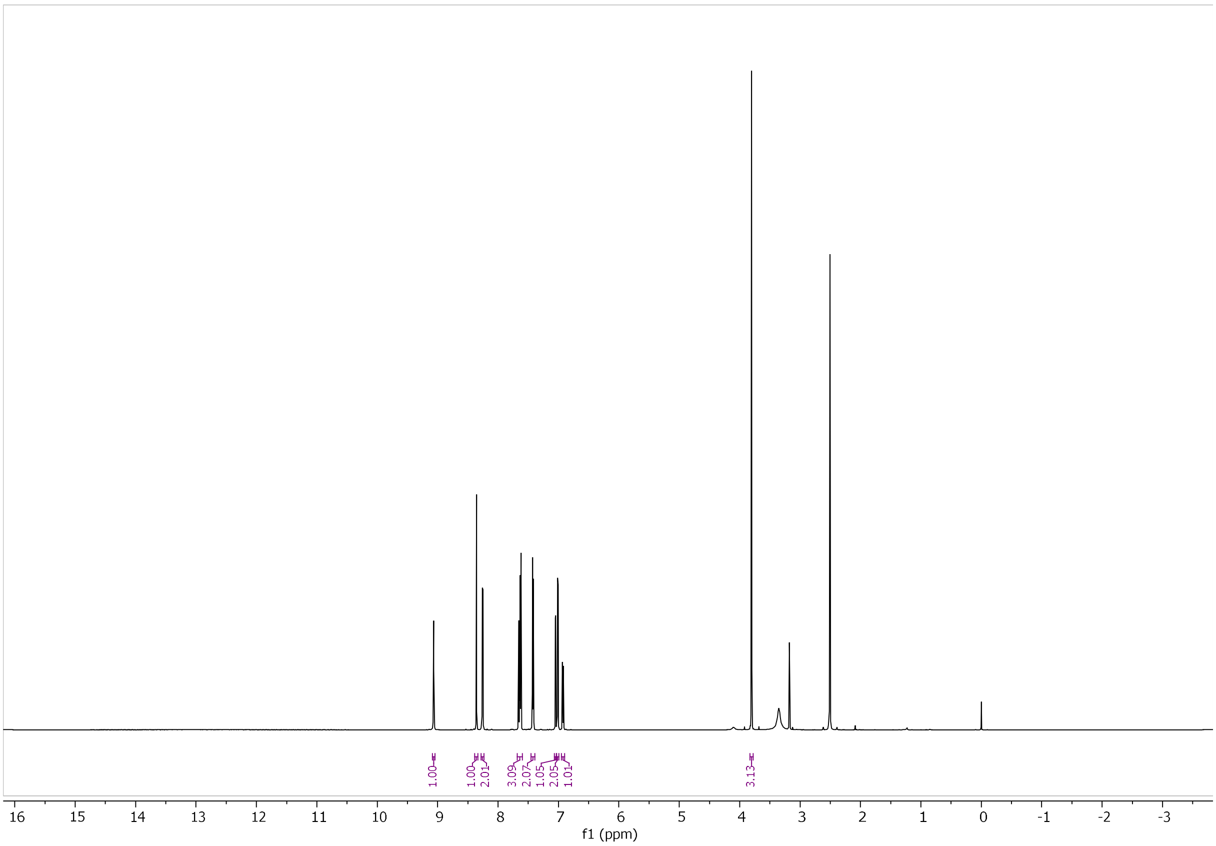

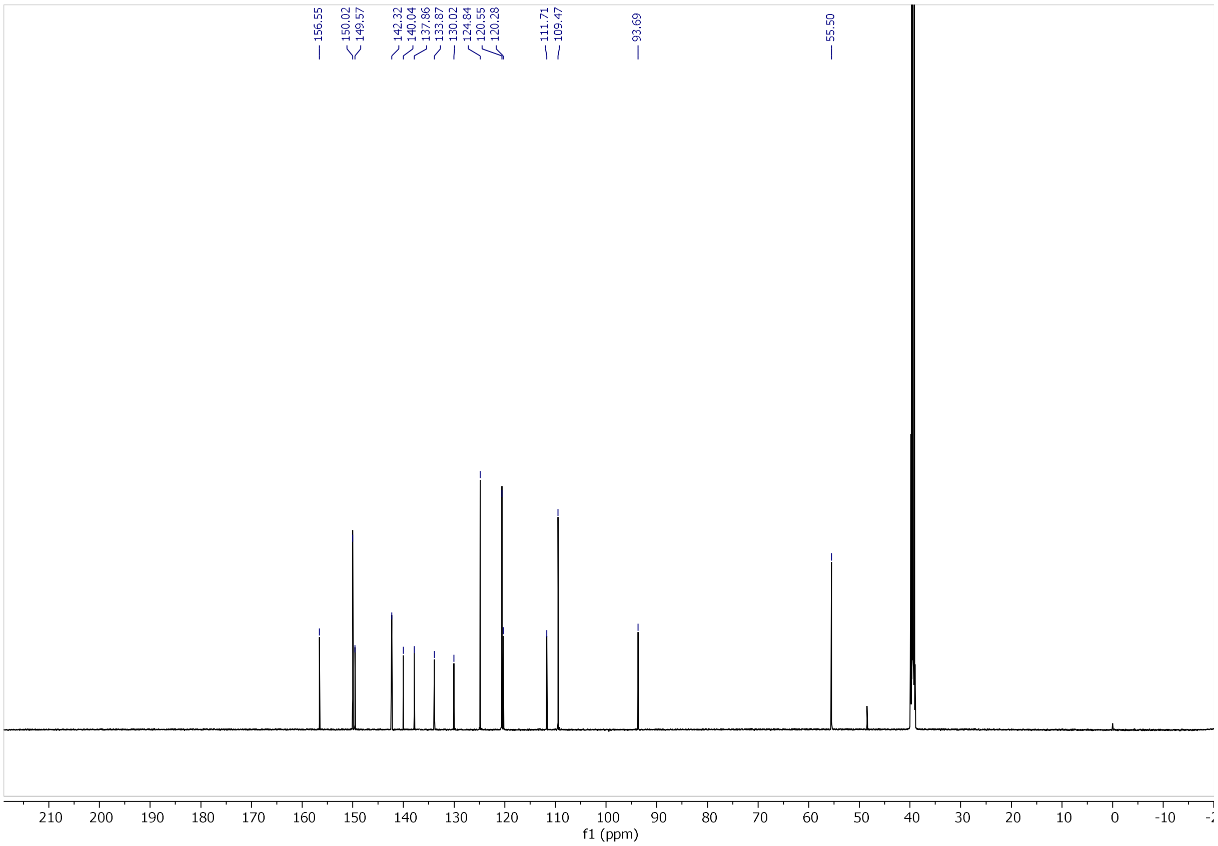

156.55
150.02
149.57
142.32
140.04
137.86
133.87
130.02
124.84
120.55
120.28
111.71
109.47
93.69
55.50
210 200 190 180 170 160 150 140 130 120 110 100 90 80 70 60 50 40 30 20 10 0 -10
f1 (ppm)

1-(4-bromophenyl)-1*H*-benzo[*d*]imidazol-5-amine (2e)

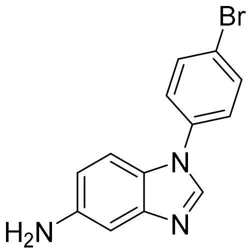

5-Aminobenzimidazole (13.32 g, 100 mmol), 1-bromo-4-fluorobenzene (19.25 g, 110 mmol) and potassium phosphate (42.45 g, 200 mmol) were stirred in anhydrous DMF (400 mL) overnight at 160 °C. Reaction was partitioned between 200 mL of DCM and 400 mL of water. Aqueous was extracted three times with 100 mL of DCM, combined organics were washed three times with 200 mL of brine and dried with anhydrous $MgSO_4$. Crude was triturated with 50 mL of EtOAc, filtered and purified by column chromatography eluting with MeOH/EtOAc (2:98 → 6:94). Obtained compound was treated with 1M HCl in ether and resulting precipitate was filtered and washed with MeOH to obtain the title compound as an off-white solid (slower eluting, 1.76 g, 5% as HCl salt).

^{1}H NMR (600 MHz, D_2O) δ 9.03 (s, 1H), 7.68 (d, *J* = 2.0 Hz, 1H), 7.67 – 7.63 (m, 2H), 7.59 (d, *J* = 8.9 Hz, 1H), 7.41 – 7.38 (m, 2H), 7.35 (dd, *J* = 8.9, 2.1 Hz, 1H). ^{13}C NMR (151 MHz, D_2O) δ 142.1, 135.1, 133.3, 132.6, 131.9, 130.2, 125.8, 123.3, 120.1, 113.8, 109.1.

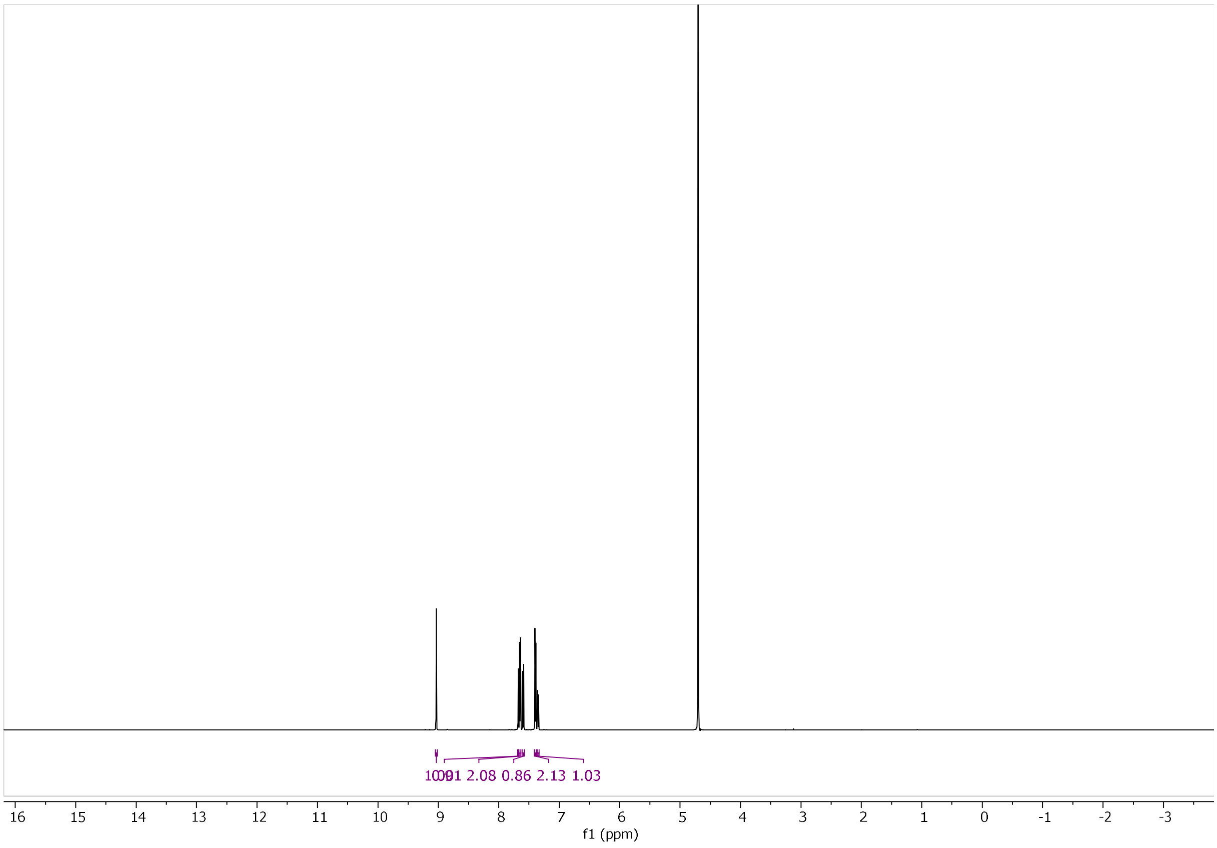

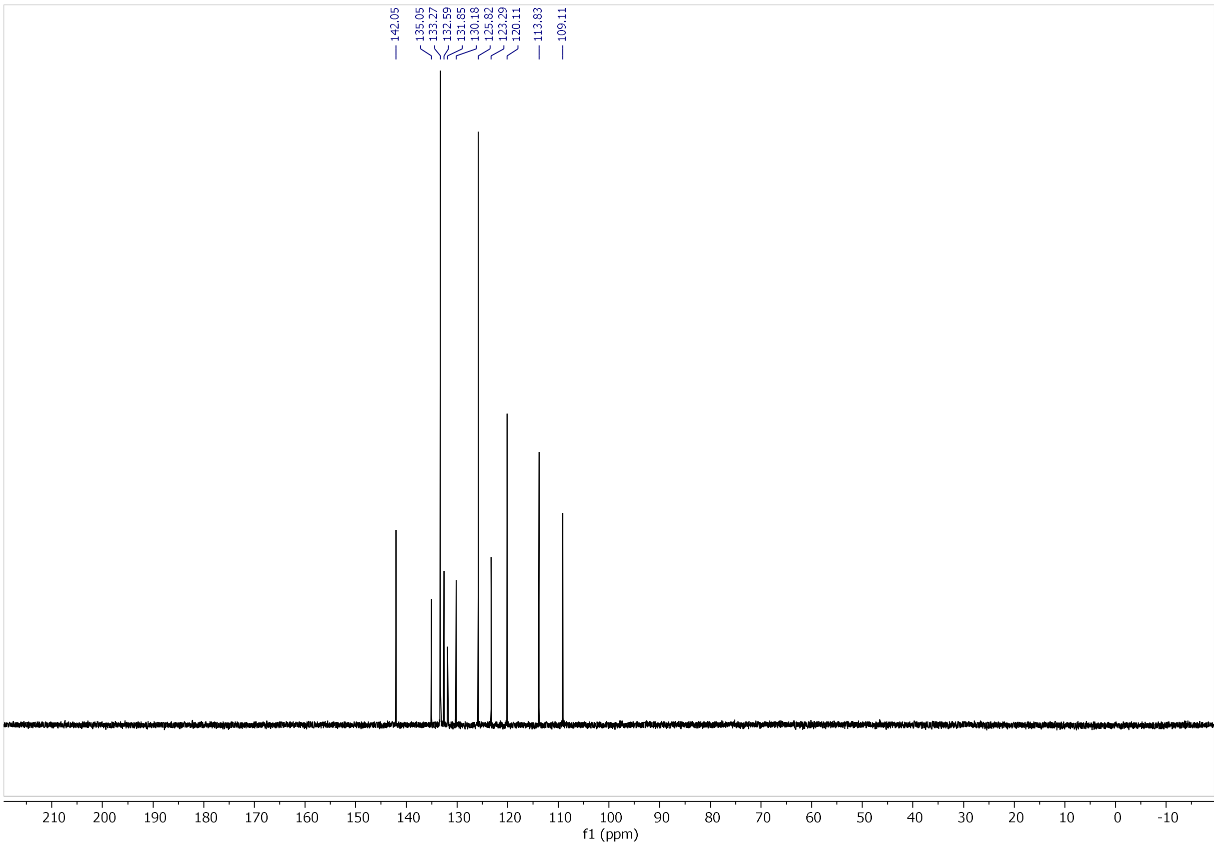

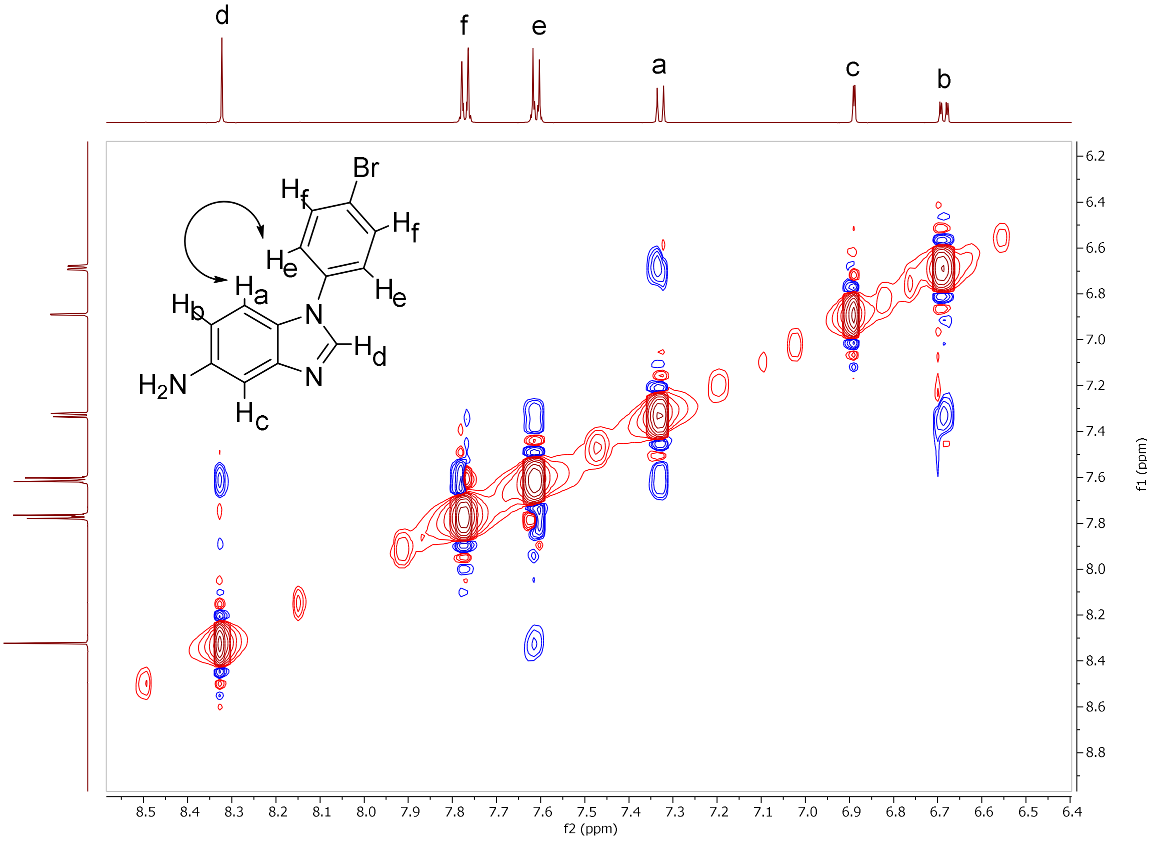

^{1}H-^{1}H ROESY of compound **2e** (freebase dissolved in DMSO-*d*6)
key correlation shown with a curved arrow

1-(4-bromophenyl)-1*H*-benzo[*d*]imidazol-6-amine (2f)

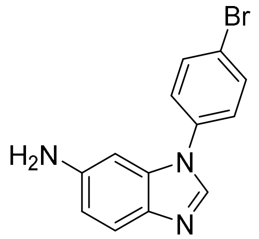

The title compound was obtained from separation of **2e** and washing with MeOH in analogous way (faster eluting, 2.63 g, 8% as HCl salt).

^{1}H NMR (600 MHz, D_2O) δ 9.48 (d, *J* = 1.0 Hz, 1H), 8.00 – 7.97 (m, 1H), 7.85 – 7.81 (m, 2H), 7.69 – 7.67 (m, 1H), 7.60 – 7.54 (m, 3H). ^{13}C NMR (151 MHz, D_2O) δ 142.1, 133.6, 133.0, 132.0 130.9, 130.8, 126.7, 124.3, 121.8, 117.2, 107.4.

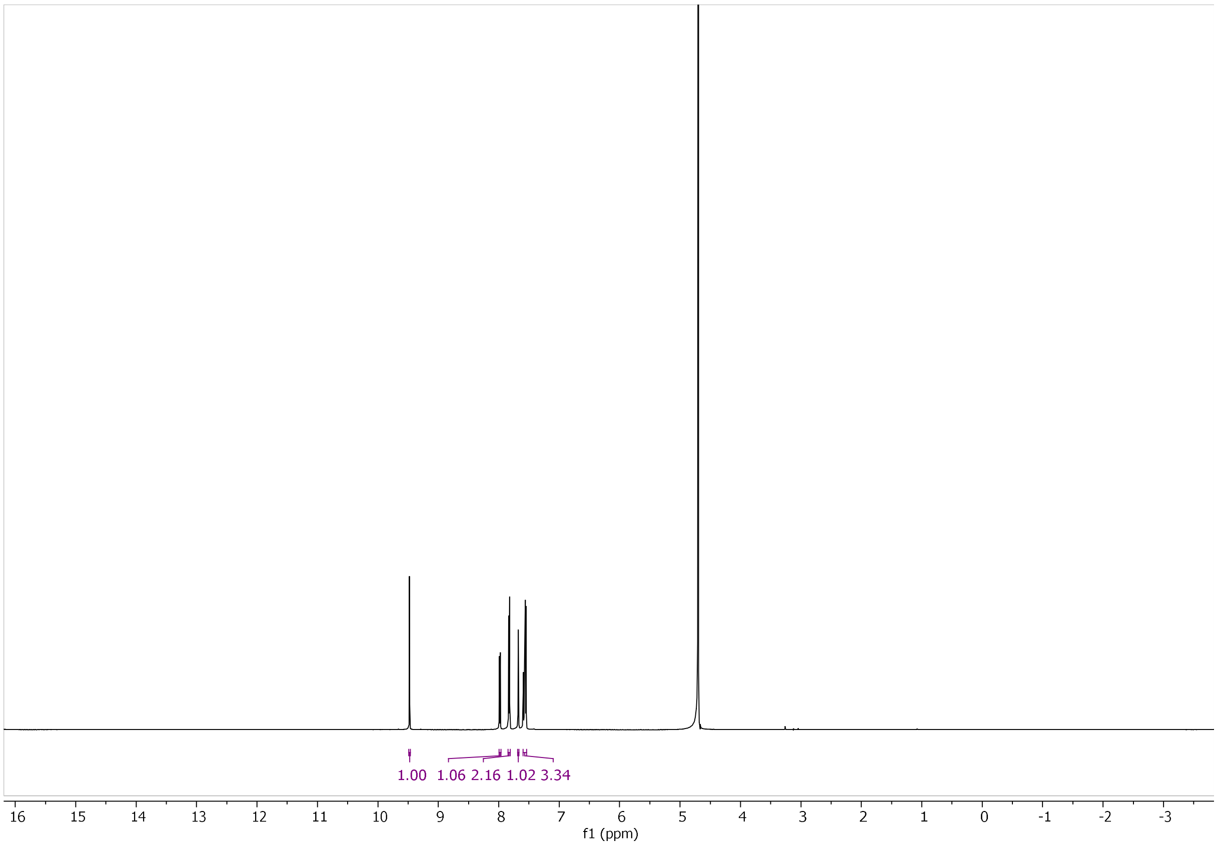

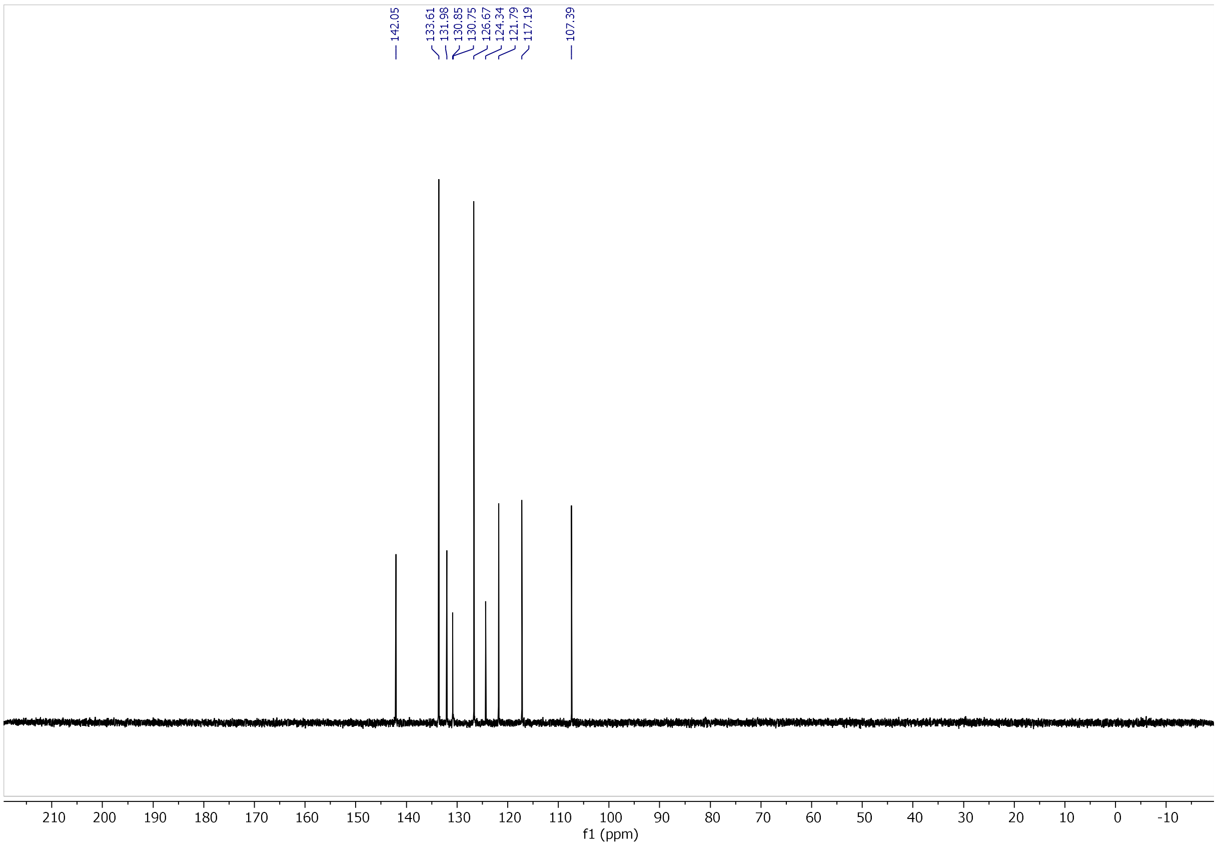

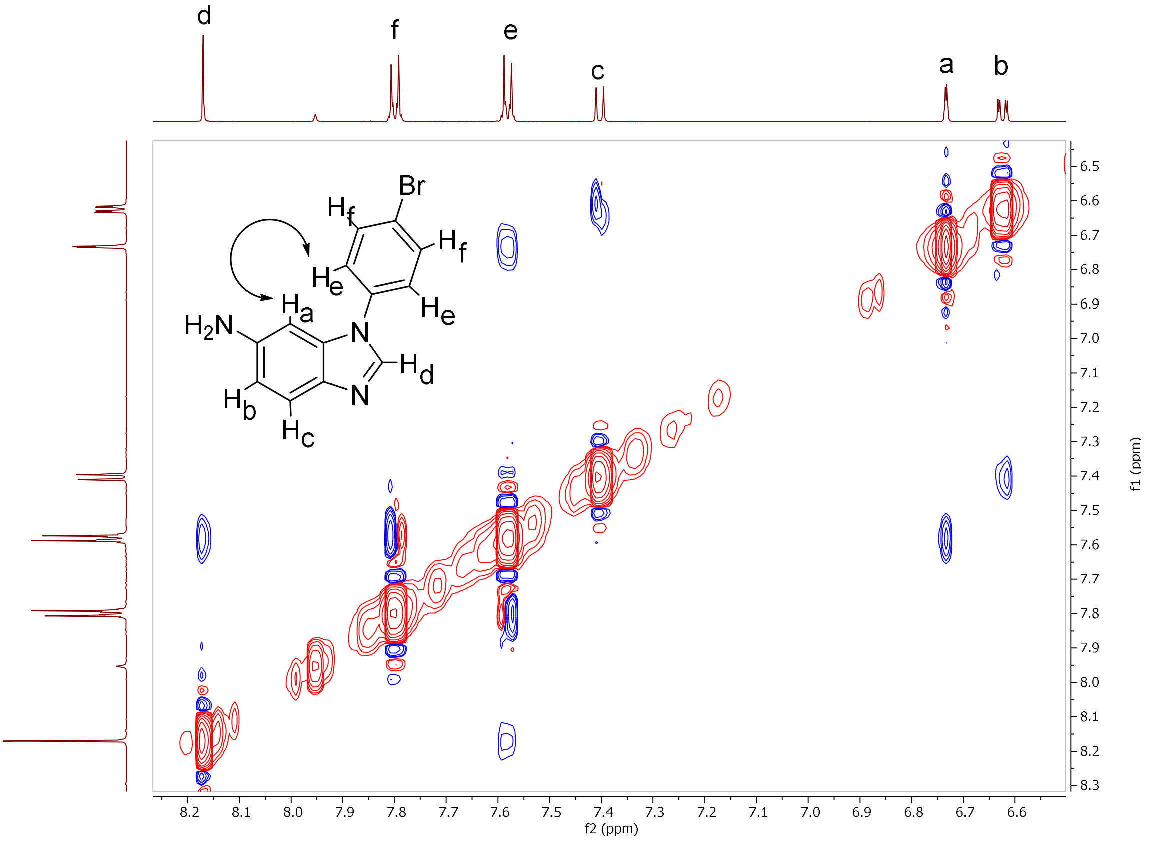

[1]H-^{1}H ROESY of compound **2f** (freebase dissolved in DMSO-*d6*)
key correlation shown with a curved arrow

tert-butyl (1-(4-bromophenyl)-1_H_-benzo[_d_]imidazol-5-yl)carbamate (2ea)

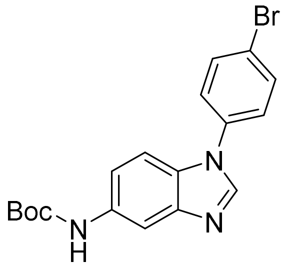

A flame-dried flask was evacuated/backfilled with argon three times and charged with freebase **2e** (490 mg, 1.7 mmol), Boc_2O (371 mg, 1.7 mmol) and 17 mL of _t_-BuOH. Reaction was stirred at 40 °C over a weekend. It was then concentrated to dryness and mixed with Celite. The product was purified by column chromatography eluting with heptane/EtOAc (5:95 → 0:100) to obtain the title compound as a yellow solid (487 mg, 74%). Product can be recrystallized from EtOAc.

^{1}H NMR (600 MHz, DMSO-d_6) δ 9.37 (s, 1H), 8.49 (s, 1H), 7.91 (s, 1H), 7.83 – 7.78 (m, 2H), 7.67 – 7.63 (m, 2H), 7.52 (d, _J_ = 8.8 Hz, 1H), 7.41 (d, _J_ = 8.8 Hz, 1H), 1.50 (s, 9H). ^{13}C NMR (151 MHz, DMSO-d_6) δ 152.9, 144.0, 143.3, 135.3, 135.0, 132.8, 128.3, 125.2, 119.9, 115.6, 110.3, 108.8, 78.8, 28.1.

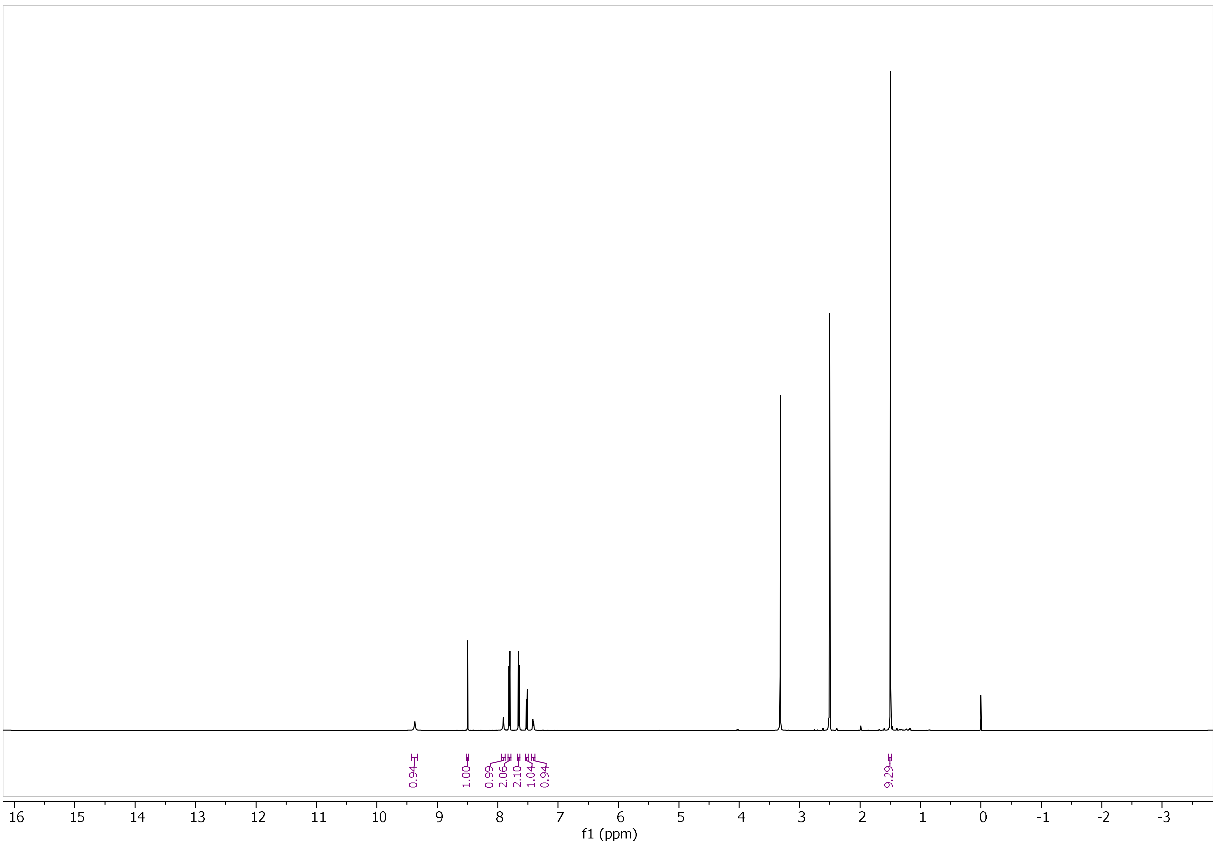

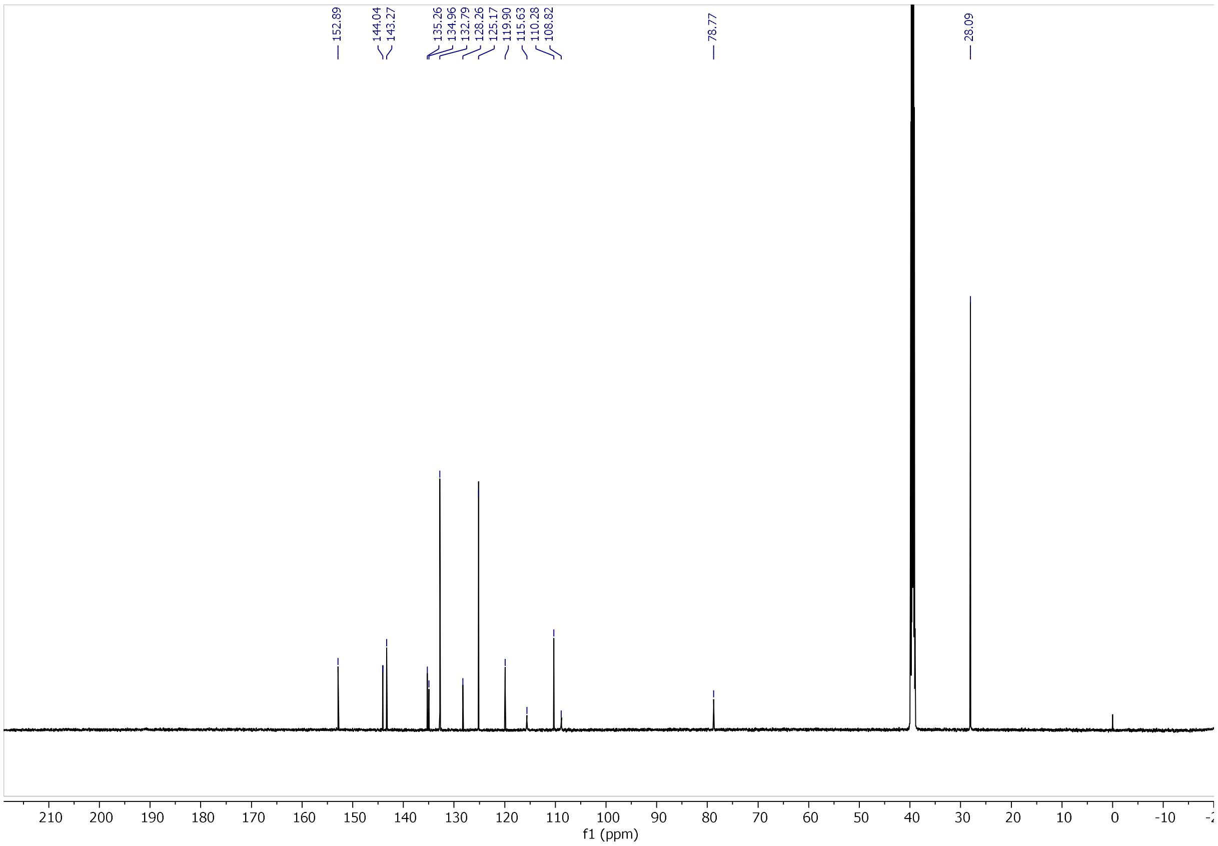
152.89
144.04
143.27
135.26
134.96
132.79
128.26
125.17
119.90
115.63
110.28
108.82
78.77
28.09
210 200 190 180 170 160 150 140 130 120 110 100 90 80 70 60 50 40 30 20 10 0 -10
f1 (ppm)

tert-butyl (1-(4-bromophenyl)-1_H_-benzo[_d_]imidazol-6-yl)carbamate (2fa)

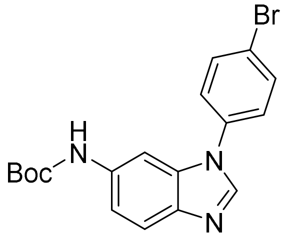

A flame-dried flask was evacuated/backfilled with argon three times and charged with **2f** (649 mg, 2 mmol), Boc_2O (437 mg, 2 mmol), TEA (0.42 mL, 3 mmol) and 20 mL of *t*-BuOH. Reaction was stirred at 40 °C for 5 hours and then stripped of solvent. Remaining residue was partitioned between 10 mL of water and 30 mL of EtOAc. Aqueous was further extracted with 3×30 mL of EtOAc. Combined organics were dried with anhydrous $MgSO_4$, and the removal of solvent afforded the title compound as a light brown solid (696 mg, 90%).

^{1}H NMR (600 MHz, DMSO-d_6) δ 9.46 (s, 1H), 8.41 (s, 1H), 7.93 (s, 1H), 7.87 – 7.84 (m, 2H), 7.65 – 7.60 (m, 3H), 7.25 (dd, *J* = 8.7, 2.0 Hz, 1H), 1.47 (s, 9H). ^{13}C NMR (151 MHz, DMSO-d_6) δ 152.8, 142.5, 139.0, 136.0, 135.2, 133.1, 132.9, 125.8, 120.3, 119.7, 114.6, 99.1, 78.9, 28.0.

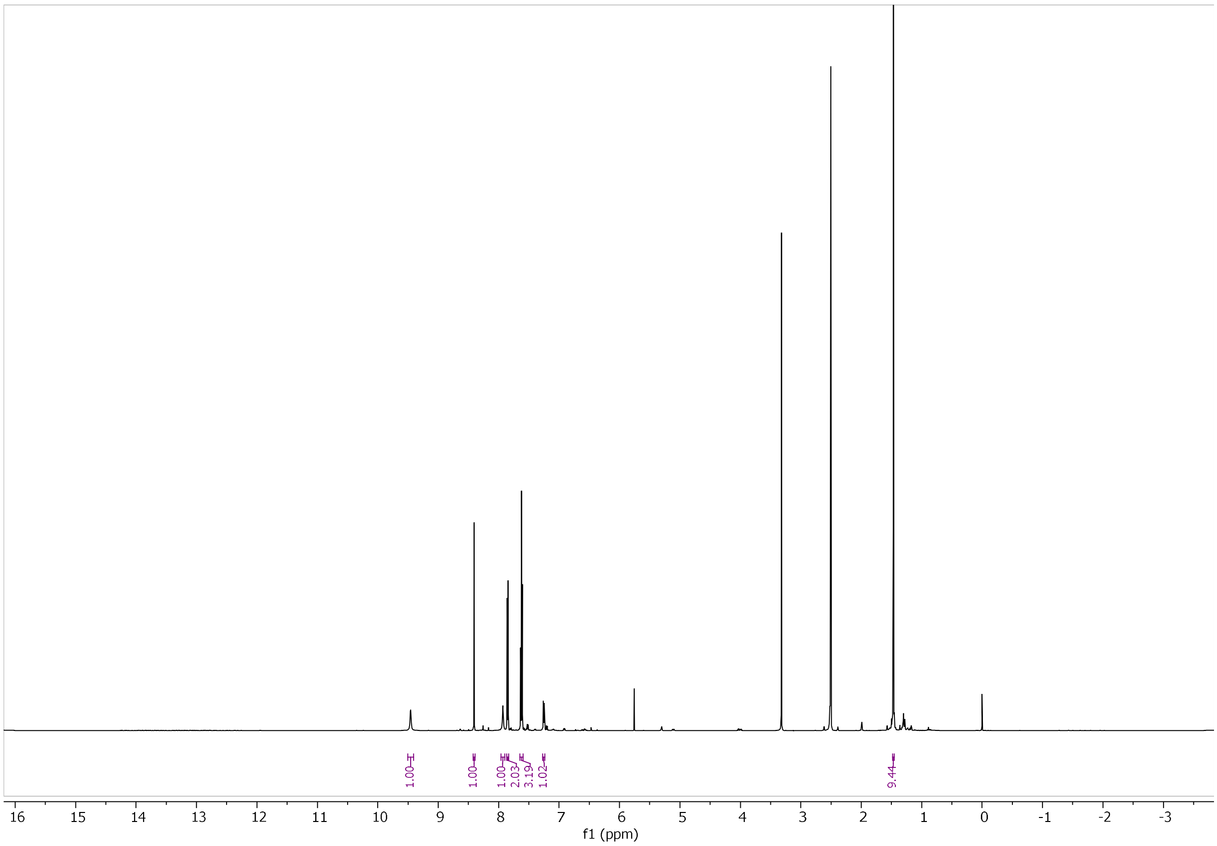

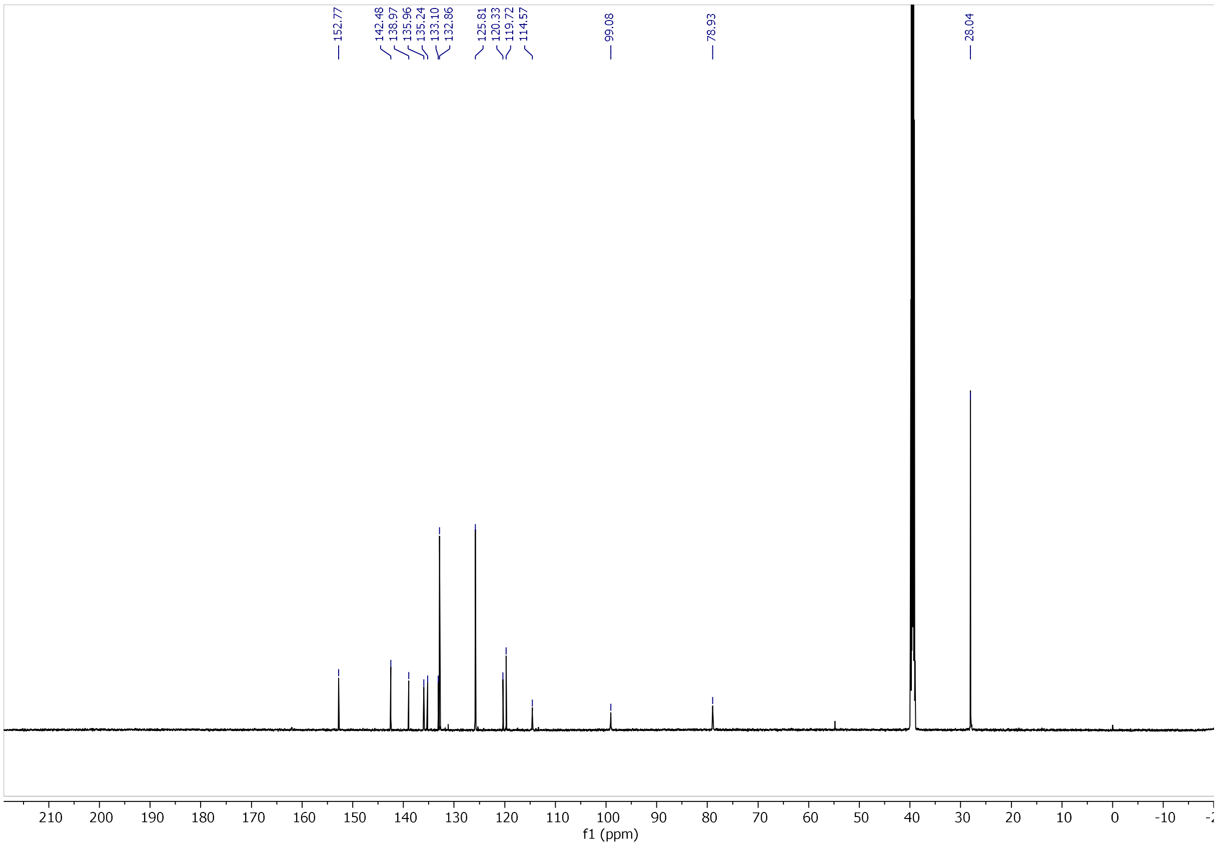

152.77
142.48
138.97
135.96
135.24
133.10
132.86
125.81
120.33
119.72
114.57
99.08
78.93
28.04
210 200 190 180 170 160 150 140 130 120 110 100 90 80 70 60 50 40 30 20 10 0 -10
f1 (ppm)

1-(4-(pyridin-4-ylamino)phenyl)-1*H*-benzo[*d*]imidazol-5-amine (15)

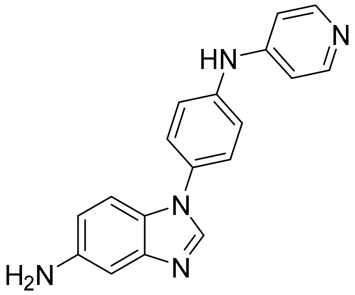

A flame-dried vial was charged with **2ea** (116 mg, 0.3 mmol), 4-aminopyridine (34 mg, 0.36 mmol), tBuXPhos Pd G1 (10 mg, 0.015 mmol), tBuXPhos (6 mg, 0.015 mmol) and sodium t-butoxide (58 mg, 0.6 mmol). It was sealed and evacuated/backfilled with argon three times. A total of 3 mL of anhydrous t-BuOH was added and the reaction was stirred at 40 °C overnight. Solvent was removed and the residue was partitioned between 5 mL of water and 10 mL of EtOAc. Aqueous was extracted with 2 × 10 mL of EtOAc. Organics were dried with anhydrous Na_2SO_4 and solvent was removed. Crude was purified by column chromatography on silica eluting with MeOH/EtOAc (0:100 → 5:95) + 2% TEA to obtain Boc-protected product (64 mg, 53%), which was deprotected according to the general procedure affording the title compound as a light brown solid (40 mg, 50% over two steps).

^{1}H NMR (400 MHz, DMSO-*d*$_6$) δ 9.86 (s, 1H), 8.34 – 8.24 (m, 3H), 7.71 – 7.63 (m, 2H), 7.51 – 7.44 (m, 2H), 7.33 (d, *J* = 8.6 Hz, 1H), 7.13 – 7.05 (m, 2H), 6.89 (d, *J* = 2.0 Hz, 1H), 6.69 (dd, *J* = 8.6, 2.1 Hz, 1H), 3.46 (s, 2H). ^{13}C NMR (101 MHz, DMSO-*d*$_6$) δ 153.64, 145.73, 145.63, 145.18, 142.61, 138.07, 133.04, 125.89, 124.66, 123.34, 113.39, 110.99, 109.63, 103.35. HRMS (MALDI): *m*/*z* calculated for [M+H]$^+$ 302.14002, found 302.14000.

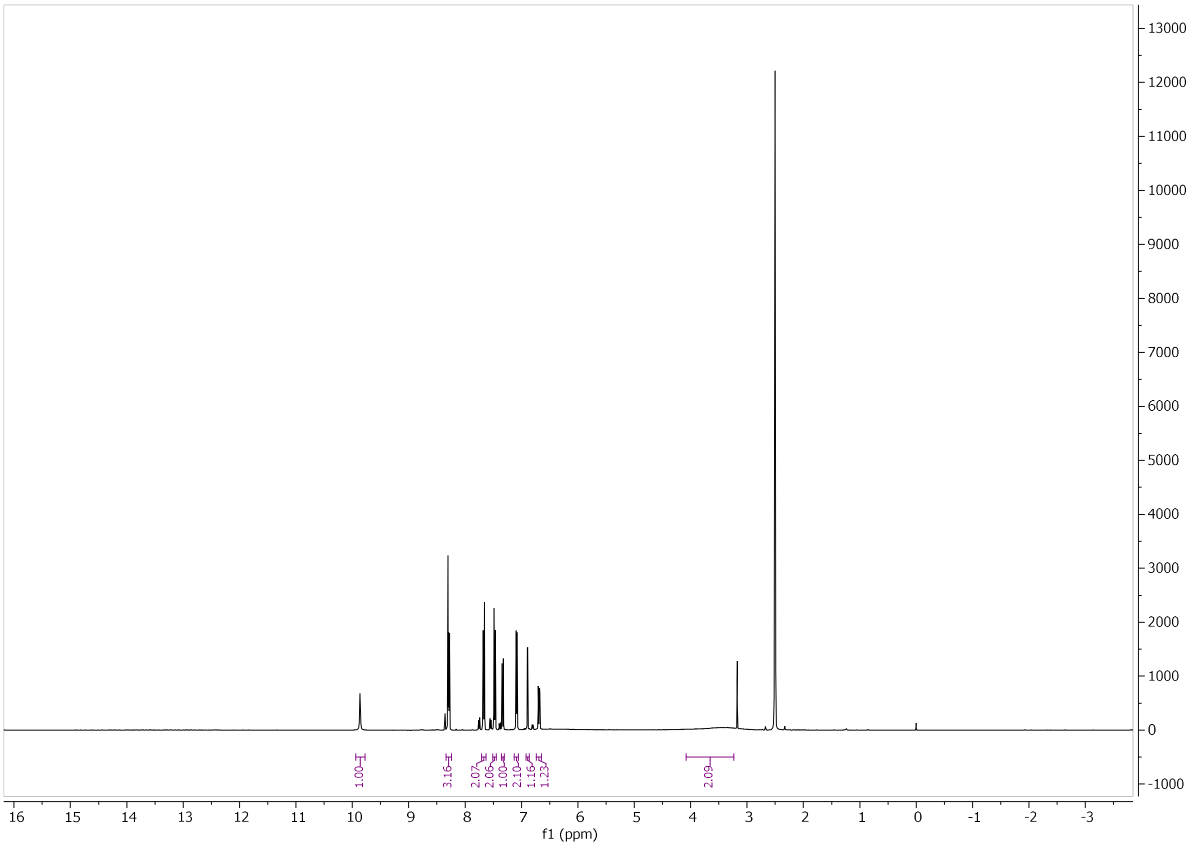

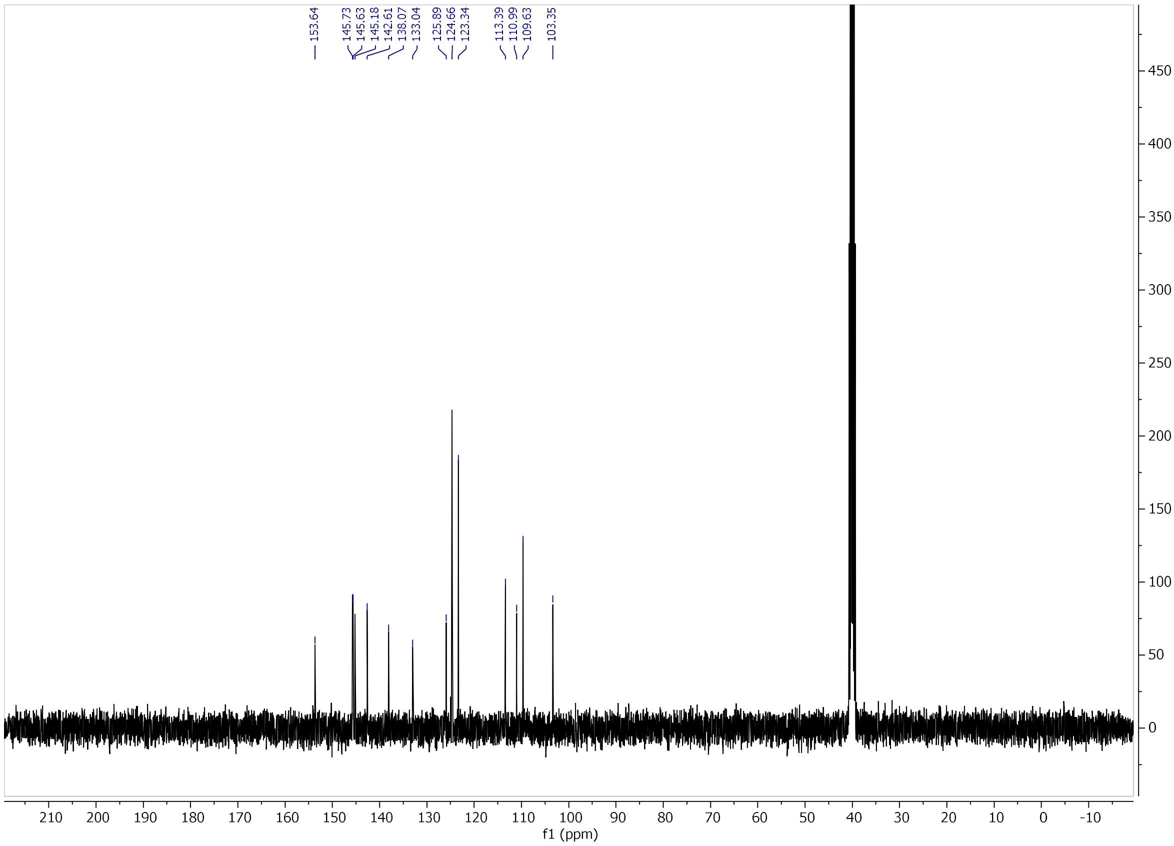
153.64
145.73
145.63
145.18
142.61
138.07
133.04
125.89
124.66
123.34
113.39
110.99
109.63
103.35
f1 (ppm)

1-(4-(pyridin-4-ylamino)phenyl)-1*H*-benzo[*d*]imidazol-6-amine (16)

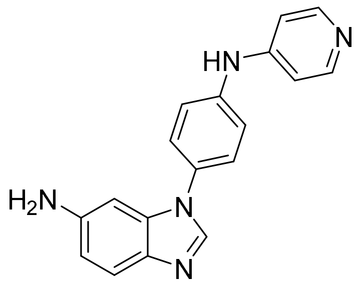

A flame-dried vial was charged with **2fa** (97 mg, 0.25 mmol), 4-aminopyridine (28 mg, 0.3 mmol), tBuXPhos Pd G1 (9 mg, 0.013 mmol), tBuXPhos (5 mg, 0.013 mmol) and sodium t-butoxide (48 mg, 0.5 mmol). It was sealed and evacuated/backfilled with argon three times. A total of 2.5 mL of anhydrous t-BuOH was added and the reaction was stirred at 40 °C overnight. Solvent was removed and the residue was partitioned between 5 mL of water and 10 mL of EtOAc. Aqueous was extracted with 2 × 10 mL of EtOAc. Combined organics were dried with anhydrous Na_2SO_4 and solvent was removed in vacuo. Crude was purified by column chromatography on silica eluting MeOH/EtOAc (0:100 → 5:95) + 2% TEA to obtain Boc-protected product (40 mg, 31%). Deprotection according to the general procedure gave the title compound as a light brown solid (22 mg, 23% over two steps).

^{1}H NMR (600 MHz, DMSO-*d*$_6$) δ 9.17 (s, 1H), 8.29 – 8.21 (m, 2H), 8.12 (s, 1H), 7.56 (d, *J* = 8.7 Hz, 2H), 7.45 – 7.37 (m, 3H), 7.04 – 6.96 (m, 2H), 6.72 (d, *J* = 2.0 Hz, 1H), 6.61 (dd, *J* = 8.6, 2.0 Hz, 1H), 5.33 (s, 2H). ^{13}C NMR (151 MHz, DMSO-*d*$_6$) δ 150.32, 149.13, 145.82, 140.10, 139.26, 135.40, 134.55, 131.01, 124.67, 121.09, 119.80, 111.65, 109.26, 93.26. HRMS (MALDI): *m*/*z* calculated for $[M+H]^+$ 302.14002, found 302.14001.

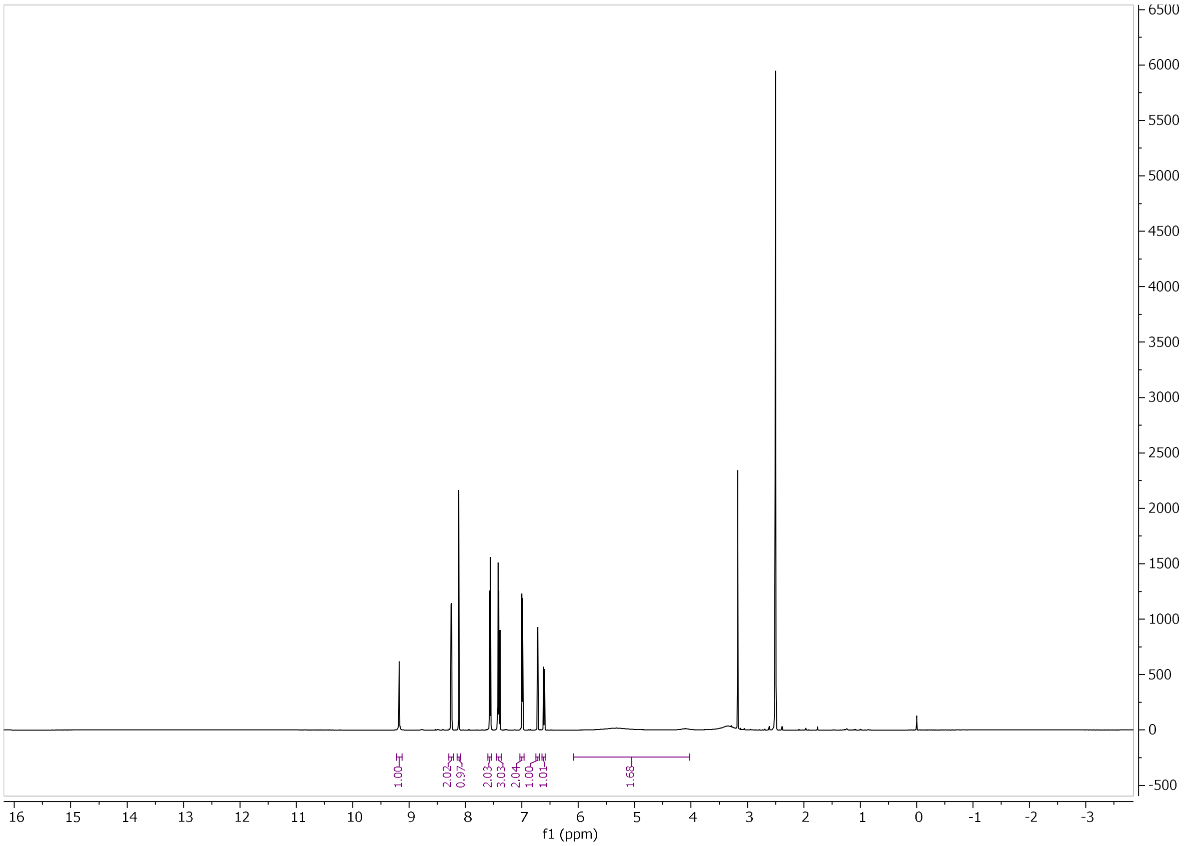

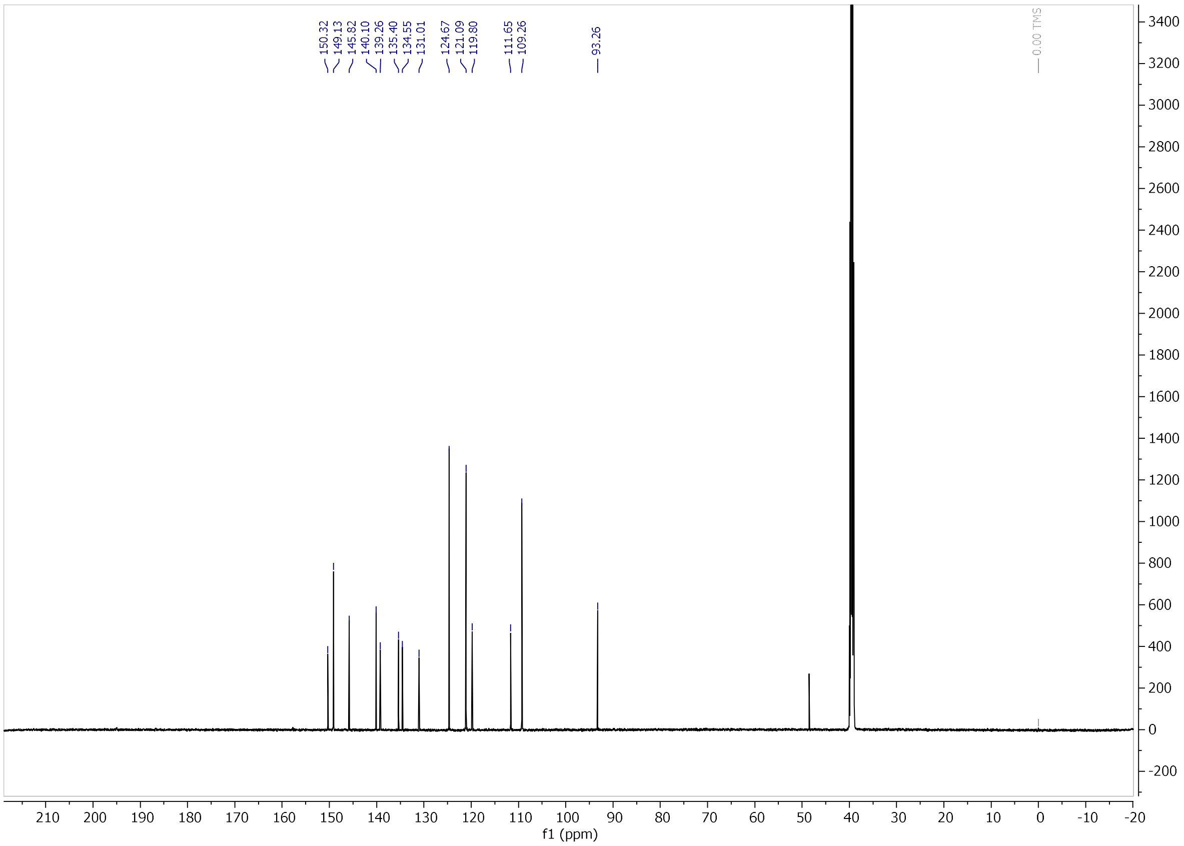

150.32
149.13
145.82
140.10
139.26
135.40
134.55
131.01
124.67
121.09
119.80
111.65
109.26
93.26
0.00 TMS
f1 (ppm)

Scheme S5 Synthetic Details

1-(4-nitrophenyl)-1H-indole (4c)

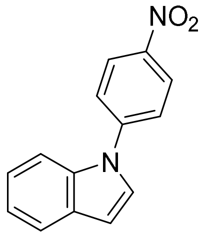

A round bottom flask was charged with indole (5.86 g, 50 mmol), 1-fluoro-4-nitrobenzene (7.76 g, 55 mmol), potassium phosphate (21.23 g, 100 mmol) and DMF (200 mL). The reaction was stirred at 160 °C under an argon balloon for 4 hours. After cooling down, the reaction was poured into 750 mL of water and left overnight. Formed precipitate was filtered and washed with water. It was then washed with boiling ethanol (100 mL) and after cooling down it was filtered and washed again with ethanol. The product was obtained as a dark yellow solid (11.05 g, 93%).

^{1}H NMR (300 MHz, $CDCl_3$) δ 8.46 – 8.37 (m, 2H), 7.77 – 7.63 (m, 4H), 7.40 (d, *J* = 3.4 Hz, 1H), 7.37 – 7.22 (m, 2H), 6.80 (d, *J* = 3.4 Hz, 1H). ^{13}C NMR (75 MHz, $CDCl_3$) δ 145.2, 145.0, 135.2, 130.1, 127.1, 125.5, 123.4, 123.3, 121.7, 121.6, 110.5, 106.2.

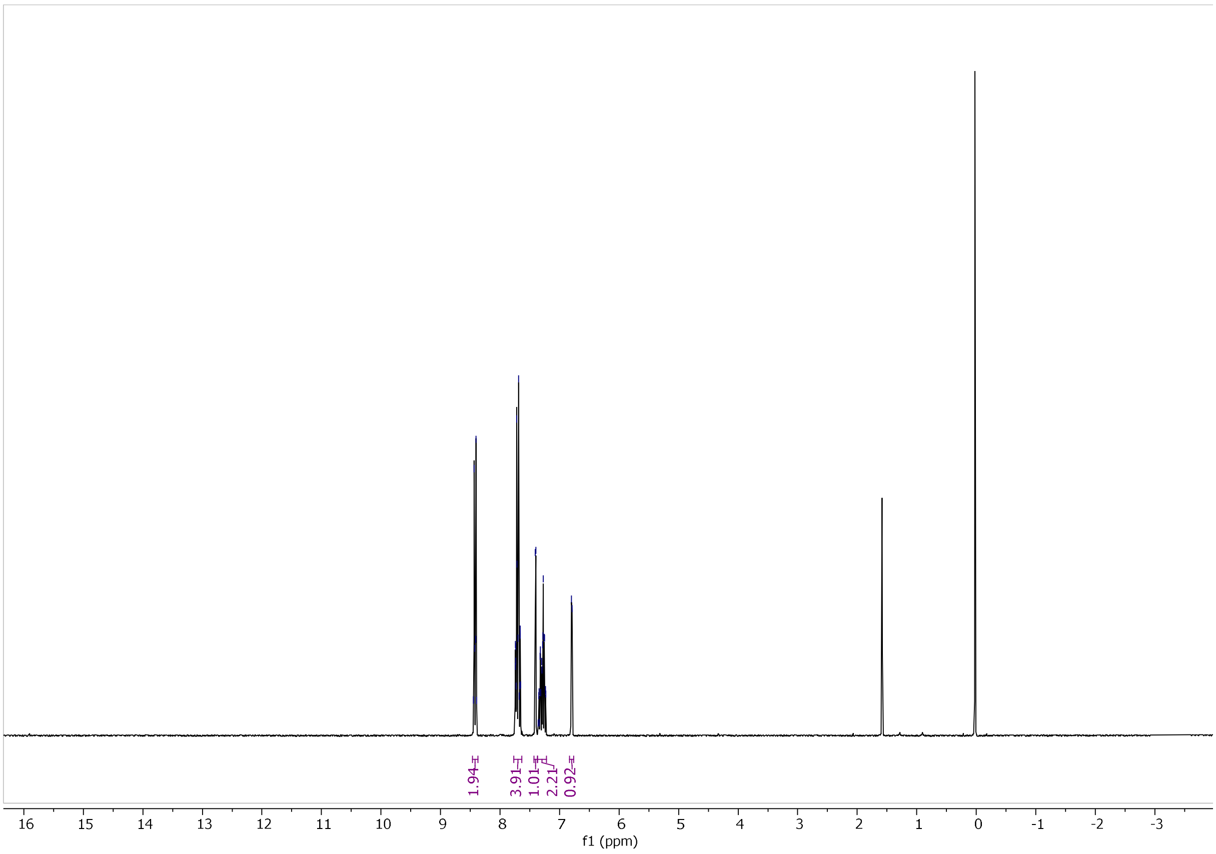

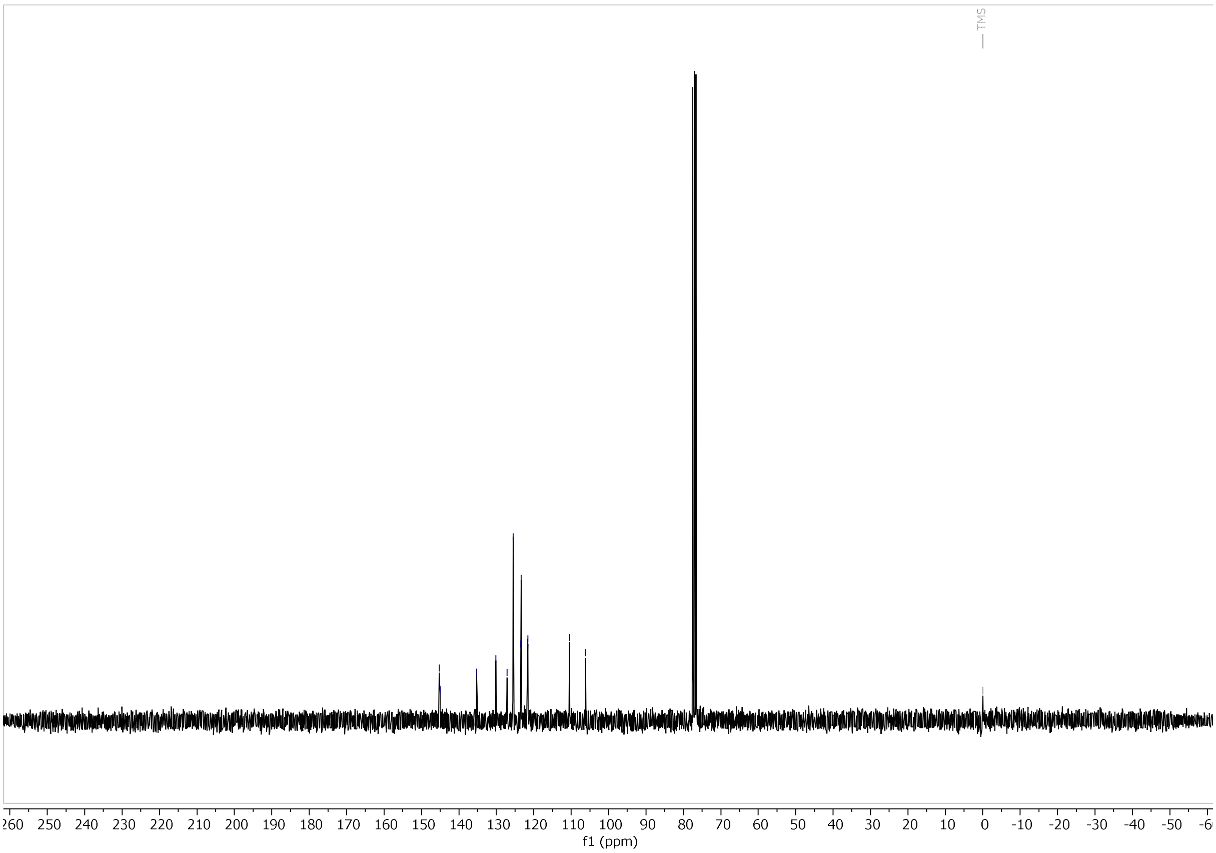
TMS
260 250 240 230 220 210 200 190 180 170 160 150 140 130 120 110 100 90 80 70 60 50 40 30 20 10 0 -10 -20 -30 -40 -50
f1 (ppm)

1-(3-methyl-4-nitrophenyl)-1H-indole (4d)

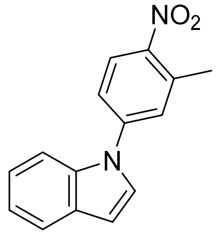

A round bottom flask was charged with indole (5.86 g, 50 mmol), 4-fluoro-2-methyl-1-nitrobenzene (8.53 g, 55 mmol), potassium phosphate (21.23 g, 100 mL) and DMF (200 mL). The reaction was stirred for 4 hours at 160 °C and then poured into 750 mL of water and left overnight. Precipitated product was filtered and washed with water. Purification by DCVC on silica gel eluting with 20% EtOAc in heptane and subsequent crystallization from EtOH gave the title product as a bright yellow solid (2.32 g, 18%).

^{1}H NMR (300 MHz, $CDCl_3$) δ 8.21 – 8.13 (m, 1H), 7.68 (ddd, *J* = 7.5, 1.5, 0.8 Hz, 1H), 7.65 – 7.57 (m, 1H), 7.49 – 7.42 (m, 2H), 7.33 (d, *J* = 3.4 Hz, 1H), 7.31 – 7.16 (m, 2H), 6.73 (dd, *J* = 3.4, 0.8 Hz, 1H), 2.70 (s, 3H). ^{13}C NMR (75 MHz, $CDCl_3$) δ 146.1, 143.7, 136.4, 135.3, 130.0, 127.2, 126.9, 126.8, 123.3, 121.6, 121.4, 121.2, 110.5, 105.8, 21.2.

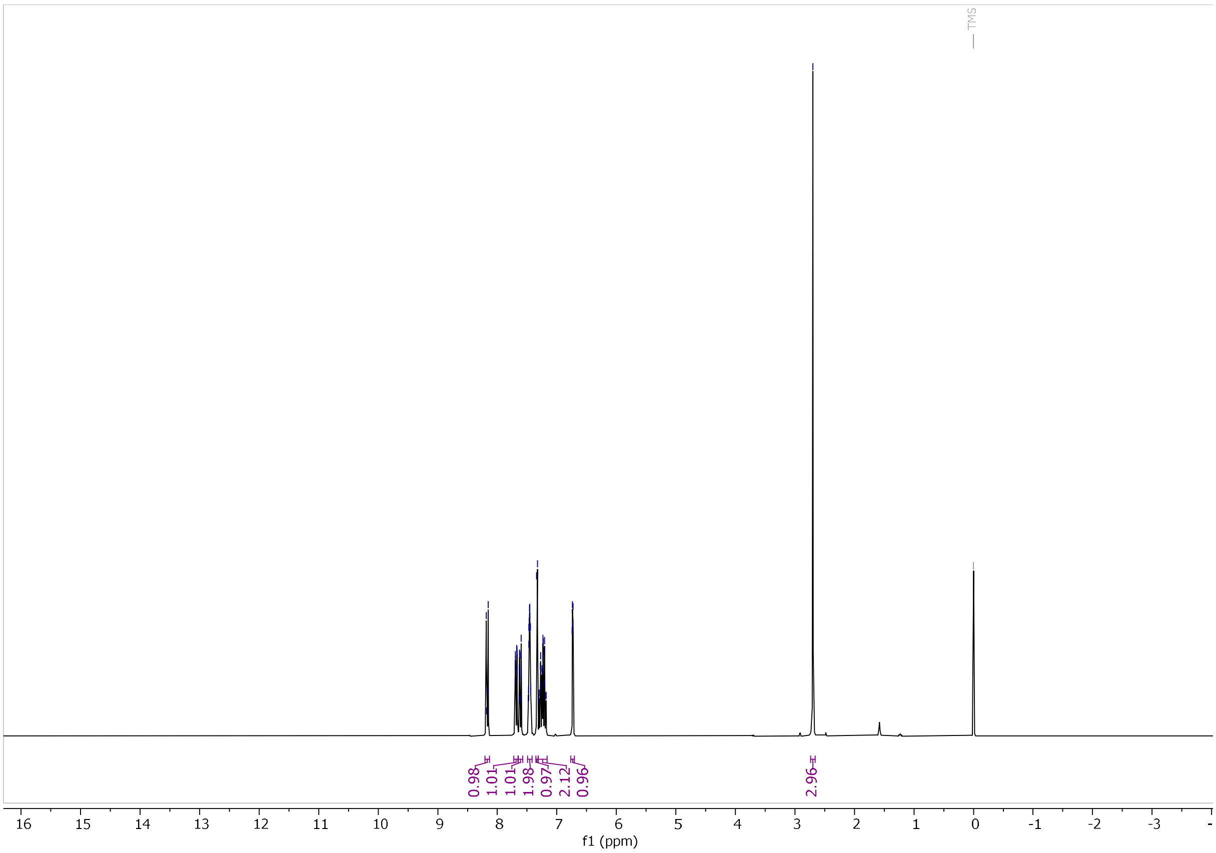

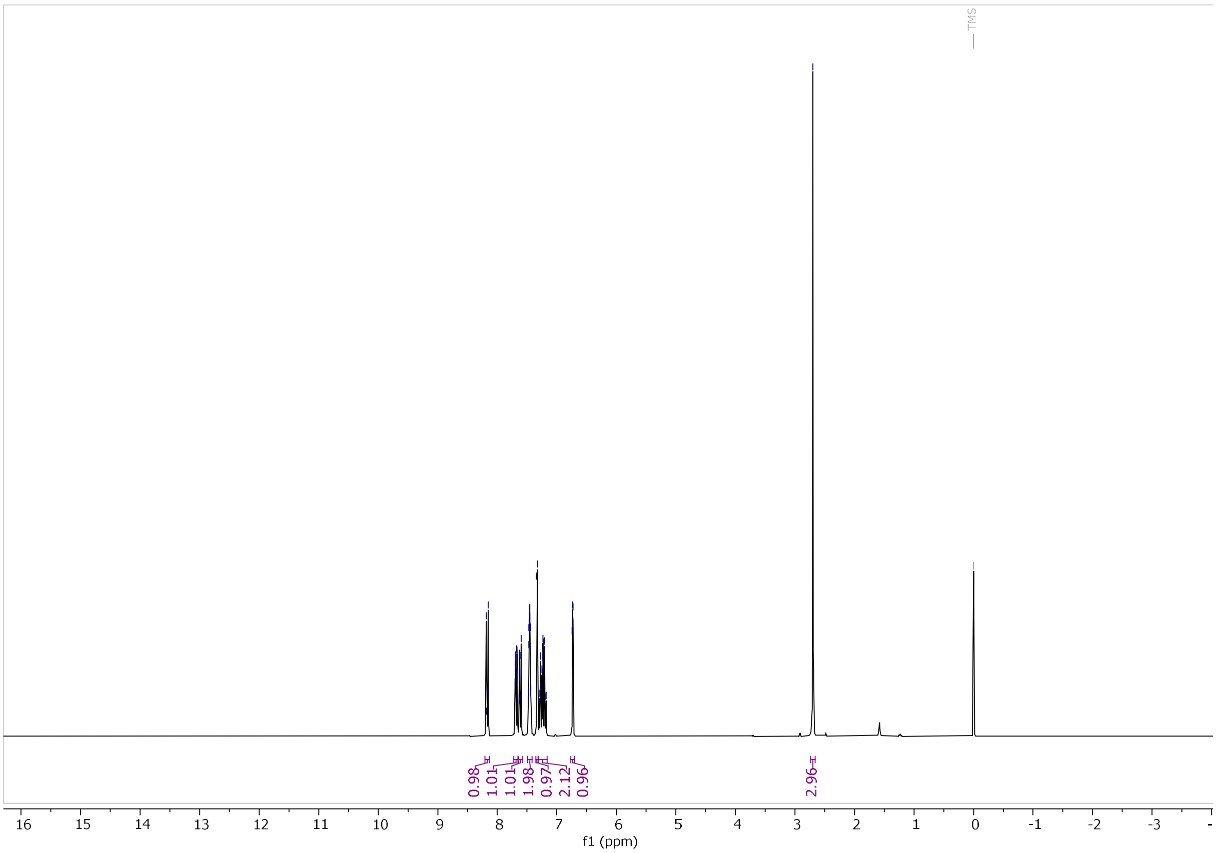

TMS
0.98
1.01
1.01
1.98
0.97
2.12
0.96
2.96
16 15 14 13 12 11 10 9 8 7 6 5 4 3 2 1 0 -1 -2 -3
f1 (ppm)

1-(2-methyl-4-nitrophenyl)-1H-indole (4e)

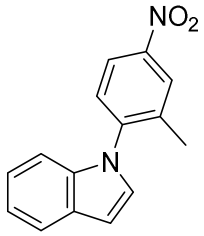

A round bottom flask was charged with indole (5.86 g, 50 mmol), 1-fluoro-2-methyl-4-nitrobenzene (8.53 g, 55 mmol), potassium phosphate (21.23 g, 100 mL) and DMF (200 mL). The reaction was stirred for 14 hours at 160 °C and then cooled down and poured into 750 mL of water. After standing for several hours, precipitate formed, which was filtered and washed with water. Purification by DCVC on silica gel eluting with 10% EtOAc in heptane and subsequent crystallization from EtOH gave the title product as a bright yellow solid (2.99 g, 24%).

^{1}H NMR (300 MHz, $CDCl_3$) δ 8.28 (d, *J* = 2.7 Hz, 1H), 8.22 – 8.13 (m, 1H), 7.75 – 7.67 (m, 1H), 7.48 (d, *J* = 8.6 Hz, 1H), 7.26 – 7.14 (m, 3H), 7.10 – 7.01 (m, 1H), 6.74 (dd, *J* = 3.3, 0.9 Hz, 1H), 2.23 (s, 3H). ^{13}C NMR (75 MHz, $CDCl_3$) δ 146.9, 144.0, 137.0, 136.5, 128.7, 128.7, 128.0, 126.6, 122.8, 122.2, 121.3, 120.8, 110.3, 104.3, 18.3.

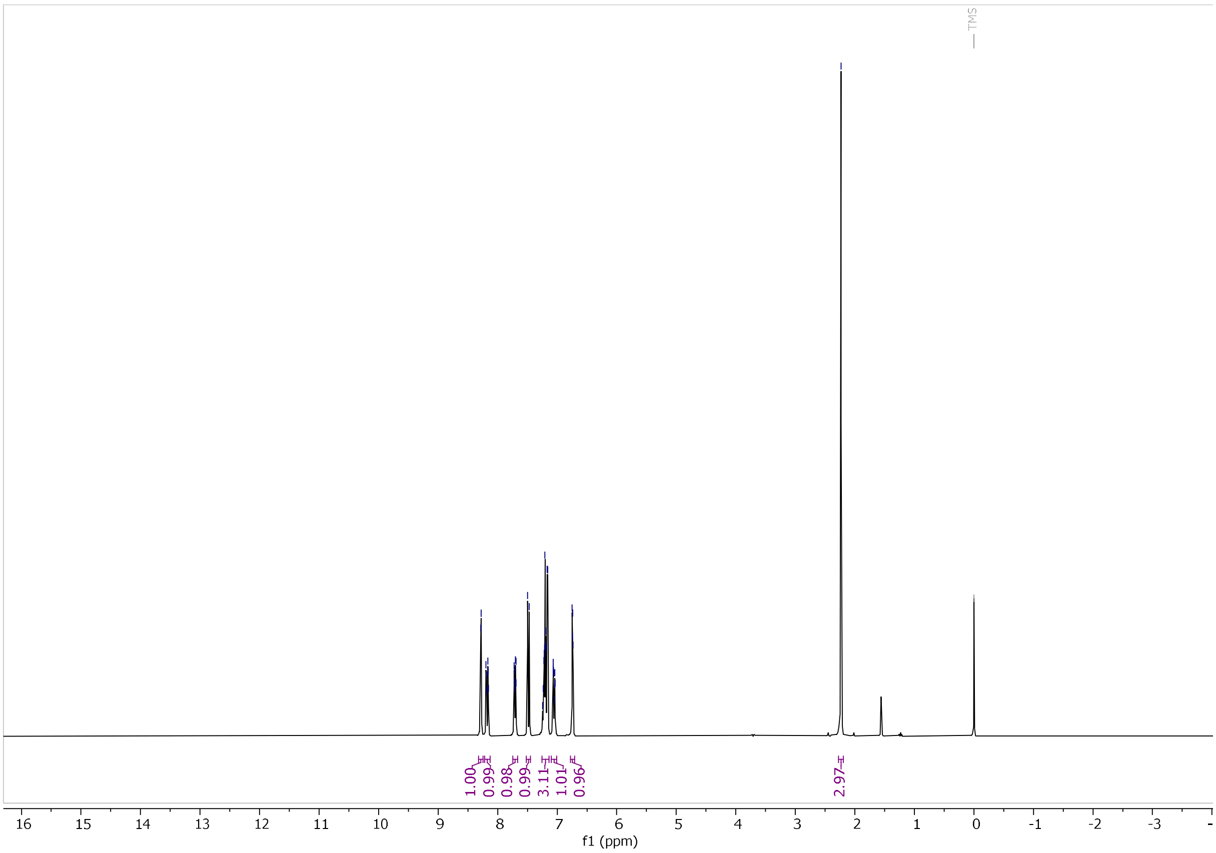

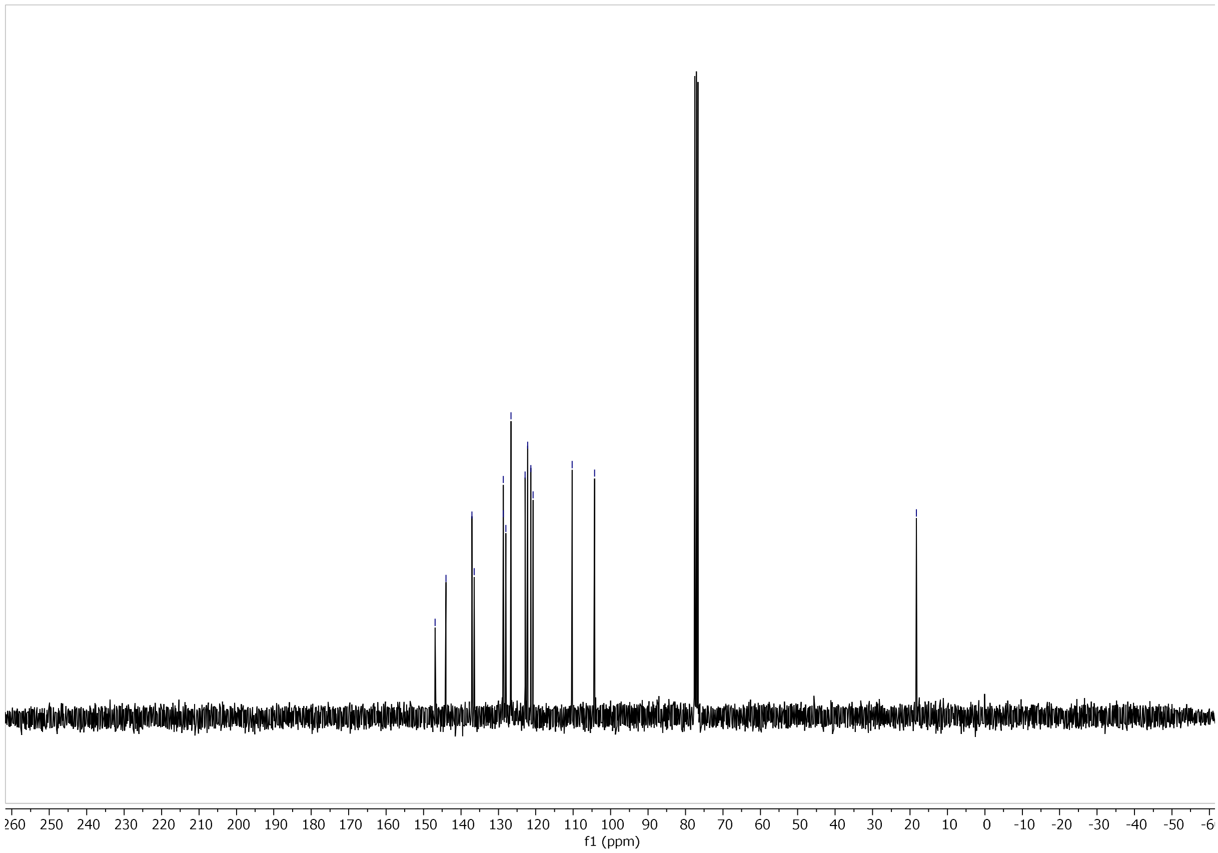
260 250 240 230 220 210 200 190 180 170 160 150 140 130 120 110 100 90 80 70 60 50 40 30 20 10 0 -10 -20 -30 -40 -50 -60
f1 (ppm)

4-(1H-indol-1-yl)aniline (5c)

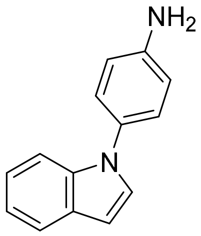

A two-neck round bottom flask was flushed with argon and charged with **4c** (953 mg, 4 mmol), Pd/C (10%, 213 mg, 5 mol%) and MeOH (50 mL). The reaction was hydrogenated at room temperature using a hydrogen-filled balloon for 24 hours. The catalyst was filtered over a plug of silica with subsequent washing with MeOH. Upon solvent removal, the product was obtained as green oil (755 mg, 91%).

^{1}H NMR (300 MHz, $CDCl_3$) δ 7.69 – 7.60 (m, 1H), 7.44 – 7.35 (m, 1H), 7.18 – 7.01 (m, 5H), 6.57 (d, *J* = 3.2 Hz, 1H), 6.49 – 6.40 (m, 2H), 3.33 (s, 2H). ^{13}C NMR (75 MHz, $CDCl_3$) δ 145.2, 136.3, 130.4, 128.7, 128.4, 125.7, 121.9, 120.9, 119.8, 115.4, 110.5, 102.4.

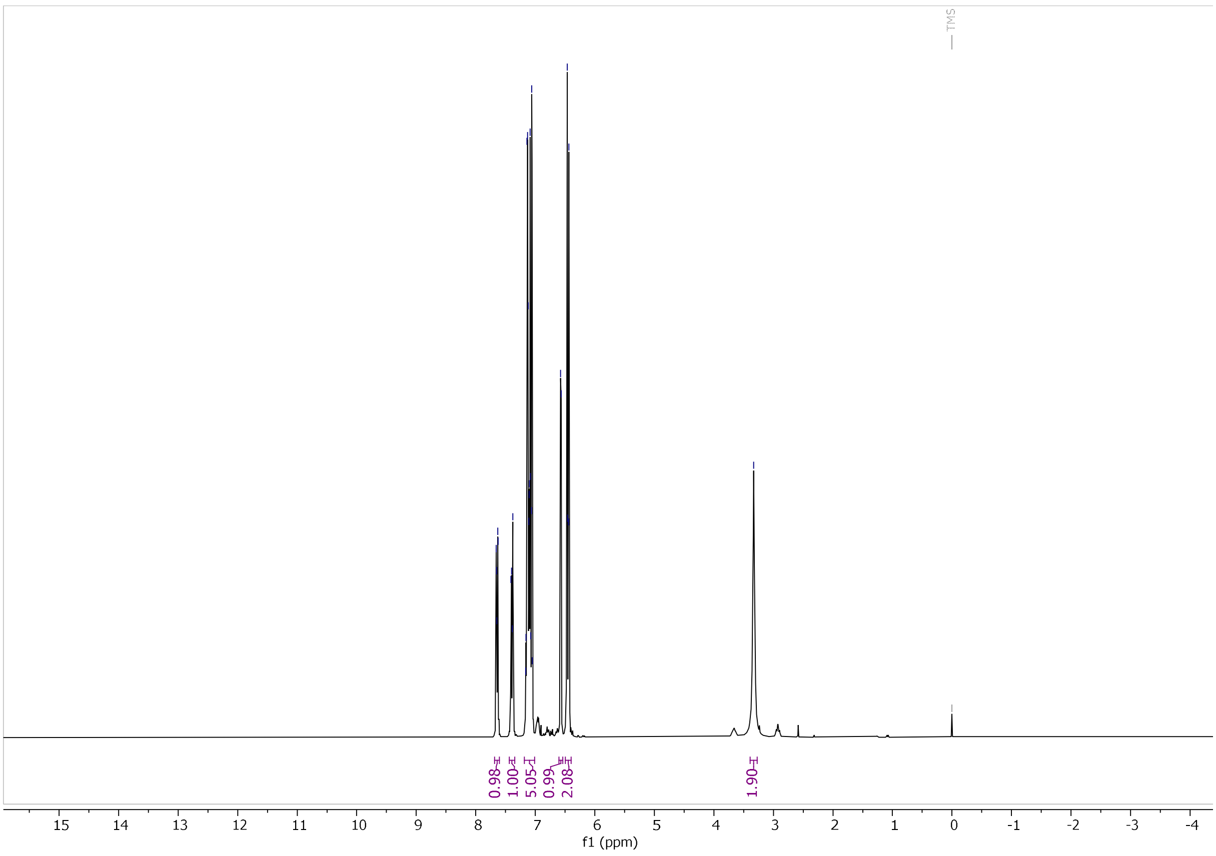

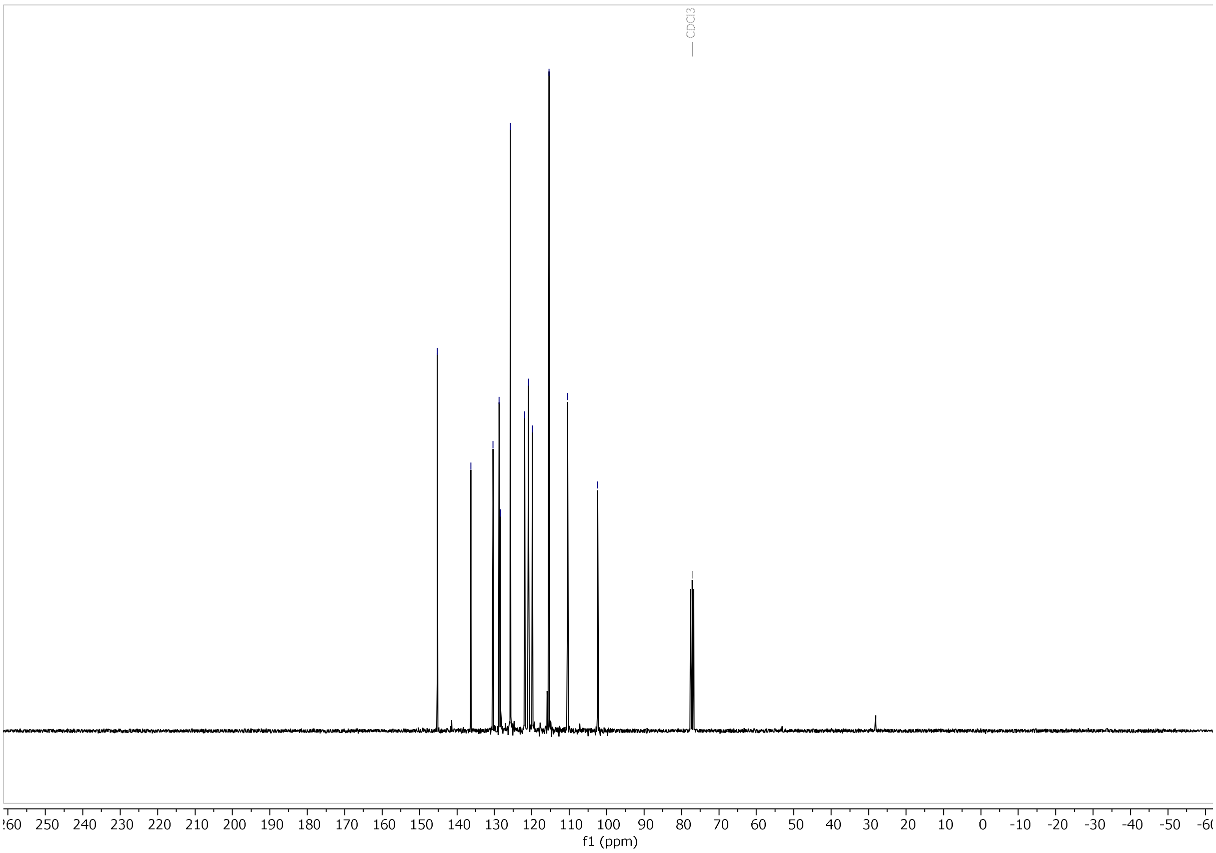
CDCl3
260 250 240 230 220 210 200 190 180 170 160 150 140 130 120 110 100 90 80 70 60 50 40 30 20 10 0 -10 -20 -30 -40 -50 -60
f1 (ppm)

4-(1H-indol-1-yl)-2-methylaniline (5d)

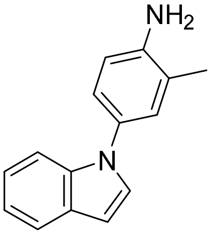

A two-neck round bottom flask was flushed with argon and charged with **4d** (1.01 g, 4 mmol), Pd/C (10%, 213 mg, 5 mol%) and MeOH (50 mL). The reaction was hydrogenated at room temperature using a hydrogen-filled balloon for 24 hours. The catalyst was filtered over a plug of silica with subsequent washing with MeOH. Upon solvent removal, the product was obtained as clear oil (837 mg, 94%).

^{1}H NMR (600 MHz, $CDCl_3$) δ 7.66 (dt, *J* = 7.8, 1.0 Hz, 1H), 7.46 – 7.43 (m, 1H), 7.24 (d, *J* = 3.2 Hz, 1H), 7.20 – 7.11 (m, 4H), 6.77 (d, *J* = 8.2 Hz, 1H), 6.61 (dd, *J* = 3.2, 0.8 Hz, 1H), 3.85 (s, 2H), 2.22 (s, 3H). ^{13}C NMR (151 MHz, $CDCl_3$) δ 143.1, 136.4, 131.0, 128.8, 128.5, 127.1, 123.6, 123.4, 121.9, 120.9, 119.8, 115.5, 110.5, 102.3, 17.5.

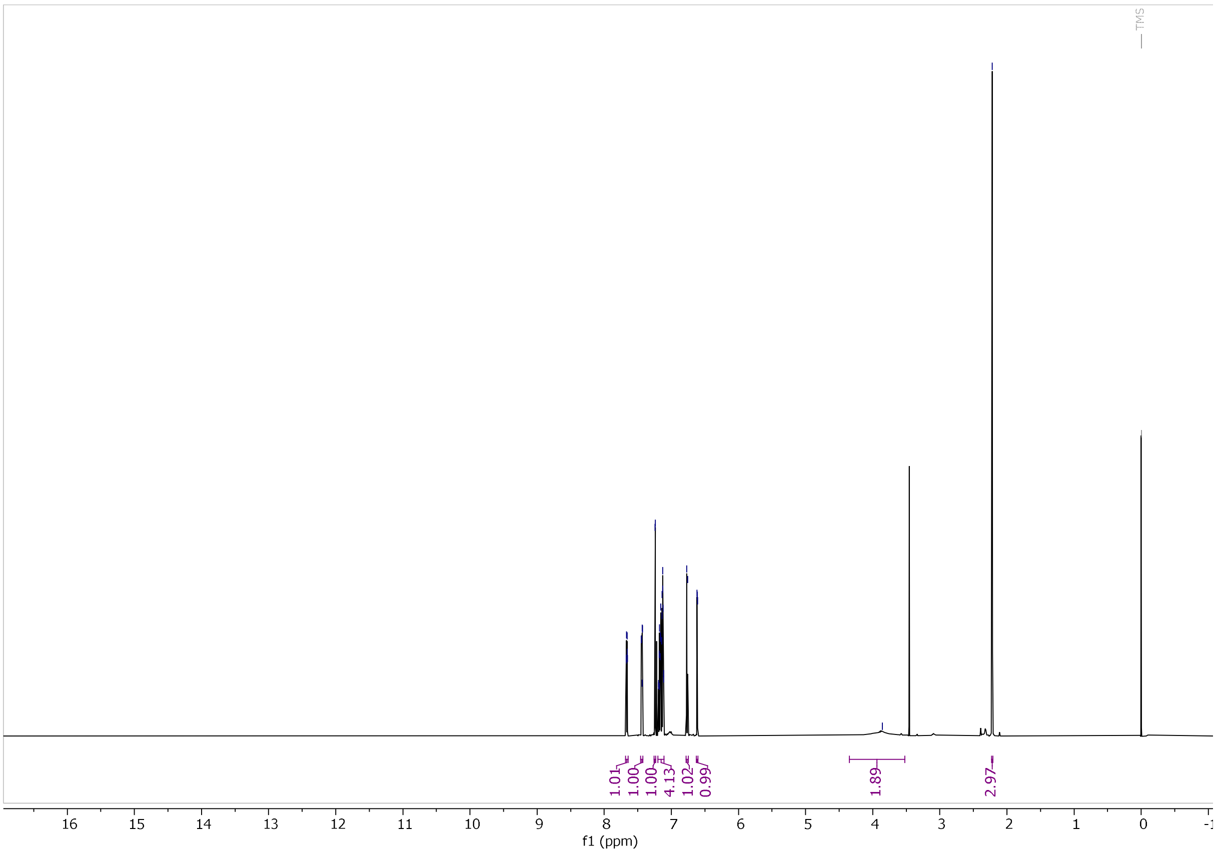

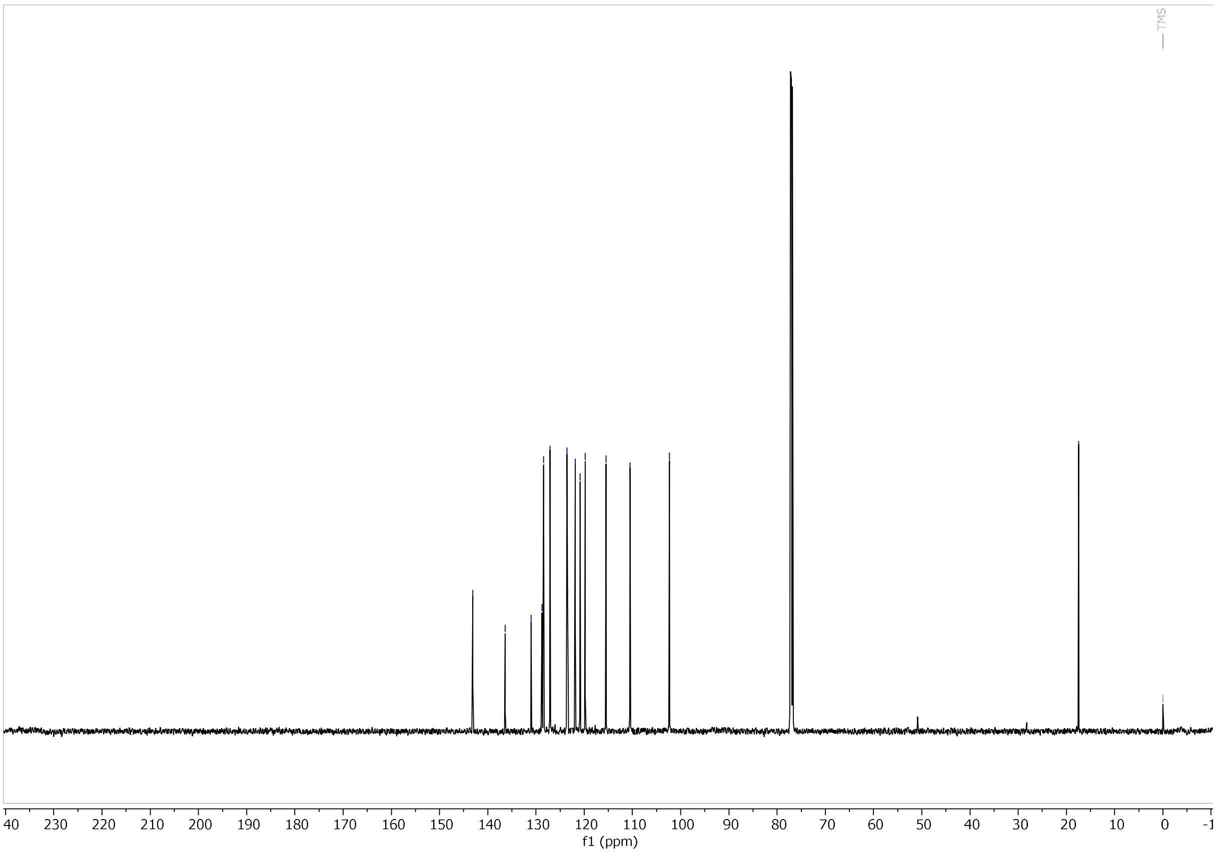

TMS
40
230
220
210
200
190
180
170
160
150
140
130
120
110
100
90
80
70
60
50
40
30
20
10
0
-1
f1 (ppm)

4-(1H-indol-1-yl)-3-methylaniline (5e)

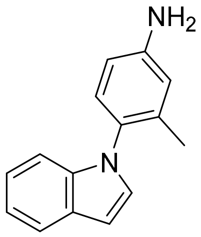

A two-neck round bottom flask was flushed with argon and charged with **4e** (1.01 g, 4 mmol), Pd/C (10%, 213 mg, 5 mol%) and MeOH (50 mL). The reaction was hydrogenated at room temperature using a hydrogen-filled balloon for 24 hours. The catalyst was filtered over a plug of silica with subsequent washing with MeOH. Upon solvent removal, the product was obtained as clear oil (854 mg, 96%).

^{1}H NMR (300 MHz, $CDCl_3$) δ 7.71 – 7.64 (m, 1H), 7.19 – 6.99 (m, 5H), 6.68 – 6.56 (m, 3H), 3.62 (s, 2H), 1.92 (s, 3H). ^{13}C NMR (75 MHz, $CDCl_3$) δ 146.0, 137.4, 137.1, 129.4, 129.2, 129.1, 128.1, 121.8, 120.7, 119.6, 117.0, 113.1, 110.5, 101.8, 17.5.

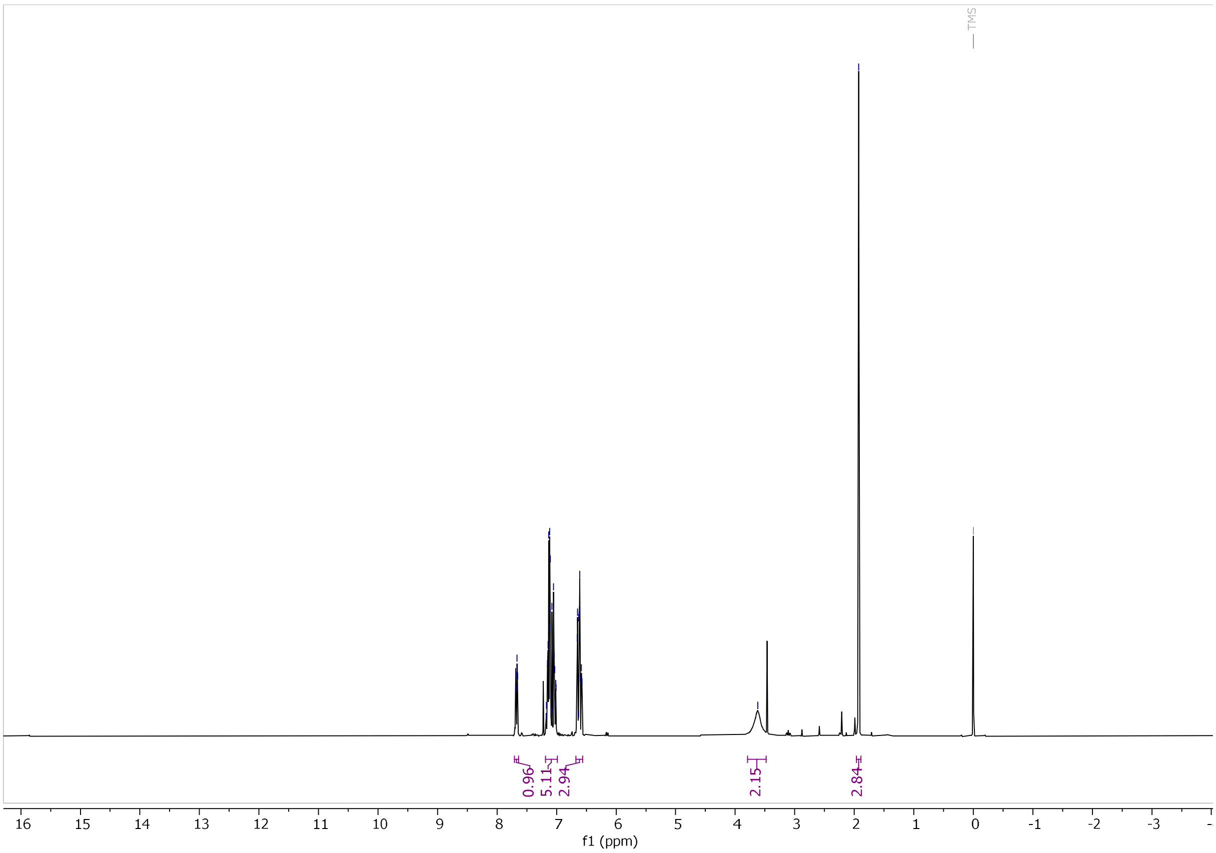

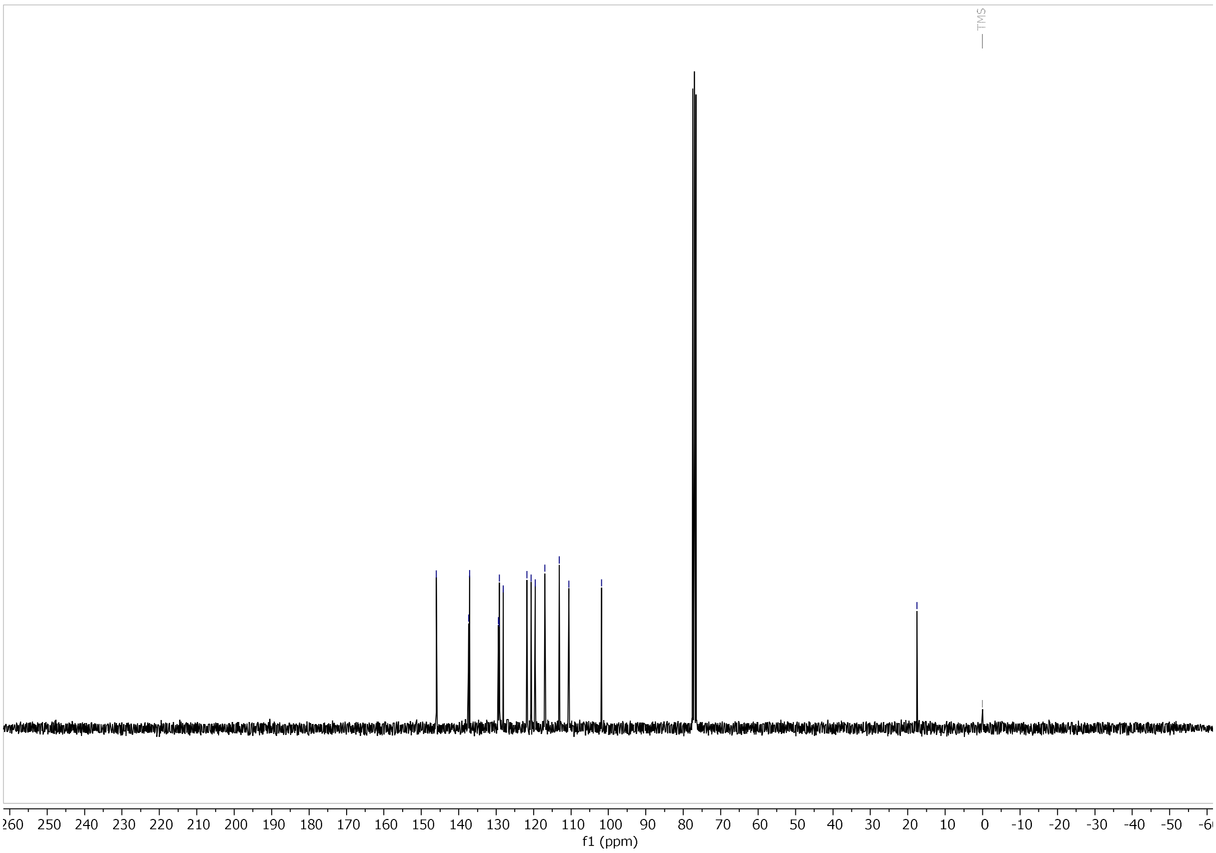
TMS
260 250 240 230 220 210 200 190 180 170 160 150 140 130 120 110 100 90 80 70 60 50 40 30 20 10 0 -10 -20 -30 -40 -50
f1 (ppm)

N-(4-(1H-indol-1-yl)phenyl)pyridin-3-amine (17)

A flame-dried vial was charged with **5c** (208 mg, 1 mmol), 3-bromopyridine (158 mg, 1 mmol), tBuXPhos Pd G3 (16 mg, 0.02 mmol), tBuXPhos (8 mg, 0.02 mmol), tBuONa (115 mg, 1.2 mmol) and THF (2 mL, Zero2, anhydrous, Sigma). The reaction was microwaved for 1 hour at 100 °C. It was then diluted with 25 mL of EtOAc and filtered through a pad of silica. The crude was purified by column chromatography on silica gel eluting with 40%-100% EtOAc in hexane. The product was obtained as brown solid (75 mg, 26%).

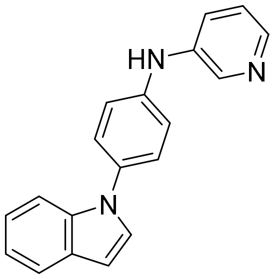

^{1}H NMR (300 MHz, $CDCl_3$) δ 8.66 (d, *J* = 2.8 Hz, 1H), 8.11 (dd, *J* = 5.0, 1.3 Hz, 1H), 7.74 – 7.62 (m, 2H), 7.54 – 7.49 (m, 1H), 7.47 – 7.41 (m, 2H), 7.35 – 7.13 (m, 7H), 6.68 (dd, *J* = 3.3, 0.9 Hz, 1H). ^{13}C NMR (75 MHz, $CDCl_3$) δ 141.5, 139.5, 137.5, 136.8, 136.0, 134.6, 129.1, 128.0, 125.8, 124.9, 124.4, 122.3, 121.1, 120.3, 120.1, 110.4, 103.3. HRMS (ESI): *m*/*z* calculated for $[M+H]^+$ 286.1339, found 286.1340.

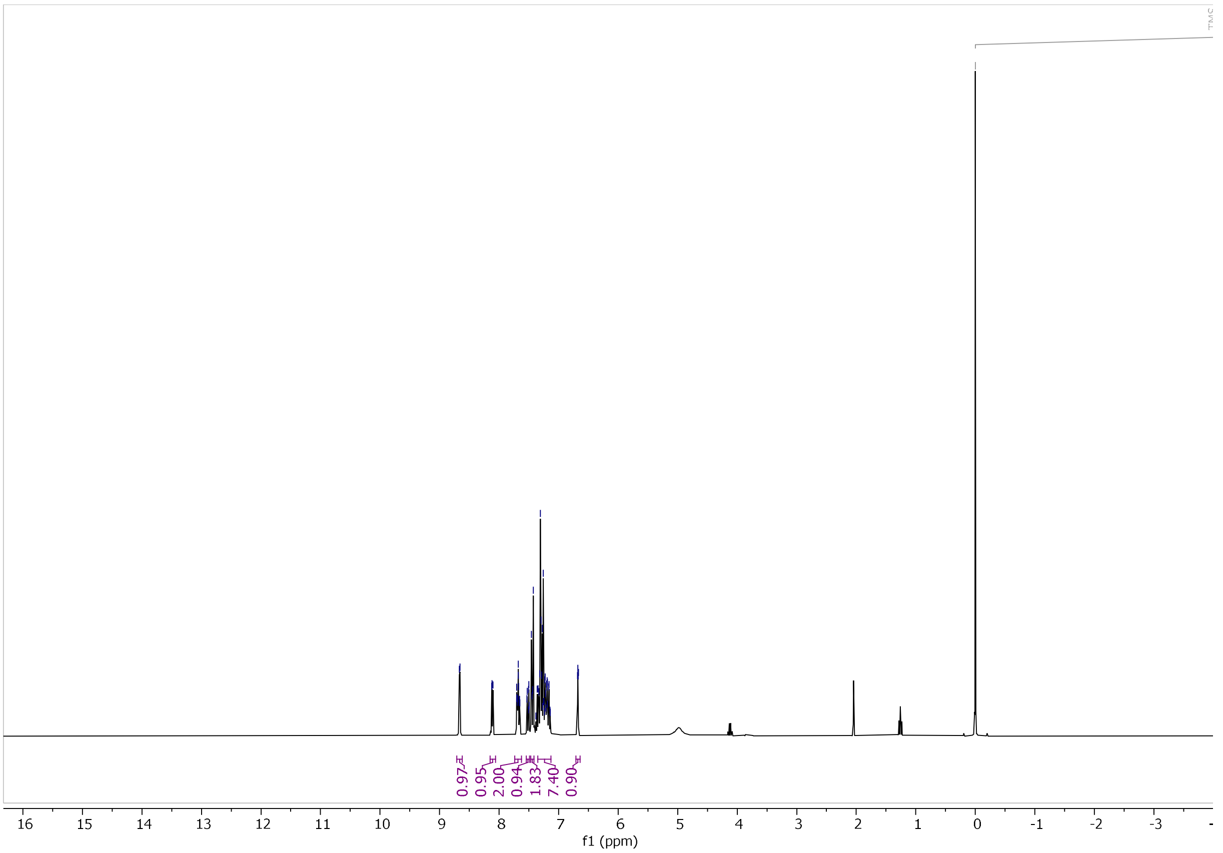

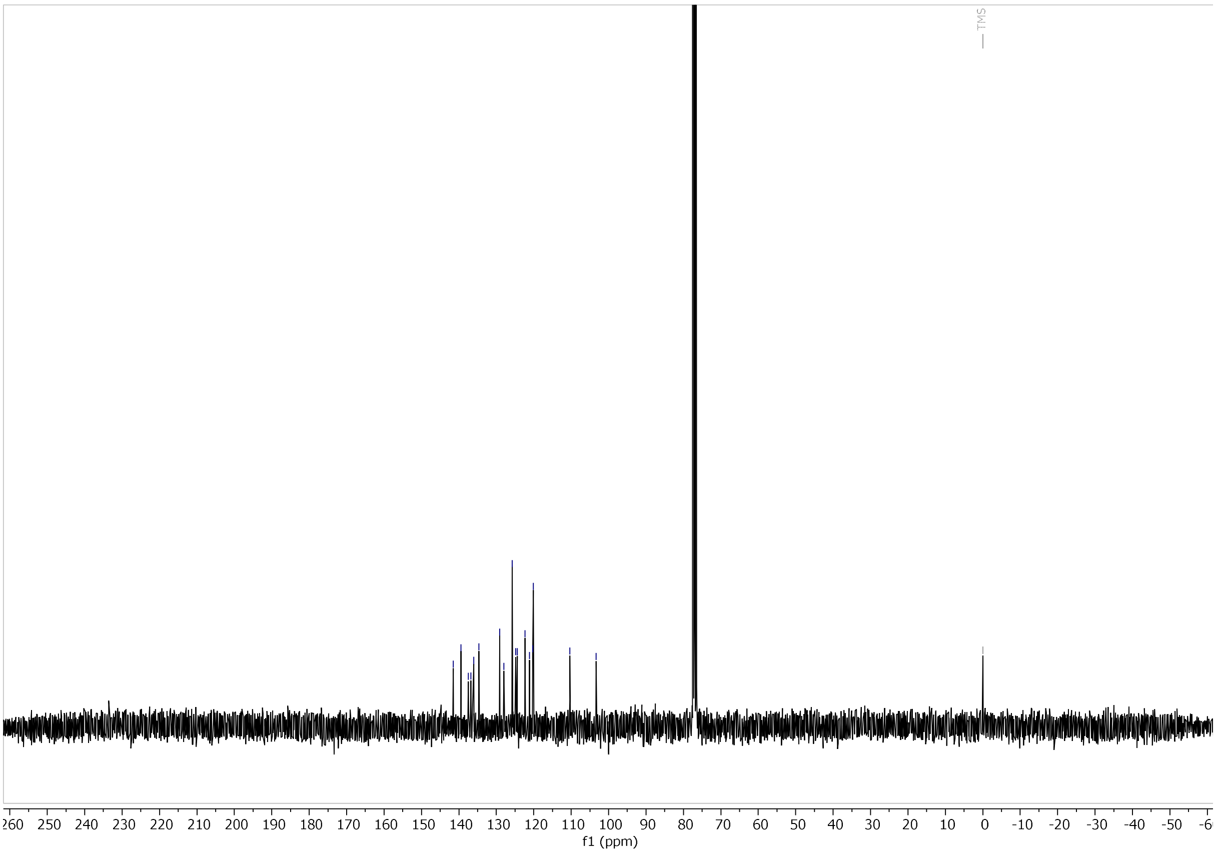
TMS
260 250 240 230 220 210 200 190 180 170 160 150 140 130 120 110 100 90 80 70 60 50 40 30 20 10 0 -10 -20 -30 -40 -50
f1 (ppm)

N-(4-(1H-indol-1-yl)-2-methylphenyl)pyridin-3-amine (18)

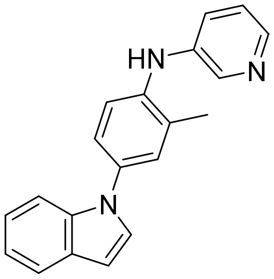

A flame-dried vial was charged with **5d** (222 mg, 1 mmol), 3-bromopyridine (174 mg, 1.1 mmol), tBuXPhos Pd G3 (40 mg, 0.05 mmol), tBuXPhos (21 mg, 0.05 mmol), tBuONa (192 mg, 2 mmol) and THF (2 mL, Zero2, anhydrous, Sigma). The reaction was microwaved for 1 hour at 100 °C. It was then diluted with 25 mL of EtOAc and filtered through a pad of silica. The crude was purified by column chromatography on silica gel eluting with 40%–100% EtOAc in hexane. The product was obtained as oil, which solidified upon treatment with hard vacuum (69 mg, 23%).

^{1}H NMR (300 MHz, DMSO) δ 8.32 (d, *J* = 2.5 Hz, 1H), 8.01 (dd, *J* = 4.5, 1.5 Hz, 1H), 7.80 (s, 1H), 7.64 (d, *J* = 7.6 Hz, 1H), 7.58 (d, *J* = 3.2 Hz, 1H), 7.54 (d, *J* = 7.8 Hz, 1H), 7.44 (s, 1H), 7.32 (d, *J* = 1.5 Hz, 2H), 7.29 (ddd, *J* = 8.3, 2.8, 1.6 Hz, 1H), 7.24 – 7.06 (m, 3H), 6.66 (dd, *J* = 3.2, 0.8 Hz, 1H), 2.31 (s, 3H). ^{13}C NMR (75 MHz, DMSO) δ 141.3, 140.1, 139.0, 138.8, 135.4, 133.6, 131.4, 128.9, 128.6, 126.6, 123.8, 122.3, 122.1, 122.0, 120.9, 120.6, 120.0, 110.4, 103.0, 18.0. HRMS (ESI): *m*/*z* calculated for [M+H]$^{+}$ 300.1495, found 300.1496.

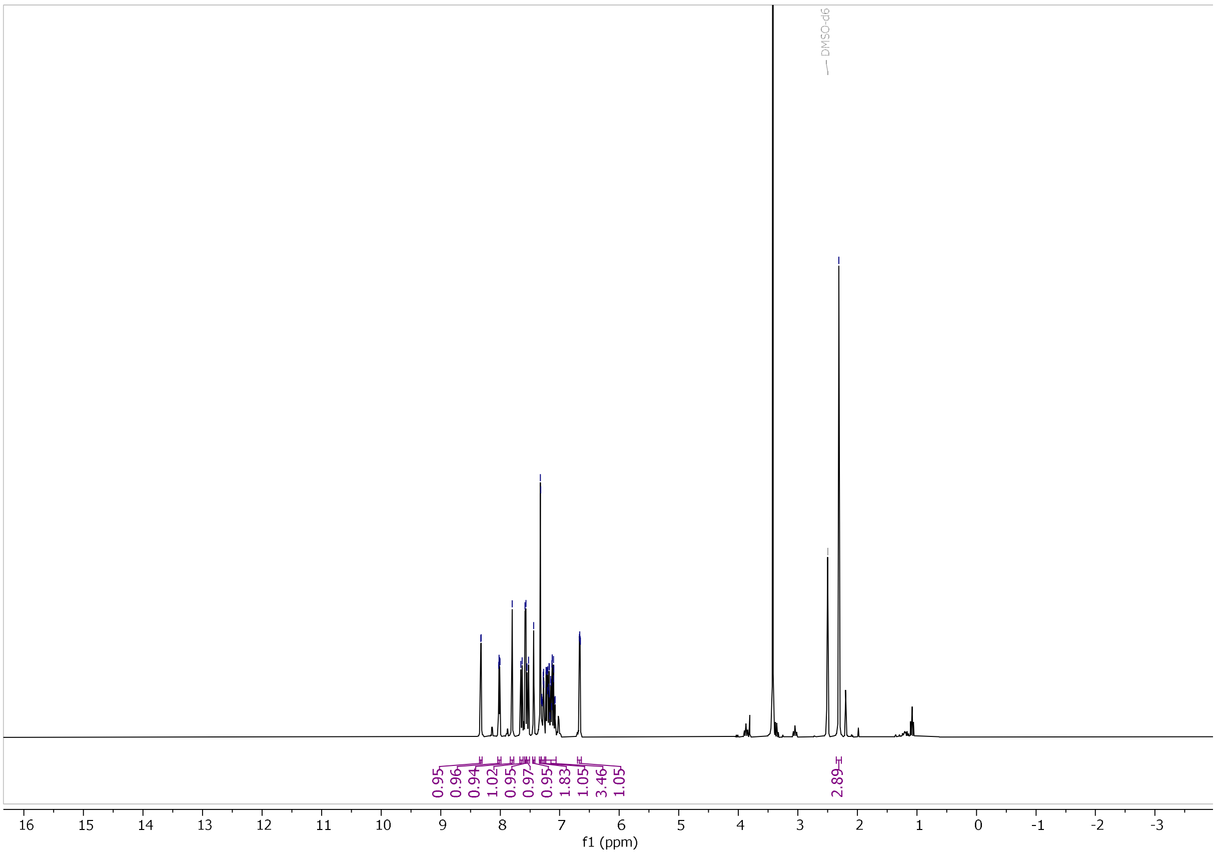

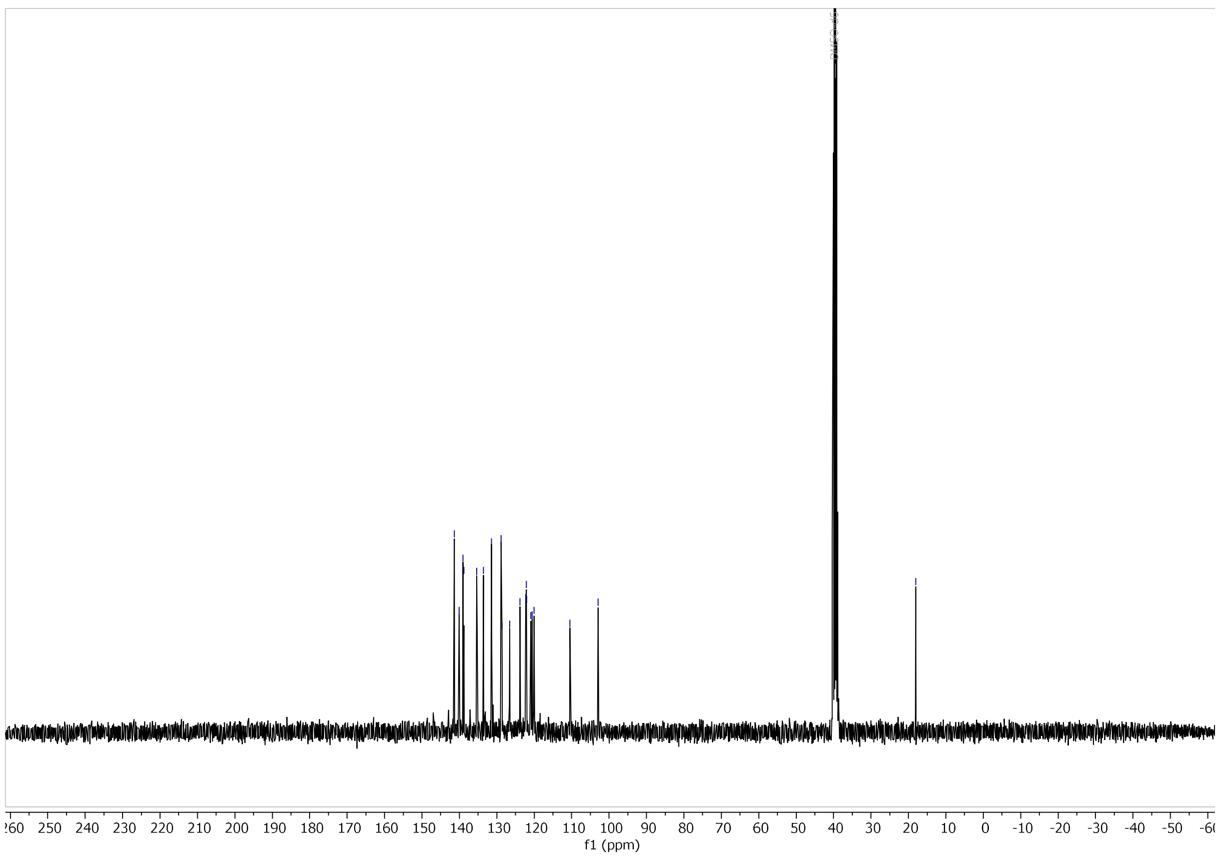
250 240 230 220 210 200 190 180 170 160 150 140 130 120 110 100 90 80 70 60 50 40 30 20 10 0 -10 -20 -30 -40 -50
f1 (ppm)

N-(4-(1H-indol-1-yl)phenyl)pyridin-2-amine (19)

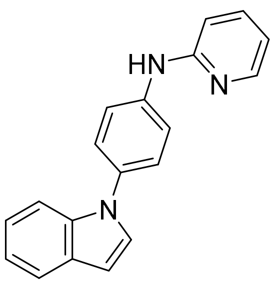

A flame-dried vial was charged with **5c** (208 mg, 1 mmol), 2-bromopyridine (174 mg, 1.1 mmol), tBuXPhos Pd G3 (40 mg, 0.05 mmol), tBuXPhos (21 mg, 0.05 mmol), tBuONa (192 mg, 2 mmol) and THF (2 mL, Zero2, anhydrous, Sigma). The reaction was microwaved for 1 hour at 100 °C. It was then diluted with 25 mL of EtOAc and filtered through a pad of silica. The crude was purified by column chromatography on silica gel eluting with 20% EtOAc in hexane. The product was obtained as hard resin (71 mg, 25%).

^{1}H NMR (300 MHz, DMSO) δ 9.25 (s, 1H), 8.18 (ddd, *J* = 5.0, 2.0, 0.8 Hz, 1H), 7.92 – 7.85 (m, 2H), 7.67 – 7.54 (m, 3H), 7.53 – 7.41 (m, 3H), 7.21 – 7.06 (m, 2H), 6.87 (dt, *J* = 8.6, 1.0 Hz, 1H), 6.77 (ddd, *J* = 7.1, 5.0, 0.9 Hz, 1H), 6.65 (dd, *J* = 3.2, 0.8 Hz, 1H). ^{13}C NMR (75 MHz, DMSO) δ 155.7, 147.3, 140.4, 137.4, 135.5, 131.6, 128.7, 128.7, 124.5, 122.1, 120.8, 119.9, 118.7, 114.6, 111.0, 110.4, 102.7. HRMS (ESI): *m/z* calculated for [M+H]$^{+}$ 286.1339, found 286.1343.

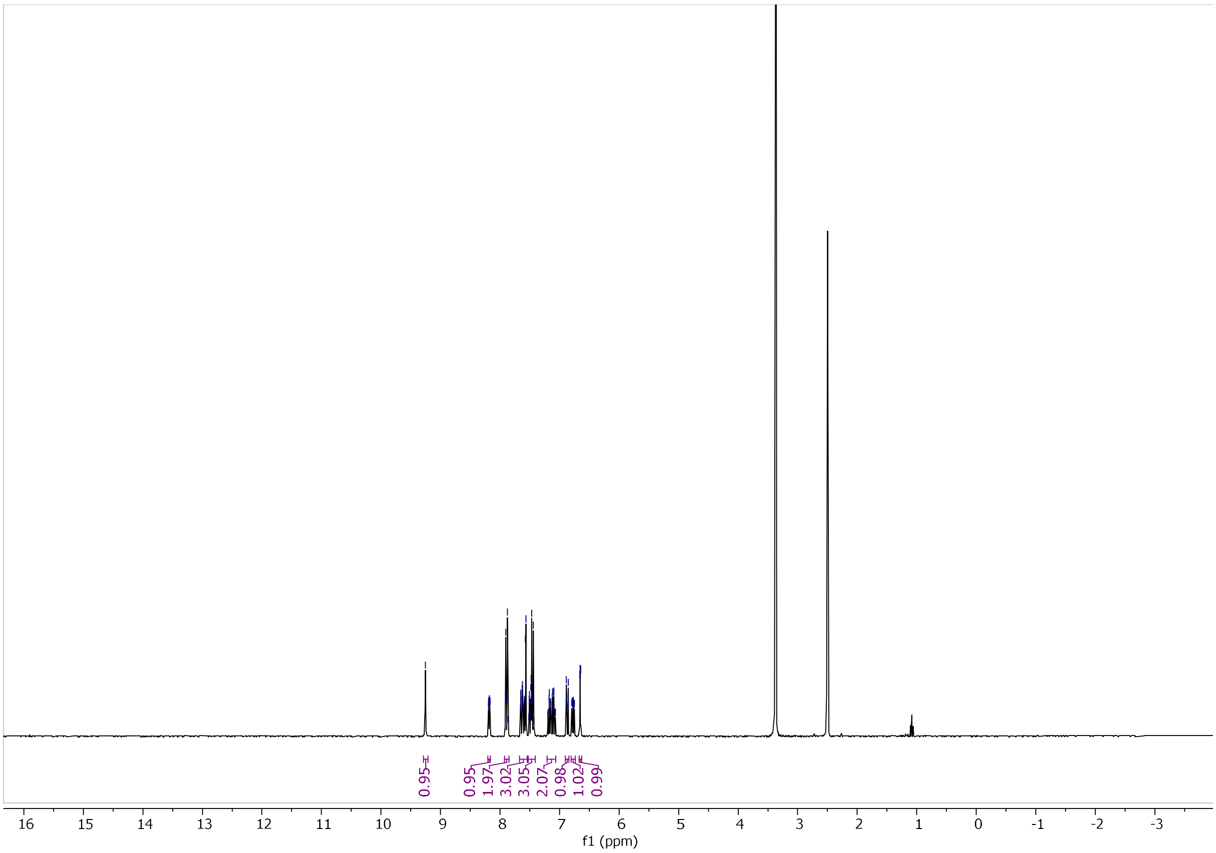

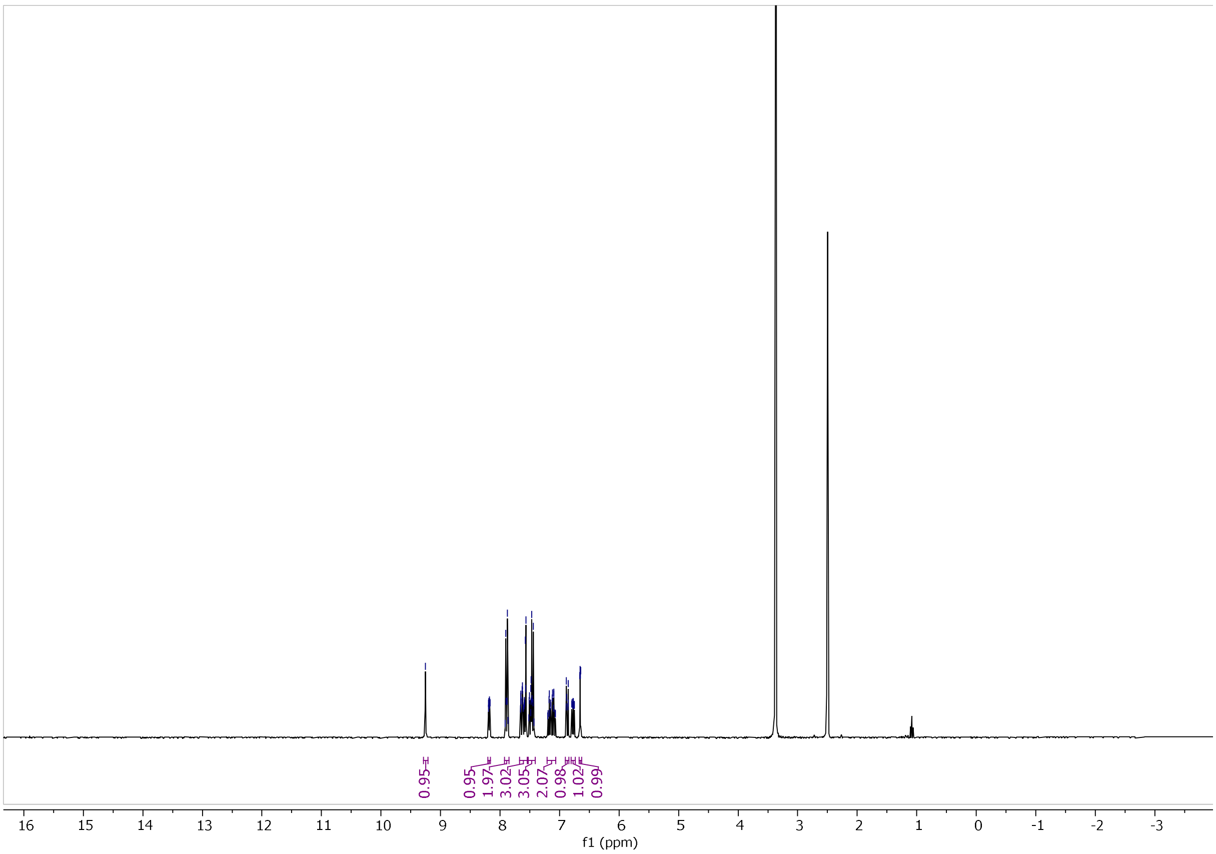
0.95
0.95
1.97
3.02
3.05
2.07
0.98
1.02
0.99
16 15 14 13 12 11 10 9 8 7 6 5 4 3 2 1 0 -1 -2 -3
f1 (ppm)

N-(4-(1H-indol-1-yl)-3-methylphenyl)pyridin-3-amine (20)

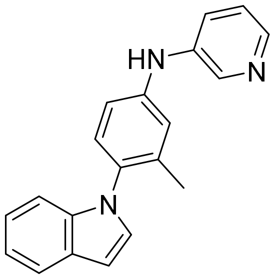

A flame-dried vial was charged with **5e** (222 mg, 1 mmol), 3-bromopyridine (174 mg, 1.1 mmol), tBuXPhos Pd G3 (40 mg, 0.05 mmol), tBuXPhos (21 mg, 0.05 mmol), tBuONa (192 mg, 2 mmol) and THF (2 mL, Zero2, anhydrous, Sigma). The reaction was microwaved for 1 hour at 100 °C. It was then diluted with 25 mL of EtOAc and filtered through a pad of silica. The crude was purified by column chromatography on silica gel eluting with 40–100% EtOAc in hexane. Obtained oil was sonicated with ether producing beige solid (97 mg, 32%)

^{1}H NMR (300 MHz, DMSO) δ 8.57 (s, 1H), 8.42 (d, *J* = 2.7 Hz, 1H), 8.08 (dd, *J* = 4.6, 1.4 Hz, 1H), 7.66 – 7.54 (m, 2H), 7.40 (d, *J* = 3.2 Hz, 1H), 7.28 (dd, *J* = 8.3, 4.6 Hz, 1H), 7.20 (d, *J* = 8.4 Hz, 1H), 7.16 – 7.03 (m, 4H), 7.03 – 6.93 (m, 1H), 6.63 (d, *J* = 3.1 Hz, 1H), 1.92 (s, 3H). ^{13}C NMR (75 MHz, DMSO) δ 142.7, 141.1, 139.8, 139.6, 136.8, 136.2, 130.1, 129.7, 128.8, 127.9, 124.0, 123.1, 121.8, 120.6, 119.6, 118.4, 114.8, 110.2, 101.9, 17.4. HRMS (ESI): *m/z* calculated for $[M+H]^+$ 300.1495, found 300.1497.

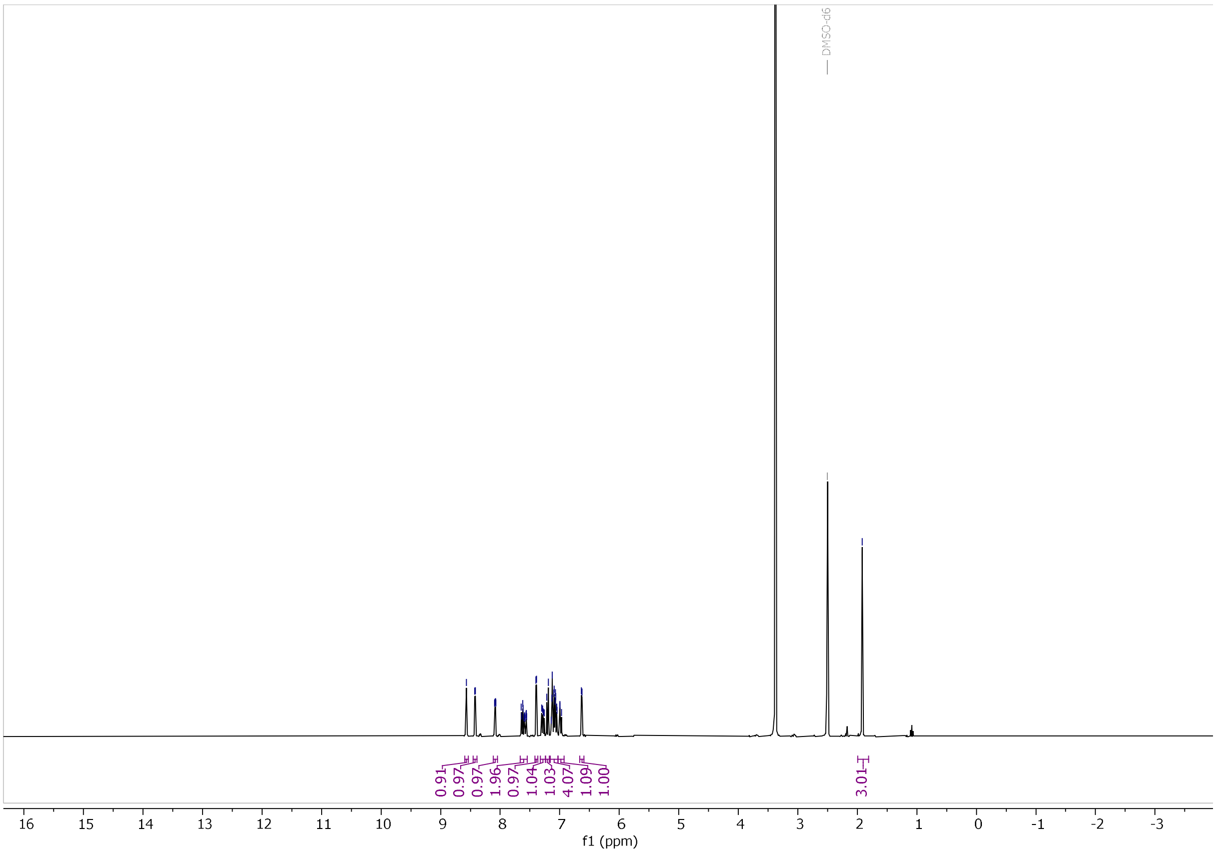

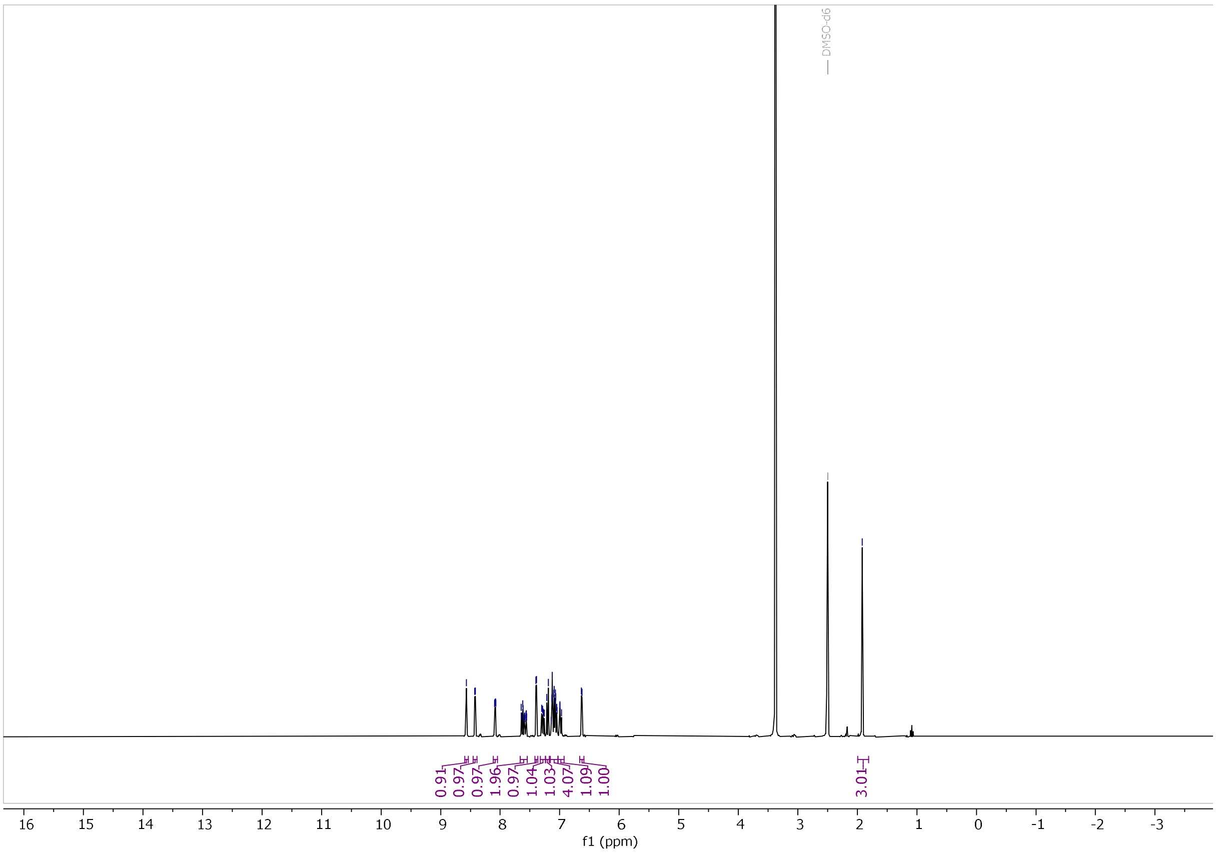
DMSO-d6
0.91
0.97
0.97
1.96
0.97
1.04
1.03
4.07
1.09
1.00
3.01
16 15 14 13 12 11 10 9 8 7 6 5 4 3 2 1 0 -1 -2 -3
f1 (ppm)

HPLC Purity Chromatograms

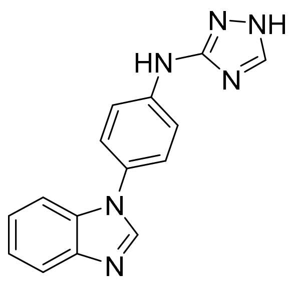

1

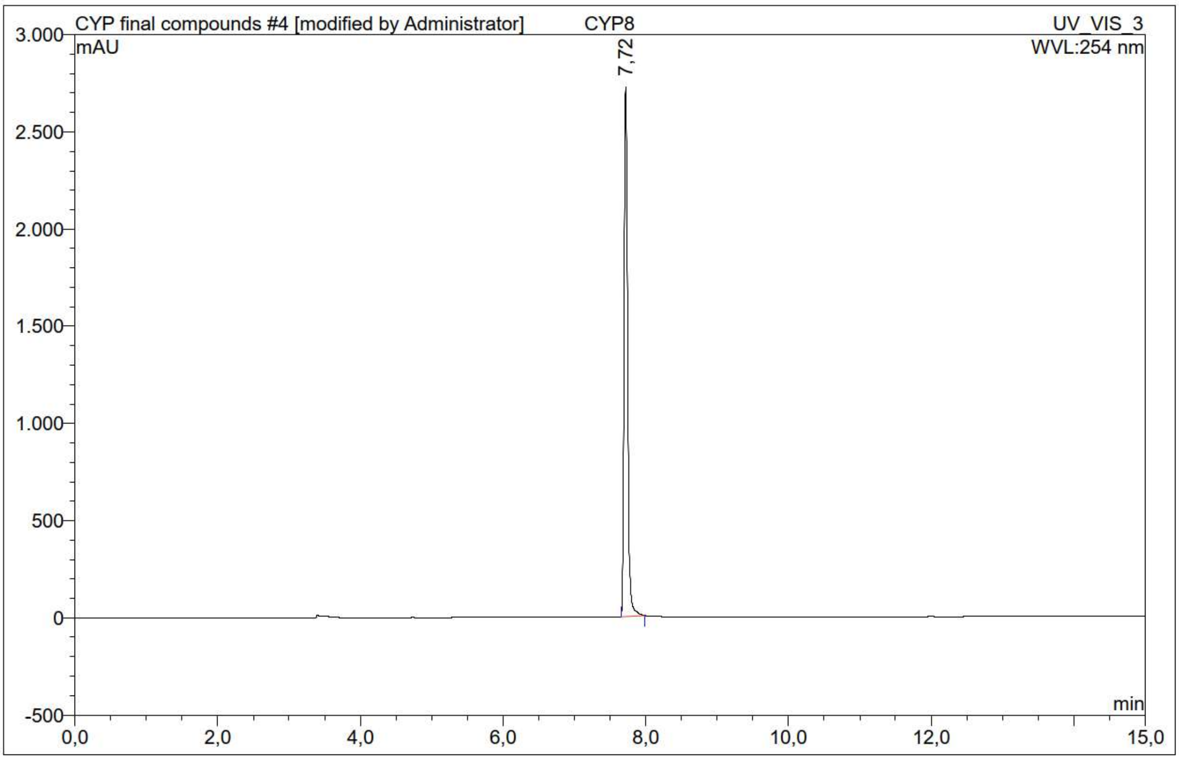

No.	Ret.Time min	Peak Name	Height mAU	Area mAU*min	Rel.Area %	Amount	Resolution(EP)
1	7,72	n.a.	2725,631	155,430	100,00	n.a.	n.a.
Total:			2725,631	155,430	100,00	0,000	

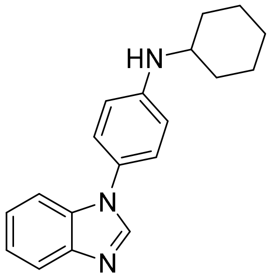

2

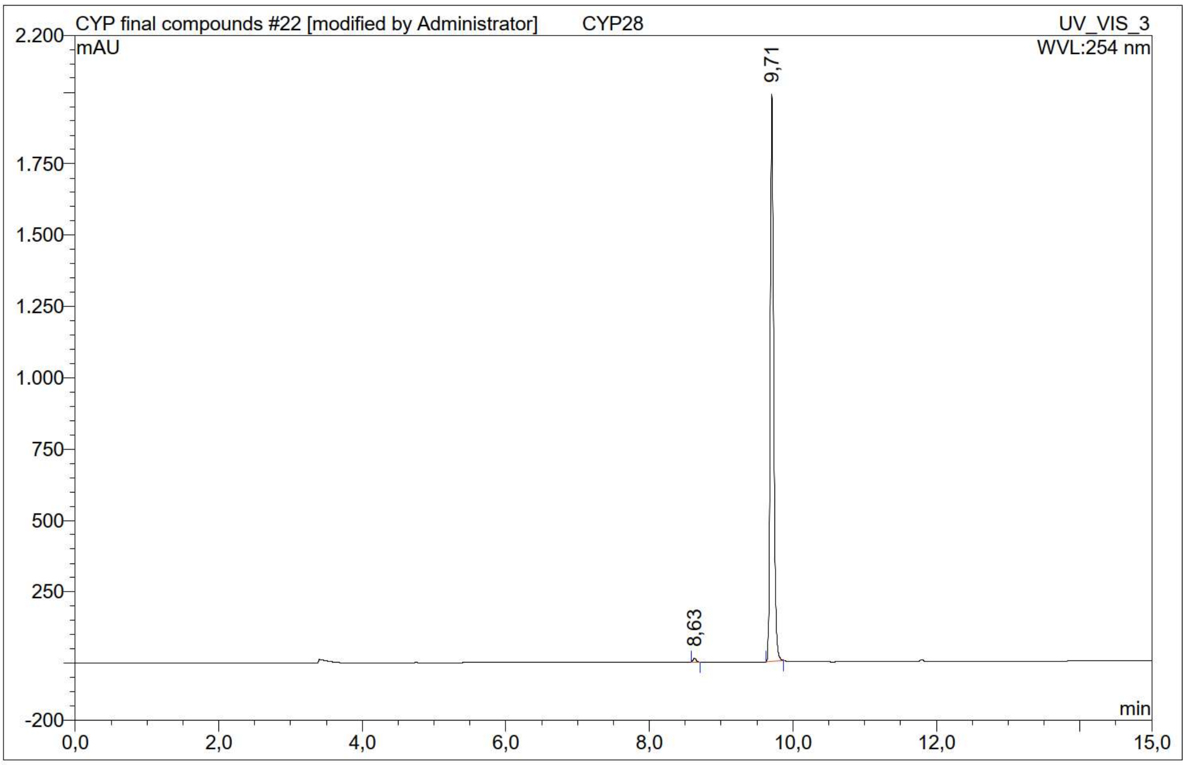

No.	Ret.Time min	Peak Name	Height mAU	Area mAU*min	Rel.Area %	Amount	Resolution(EP)
1	8,63	n.a.	13,397	0,682	0,59	n.a.	12,62
2	9,71	n.a.	1987,781	114,143	99,41	n.a.	n.a.
**Total:**			2001,178	114,825	100,00	0,000	

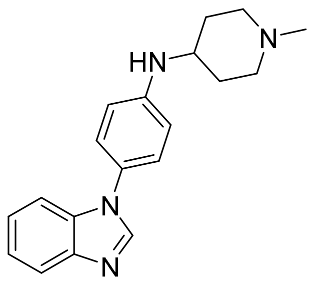

3

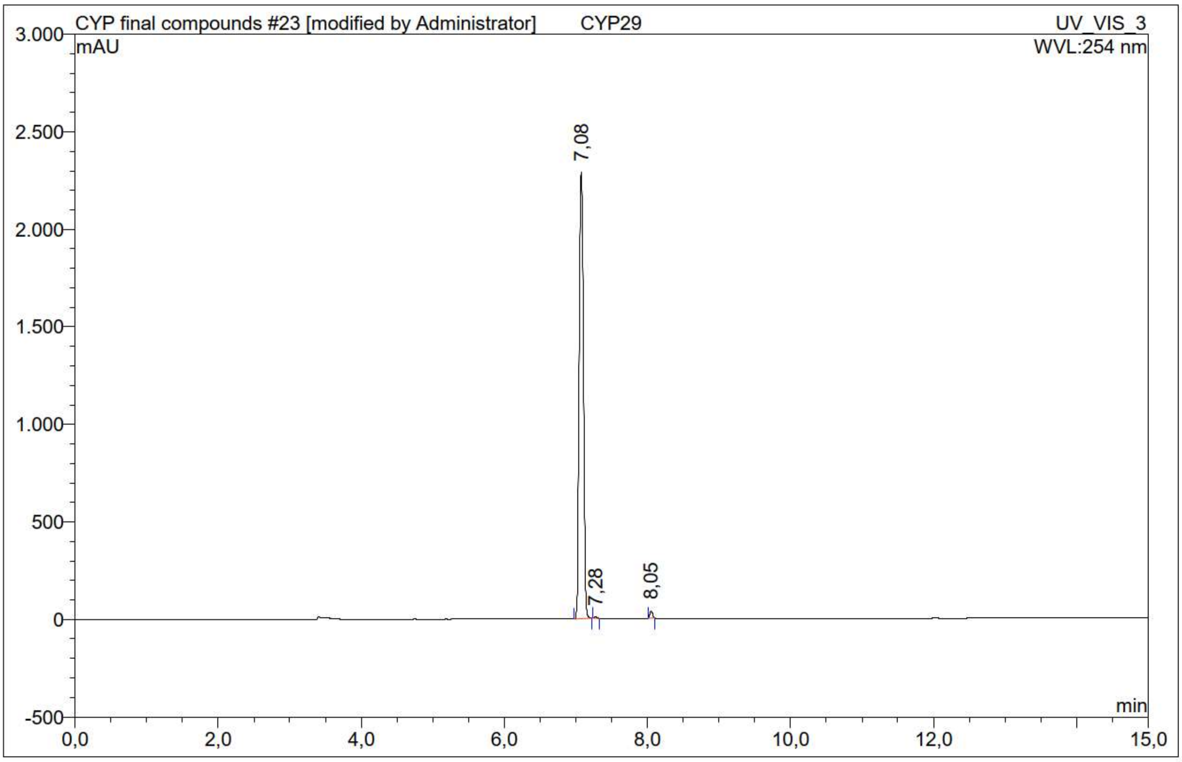

No.	Ret.Time min	Peak Name	Height mAU	Area mAU*min	Rel.Area %	Amount	Resolution(EP)
1	7,08	n.a.	2290,113	159,700	98,72	n.a.	1,97
2	7,28	n.a.	6,711	0,353	0,22	n.a.	9,27
3	8,05	n.a.	36,453	1,711	1,06	n.a.	n.a.
**Total:**			2333,277	161,763	100,00	0,000	

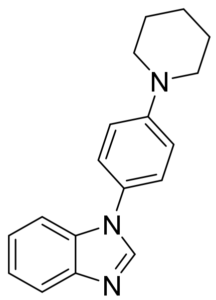

4

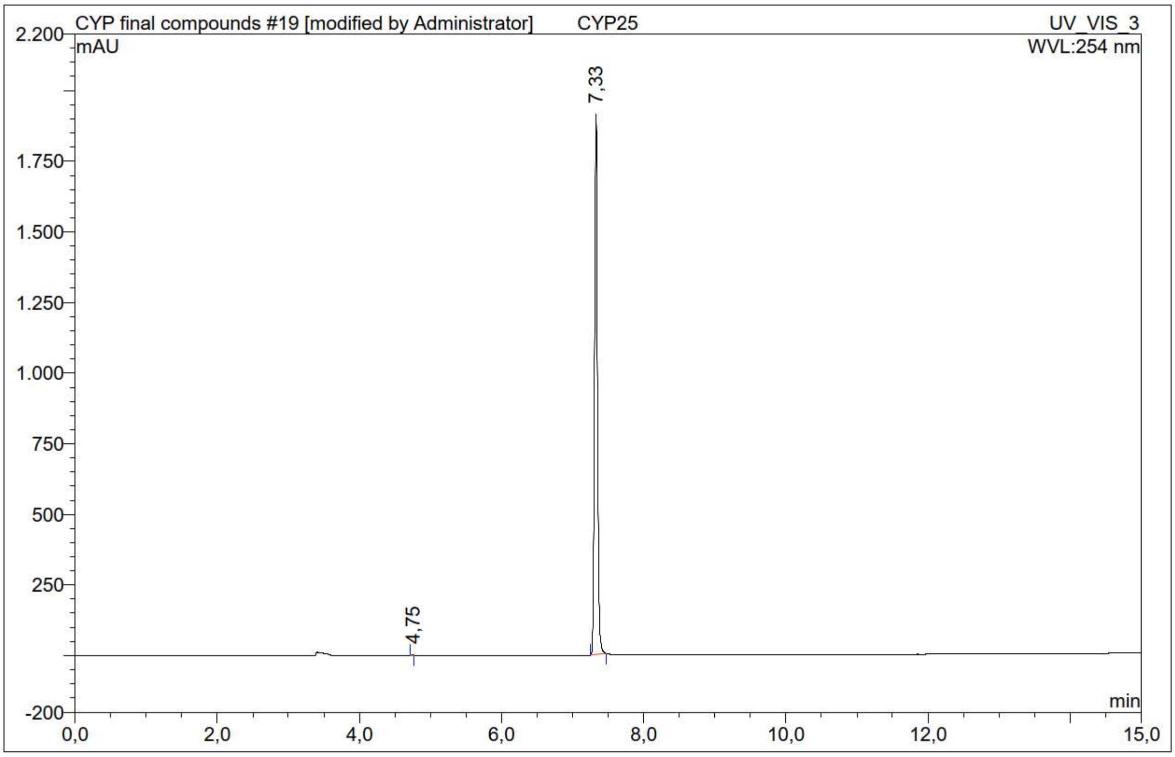

No.	Ret.Time min	Peak Name	Height mAU	Area mAU*min	Rel.Area %	Amount	Resolution(EP)
1	4,75	n.a.	2,197	0,043	0,04	n.a.	45,95
2	7,33	n.a.	1912,183	97,253	99,96	n.a.	n.a.
**Total:**			1914,380	97,296	100,00	0,000	

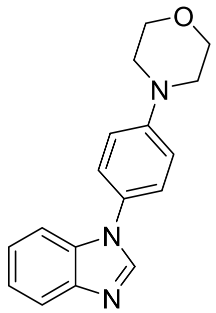

5

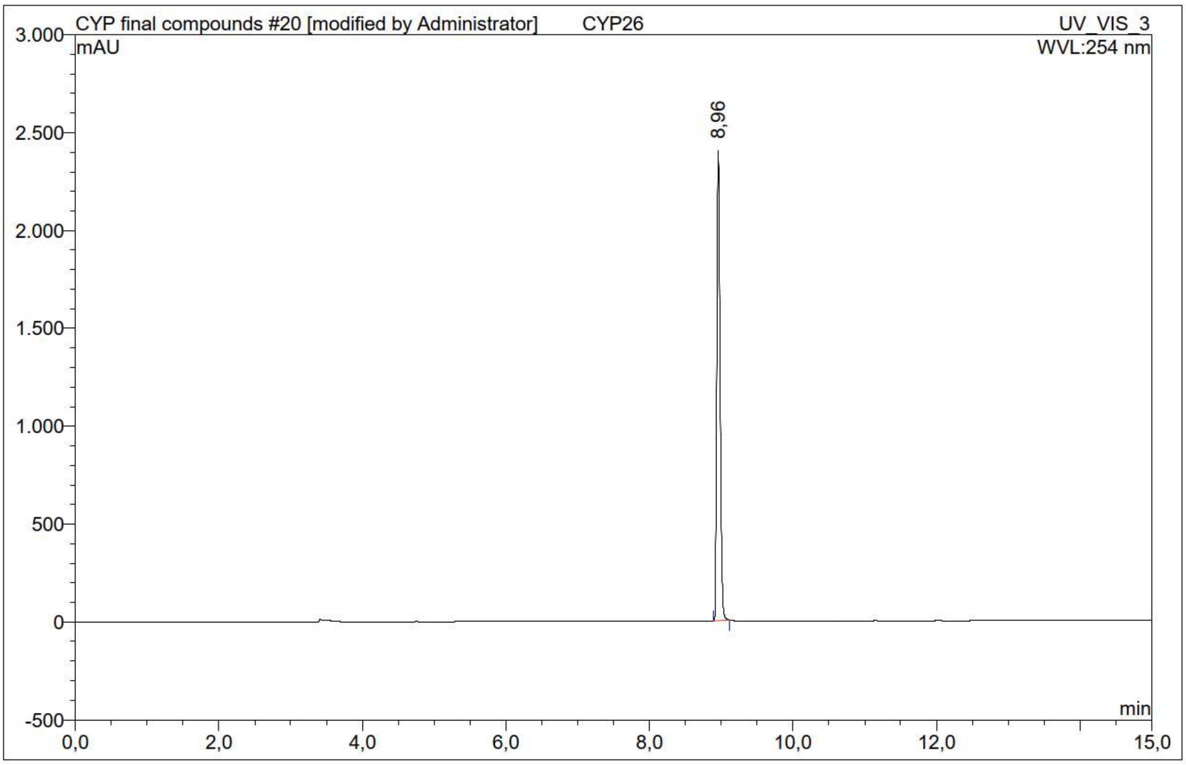

No.	Ret.Time min	Peak Name	Height mAU	Area mAU*min	Rel.Area %	Amount	Resolution(EP)
1	8,96	n.a.	2403,871	126,629	100,00	n.a.	n.a.
Total:			2403,871	126,629	100,00	0,000	

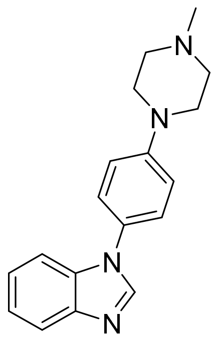

6

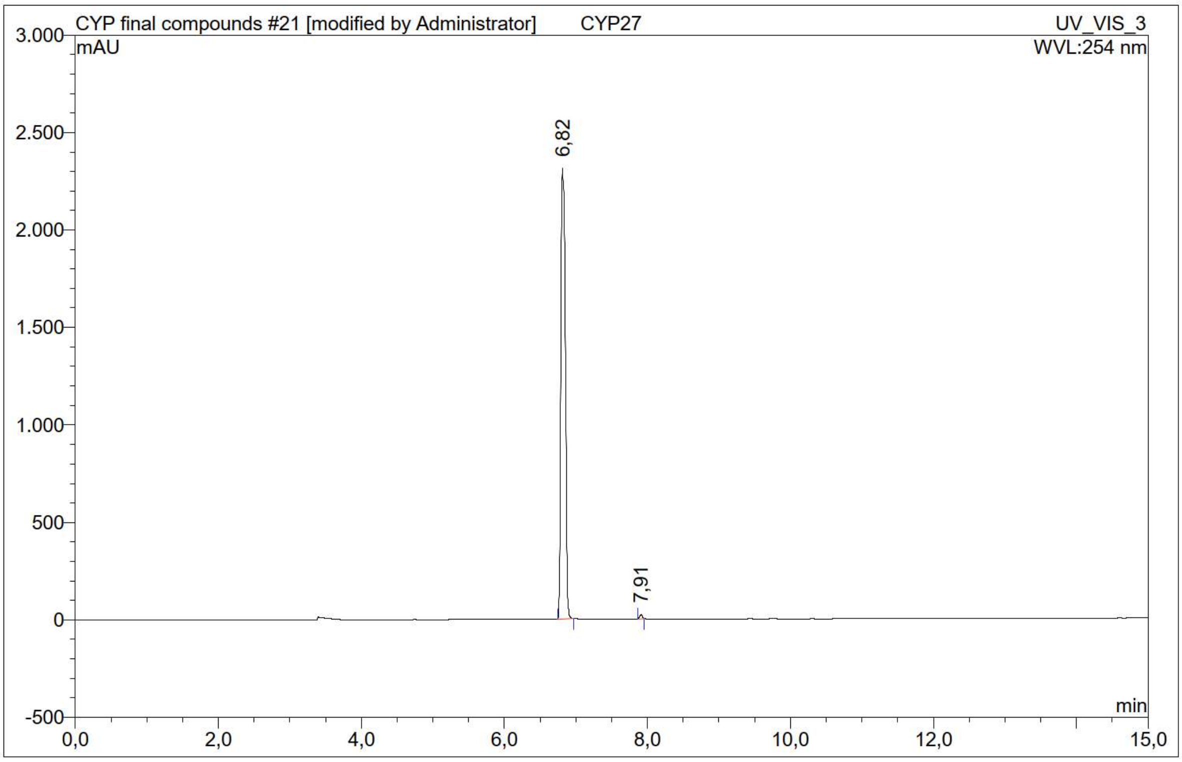

No.	Ret.Time min	Peak Name	Height mAU	Area mAU*min	Rel.Area %	Amount	Resolution(EP)
1	6,82	n.a.	2314,540	159,743	99,37	n.a.	11,47
2	7,91	n.a.	21,673	1,007	0,63	n.a.	n.a.
**Total:**			2336,213	160,750	100,00	0,000	

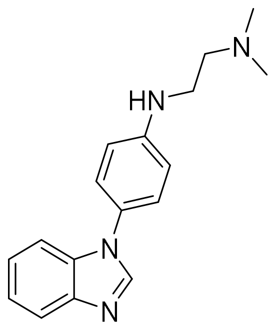

7

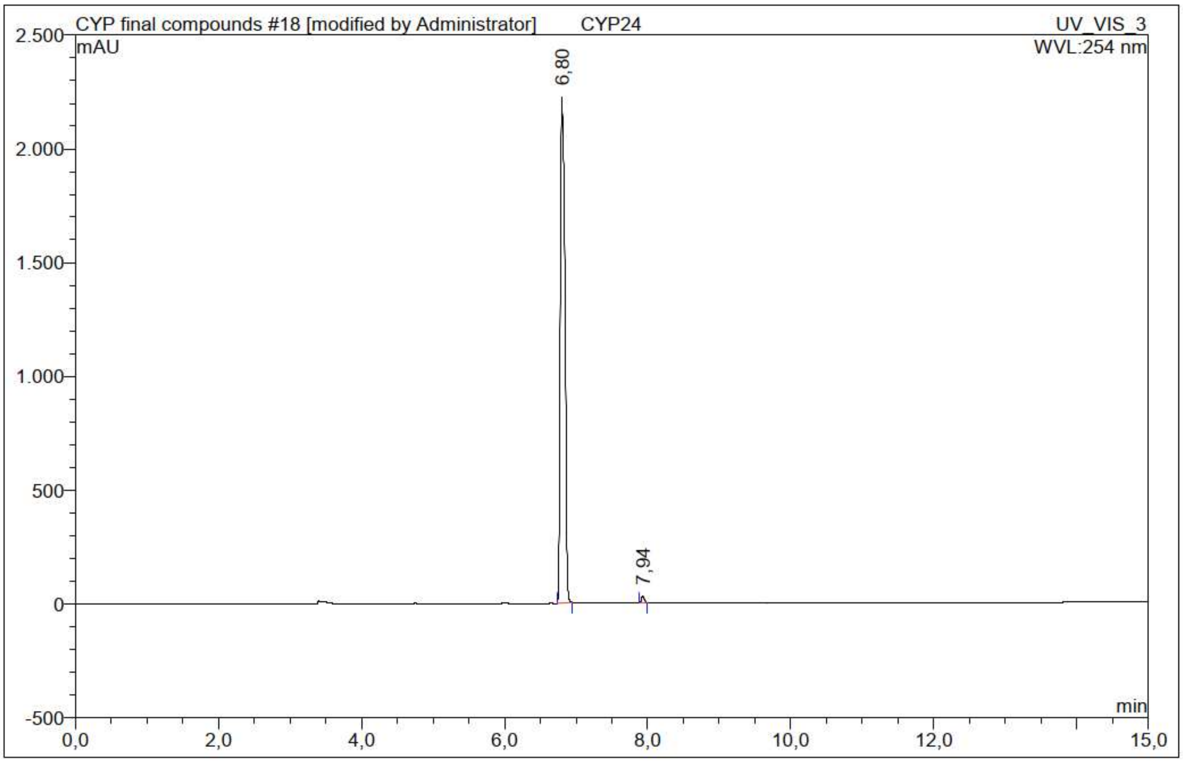

No.	Ret.Time min	Peak Name	Height mAU	Area mAU*min	Rel.Area %	Amount	Resolution(EP)
1	6,80	n.a.	2224,184	160,404	99,13	n.a.	11,53
2	7,94	n.a.	30,058	1,403	0,87	n.a.	n.a.
**Total:**			2254,242	161,807	100,00	0,000	

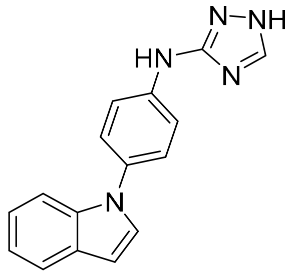

8

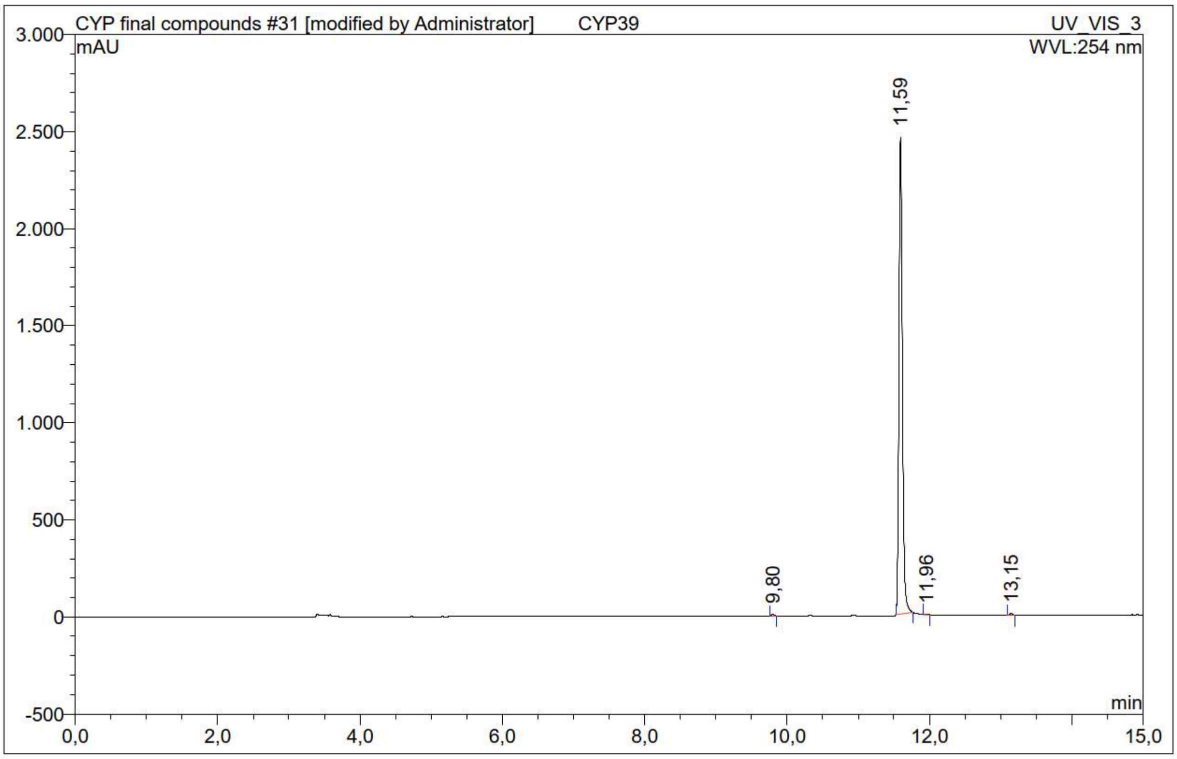

No.	Ret.Time min	Peak Name	Height mAU	Area mAU*min	Rel.Area %	Amount	Resolution(EP)
1	9,80	n.a.	7,502	0,330	0,24	n.a.	22,75
2	11,59	n.a.	2455,410	137,937	99,37	n.a.	4,15
3	11,96	n.a.	1,706	0,092	0,07	n.a.	13,29
4	13,15	n.a.	8,390	0,448	0,32	n.a.	n.a.
**Total:**			2473,009	138,807	100,00	0,000	

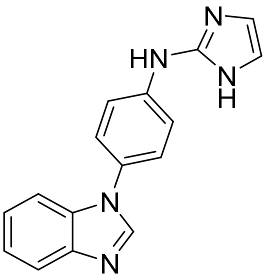

9

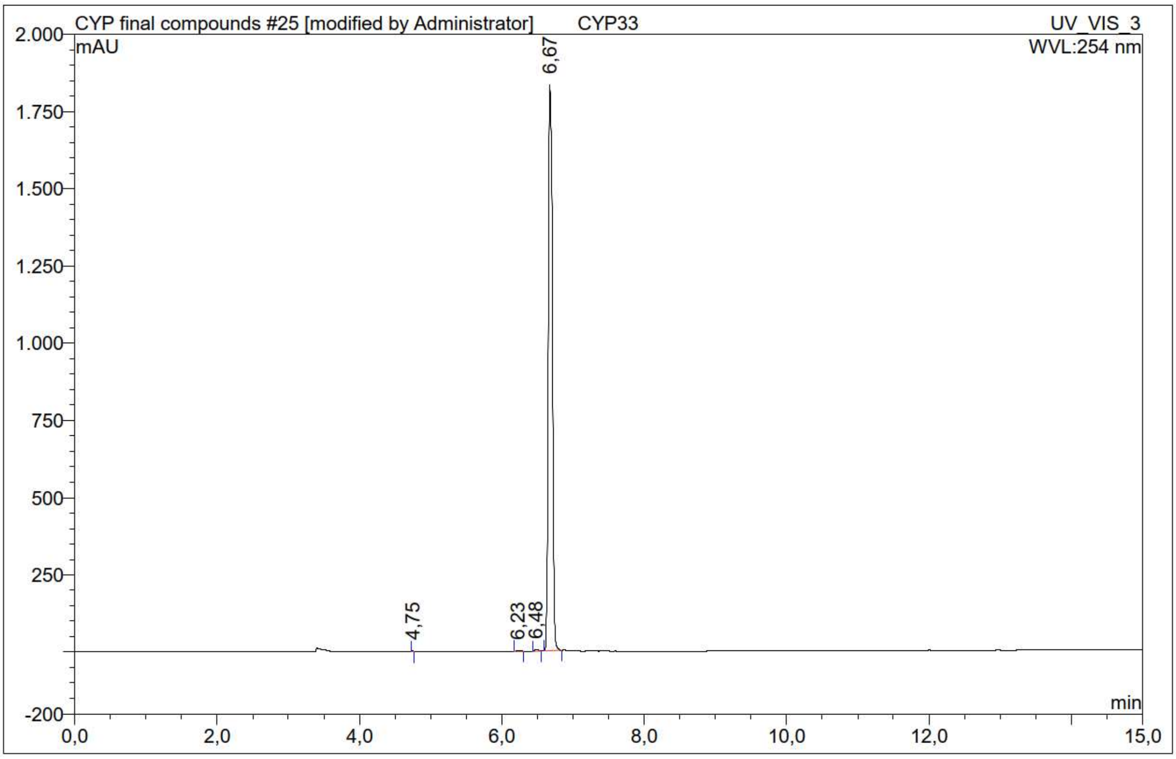

No.	Ret.Time min	Peak Name	Height mAU	Area mAU*min	Rel.Area %	Amount	Resolution(EP)
1	4,75	n.a.	2,337	0,045	0,04	n.a.	27,91
2	6,23	n.a.	1,945	0,111	0,09	n.a.	2,59
3	6,48	n.a.	4,889	0,339	0,27	n.a.	1,68
4	6,67	n.a.	1832,004	123,824	99,60	n.a.	n.a.
**Total:**			1841,175	124,319	100,00	0,000	

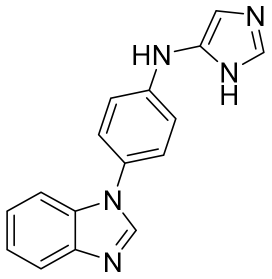

10

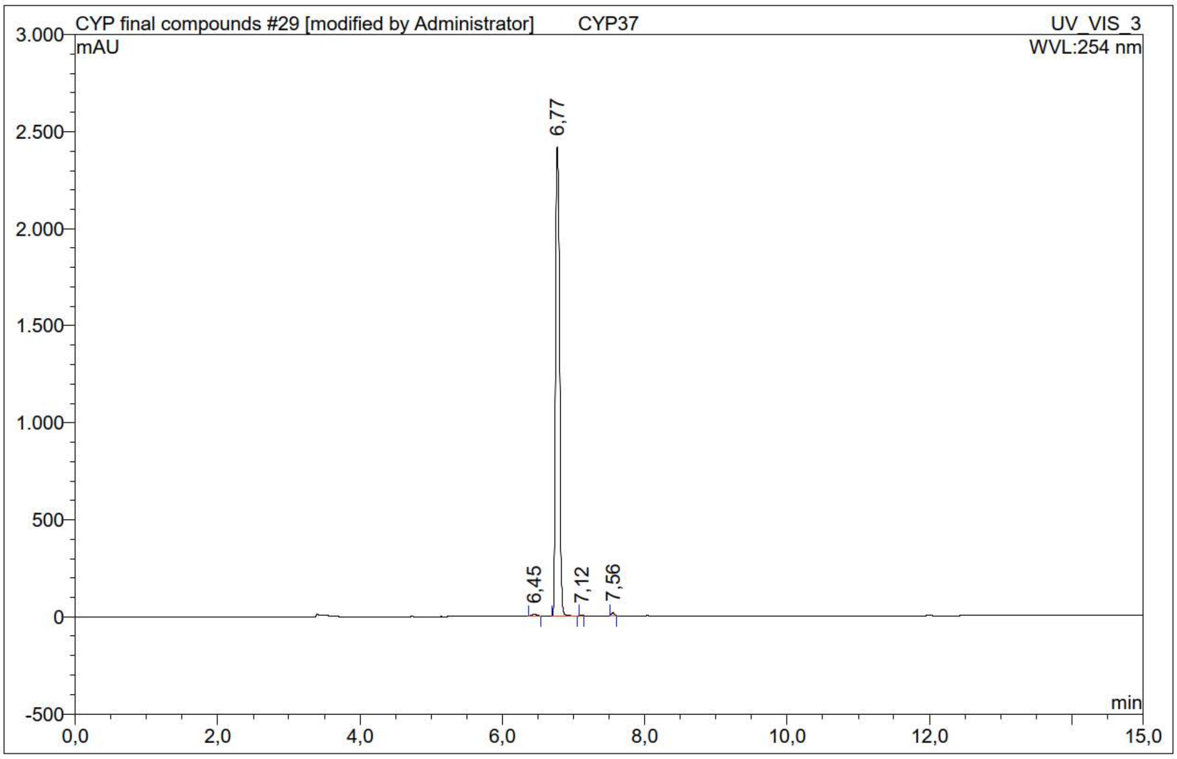

No.	Ret.Time min	Peak Name	Height mAU	Area mAU*min	Rel.Area %	Amount	Resolution(EP)
1	6,45	n.a.	7,430	0,769	0,49	n.a.	2,25
2	6,77	n.a.	2418,341	156,580	98,93	n.a.	4,02
3	7,12	n.a.	3,353	0,126	0,08	n.a.	6,24
4	7,56	n.a.	17,267	0,791	0,50	n.a.	n.a.
**Total:**			2446,392	158,266	100,00	0,000	

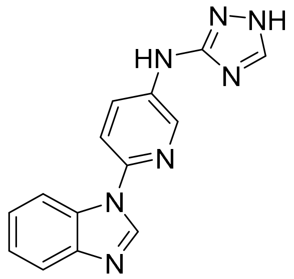

11

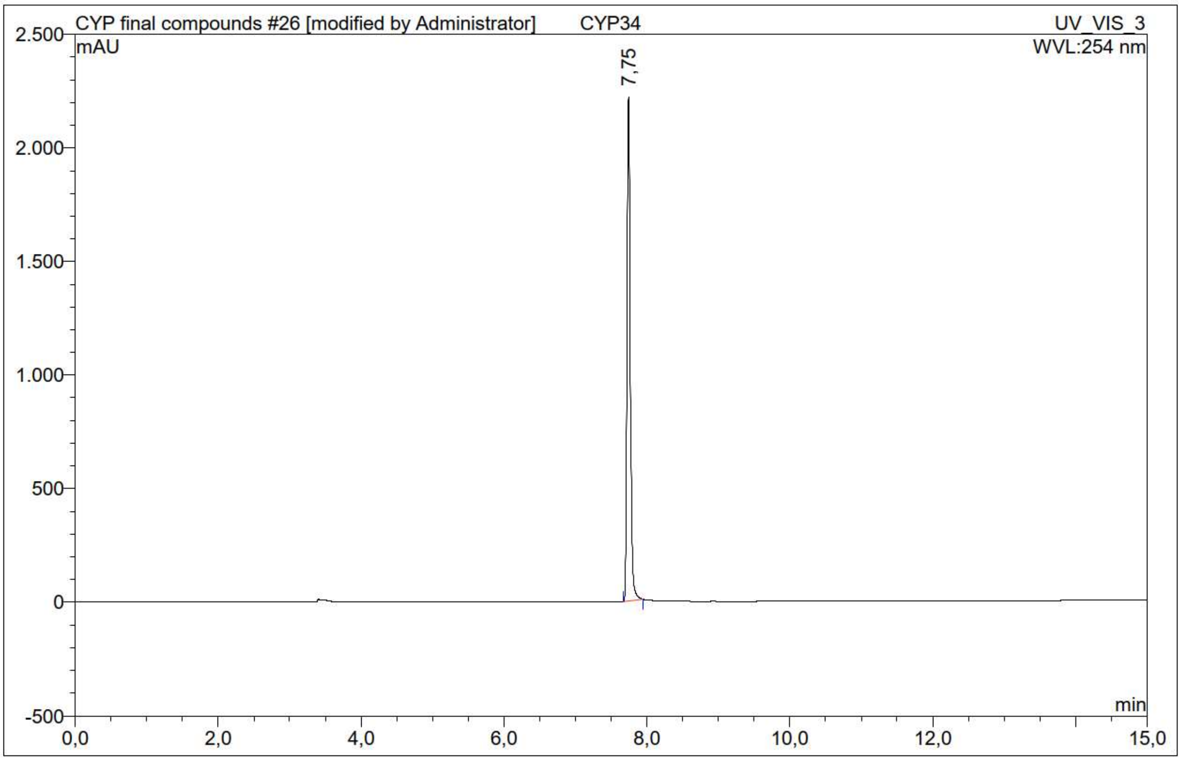

No.	Ret.Time min	Peak Name	Height mAU	Area mAU*min	Rel.Area %	Amount	Resolution(EP)
1	7,75	n.a.	2218,828	116,296	100,00	n.a.	n.a.
Total:			2218,828	116,296	100,00	0,000	

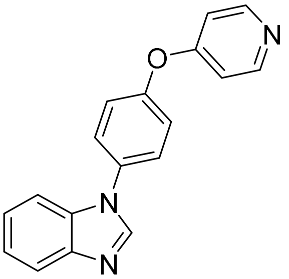

12

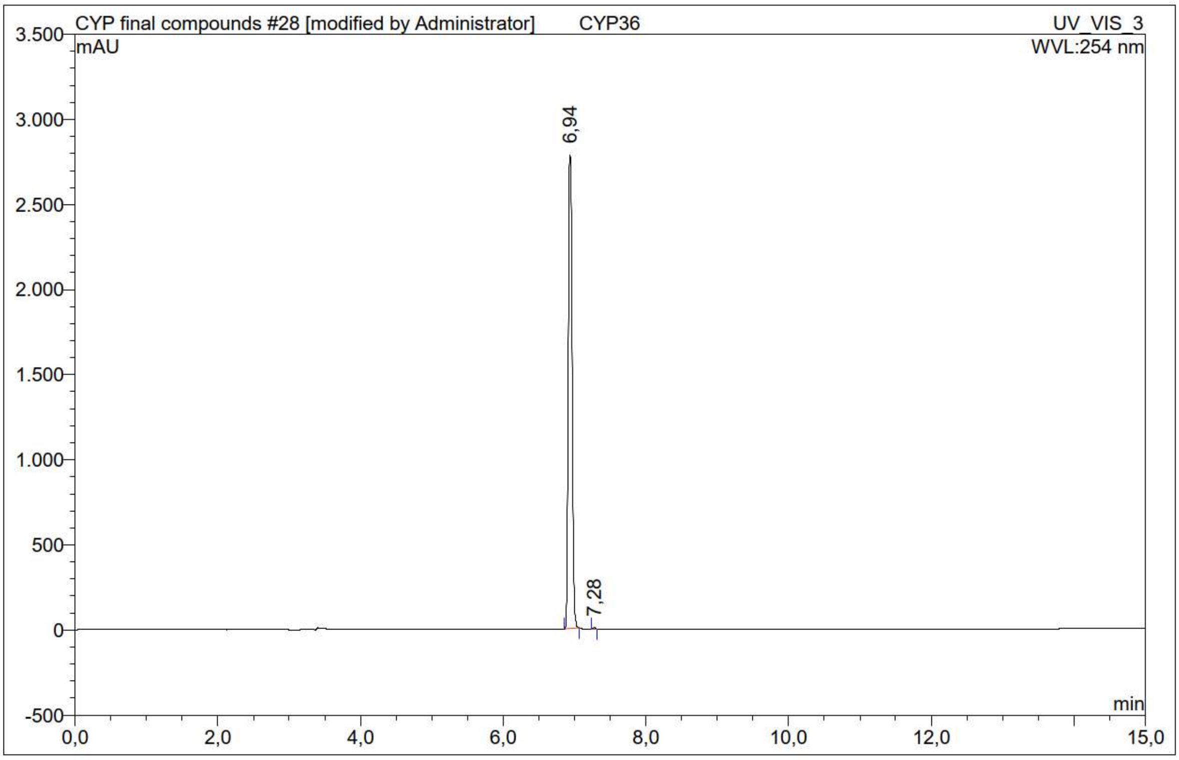

No.	Ret.Time min	Peak Name	Height mAU	Area mAU*min	Rel.Area %	Amount	Resolution(EP)
1	6,94	n.a.	2783,988	182,253	99,81	n.a.	3,80
2	7,28	n.a.	8,102	0,342	0,19	n.a.	n.a.
**Total:**			2792,090	182,595	100,00	0,000	

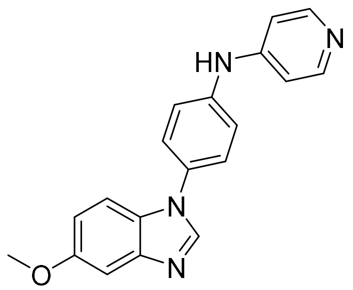

13

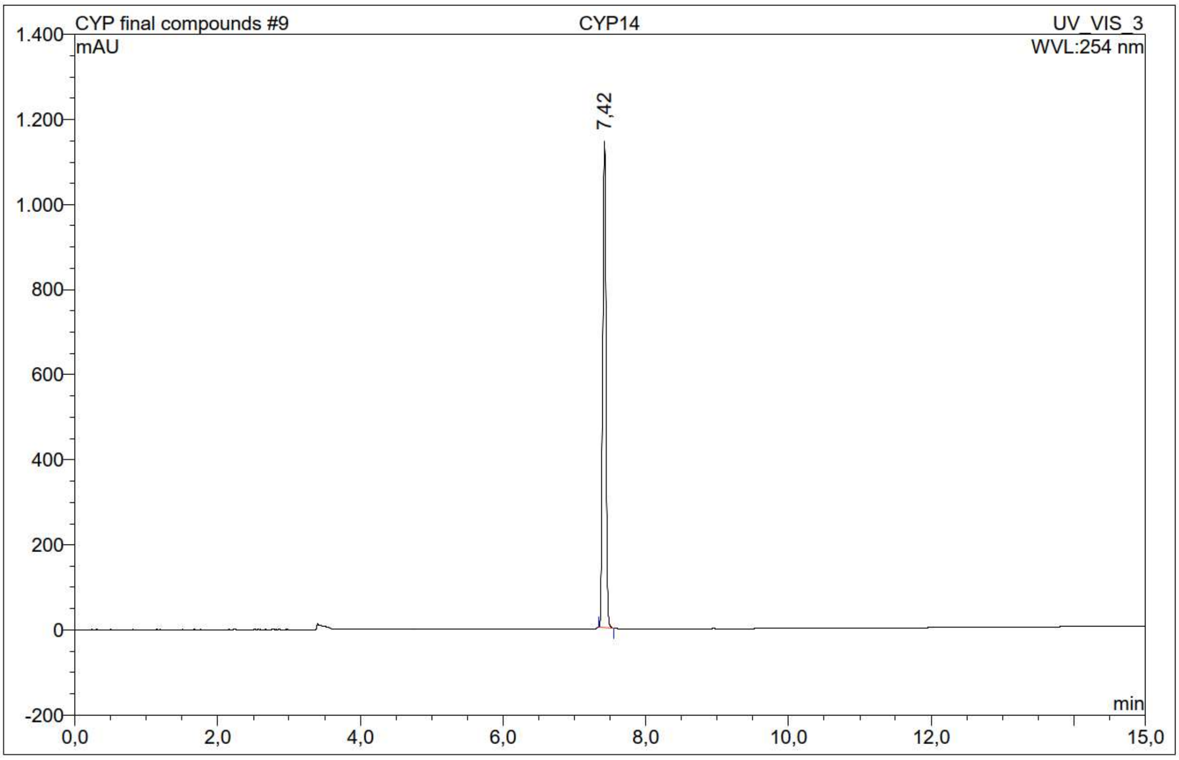

No.	Ret.Time min	Peak Name	Height mAU	Area mAU*min	Rel.Area %	Amount	Resolution(EP)
1	7,42	n.a.	1143,211	59,405	100,00	n.a.	n.a.
Total:			1143,211	59,405	100,00	0,000	

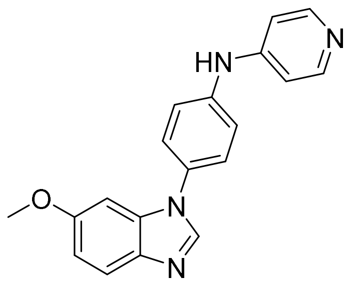

14

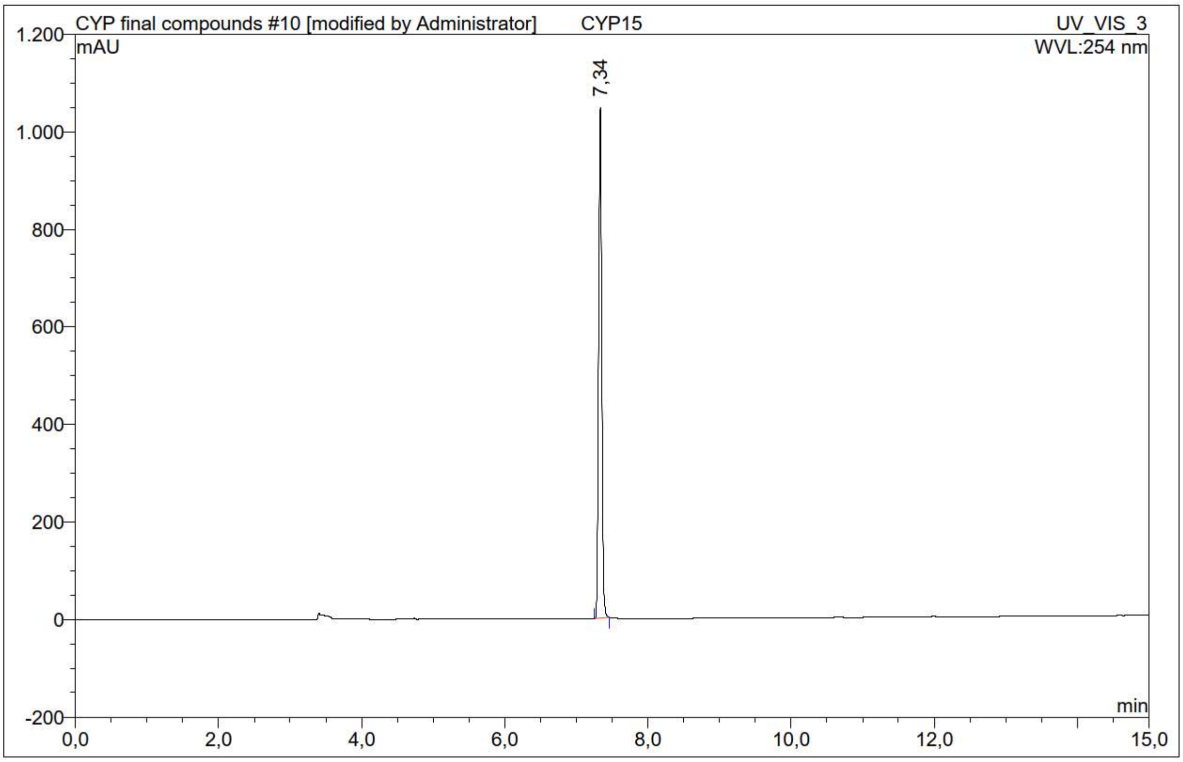

No.	Ret.Time min	Peak Name	Height mAU	Area mAU*min	Rel.Area %	Amount	Resolution(EP)
1	7,34	n.a.	1047,053	57,081	100,00	n.a.	n.a.
Total:			1047,053	57,081	100,00	0,000	

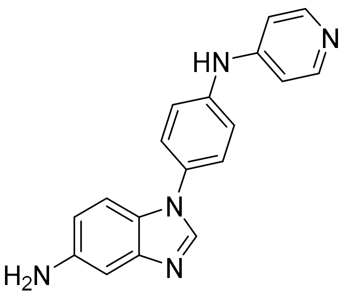

15

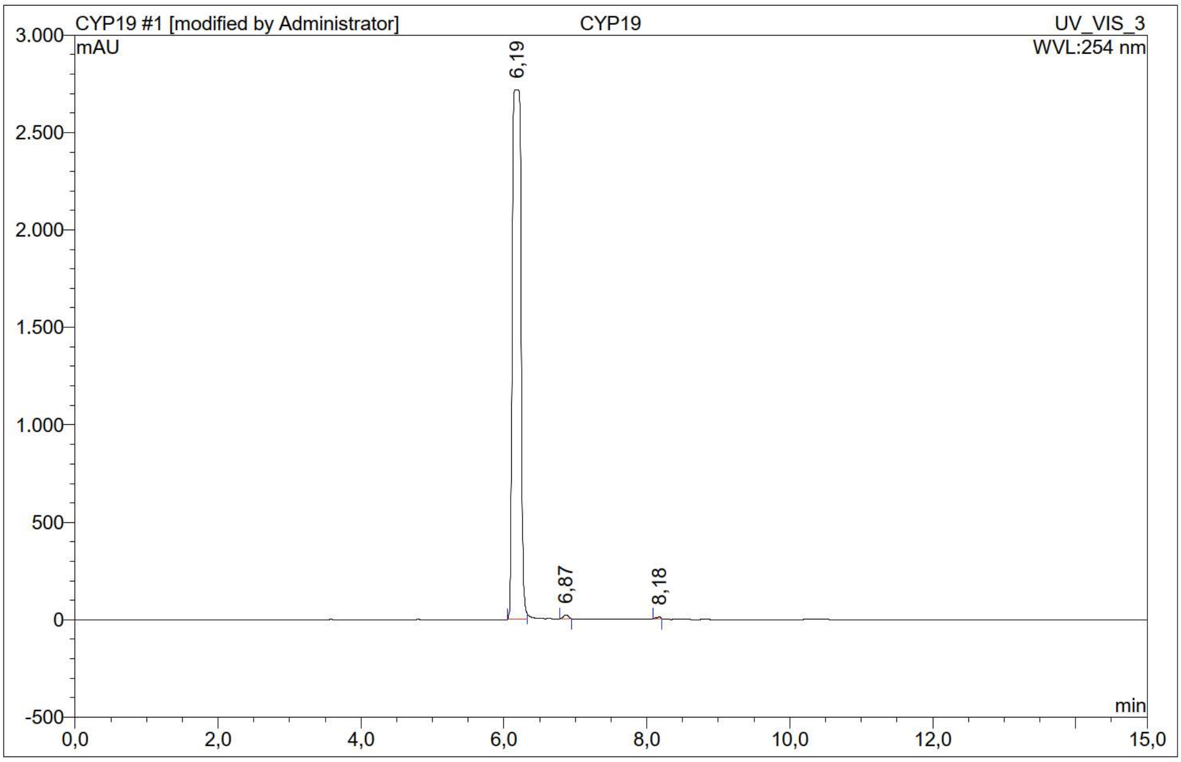

No.	Ret.Time min	Peak Name	Height mAU	Area mAU*min	Rel.Area %	Amount	Resolution(EP)
1	6,19	n.a.	2717,897	379,064	99,36	n.a.	3,72
2	6,87	n.a.	21,414	1,754	0,46	n.a.	12,47
3	8,18	n.a.	11,555	0,692	0,18	n.a.	n.a.
**Total:**			2750,866	381,511	100,00	0,000	

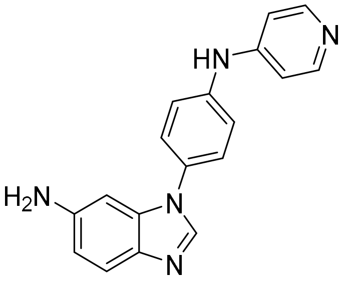

16

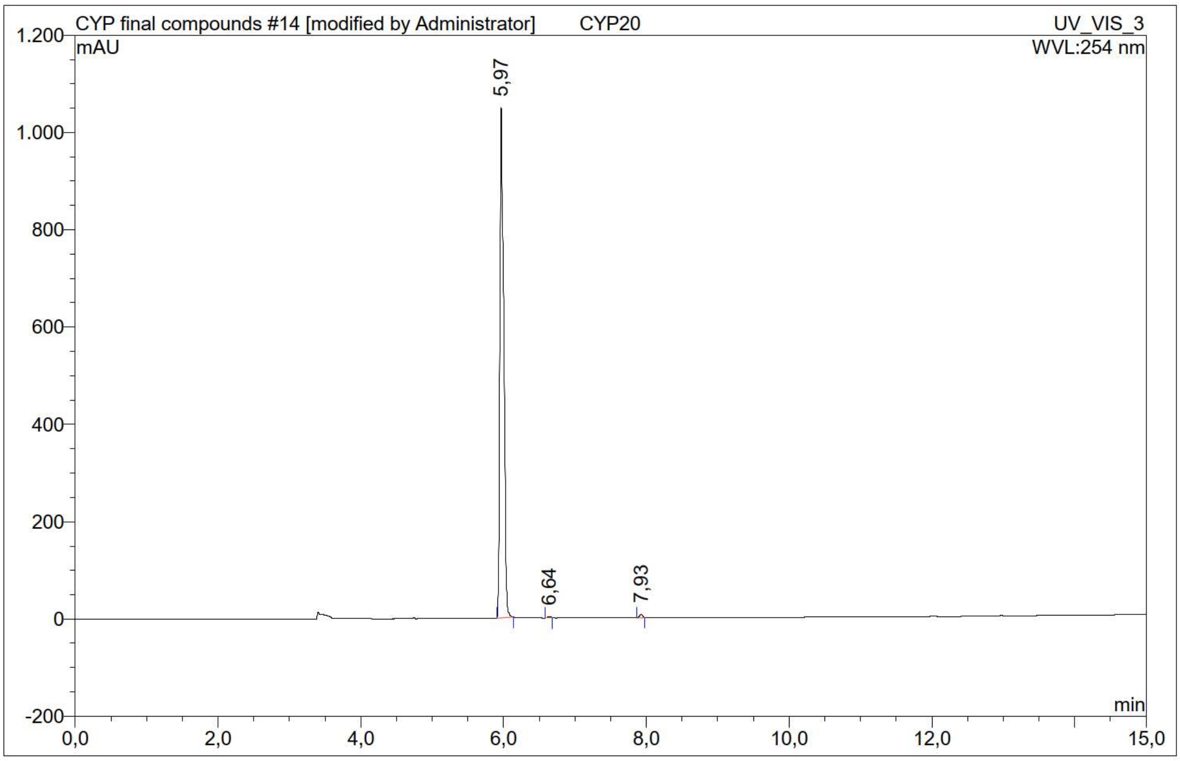

No.	Ret.Time min	Peak Name	Height mAU	Area mAU*min	Rel.Area %	Amount	Resolution(EP)
1	5,97	n.a.	1049,230	67,331	99,27	n.a.	6,49
2	6,64	n.a.	2,575	0,141	0,21	n.a.	15,24
3	7,93	n.a.	7,674	0,353	0,52	n.a.	n.a.
**Total:**			1059,478	67,825	100,00	0,000	

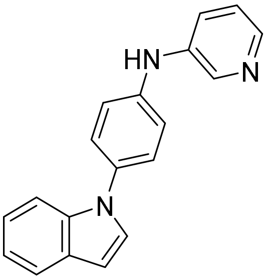

17

Chromatogram and Results

Injection Details

Injection Name:	**TWM013**	*Run Time (min):*	**10.00**
Vial Number:	**RD1**	*Injection Volume:*	**1.00**
Injection Type:	**Unknown**	*Channel:*	**UV_VIS_1**
Calibration Level:		*Wavelength:*	**254**
Instrument Method:	**method7**	*Bandwidth:*	**2**
Processing Method:	**Qualitative**	*Dilution Factor:*	**1.0000**
Injection Date/Time:	**08-Dec-21 16:08**	*Sample Weight:*	**1.0000**

Chromatogram

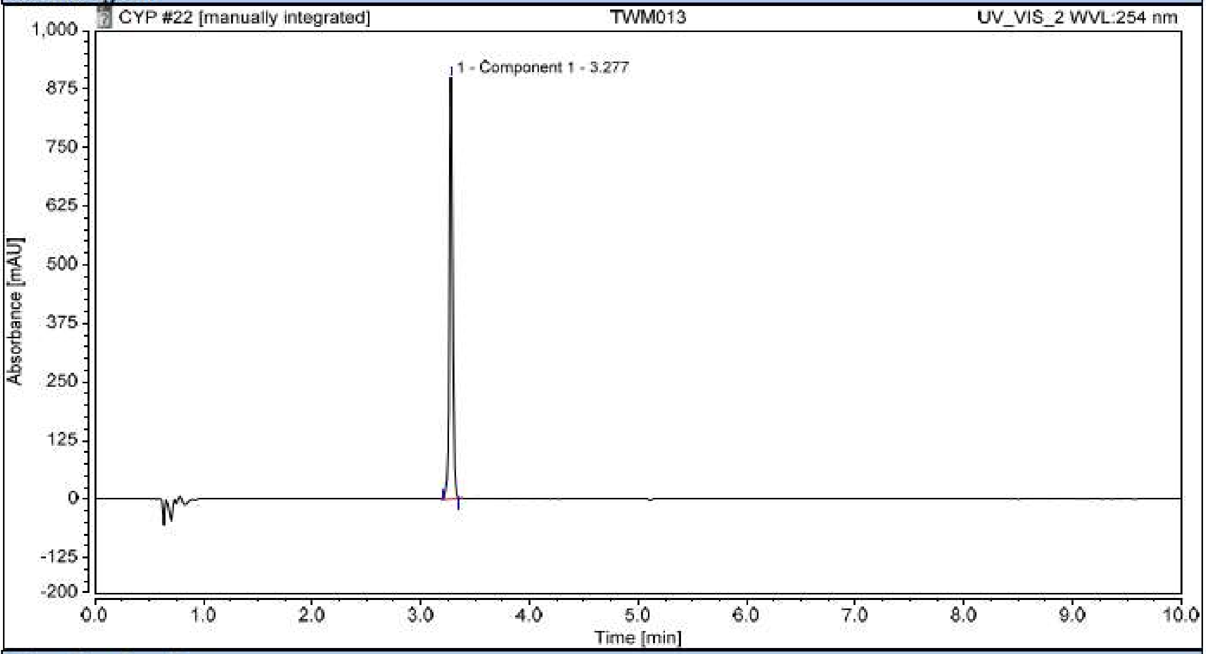

Integration Results

No.	Peak Name	Retention Time min	Area mAU*min	Height mAU	Relative Area %	Relative Height %	Amount
1	Component 1	3.28	30.48	899.61	100.0	100.0	n.a.
**Total:**			**30.48**	**899.61**	**100.0**	**100.0**	

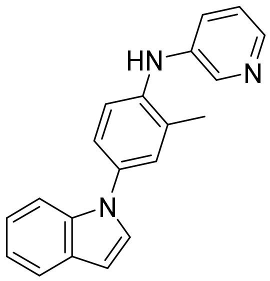

18

Chromatogram and Results

Injection Details

Injection Name:	**TWM015**	*Run Time (min):*	**10.00**
Vial Number:	**RD3**	*Injection Volume:*	**1.00**
Injection Type:	**Unknown**	*Channel:*	**UV_VIS_1**
Calibration Level:		*Wavelength:*	**254**
Instrument Method:	**method7**	*Bandwidth:*	**2**
Processing Method:	**Qualitative**	*Dilution Factor:*	**1.0000**
Injection Date/Time:	**08-Dec-21 16:30**	*Sample Weight:*	**1.0000**

Chromatogram

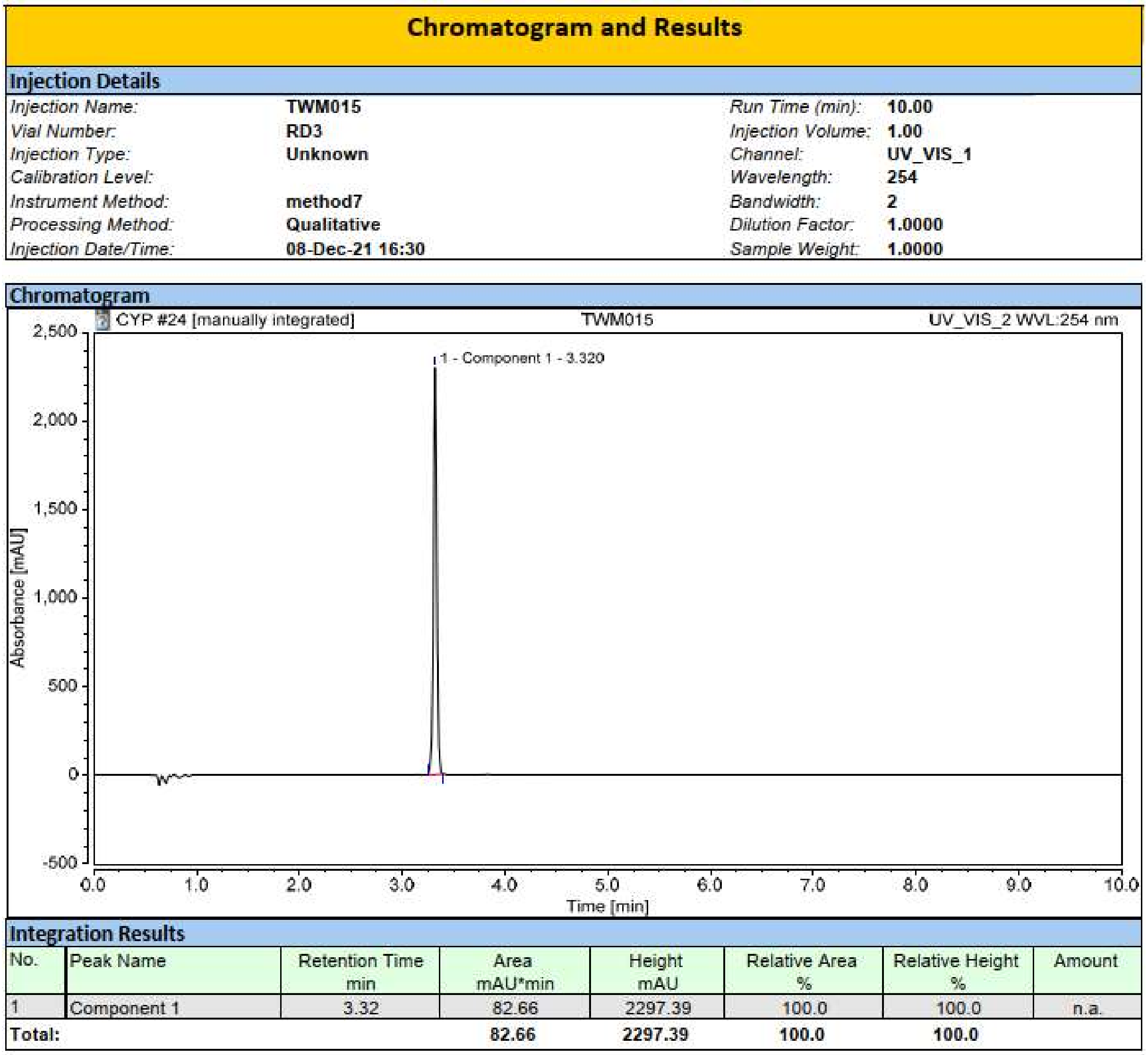

Integration Results

No.	Peak Name	Retention Time min	Area mAU*min	Height mAU	Relative Area %	Relative Height %	Amount
1	Component 1	3.32	82.66	2297.39	100.0	100.0	n.a.
**Total:**			**82.66**	**2297.39**	**100.0**	**100.0**	

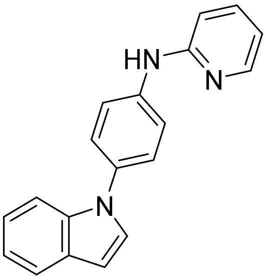

19

Chromatogram and Results

Injection Details

Injection Name:	**TWM014**	*Run Time (min):*	**10.00**
Vial Number:	**RD2**	*Injection Volume:*	**1.00**
Injection Type:	**Unknown**	*Channel:*	**UV_VIS_1**
Calibration Level:		*Wavelength:*	**254**
Instrument Method:	**method7**	*Bandwidth:*	**2**
Processing Method:	**Qualitative**	*Dilution Factor:*	**1.0000**
Injection Date/Time:	**08-Dec-21 16:19**	*Sample Weight:*	**1.0000**

Chromatogram

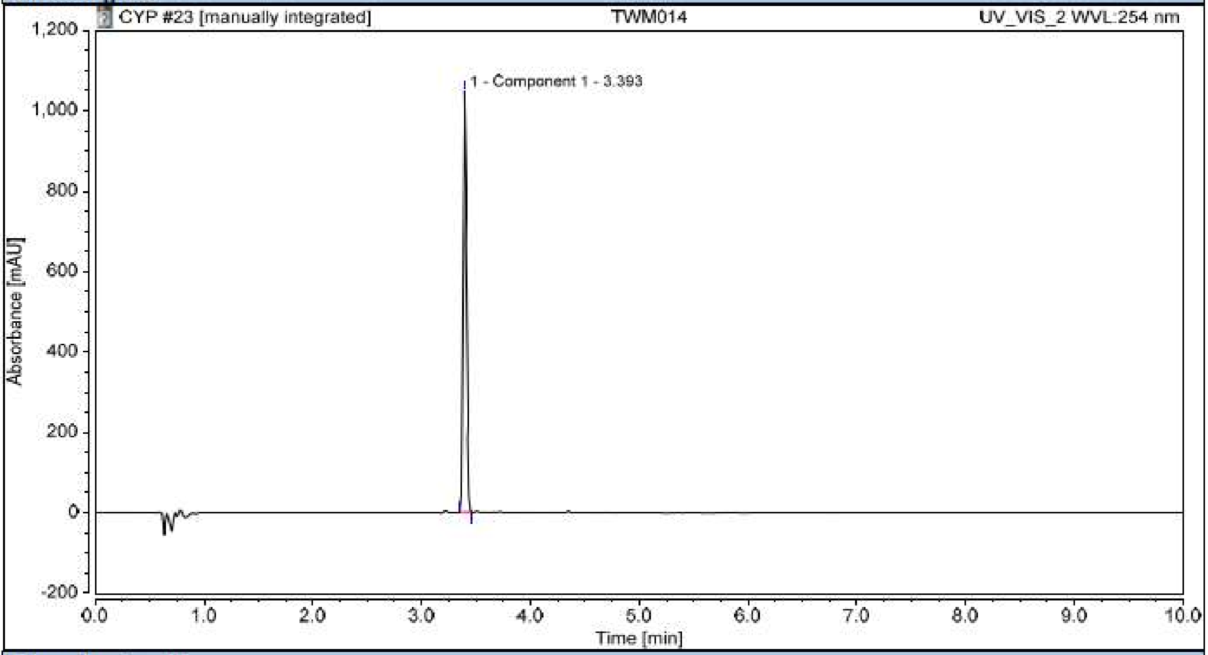

Integration Results

No.	Peak Name	Retention Time min	Area mAU*min	Height mAU	Relative Area %	Relative Height %	Amount
1	Component 1	3.39	36.75	1046.51	100.0	100.0	n.a.
**Total:**			**36.75**	**1046.51**	**100.0**	**100.0**	

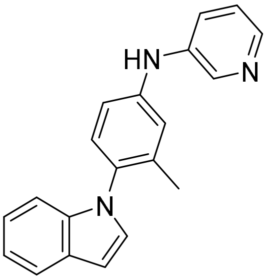

20

Chromatogram and Results

Injection Details

Injection Name:	**TWM016**	*Run Time (min):*	**10.00**
Vial Number:	**RD4**	*Injection Volume:*	**1.00**
Injection Type:	**Unknown**	*Channel:*	**UV_VIS_1**
Calibration Level:		*Wavelength:*	**254**
Instrument Method:	**method7**	*Bandwidth:*	**2**
Processing Method:	**Qualitative**	*Dilution Factor:*	**1.0000**
Injection Date/Time:	**08-Dec-21 16:41**	*Sample Weight:*	**1.0000**

Chromatogram

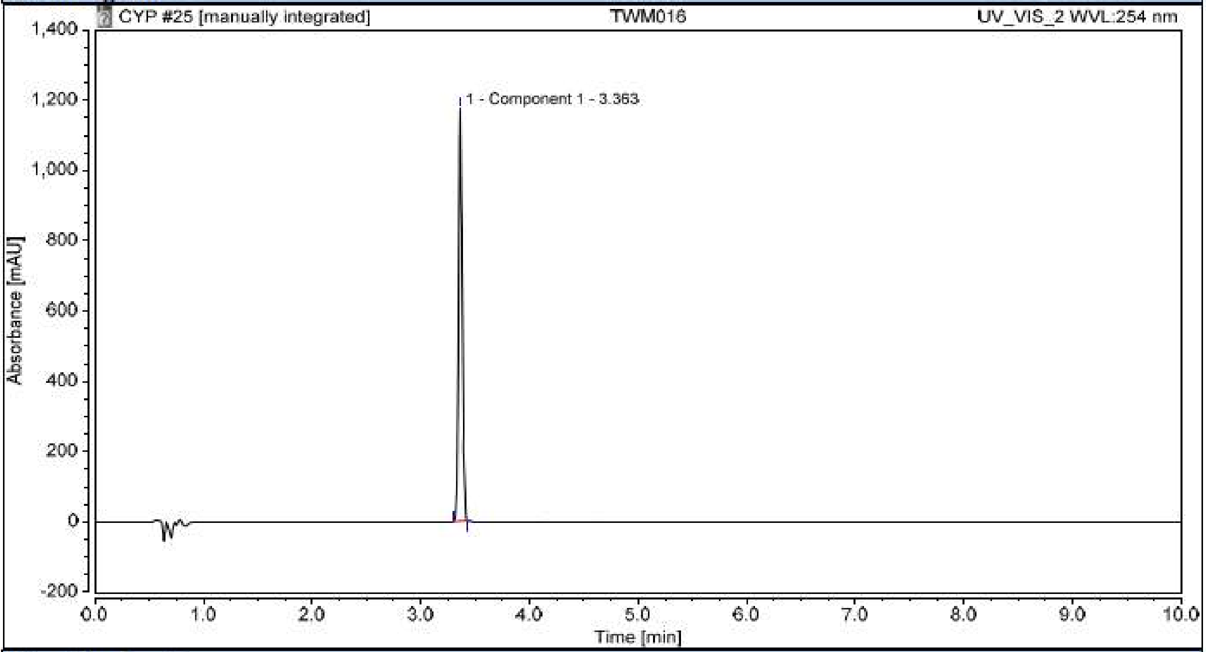

Integration Results

No.	Peak Name	Retention Time min	Area mAU*min	Height mAU	Relative Area %	Relative Height %	Amount
1	Component 1	3.36	41.87	1173.04	100.0	100.0	n.a.
**Total:**			**41.87**	**1173.04**	**100.0**	**100.0**	

CYP17A1 Hydroxylase, CYP17A1 Lyase, CYP3A4 and POR Inhibition Values

	CYP17A1 Hydroxylase Activity		CYP17A1 Lyase Activity		CYP3A4 Activity		POR Activity	
**Comp**	**% of Control**	**SD**	**% of Control**	**SD**	**% of Control**	**SD**	**% of Control**	**SD**
Control	100	0	100	0				
1	55.96	2.30						
2	16.92	1.09	78.47	9.76	103	7	93	9
3	71.10	1.74						
4	39.14	1.85						
5	47.23	3.12						
6	94.32	7.54						
7	100.32	4.07						
8	78.62	0.00						
9	86.94	4.76						
10	97.76	0.91						
11	84.65	2.51						
12	37.26	2.16	77.02	4.29	75	5	103	9
13	71.63	0.00						
14	73.51	0.00						
15	95.62	1.89						
16	89.63	1.83						
17	44.98	15.09						
18	75.37	9.11						
19	64.21	3.87						
20	35.84	0.05	82.17	4.92	117	8	88	16
Abiraterone	16.82	3.64	4.94	2.17				

PC-3 Whole-Cell Assay GR50 Values

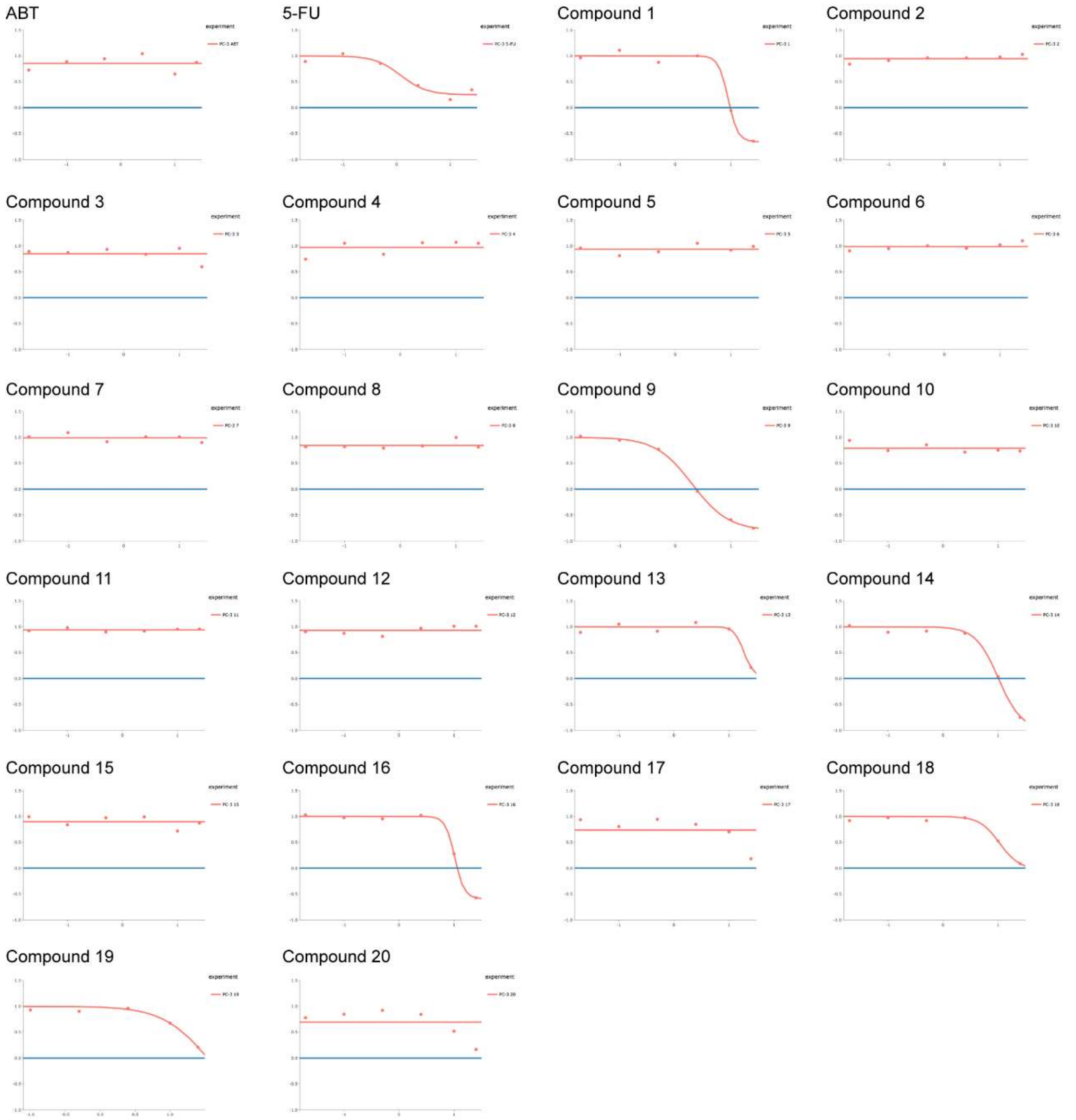

Cell Line	Treatment/cmp.	GR50 (µM)
PC-3	ABT	Inf.
PC-3	5-FU	1.7
PC-3	1	7.6
PC-3	2	Inf.
PC-3	3	Inf.
PC-3	4	Inf.
PC-3	5	Inf.
PC-3	6	Inf.
PC-3	7	Inf.
PC-3	8	Inf.
PC-3	9	1.0

Cell Line	Treatment/cmp.	GR50 (µM)
PC-3	10	Inf.
PC-3	11	Inf.
PC-3	12	Inf.
PC-3	13	18.8
PC-3	14	6.0
PC-3	15	Inf.
PC-3	16	8.9
PC-3	17	Inf.
PC-3	18	10.4
PC-3	19	15.0
PC-3	20	Inf.

Half-maximal Growth Rate Inhibition (GR50) values assessed in the prostate cancer cell line PC-3. Growth Rate Inhibition (GR, red points) for dilution series (0-25 µM) of 5-FU, ABT controls, and compound 1-20 were calculated and plotted against the log10 concentration range. Cytostasis (GR=0) is indicated by blue lines. GR50 curves (red) are based on the average GR from three replicate dilutions series. Fitted curves and calculated GR50 values presented in graphs and tables were produced using GRcalculator (Clark et al. 2017).

References

1. Clark, N.A.; Hafner, M.; Kouril, M.; Williams, E.H.; Muhlich, J.L.; Pilarczyk, M.; Niepel, M.; Sorger, P.K.; Medvedovic, M. GRcalculator: an online tool for calculating and mining dose–response data. *BMC Cancer* **2017**, *17*, 698, doi:10.1186/s12885-017-3689-3.
